# Supplementary material for: Competition and growth among Aedes aegypti larvae: Effects of distributing food inputs over time
Source: PLoS One. 2020 Oct 2;15(10):e0234676. doi: 10.1371/journal.pone.0234676 (PMC7531853; doi:10.1371/journal.pone.0234676)

S4 Fig. Experiment 1. Scatterplot of age at pupation (days) versus the number of males pupating on each day.


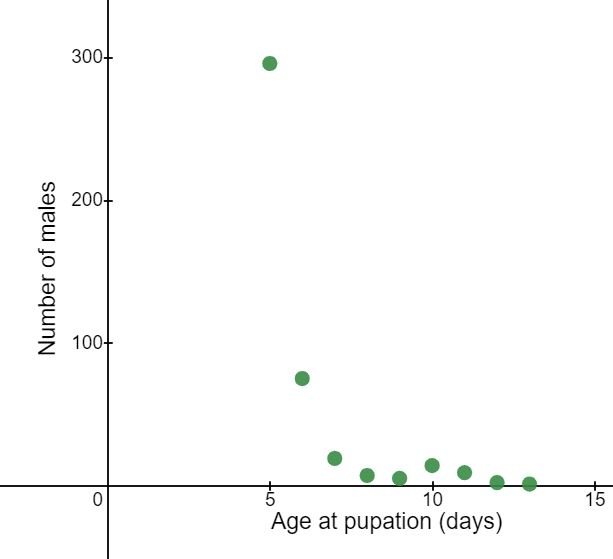

Supplement: S4 Fig — Scatterplot of age at pupation (days) versus the number of males pupating on each day. (DOCX) [file pone.0234676.s007.docx]
