# Supplementary material for: Competition and growth among Aedes aegypti larvae: Effects of distributing food inputs over time
Source: PLoS One. 2020 Oct 2;15(10):e0234676. doi: 10.1371/journal.pone.0234676 (PMC7531853; doi:10.1371/journal.pone.0234676)
Supplement: S5 Fig — 3D visualization of Survival for FxDxT. (DOCX) [file pone.0234676.s008.docx]

S5 Fig. Experiment 1. 3D visualization of Survival for FxDxT.


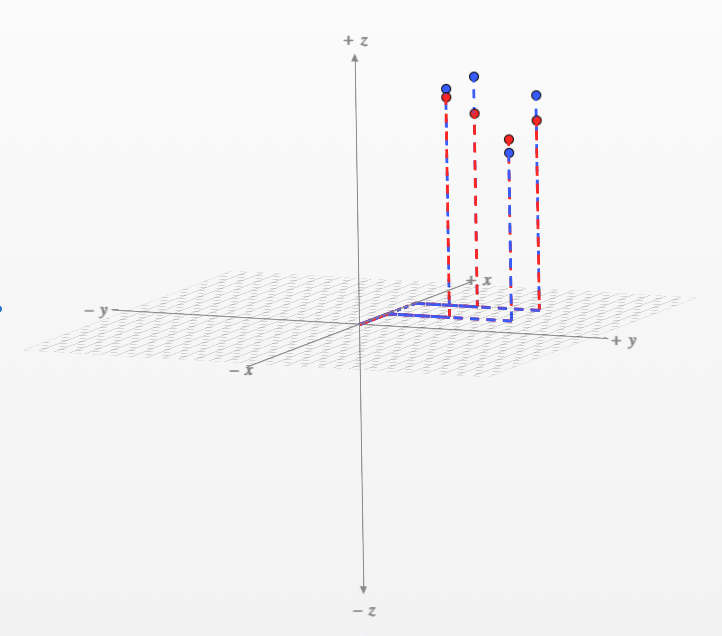


The horizontal axis (y) is density, 4 or 8 larvae per test tube. The axis receding into the plane of the page (x) is total food, 16 mg or 32 mg per test tube. The vertical axis (z) is the dependent variable, Survival (arc sine transformed % survival per test tube). The axes are not to the same scale; the food axis has been compressed relative to density and the dependent variable axis has been expanded to enhance the differences among the mean values. The red circles represent the 3 day timespan and the blue circles represent the 6 day timespan. The dotted lines serve to align the blue and red circles for the same treatments. From left to right, the four competitive environments are: low food, low density (intermediate competition); high food, low density (least competition); low food, high density (most competition); and high food, high density (intermediate competition).

Survival is affected by all three factors, food, density and timespan. Survival is higher on the 6 day timespan (blue circles) except in the test tubes with the most competition. The largest difference between the two timespans is in the test tubes with the least competition, followed by the high food, high density ones; these are both at the higher food level. See the text for further explanation.
