# Supplementary material for: Competition and growth among Aedes aegypti larvae: Effects of distributing food inputs over time
Source: PLoS One. 2020 Oct 2;15(10):e0234676. doi: 10.1371/journal.pone.0234676 (PMC7531853; doi:10.1371/journal.pone.0234676)
Supplement: S6 Fig — 3D visualization of Prime female mass for FxDxT. (DOCX) [file pone.0234676.s009.docx]

S6 Fig. Experiment 1. 3D visualization of Prime female mass for FxDxT.


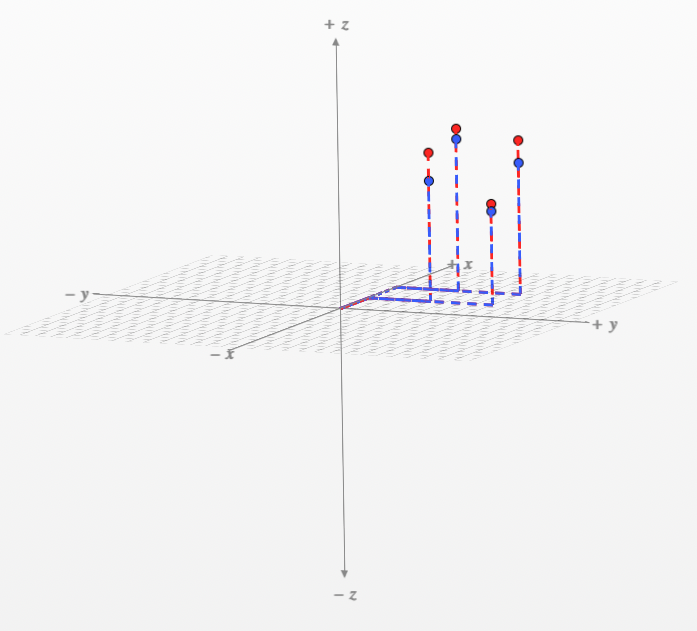


The horizontal axis (y) is density, 4 or 8 larvae per test tube. The axis receding into the plane of the page (x) is total food, 16 mg or 32 mg per test tube. The vertical axis (z) is the dependent variable, Prime female mass (mg). The axes are not to the same scale; the food axis has been compressed relative to density and the dependent variable axis has been expanded to enhance the differences among the mean values. The red circles represent the 3 day timespan and the blue circles represent the 6 day timespan. The dotted lines serve to align the blue and red circles for the same treatments. From left to right, the four competitive environments are: low food, low density (intermediate competition); high food, low density (least competition); low food, high density (most competition); and high food, high density (intermediate competition).

Prime female mass is always greater for the 3 day timespan (red circles) than for the 6 day timespan (blue circles). There are larger differences in mass due to timespan at intermediate levels of competition rather than in either the test tubes with the most competition or the least competition. See the text for further explanation.
