# Supplementary material for: Competition and growth among Aedes aegypti larvae: Effects of distributing food inputs over time
Source: PLoS One. 2020 Oct 2;15(10):e0234676. doi: 10.1371/journal.pone.0234676 (PMC7531853; doi:10.1371/journal.pone.0234676)
Supplement: S7 Fig — 3D visualization of Prime female age for FxDxT. (DOCX) [file pone.0234676.s010.docx]

S7 Fig. Experiment 1. 3D visualization of Prime female age for FxDxT.


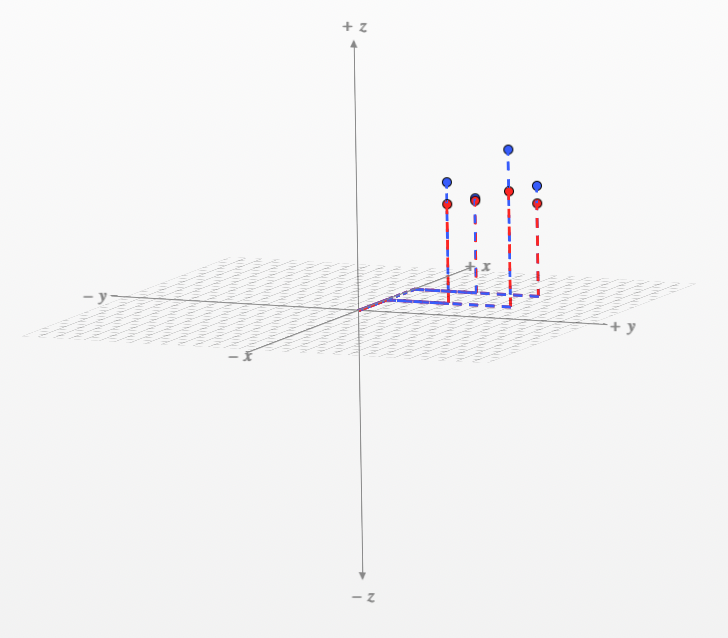


The horizontal axis (y) is density, 4 or 8 larvae per test tube. The axis receding into the plane of the page (x) is total food, 16 mg or 32 mg per test tube. The vertical axis (z) is the dependent variable, Prime female age (days). The axes are not to the same scale; the food axis has been compressed relative to density and the dependent variable axis has been expanded to enhance the differences among the mean values. The red circles represent the 3 day timespan and the blue circles represent the 6 day timespan. The dotted lines serve to align the blue and red circles for the same treatments. From left to right, the four competitive environments are: low food, low density (intermediate competition); high food, low density (least competition); low food, high density (most competition); and high food, high density (intermediate competition).

The Prime female age is always greater for the 6 day timespan (blue circles). The interaction between food, density and timespan is due to the differences between the two timespan treatments at the different levels of competition. The ages at pupation are similar in the test tubes with the least competition, somewhat larger at high food and high density (right hand pair of circles), even larger at low food and low density (left hand pair of circles), and largest in the test tubes with the most competition. See the text for further explanation.
