# Supplementary material for: Competition and growth among Aedes aegypti larvae: Effects of distributing food inputs over time
Source: PLoS One. 2020 Oct 2;15(10):e0234676. doi: 10.1371/journal.pone.0234676 (PMC7531853; doi:10.1371/journal.pone.0234676)
Supplement: S8 Fig — 3D visualization of estimated growth rate for Prime females for FxDxT. (DOCX) [file pone.0234676.s011.docx]

S8 Fig. Experiment 1. 3D visualization of estimated growth rate for Prime females for FxDxT.


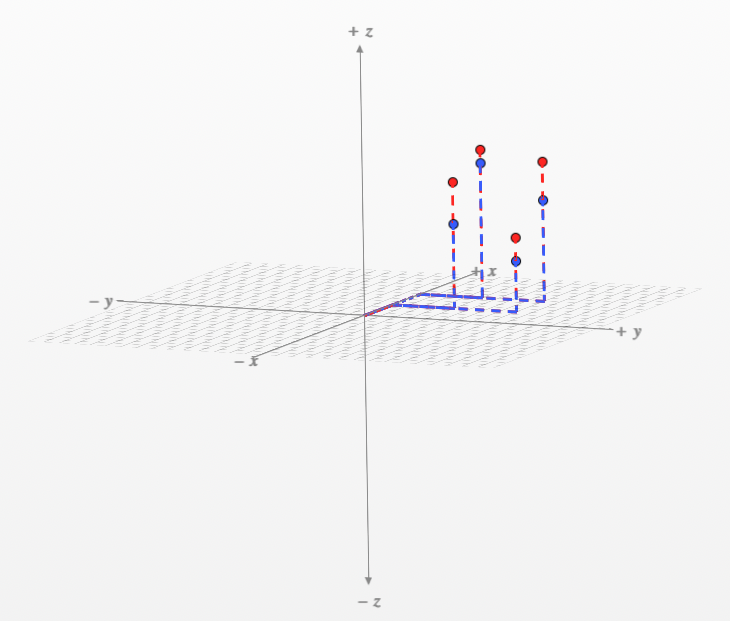


The horizontal axis (y) is density, 4 or 8 larvae per test tube. The axis receding into the plane of the page (x) is total food, 16 mg or 32 mg per test tube. The vertical axis (z) is the dependent variable, estimated growth rate for Prime females (mg/day). The axes are not to the same scale; the food axis has been compressed relative to density and the dependent variable axis has been expanded to enhance the differences among the mean values. The red circles represent the 3 day timespan and the blue circles represent the 6 day timespan. The dotted lines serve to align the blue and red circles for the same treatments. From left to right, the four competitive environments are: low food, low density (intermediate competition); high food, low density (least competition); low food, high density (most competition); and high food, high density (intermediate competition).

Growth rates are higher at the 3 day timespan (red circles) than at the 6 day timespan (blue circles) for all levels of competition. The effect of the timespan treatment (the vertical distance between the red circle and the blue circle) is greater at the intermediate levels of competition (extreme right and left pairs of circles) and least in the test tubes with the least competition (pair of circles second from the left). See the text for further explanation.
