# Supplementary material for: Competition and growth among Aedes aegypti larvae: Effects of distributing food inputs over time
Source: PLoS One. 2020 Oct 2;15(10):e0234676. doi: 10.1371/journal.pone.0234676 (PMC7531853; doi:10.1371/journal.pone.0234676)
Supplement: S9 Fig — 3D visualization of Average female mass for FxDxT. (DOCX) [file pone.0234676.s012.docx]

S9 Fig. Experiment 1. 3D visualization of Average female mass for FxDxT.


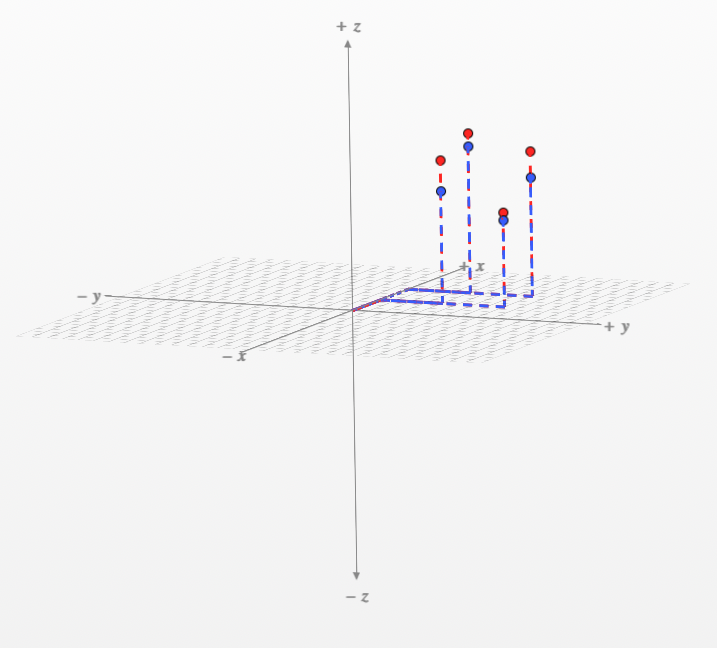


The horizontal axis (y) is density, 4 or 8 larvae per test tube. The axis receding into the plane of the page (x) is total food, 16 mg or 32 mg per test tube. The vertical axis (z) is the dependent variable, Average female mass (mg). The axes are not to the same scale; the food axis has been compressed relative to density and the dependent variable axis has been expanded to enhance the differences among the mean values. The red circles represent the 3 day timespan and the blue circles represent the 6 day timespan. The dotted lines serve to align the blue and red circles for the same treatments. From left to right, the four competitive environments are: low food, low density (intermediate competition); high food, low density (least competition); low food, high density (most competition); and high food, high density (intermediate competition).

The Average female mass is always greater for the 3 day timespan (red circles). Food, density and timespan all affect the Average female mass. The interaction is due to the different effect that timespan has at different levels of competition. Timespan has a greater effect on Average female mass at the two intermediate competition levels (extreme right and left pairs of circles) compared to the most and least competition levels (two central pairs of circles). See the text for further explanation.
