# Supplementary material for: Competition and growth among Aedes aegypti larvae: Effects of distributing food inputs over time
Source: PLoS One. 2020 Oct 2;15(10):e0234676. doi: 10.1371/journal.pone.0234676 (PMC7531853; doi:10.1371/journal.pone.0234676)
Supplement: S10 Fig — 3D visualization of Prime female mass MINUS Average female mass for FxDxT. (DOCX) [file pone.0234676.s013.docx]

S10 Fig. Experiment 1. 3D visualization of Prime female mass MINUS Average female mass for FxDxT.


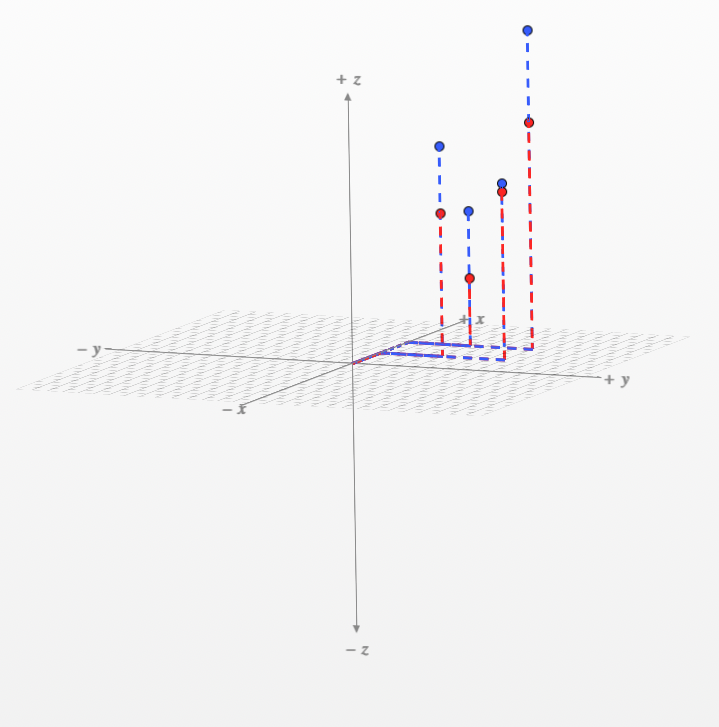


The horizontal axis (y) is density, 4 or 8 larvae per test tube. The axis receding into the plane of the page (x) is total food, 16 mg or 32 mg per test tube. The vertical axis (z) is the dependent variable, Prime female mass MINUS Average female mass (mg). The axes are not to the same scale; the food axis has been compressed relative to density and the dependent variable axis has been expanded to enhance the differences among the mean values. The red circles represent the 3 day timespan and the blue circles represent the 6 day timespan. The dotted lines serve to align the blue and red circles for the same treatments. From left to right, the four competitive environments are: low food, low density (intermediate competition); high food, low density (least competition); low food, high density (most competition); and high food, high density (intermediate competition).

The largest differences between Prime female mass and Average female mass are due to the 6 day timespan (blue circles). Most of the differences between the Prime female mass and the Average female mass lie in the range 0.16 mg to 0.27 mg, with two outliers at the top and bottom. The smallest difference is in the least competition treatment with the 3 day timespan (red circle, second from left). The largest difference is in the high food, high density treatment with the 6 day timespan (blue circle, extreme right). See the text for further explanation.
