# Supplementary material for: Competition and growth among Aedes aegypti larvae: Effects of distributing food inputs over time
Source: PLoS One. 2020 Oct 2;15(10):e0234676. doi: 10.1371/journal.pone.0234676 (PMC7531853; doi:10.1371/journal.pone.0234676)
Supplement: S11 Fig — 3D visualization of Prime male mass for FxDxT. (DOCX) [file pone.0234676.s014.docx]

S11 Fig. Experiment 1. 3D visualization of Prime male mass for FxDxT.


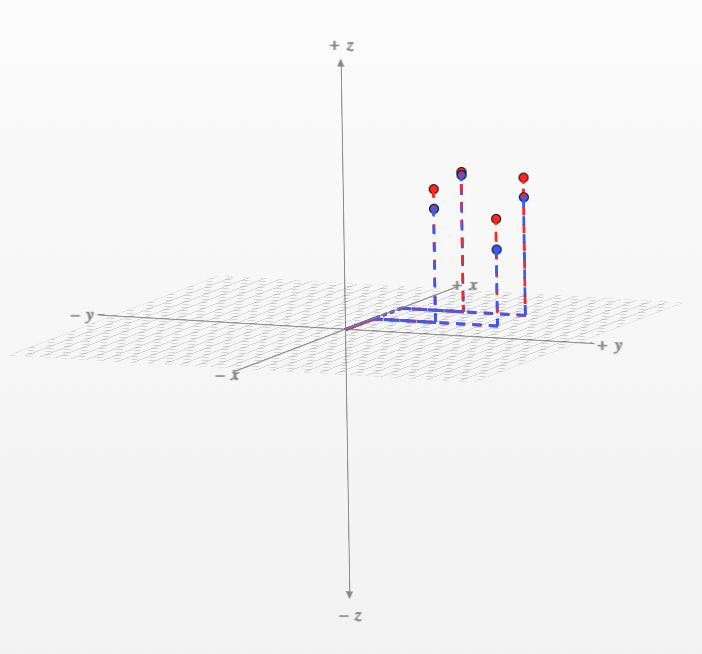


The horizontal axis (y) is density, 4 or 8 larvae per test tube. The axis receding into the plane of the page (x) is total food, 16 mg or 32 mg per test tube. The vertical axis (z) is the dependent variable, Prime male mass (mg). The axes are not to the same scale; the food axis has been compressed relative to density and the dependent variable axis has been expanded to enhance the differences among the mean values. The red circles represent the 3 day timespan and the blue circles represent the 6 day timespan. The dotted lines serve to align the blue and red circles for the same treatments. From left to right, the four competitive environments are: low food, low density (intermediate competition); high food, low density (least competition); low food, high density (most competition); and high food, high density (intermediate competition).

Prime male mass is always greater for the 3 day timespan (red circles). Food, density and timespan all affect the Prime male mass. The interaction is due to the different effects of timespan at different levels of competition. The Prime male mass is not much affected by timespan in the test tubes with the least competition (the pair of circles second from the left). It is more affected by timespan in the test tubes with intermediate competition (the two pairs of circles on the extreme left and right) and most affected in the test tubes with the most competition (the pair of circles second from the right). See the text for further explanation.
