# Supplementary material for: Competition and growth among Aedes aegypti larvae: Effects of distributing food inputs over time
Source: PLoS One. 2020 Oct 2;15(10):e0234676. doi: 10.1371/journal.pone.0234676 (PMC7531853; doi:10.1371/journal.pone.0234676)
Supplement: S12 Fig — 3D visualization of Prime male age for FxDxT. (DOCX) [file pone.0234676.s015.docx]

S12 Fig. Experiment 1. 3D visualization of Prime male age for FxDxT.


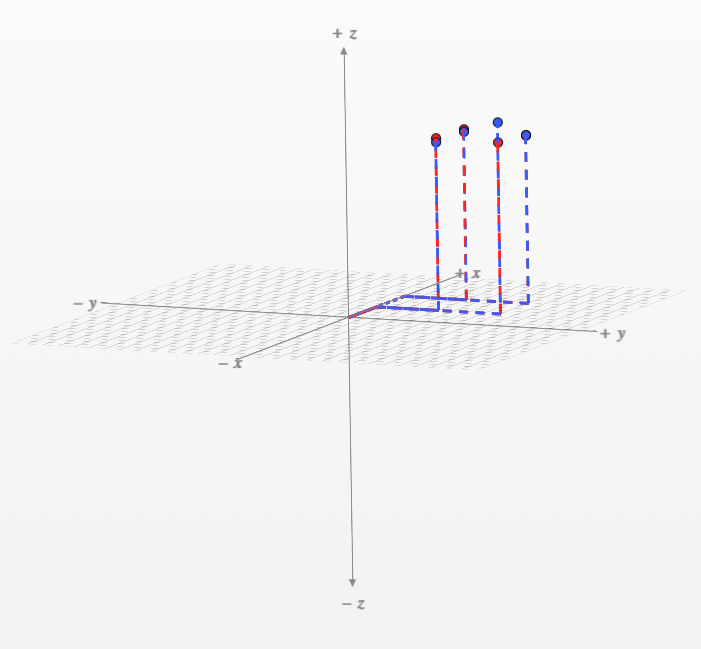


The horizontal axis (y) is density, 4 or 8 larvae per test tube. The axis receding into the plane of the page (x) is total food, 16 mg or 32 mg per test tube. The vertical axis (z) is the dependent variable, Prime male age (days). The axes are not to the same scale; the food axis has been compressed relative to density and the dependent variable axis has been expanded to enhance the differences among the mean values. The red circles represent the 3 day timespan and the blue circles represent the 6 day timespan. The dotted lines serve to align the blue and red circles for the same treatments. From left to right, the four competitive environments are: low food, low density (intermediate competition); high food, low density (least competition); low food, high density (most competition); and high food, high density (intermediate competition).

The Prime male age at pupation is similar for both timespans across three of the competition treatments. The 6 day timespan (blue circle) results in a later pupation for the Prime male in the test tubes with the most competition. It is difficult to see on the graph, but the 3 day timespan red circles) is slightly later for the two low density treatments (least competition and low food, low density, the two pairs of circles on the left). See the text for further explanation.
