# Supplementary material for: Competition and growth among Aedes aegypti larvae: Effects of distributing food inputs over time
Source: PLoS One. 2020 Oct 2;15(10):e0234676. doi: 10.1371/journal.pone.0234676 (PMC7531853; doi:10.1371/journal.pone.0234676)
Supplement: S14 Fig — 3D visualization of Prime male mass MINUS Average male mass for FxDxT. (DOCX) [file pone.0234676.s017.docx]

S14 Fig. Experiment 1. 3D visualization of Prime male mass MINUS Average male mass for FxDxT.


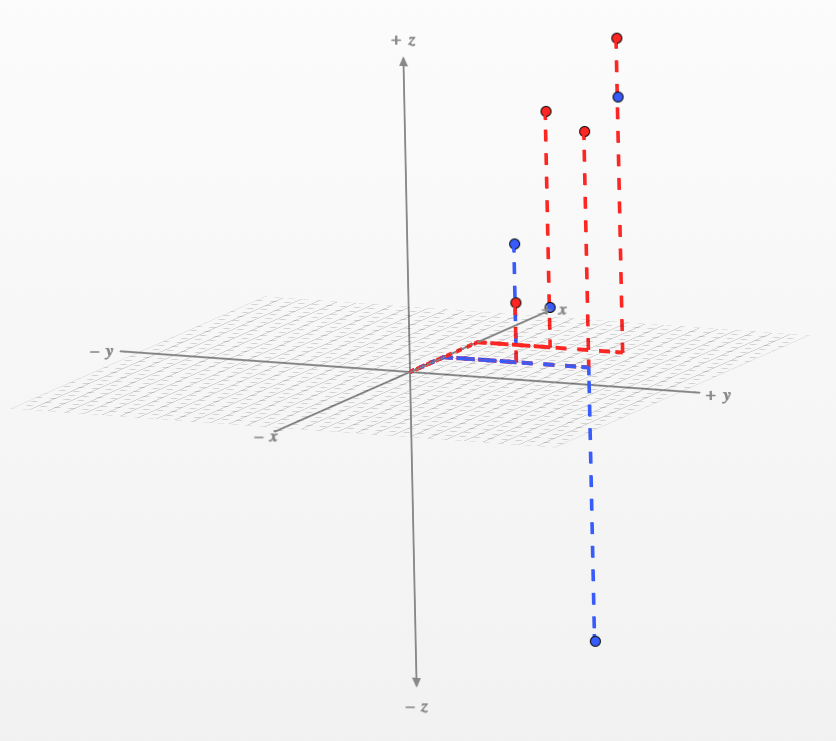


The horizontal axis (y) is density, 4 or 8 larvae per test tube. The axis receding into the plane of the page (x) is total food, 16 mg or 32 mg per test tube. The vertical axis (z) is the dependent variable, Prime male mass MINUS Average male mass (mg). The axes are not to the same scale; the food axis has been compressed relative to density and the dependent variable axis has been expanded to enhance the differences among the mean values. The red circles represent the 3 day timespan and the blue circles represent the 6 day timespan. The dotted lines serve to align the blue and red circles for the same treatments. From left to right, the four competitive environments are: low food, low density (intermediate competition); high food, low density (least competition); low food, high density (most competition); and high food, high density (intermediate competition).

The difference between the Prime male mass and the Average male mass is an indication of the relative advantage of the Prime male over the non-Prime males. At the high food levels the Prime male is larger at the 3 day timespan (back row, red circles) than at the 6 day timespan (blue circles). For the low food, low density treatment (extreme left), the Prime male is larger at the 6 day timespan (blue circle) than the 3 day timespan. For the most competition treatment (second from right) the Average male mass is greater than the Prime male mass and the difference is negative for the 6 day timespan (blue circle). See the text for further explanation.
