# Supplementary material for: Competition and growth among Aedes aegypti larvae: Effects of distributing food inputs over time
Source: PLoS One. 2020 Oct 2;15(10):e0234676. doi: 10.1371/journal.pone.0234676 (PMC7531853; doi:10.1371/journal.pone.0234676)
Supplement: S15 Fig — 3D visualization of estimated growth rates for both Prime females and Prime males for FxDxT. (DOCX) [file pone.0234676.s018.docx]

S15 Fig. Experiment 1. 3D visualization of estimated growth rates for both Prime females and Prime males for FxDxT.


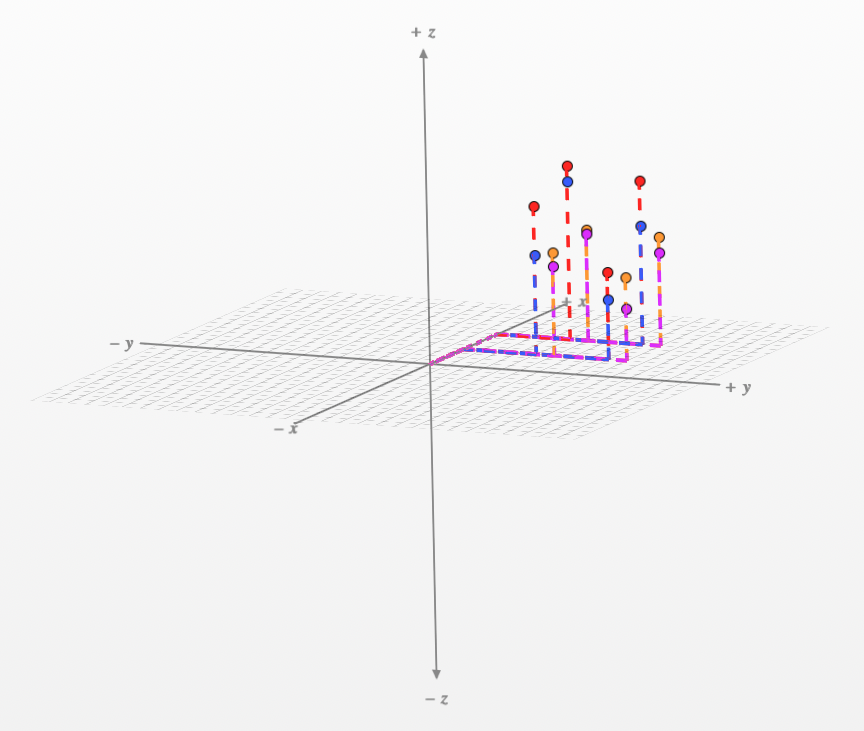


The horizontal axis (y) is density, 4 or 8 larvae per test tube. The orange and purple lines are offset to enhance the clarity of the representation; they stand for the same densities as the red and blue lines. The axis receding into the plane of the page (x) is total food, 16 mg or 32 mg per test tube. The vertical axis (z) is the dependent variable, growth rate (mg/day). The axes are not to the same scale; the food axis has been compressed relative to density and the dependent variable axis has been expanded to enhance the differences among the mean values. The red circles represent the 3 day timespan and the blue circles represent the 6 day timespan for Prime females. The orange circles represent the 3 day timespan and the purple circles represent the 6 day timespan for Prime males. The dotted lines serve to align the blue and red circles, and the orange and purple circles for the same treatments. From left to right, the four competitive environments are: low food, low density (intermediate competition); high food, low density (least competition); low food, high density (most competition); and high food, high density (intermediate competition).

Growth rates for the 3 day timespan (red circles for Prime females, orange circles for Prime males) are always greater than for the 6 day timespan (blue circles for Prime females, purple circles for Prime males). Prime female growth rates for the 3 day timespan (red circles) are always greater than Prime male growth rates (orange and purple circles). The Prime female growth rate at the 6 day timespan (blue circles) is only greater than the Prime male growth rate (orange circle) at high food (back row) and only much greater for the least competition treatment (third and fourth pairs of circles from the left). Overall the growth rates of Prime males (orange and purple circles) are compressed relative to that of females (red and blue circles); however, the growth rates of both Prime females and males are compressed in the most competition treatments (third and fourth pairs of circles from the right) relative to the other treatments. See the text for further explanation.
