# Supplementary material for: Competition and growth among Aedes aegypti larvae: Effects of distributing food inputs over time
Source: PLoS One. 2020 Oct 2;15(10):e0234676. doi: 10.1371/journal.pone.0234676 (PMC7531853; doi:10.1371/journal.pone.0234676)
Supplement: S18 Fig — 3D visualization of Average female mass for FxDxA. (DOCX) [file pone.0234676.s021.docx]

S18 Fig. Experiment 1. 3D visualization of Average female mass for FxDxA.


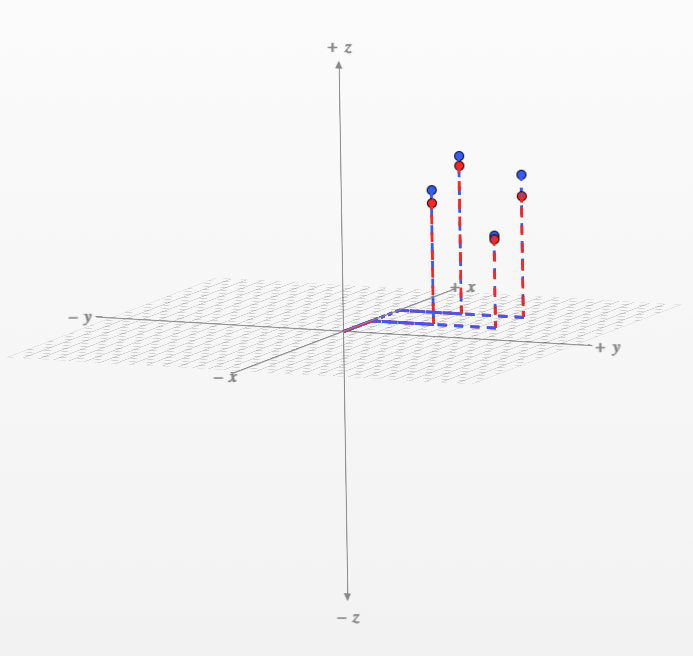


The horizontal axis (y) is density, 4 or 8 larvae per test tube. The axis receding into the plane of the page (x) is total food, 16 mg or 32 mg per test tube. The vertical axis (z) is the dependent variable, Average female mass (mg). The axes are not to the same scale; the food axis has been compressed relative to density and the dependent variable axis has been expanded to enhance the differences among the mean values. The red circles represent the 2 aliquot treatment and the blue circles represent the 4 aliquot treatment. The dotted lines serve to align the blue and red circles for the same treatments. From left to right, the four competitive environments are: low food, low density (intermediate competition); high food, low density (least competition); low food, high density (most competition); and high food, high density (intermediate competition).

The values of the Average female mass are stratified into 3 groups. The three largest Average female masses are the two circles in the least competition treatment (one red, one blue, second from left) and the blue circle (4 aliquots) in the high food, high density treatment (extreme right). The middle group of Average female masses are the red circle (2 aliquots) in the high food, high density treatment (extreme right) and the two circles in the low food, low density treatment (one red, one blue, extreme left). The smallest Average female masses are the two circles in the most competition treatment (one red, one blue, second from right). See the text for further explanation.
