# Supplementary material for: Competition and growth among Aedes aegypti larvae: Effects of distributing food inputs over time
Source: PLoS One. 2020 Oct 2;15(10):e0234676. doi: 10.1371/journal.pone.0234676 (PMC7531853; doi:10.1371/journal.pone.0234676)
Supplement: S19 Fig — 3D visualization of Prime female mass MINUS Average female mass for FxDxA. (DOCX) [file pone.0234676.s022.docx]

S19 Fig. Experiment 1. 3D visualization of Prime female mass MINUS Average female mass for FxDxA.


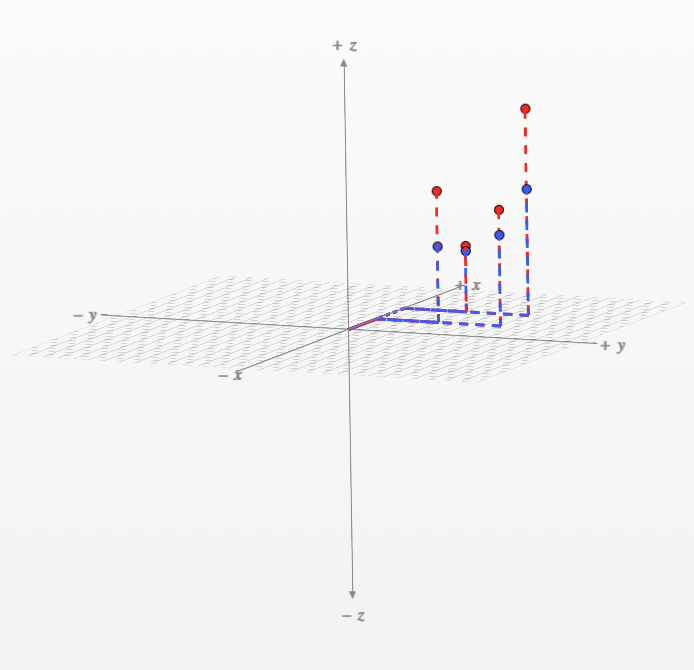


The horizontal axis (y) is density, 4 or 8 larvae per test tube. The axis receding into the plane of the page (x) is total food, 16 mg or 32 mg per test tube. The vertical axis (z) is the dependent variable, Prime female mass MINUS Average female mass (mg). The axes are not to the same scale; the food axis has been compressed relative to density and the dependent variable axis has been expanded to enhance the differences among the mean values. The red circles represent the 2 aliquot treatment and the blue circles represent the 4 aliquot treatment. The dotted lines serve to align the blue and red circles for the same treatments. From left to right, the four competitive environments are: low food, low density (intermediate competition); high food, low density (least competition); low food, high density (most competition); and high food, high density (intermediate competition).

The difference between the Prime female mass and the Average female mass is always greater with the 2 aliquot treatment (red circles). The differences are smallest in the test tubes with the least competition (second from left) where all the females grow largest. The differences increase at higher levels of competition. See the text for further explanation.
