# Supplementary material for: Competition and growth among Aedes aegypti larvae: Effects of distributing food inputs over time
Source: PLoS One. 2020 Oct 2;15(10):e0234676. doi: 10.1371/journal.pone.0234676 (PMC7531853; doi:10.1371/journal.pone.0234676)
Supplement: S20 Fig — 3D visualization of estimated Prime female growth rate for FxDxA. (DOCX) [file pone.0234676.s023.docx]

S20 Fig. Experiment 1. 3D visualization of estimated Prime female growth rate for FxDxA.
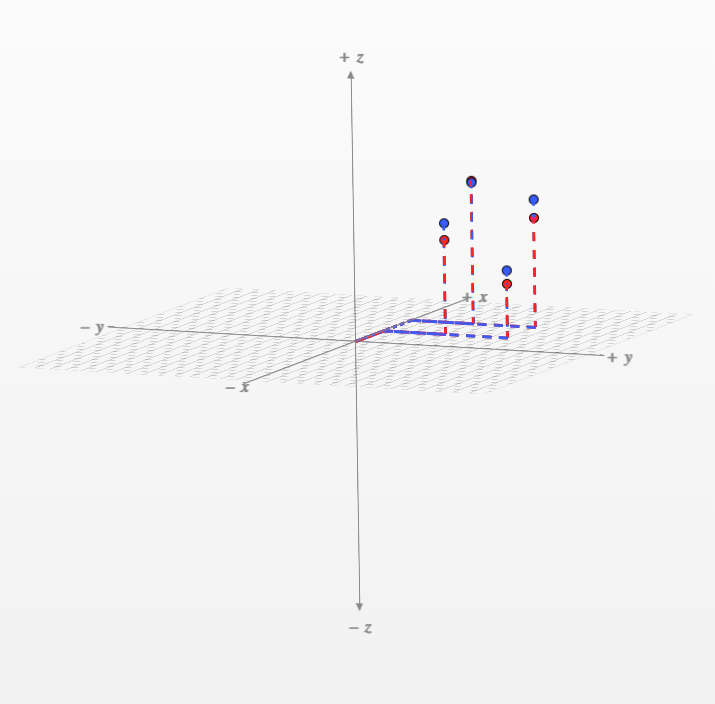


The horizontal axis (y) is density, 4 or 8 larvae per test tube. The axis receding into the plane of the page (x) is total food, 16 mg or 32 mg per test tube. The vertical axis (z) is the dependent variable, estimated Prime female growth rate (mg/day). The axes are not to the same scale; the food axis has been compressed relative to density and the dependent variable axis has been expanded to enhance the differences among the mean values. The red circles represent the 2 aliquot treatment and the blue circles represent the 4 aliquot treatment. The dotted lines serve to align the blue and red circles for the same treatments. From left to right, the four competitive environments are: low food, low density (intermediate competition); high food, low density (least competition); low food, high density (most competition); and high food, high density (intermediate competition).

The estimated growth rates for the Prime females are higher with 4 aliquots (blue circles) than with 2 aliquots (red circles). The estimated growth rates for the two least competition treatments (second from left) are almost the same (only the blue circle shows). See text for further explanation.
