# Supplementary material for: Competition and growth among Aedes aegypti larvae: Effects of distributing food inputs over time
Source: PLoS One. 2020 Oct 2;15(10):e0234676. doi: 10.1371/journal.pone.0234676 (PMC7531853; doi:10.1371/journal.pone.0234676)
Supplement: S21 Fig — 3D visualization of Prime female age and Average female mass for FxD. (DOCX) [file pone.0234676.s024.docx]

S21 Fig. Experiment 1. 3D visualization of Prime female age and Average female mass for FxD.


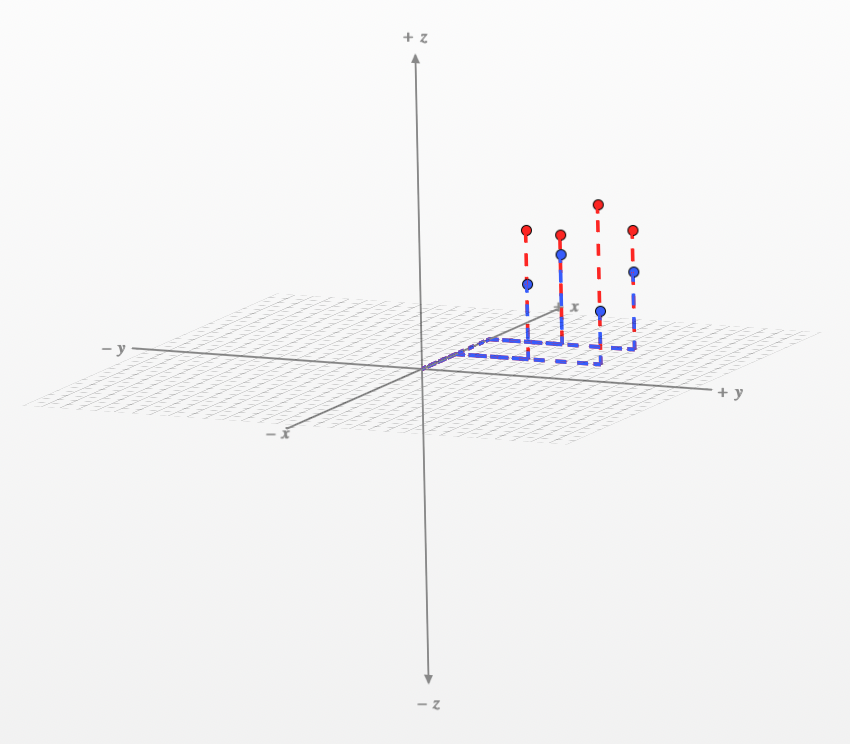


The horizontal axis (y) is density, 4 or 8 larvae per test tube. The axis receding into the plane of the page (x) is total food, 16 mg or 32 mg per test tube. The vertical axis (z) shows the dependent variables, Prime female age (days) and Average female mass (mg). The axes are not to the same scale; the food axis has been compressed relative to density and the dependent variable axis has been mapped in both days and mg to show the relative relationships between the means of the two dependent variables (1 day = 1.0 mg). The red circles represent the Prime female age and the blue circles represent Average female mass. The dotted lines serve to align the blue and red circles for the same treatments. From left to right, the four competitive environments are: low food, low density (intermediate competition); high food, low density (least competition); low food, high density (most competition); and high food, high density (intermediate competition).

Prime female age and Average female mass should be inversely related; an early age at pupation (red circles) and a large mass at pupation (blue circles) are both indications of good growing conditions for the larvae, while late pupation and small mass indicate poor conditions. The two circles for the least competition treatment (second from left) are the lowest age (red) and the highest mass (blue). The two circles for the most competition treatment (second from right) are the highest age (red) and the lowest mass (blue). The values for age (red) and mass (blue) at the intermediate levels of competition (extreme right and extreme left) are intermediate between the highest and lowest for both age (red) and mass (blue). See the text for further explanation.
