# Supplementary material for: Competition and growth among Aedes aegypti larvae: Effects of distributing food inputs over time
Source: PLoS One. 2020 Oct 2;15(10):e0234676. doi: 10.1371/journal.pone.0234676 (PMC7531853; doi:10.1371/journal.pone.0234676)
Supplement: S22 Fig — 3D visualization of Prime male age and Average male mass for FxD. (DOCX) [file pone.0234676.s025.docx]

S22 Fig. Experiment 1. 3D visualization of Prime male age and Average male mass for FxD.


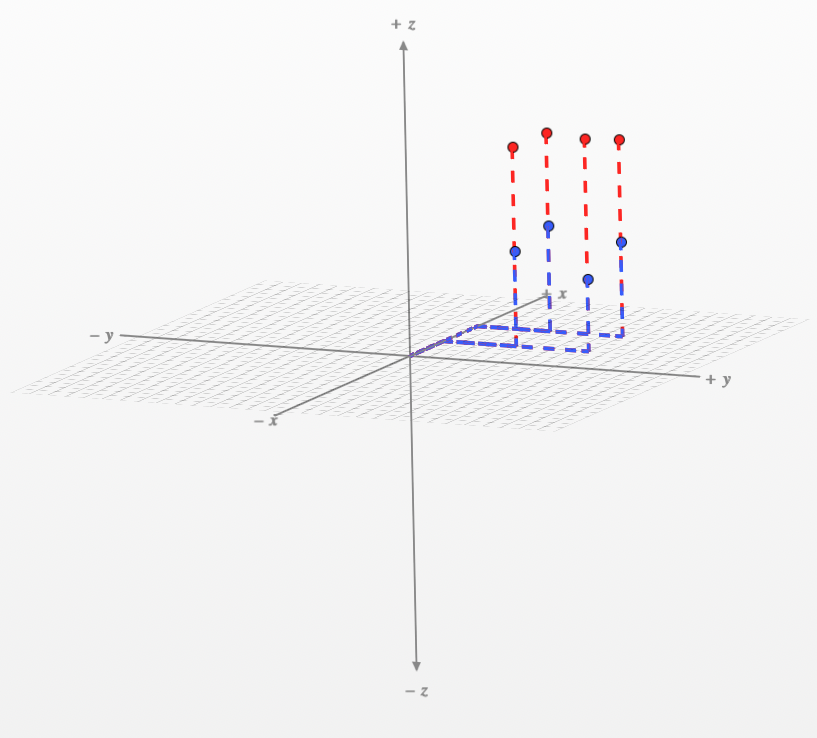


The horizontal axis (y) is density, 4 or 8 larvae per test tube. The axis receding into the plane of the page (x) is total food, 16 mg or 32 mg per test tube. The vertical axis (z) shows the dependent variables, Prime male age (days) and Average male mass (mg). The axes are not to the same scale; the food axis has been compressed relative to density and the dependent variable axis has been mapped in both days and mg to show the relative relationships between the means of the two dependent variables (1 day = 1.0 mg). The red circles represent the Prime male age and the blue circles represent Average male mass. The dotted lines serve to align the blue and red circles for the same treatments. From left to right, the four competitive environments are: low food, low density (intermediate competition); high food, low density (least competition); low food, high density (most competition); and high food, high density (intermediate competition).

Prime male age and Average male mass should be inversely related; an early age at pupation (red circles) and a large mass at pupation (blue circles) are both indications of good growing conditions for the larvae, while late pupation and small mass indicate poor conditions. The Prime male age (red) is similar for all the treatments except for the most competition (second from right) where age at pupation is latest for this interaction. This corresponds to the smallest Average male mass (blue, also second from right). The highest value for Average male mass (blue) is in the least competition treatment (second from left). The intermediate competition treatments have values for the Average male mass closer to the least competition than the most competition treatment. See the text for further explanation.
