# Supplementary material for: Competition and growth among Aedes aegypti larvae: Effects of distributing food inputs over time
Source: PLoS One. 2020 Oct 2;15(10):e0234676. doi: 10.1371/journal.pone.0234676 (PMC7531853; doi:10.1371/journal.pone.0234676)
Supplement: S24 Fig — 3D visualization of Average female mass for FxAxT. (DOCX) [file pone.0234676.s027.docx]

S24 Fig. Experiment 1. 3D visualization of Average female mass for FxAxT.


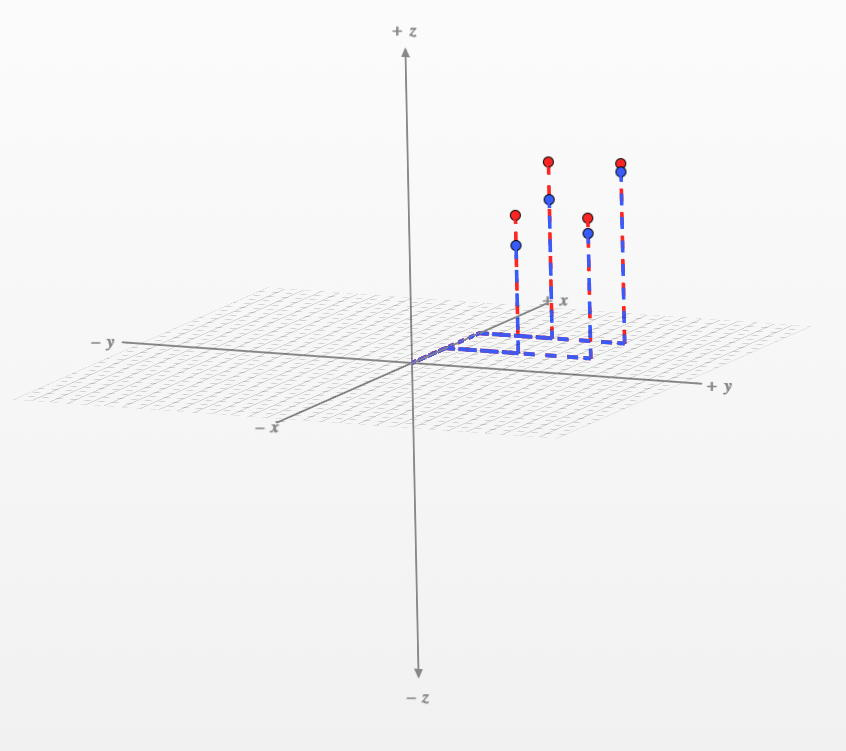


The horizontal axis (y) is aliquot, 2 or 4 aliquots of food spread over the timespan. The axis receding into the plane of the page (x) is total food, 16 mg or 32 mg per test tube. The vertical axis (z) is the dependent variable, Average female mass (mg). The axes are not to the same scale; the food axis has been compressed relative to density and the dependent variable axis has been expanded to enhance the differences among the mean values. The red circles represent the 3 day timespan and the blue circles represent the 6 day timespan. The dotted lines serve to align the blue and red circles for the same treatments. From left to right, the treatments are: low food, 2 aliquots; high food, 2 aliquots; low food, 4 aliquots; and high food, 4 aliquots.

All the circles in the front row (low food) appear lower than all the circles in the back row (high food). If fact, the lower blue circle in the back row (high food) is numerically between the two red circles in the front row (low food). The red circles (3 day timespan) are about the same within each food level regardless of the number of aliquots. The blue circles (6 day timespan) are affected by aliquot (4 aliquots are better than 2 aliquots), but also by the food level. 4 aliquots are better at high food than at low food (the extreme right pair of circles compared to the second from the right). 2 aliquots are worse at high food than at low food (the second from the left pair of circles compared to the extreme left). See text for further explanation.
