# Supplementary material for: Competition and growth among Aedes aegypti larvae: Effects of distributing food inputs over time
Source: PLoS One. 2020 Oct 2;15(10):e0234676. doi: 10.1371/journal.pone.0234676 (PMC7531853; doi:10.1371/journal.pone.0234676)
Supplement: S25 Fig — 3D visualization of Prime male mass and age for FxA. (DOCX) [file pone.0234676.s028.docx]

S25 Fig. Experiment 1. 3D visualization of Prime male mass and age for FxA.


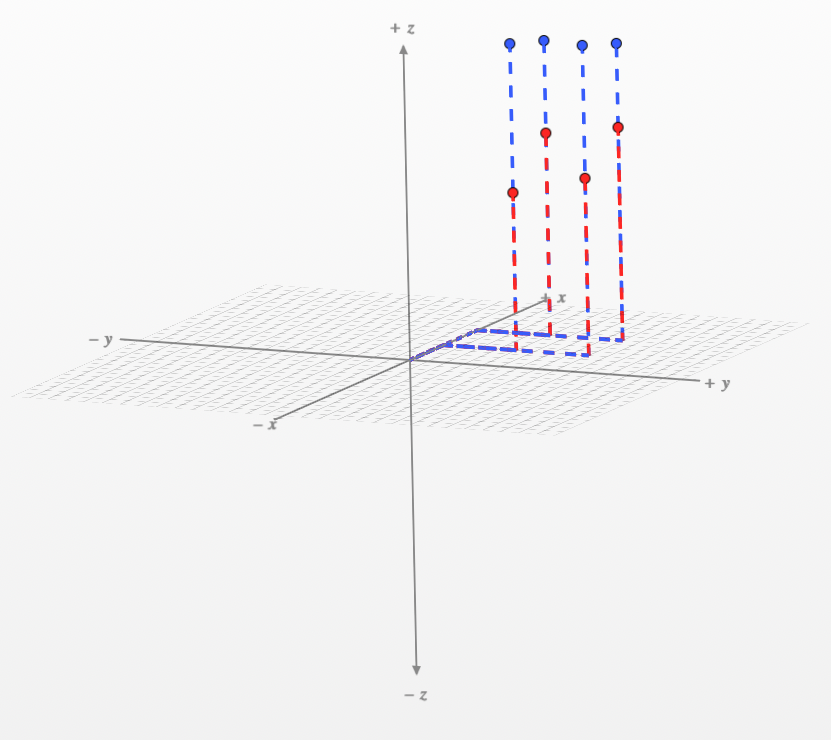


The horizontal axis (y) is aliquot, 2 or 4 aliquots of food spread over the timespan. The axis receding into the plane of the page (x) is total food, 16 mg or 32 mg per test tube. The vertical axis (z) shows the two dependent variables, Prime male mass (mg) and Prime male age (days). The axes are not to the same scale; the food axis has been compressed relative to density and the dependent variable axis has been expanded to enhance the differences among the mean values. The red circles represent the Prime male mass and the blue circles represent the Prime male age at pupation. The dotted lines serve to align the blue and red circles for the same treatments. From left to right, the treatments are: low food, 2 aliquots; high food, 2 aliquots; low food, 4 aliquots; and high food, 4 aliquots.

The Prime male mass is smallest at the low food, 2 aliquot treatment (red circle, extreme left). The Prime male mass is somewhat higher in the low food, 4 aliquot treatment (red circle, second from right). The Prime male mass is higher at the high food level (red circles, the back row) and again higher at 4 aliquots (red circle, extreme right) than at 2 aliquots (red circle, second from left). The Average male mass closely resembles the Prime male mass, and is not plotted. The difference in Prime male age at pupation is harder to see, although it is more significant (P<.001) than either of the two male mass variables (for both, P<.01). The earliest pupation is at high food and 2 aliquots (blue circle, second from left). The next earliest is at high food and 4 aliquots (blue circle, extreme right). The two latest to pupate are in the front row (low food), and the latest is also in the treatment with 4 aliquots (blue circle, second from right). The blue circles in the back row (high food) appear to be the same as the blue circles in the front row (low food) because there is a visual distortion to achieve the 3D effect and it reduces the apparent difference between the rows. See the text for further explanation.
