# Supplementary material for: Competition and growth among Aedes aegypti larvae: Effects of distributing food inputs over time
Source: PLoS One. 2020 Oct 2;15(10):e0234676. doi: 10.1371/journal.pone.0234676 (PMC7531853; doi:10.1371/journal.pone.0234676)
Supplement: S26 Fig — 3D visualization of Prime female mass and age for FxT. (DOCX) [file pone.0234676.s029.docx]

S26 Fig. Experiment 1. 3D visualization of Prime female mass and age for FxT.


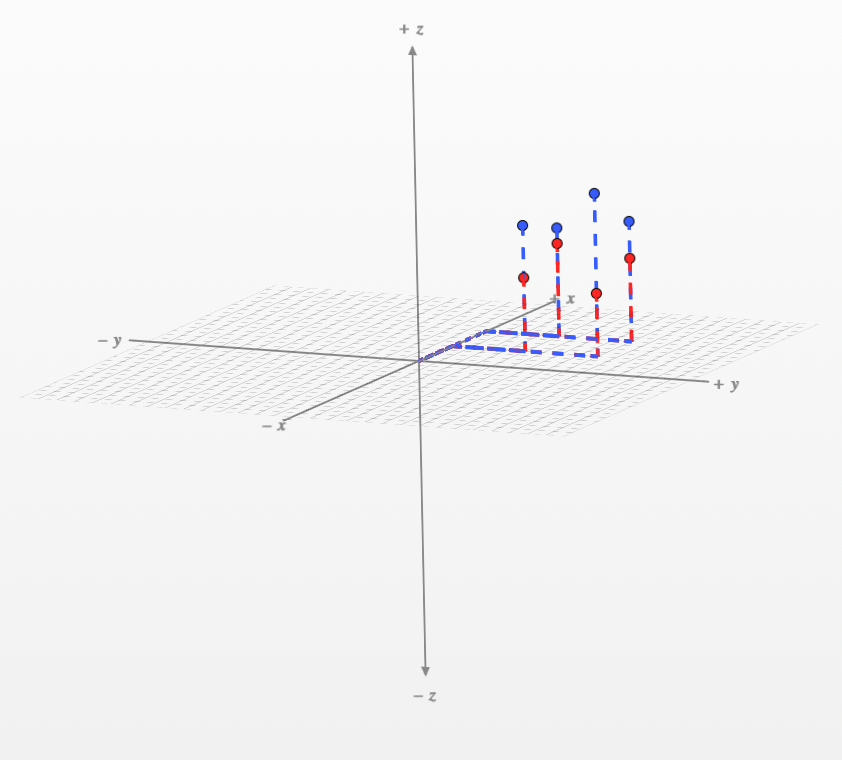


The horizontal axis (y) is timespan, 3 days or 6 days. The axis receding into the plane of the page (x) is total food, 16 mg or 32 mg per test tube. The vertical axis (z) shows the dependent variables, Prime female mass (mg) and Prime female age (days). The axes are not to the same scale; the food axis has been compressed relative to timespan and the dependent variable axis has been expanded to enhance the differences among the mean values. The red circles represent the Prime female age and the blue circles represent the Prime female mass. The dotted lines serve to align the blue and red circles for the same treatments. From left to right, the treatments are: low food, 3 day timespan; high food, 3 day timespan; low food, 6 day timespan; and high food, 6 day timespan.

The test tubes producing Prime females with the largest masses and earliest pupation are in the high food, 3 day timespan treatment (blue and red circles, respectively, second pair of circles from the left). The test tubes producing Prime females with the smallest masses and latest pupation are in the low food, 6 day timespan treatment (blue and red circles, respectively, second pair from the right). See text for further explanation.
