# Supplementary material for: Competition and growth among Aedes aegypti larvae: Effects of distributing food inputs over time
Source: PLoS One. 2020 Oct 2;15(10):e0234676. doi: 10.1371/journal.pone.0234676 (PMC7531853; doi:10.1371/journal.pone.0234676)
Supplement: S28 Fig — 3D visualization of mass versus total food after day 4 for FxT. (DOCX) [file pone.0234676.s031.docx]

S28 Fig. Experiment 1. 3D visualization of mass versus total food after day 4 for FxT.


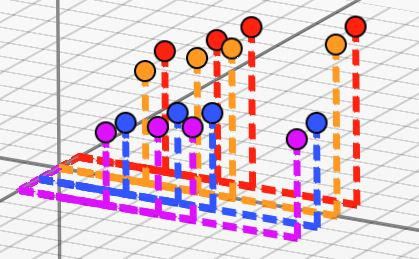


The horizontal axis (left to right) is the average total food after day 4 (2.5 mg, 4 mg, 5 mg, 8 mg) for the four treatments in FxT. The vertical axis is mass (mg) (see S31 Table-S32 Table). The four colors represent the Prime female mass (red), the Average female mass (orange), the Prime male mass (blue), and the Average male mass (purple). Females (both mass variables) increase in size almost linearly with increasing food/larva (left to right). Males (both mass variables) also increase in size with increasing food/larva (left to right), but not at the same rate and not as linearly as the females. The total food in the test tubes varies due to the aliquot treatment that is not part of this interaction; the average of those values is used for the graphical representation. No implication that this average is biologically or statistically important is suggested. See text for further explanation.
