# Supplementary material for: Competition and growth among Aedes aegypti larvae: Effects of distributing food inputs over time
Source: PLoS One. 2020 Oct 2;15(10):e0234676. doi: 10.1371/journal.pone.0234676 (PMC7531853; doi:10.1371/journal.pone.0234676)
Supplement: S29 Fig — 3D visualization of Prime female mass and age for AxT. (DOCX) [file pone.0234676.s032.docx]

S29 Fig. Experiment 1. 3D visualization of Prime female mass and age for AxT.


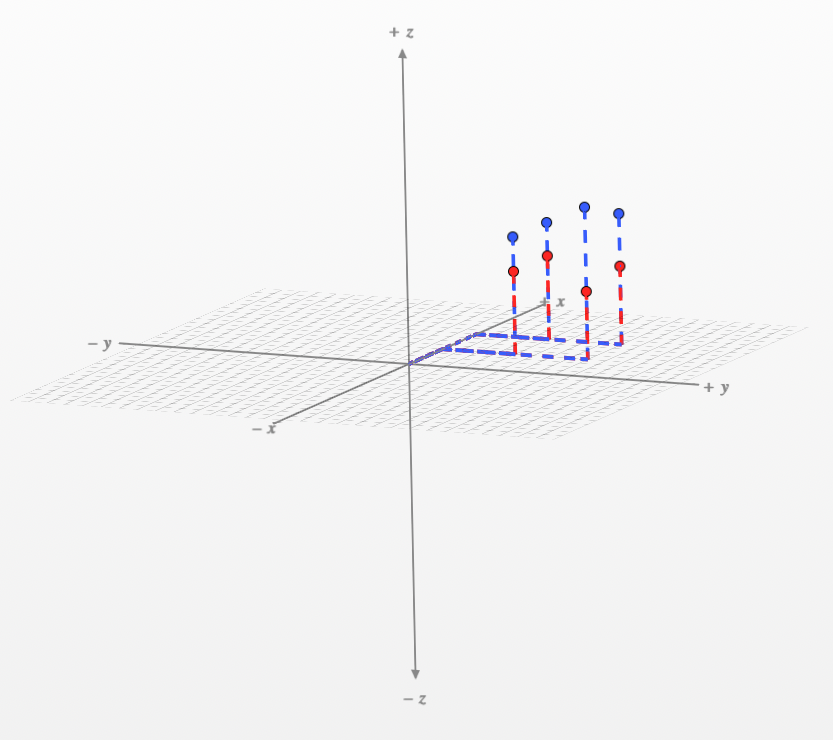


The horizontal axis (y) is timespan, 3 days or 6 days. The axis receding into the plane of the page (x) is aliquot, 2 or 4. The vertical axis (z) shows the dependent variables, Prime female mass (mg) and Prime female age (days). The axes are not to the same scale; aliquot and timespan are not in similar units, and the dependent variable axis has been expanded to enhance the differences among the mean values. The red circles represent the Prime female mass (mg) and the blue circles represent the Prime female age (days). Prime female age is not significantly affected by this interaction, but it is affected by the main effects of aliquot and timespan. The dotted lines serve to align the blue and red circles for the same treatments. From left to right, the treatments are: 2 aliquots, 3 day timespan; 4 aliquots, 3 day timespan; 2 aliquots, 6 day timespan; and 4 aliquots, 6 day timespan.

The largest Prime female mass is in the test tubes with 4 aliquots and the 3 day timespan (red circle, second from left). This is also the earliest pupation (blue circle, second from left). The Prime female mass in the test tubes with 2 aliquots and the 3 day timespan (red circle, extreme left) are almost as large and pupate almost as early (blue circle, extreme left). The smallest Prime female mass is in the test tubes with 2 aliquots and the 6 day timespan (red circle, second from right); this treatment is the latest to pupate (blue circle, second from right). The Prime female mass in the test tubes with 4 aliquots and the 6 day timespan is intermediate between the two largest and the smallest (red circle, extreme right). These females also pupate between the others (blue circle, extreme right). See text for further explanation.
