# Supplementary material for: Competition and growth among Aedes aegypti larvae: Effects of distributing food inputs over time
Source: PLoS One. 2020 Oct 2;15(10):e0234676. doi: 10.1371/journal.pone.0234676 (PMC7531853; doi:10.1371/journal.pone.0234676)
Supplement: S30 Fig — 3D visualization of Prime male mass and age for AxT. (DOCX) [file pone.0234676.s033.docx]

S30 Fig. Experiment 1. 3D visualization of Prime male mass and age for AxT.


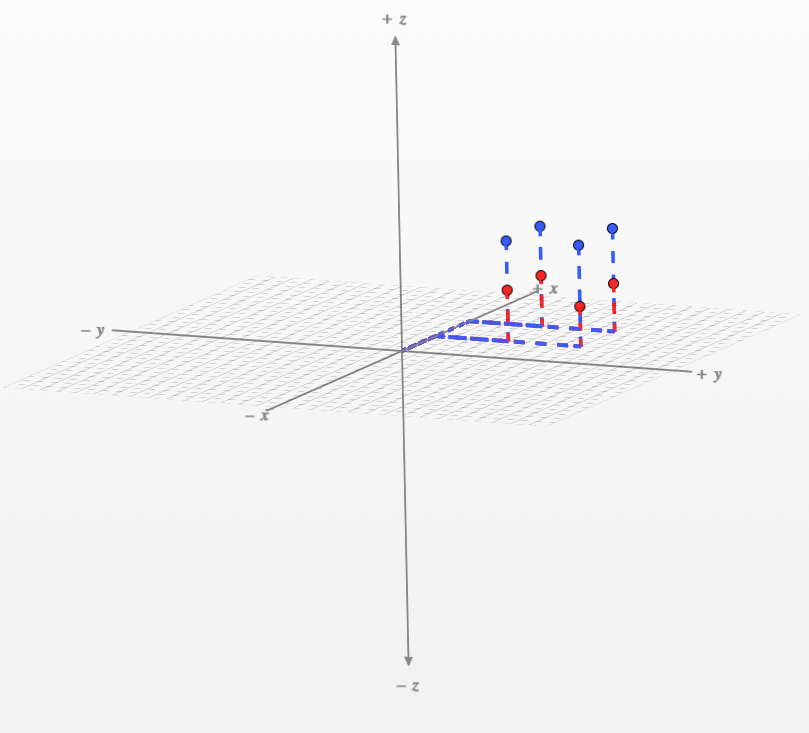


The horizontal axis (y) is timespan, 3 days or 6 days. The axis receding into the plane of the page (x) is aliquot, 2 or 4. The vertical axis (z) shows the dependent variables, Prime male mass (mg) and Prime male age (days). The axes are not to the same scale; aliquot and timespan are not in similar units, and the dependent variable axis has been expanded to enhance the differences among the mean values. The red circles represent the Prime male mass (mg) and the blue circles represent the Prime male age (days). The dotted lines serve to align the blue and red circles for the same treatments. From left to right, the treatments are: 2 aliquots, 3 day timespan; 4 aliquots, 3 day timespan; 2 aliquots, 6 day timespan; and 4 aliquots, 6 day timespan.

The largest Prime male masses are in the test tubes with 4 aliquots and the 3 day timespan (red circle, second from left), and in those with 2 aliquots and the 3 day timespan (red circle, extreme left). These are also the earliest to pupate (blue circles, second from left and extreme left). The smallest Prime male mass is in the test tubes with 2 aliquots and the 6 day timespan (red circle, second from right); this treatment is not the latest to pupate (blue circle, second from right). The Prime male mass in the test tubes with 4 aliquots and the 6 day timespan is intermediate between the two largest and the smallest masses (red circle, extreme right). These males pupate latest (blue circle, extreme right). See text for further explanation.
