# Supplementary material for: Competition and growth among Aedes aegypti larvae: Effects of distributing food inputs over time
Source: PLoS One. 2020 Oct 2;15(10):e0234676. doi: 10.1371/journal.pone.0234676 (PMC7531853; doi:10.1371/journal.pone.0234676)
Supplement: S31 Fig — 3D visualization of Prime female mass for DxAxT. (DOCX) [file pone.0234676.s034.docx]

S31 Fig. Experiment 1. 3D visualization of Prime female mass for DxAxT.


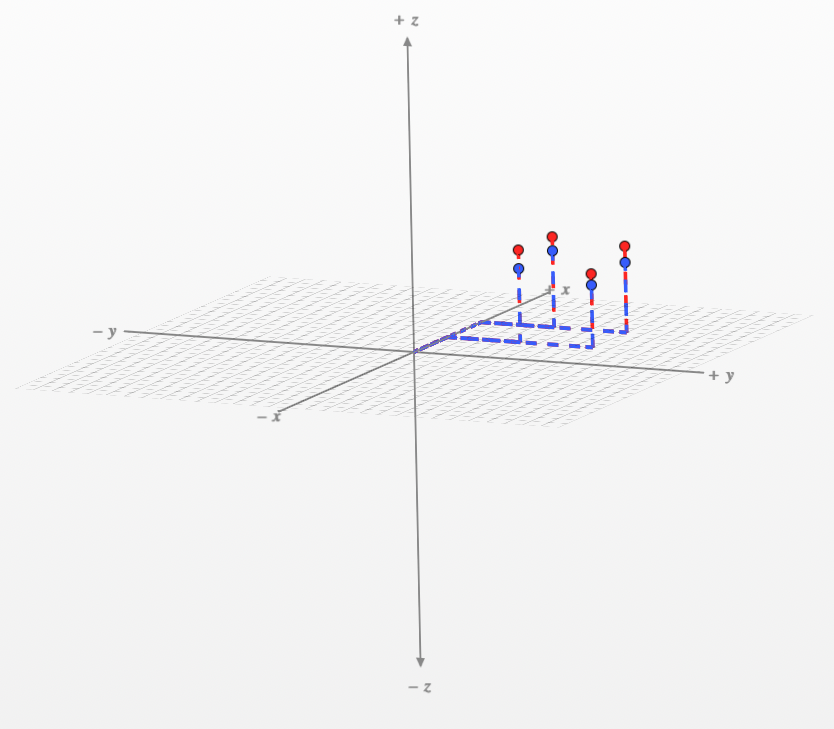


The horizontal axis (y) is timespan, 3 days or 6 days. The axis receding into the plane of the page (x) is aliquot, 2 or 4. The vertical axis (z) is the dependent variable, Prime female mass (mg). The axes are not to the same scale; aliquot and timespan are not in similar units, and the dependent variable axis has been expanded to enhance the differences among the mean values. The red circles represent the low density (4 larvae per test tube) and the blue circles represent the high density (8 larvae per test tube). The dotted lines serve to align the blue and red circles for the same treatments. From left to right, the treatments are: 2 aliquots, 3 day timespan; 4 aliquots, 3 day timespan; 2 aliquots, 6 day timespan; and 4 aliquots, 6 day timespan.

For each combination of aliquot (x) and timespan (y), the Prime female mass at low density (red circles) is greater than at high density (blue circles). At each density, the two highest Prime female masses are in the treatments with the 3 day timespan (red and blue circles, extreme left and second from left). The smallest Prime female masses are in the treatment with 2 aliquots and the 6 day timespan (pair of red and blue circles, second from right). The Prime females in the treatment with 4 aliquots and the 6 day timespan (pair of red and blue circles, extreme right) are intermediate between the largest and the smallest. See the text for additional explanation.
