# Supplementary material for: Competition and growth among Aedes aegypti larvae: Effects of distributing food inputs over time
Source: PLoS One. 2020 Oct 2;15(10):e0234676. doi: 10.1371/journal.pone.0234676 (PMC7531853; doi:10.1371/journal.pone.0234676)
Supplement: S32 Fig — 3D visualization of Average male mass for DxAxT. (DOCX) [file pone.0234676.s035.docx]

S32 Fig. Experiment 1. 3D visualization of Average male mass for DxAxT.


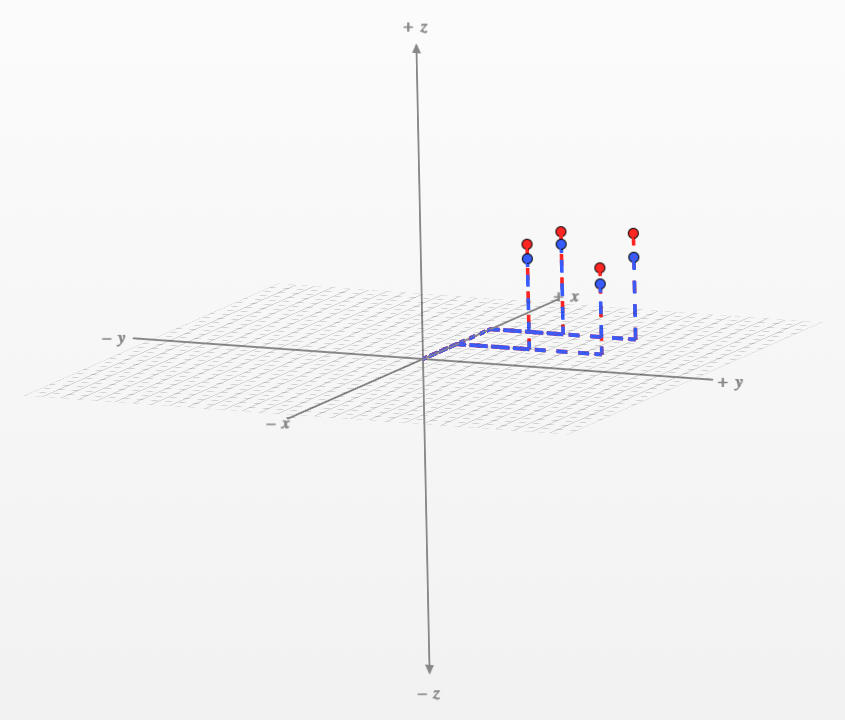


The horizontal axis (y) is timespan, 3 days or 6 days. The axis receding into the plane of the page (x) is aliquot, 2 or 4. The vertical axis (z) is the dependent variable Average male mass (mg). The axes are not to the same scale; aliquot and timespan are not in similar units, and the dependent variable axis has been expanded to enhance the differences among the mean values. The red circles represent the low density (4 larvae per test tube) and the blue circles represent the high density (8 larvae per test tube). The dotted lines serve to align the blue and red circles for the same treatments. From left to right, the treatments are: 2 aliquots, 3 day timespan; 4 aliquots, 3 day timespan; 2 aliquots, 6 day timespan; and 4 aliquots, 6 day timespan.

For each combination of aliquot (x) and timespan (y), the Average male mass at low density (red circles) is greater than at high density (blue circles). At the high density, the two highest Average male masses are in the treatments with the 3 day timespan (blue circles, extreme left and second from left). At the low density, the Average males in the treatment with 4 aliquots and the 6 day timespan (red circle, extreme right) are the largest. At both densities, the smallest Average male masses are in the treatment with 2 aliquots and the 6 day timespan (pair of red and blue circles, second from right). See the text for additional explanation.
