# Supplementary material for: Competition and growth among Aedes aegypti larvae: Effects of distributing food inputs over time
Source: PLoS One. 2020 Oct 2;15(10):e0234676. doi: 10.1371/journal.pone.0234676 (PMC7531853; doi:10.1371/journal.pone.0234676)
Supplement: S33 Fig — 3D visualization of Prime female age at pupation for DxA. (DOCX) [file pone.0234676.s036.docx]

S33 Fig. Experiment 1. 3D visualization of Prime female age at pupation for DxA.


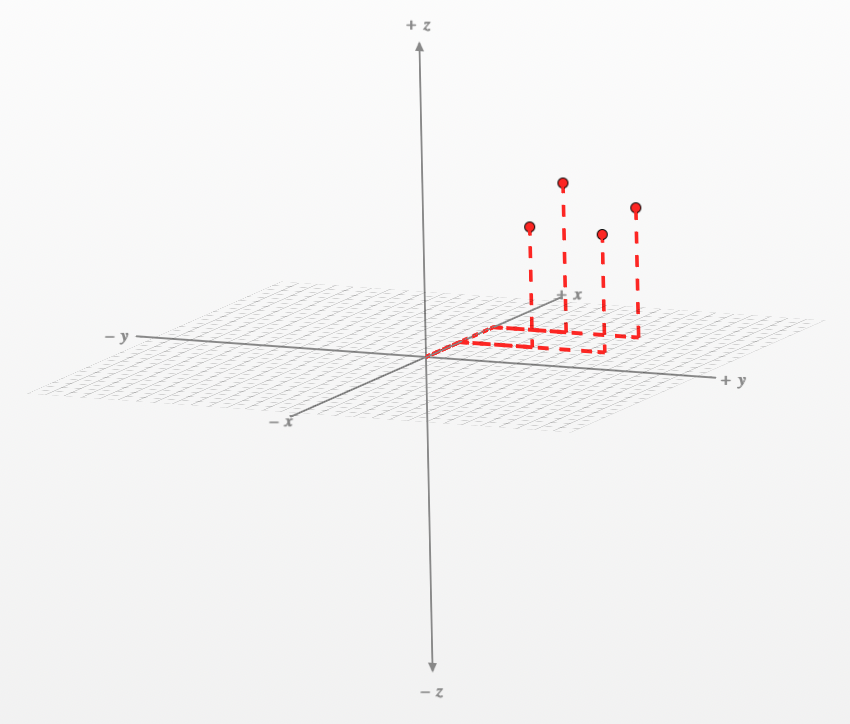


The horizontal axis (y) is aliquot, 2 or 4. The axis receding into the plane of the page (x) is density, 4 or 8 larvae per test tube. The vertical axis (z) is the dependent variable, Prime female age at pupation (days) The axes are not to the same scale; aliquot and density are not in similar units, and the dependent variable axis has been expanded to enhance the differences among the mean values. The red circles represent the Prime female age at pupation in days. The dotted lines serve to align the circles with the grid. From left to right, the four combinations are: low density, 2 aliquots, high density, 2 aliquots, low density, 4 aliquots, high density, 4 aliquots.

The high density treatments (back row) result in later pupation than the low density treatments (front row). There is a greater difference between the 2 aliquot treatments due to density than between the 4 aliquot treatments. See the text for further explanation.
