# Supplementary material for: Competition and growth among Aedes aegypti larvae: Effects of distributing food inputs over time
Source: PLoS One. 2020 Oct 2;15(10):e0234676. doi: 10.1371/journal.pone.0234676 (PMC7531853; doi:10.1371/journal.pone.0234676)
Supplement: S34 Fig — 3D visualization of Prime female mass and age for DxT. (DOCX) [file pone.0234676.s037.docx]

S34 Fig. Experiment 1. 3D visualization of Prime female mass and age for DxT.


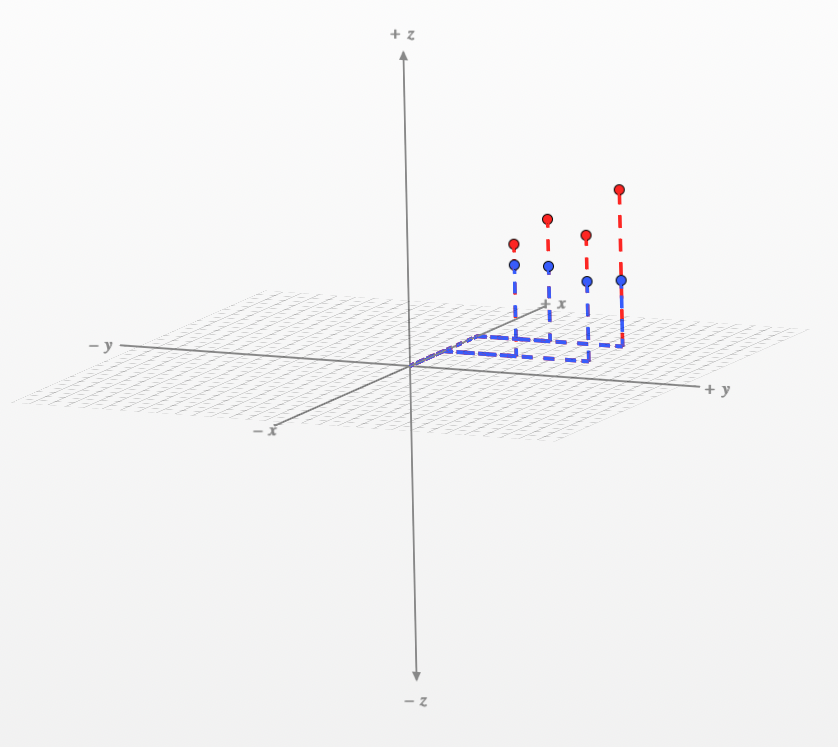


The horizontal axis (y) is timespan, 3 days or 6 days. The axis receding into the plane of the page (x) is density, 4 larvae or 8 larvae per test tube. The vertical axis (z) shows the dependent variables, Prime female mass (mg) and Prime female age (days). The axes are not to the same scale; density and timespan are not in the same units, and the dependent variable axis has been expanded to enhance the differences among the mean values. The red circles represent the Prime female age and the blue circles represent the Prime female mass. The dotted lines serve to align the blue and red circles for the same treatments. From left to right, the treatments are: low density, 3 day timespan; high density, 3 day timespan; low density, 6 day timespan; and high density, 6 day timespan.

The Prime female mass is largest and the age at pupation earliest in the low density, 3 day timespan treatment (pair of circles, extreme left). The Prime female mass is smallest and the age at pupation latest in the high density, 6 day timespan treatment (pair of circles, extreme right). The other two treatments are intermediate. There is an almost linear decrease in the mass of females (blue circles) from left to right as the amount of food in the test tubes decreases (after day 4). See the text for further explanation.
