# Supplementary material for: Competition and growth among Aedes aegypti larvae: Effects of distributing food inputs over time
Source: PLoS One. 2020 Oct 2;15(10):e0234676. doi: 10.1371/journal.pone.0234676 (PMC7531853; doi:10.1371/journal.pone.0234676)
Supplement: S35 Fig — 3D visualization of Prime male mass and age for DxT. (DOCX) [file pone.0234676.s038.docx]

S35 Fig. Experiment 1. 3D visualization of Prime male mass and age for DxT.


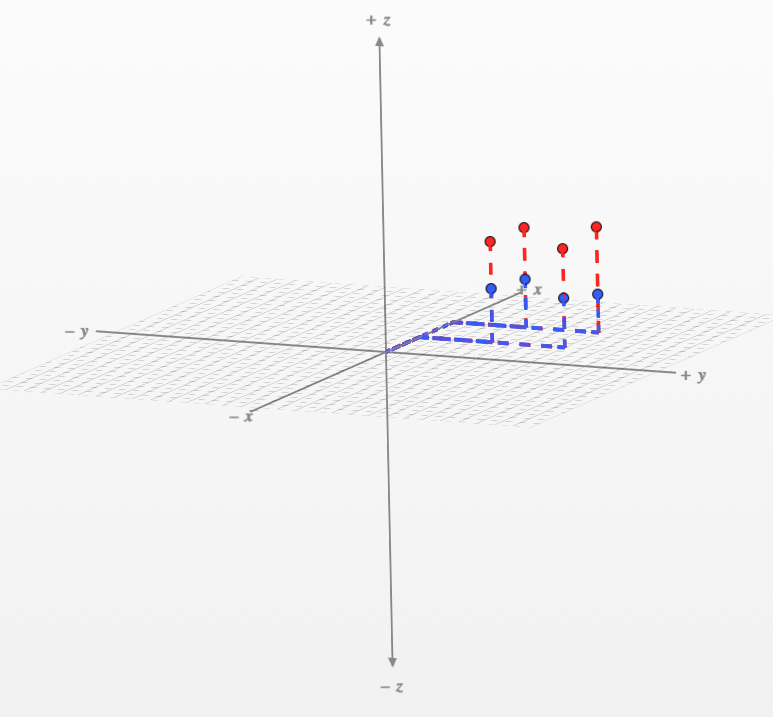


The horizontal axis (y) is timespan, 3 days or 6 days. The axis receding into the plane of the page (x) is density, 4 larvae or 8 larvae per test tube. The vertical axis (z) shows the dependent variables, Prime male mass (mg) and Prime male age (days). The axes are not to the same scale; density and timespan are not in the same units, and the dependent variable axis has been expanded to enhance the differences among the mean values. The red circles represent the Prime male age and the blue circles represent the Prime male mass. The dotted lines serve to align the blue and red circles for the same treatments. From left to right, the treatments are: low density, 3 day timespan; high density, 3 day timespan; low density, 6 day timespan; and high density, 6 day timespan.

The Prime male mass is largest in the low density, 3 day timespan treatment (blue circle, extreme left). The Prime male mass is smallest in the high density, 6 day timespan treatment (blue circle, extreme right). The other two treatments are intermediate in mass. The Prime male age at pupation is earliest in the low density, 6 day timespan treatment (red circle, second from right). The Prime male age at pupation latest in the high density, 6 day timespan treatment (red circle, extreme right). The two 3 day timespan treatments are intermediate with respect to age at pupation. See the text for further explanation.
