# Supplementary material for: Competition and growth among Aedes aegypti larvae: Effects of distributing food inputs over time
Source: PLoS One. 2020 Oct 2;15(10):e0234676. doi: 10.1371/journal.pone.0234676 (PMC7531853; doi:10.1371/journal.pone.0234676)
Supplement: S36 Fig — 3D visualization of mass versus average food/larva after day 4 for DxT. (DOCX) [file pone.0234676.s039.docx]

S36 Fig. Experiment 1. 3D visualization of mass versus average food/larva after day 4 for DxT.


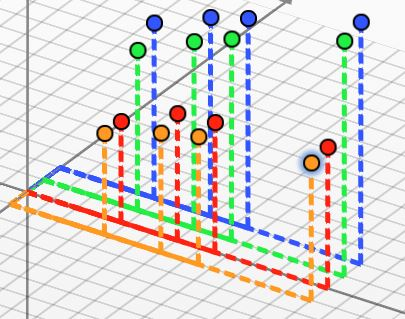


The horizontal axis (left to right) is the average food/larva after day 4 (1.875 mg, 3 mg, 3.75 mg, 6 mg) for the four treatments in DxT. The vertical axis is mass (mg) (see S41 Table-S42 Table). The four colors represent the Prime female mass (blue), the Average female mass (green), the Prime male mass (red), the Average male mass (orange). Females (both mass variables) increase in size almost linearly with increasing food/larva (left to right). Males (both mass variables) also increase in size with increasing food/larva (left to right), but not at the same rate and not as linearly as the females. The food/larva in the test tubes varies due to the food and aliquot treatments that are not part of this interaction; the average of those values is used for the graphical representation. No implication that this average is biologically or statistically important is suggested. See text for further explanation.
