# Supplementary material for: Competition and growth among Aedes aegypti larvae: Effects of distributing food inputs over time
Source: PLoS One. 2020 Oct 2;15(10):e0234676. doi: 10.1371/journal.pone.0234676 (PMC7531853; doi:10.1371/journal.pone.0234676)
Supplement: S37 Fig — Heuristic 3D graph of male and female masses and ages at pupation for the 3-way interaction between second food input, delay and sex in the third experiment. (DOCX) [file pone.0234676.s040.docx]

S37 Fig. Experiment 3. Heuristic 3D graph of male and female masses and ages at pupation for the 3 way interaction between second food input, delay and sex in the third experiment.


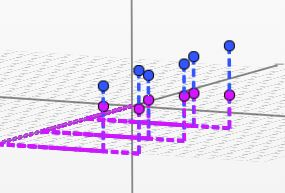


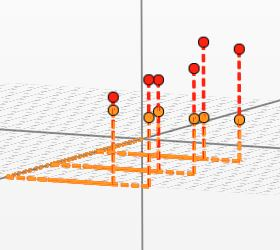


The x-axis (colored line) represents the amount of food in the second food input (1 mg, 2 mg or 3 mg dry weight of yeast). The y-axis (horizontal line) represents the 6 or 8 day delay between the initial food input (1 mg on day 0) and the second food input. The z-axis represents the mass and age of the pupae. Male mass (mg) is represented by purple dots and male age (days) is represented by blue dots (top schematic). Female mass (mg) is represented by orange dots and female age (days) is represented by red dots (bottom schematic). The dotted lines connect the dots with their x and y coordinates for clarity. Males and females are presented at the same scale. Females are larger and take longer to pupate at all combinations of second food input and delay. Females increase in mass and decrease in age at pupation as the second food input increases, but there are differences due to the delay treatment, especially for age at pupation. Males pupate earlier on the 6 day delay than the 8 day delay rather than according to the amount of food in the second input, and 3 of the 6 treatments resulted in males of the same size (2.27 mg) indicating that males reach a maximum size and pupate rather than growing as large as possible as the females do.
