# Supplementary material for: Competition and growth among Aedes aegypti larvae: Effects of distributing food inputs over time
Source: PLoS One. 2020 Oct 2;15(10):e0234676. doi: 10.1371/journal.pone.0234676 (PMC7531853; doi:10.1371/journal.pone.0234676)
Supplement: S38 Fig — Graph of the wet weight (mg) of male and female pupae against the dry weight of the yeast food source. (DOCX) [file pone.0234676.s041.docx]

S38 Fig. Experiment 3. Graph of the wet weight (mg) of male and female pupae against the dry weight of the yeast food source.


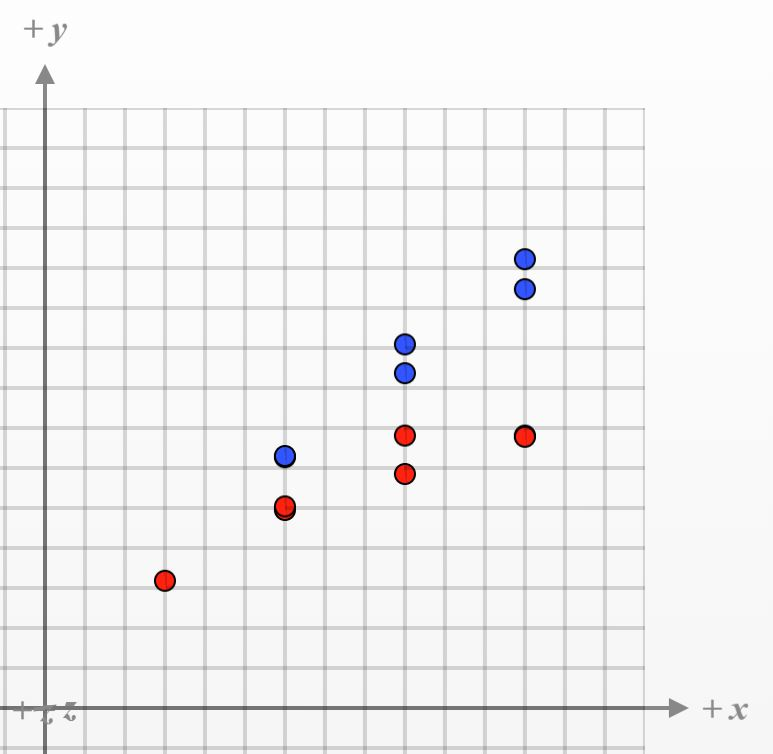


The x-axis (horizontal) represents the total dry weight of yeast added to each test tube in the third experiment: 1 mg, 2 mg, 3 mg, and 4 mg. The y-axis (vertical) represents the wet weight of the male (red dots) and female (blue dots) pupae in each test tube. There are 6 points representing female larvae and 7 points representing male larvae. There is only one point for the x = 1 mg value; this represents male larvae that pupated before receiving the second food input. These males pupated on 1 mg dry weight of yeast and attained a wet mass of 1.06 mg (SD=0.22). These males are excluded from the MANOVA analysis, but provide an additional reference point to compare the wet mass of males and females against the total dry weight of yeast in the rest of the experiment. There are two points for each sex for the x = 2 mg value; the two points for males and for females are overlapping. The two points for males for the x = 3 mg value are also overlapping. Both males and females increase in size as the total dry weight of yeast increases. Females are larger than males at all food levels and the difference between the female wet mass and the male wet mass increases as the total dry weight of yeast increases. The slope of the 6 female wet masses plotted against the dry weight of yeast is 0.76. The slope of the 7 male wet masses plotted against the dry weight of yeast is half that, 0.38. Females appear to grow larger than males on equal dry weights of yeast.
