# Supplementary material for: Competition and growth among Aedes aegypti larvae: Effects of distributing food inputs over time
Source: PLoS One. 2020 Oct 2;15(10):e0234676. doi: 10.1371/journal.pone.0234676 (PMC7531853; doi:10.1371/journal.pone.0234676)
Supplement: S1 Table — Food input schedule for each treatment according to the factors: food level (16 mg, 32 mg), aliquot (2 portions, 4 portions), and timespan (over days 0 to 3 or days 0 to 6). Density affects the food/larva which is also affected by the food input schedule. (DOCX) [file pone.0234676.s042.docx]

S1 Table. Experiment 1. Food input schedule for each treatment according to the factors: food level (16 mg, 32 mg), aliquot (2 portions, 4 portions), and timespan (over days 0 to 3 or days 0 to 6). Density affects the food/larva which is also affected by the food input schedule.

| Treatment combination | Food level (mg/vial) | Density (larvae/vial) | Aliquots (# inputs) | Timespan (days between first and last input) | Actual food input (mg) on day [0 1 2 3 4 5 6] |
| --- | --- | --- | --- | --- | --- |
| 1 | 16 | 4 | 2 | 3 | 8 0 0 8 - - - |
| 2 | 16 | 4 | 2 | 6 | 8 0 0 0 0 0 8 |
| 3 | 16 | 4 | 4 | 3 | 4 4 4 4 - - - |
| 4 | 16 | 4 | 4 | 6 | 4 0 4 0 4 0 4 |
| 5 | 16 | 8 | 2 | 3 | 8 0 0 8 - - - |
| 6 | 16 | 8 | 2 | 6 | 8 0 0 0 0 0 8 |
| 7 | 16 | 8 | 4 | 3 | 4 4 4 4 - - - |
| 8 | 16 | 8 | 4 | 6 | 4 0 4 0 4 0 4 |
| 9 | 32 | 4 | 2 | 3 | 16 0 0 16 - - - |
| 10 | 32 | 4 | 2 | 6 | 16 0 0 0 0 0 16 |
| 11 | 32 | 4 | 4 | 3 | 8 8 8 8 - - - |
| 12 | 32 | 4 | 4 | 6 | 8 0 8 0 8 0 8 |
| 13 | 32 | 8 | 2 | 3 | 16 0 0 16 - - - |
| 14 | 32 | 8 | 2 | 6 | 16 0 0 0 0 0 16 |
| 15 | 32 | 8 | 4 | 3 | 8 8 8 8 - - - |
| 16 | 32 | 8 | 4 | 6 | 8 0 8 0 8 0 8 |
