# Supplementary material for: Competition and growth among Aedes aegypti larvae: Effects of distributing food inputs over time
Source: PLoS One. 2020 Oct 2;15(10):e0234676. doi: 10.1371/journal.pone.0234676 (PMC7531853; doi:10.1371/journal.pone.0234676)
Supplement: S2 Table — Treatment numbers with density (larvae/vial), food/larva (2 mg, 3 mg, 4 mg, and 5 mg dry weight of yeast), and number of replicates. (DOCX) [file pone.0234676.s043.docx]

S2 Table. Experiment 2. Treatment numbers with density (larvae/vial), food/larva (2 mg, 3 mg, 4 mg, and 5 mg dry weight of yeast), and number of replicates.

| Treatment number | Density (larvae/vial) | Food/larva (mg) | Number of replicates |
| --- | --- | --- | --- |
| 1 | 1 | 2 | 5 |
| 2 | 1 | 3 | 5 |
| 3 | 1 | 4 | 5 |
| 4 | 1 | 5 | 5 |
| 5 | 2 | 2 | 5 |
| 6 | 2 | 3 | 5 |
| 7 | 2 | 4 | 5 |
| 8 | 2 | 5 | 5 |
| 9 | 3 | 2 | 5 |
| 10 | 3 | 3 | 5 |
| 11 | 3 | 4 | 5 |
| 12 | 3 | 5 | 5 |
