# Supplementary material for: Competition and growth among Aedes aegypti larvae: Effects of distributing food inputs over time
Source: PLoS One. 2020 Oct 2;15(10):e0234676. doi: 10.1371/journal.pone.0234676 (PMC7531853; doi:10.1371/journal.pone.0234676)
Supplement: S3 Table — Treatments showing the amount of the second food input (1 mg, 2 mg, 3 mg dry weight of yeast) and the delay (day of second food input, day 6 or day 8) with the number of replicates. All treatments received 1 mg dry weight of yeast on day 0 of the experiment. (DOCX) [file pone.0234676.s044.docx]

S3 Table. Experiment 3. Treatments showing the amount of the second food input (1 mg, 2 mg, 3 mg dry weight of yeast) and the delay (day of second food input, day 6 or day 8) with the number of replicates. All treatments received 1 mg dry weight of yeast on day 0 of the experiment.

| Treatment number | Incremental food quantity (mg) | Day of second food input | Number of replicates |
| --- | --- | --- | --- |
| 1 | 1 mg | day 6 | 25 |
| 2 | 2 mg | day 6 | 25 |
| 3 | 3 mg | day 6 | 25 |
| 4 | 1 mg | day 8 | 25 |
| 5 | 2 mg | day 8 | 25 |
| 6 | 3 mg | day 8 | 25 |
