# Supplementary material for: Competition and growth among Aedes aegypti larvae: Effects of distributing food inputs over time
Source: PLoS One. 2020 Oct 2;15(10):e0234676. doi: 10.1371/journal.pone.0234676 (PMC7531853; doi:10.1371/journal.pone.0234676)
Supplement: S4 Table — Significant correlations between composite scores and the 7 dependent variables with MANOVA significance levels and R squared by significant contrasts. (DOCX) [file pone.0234676.s045.docx]

S4 Table. Experiment 1. Significant correlations between composite scores and the 7 dependent variables with MANOVA significance levels and R squared by significant contrasts.

| Contrast | Survival | Prime male mass at pupation | Prime male age at pupation | Average male mass at pupation | Prime female mass at pupation | Prime female age at pupation | Average female mass at pupation | MANOVA P< | R squared |
| --- | --- | --- | --- | --- | --- | --- | --- | --- | --- |
| Food | 0.069 | -0.033 | -0.072 | 0.481 | 0.391 | -0.486 | 0.480 | 0.001 | 0.84 |
| Density | 0.070 | 0.550 | 0.879 | -0.316 | -0.037 | 0.566 | -0.094 | 0.001 | 0.89 |
| Aliquots | 0.327 | 0.141 | 0.798 | 0.219 | -0.076 | 0.503 | 0.217 | 0.001 | 0.70 |
| Timespan | 0.120 | 0.190 | 0.484 | 0.409 | 0.121 | -0.121 | 0.579 | 0.001 | 0.88 |
| F x D | 0.126 | -0.097 | 0.857 | 0.322 | -0.450 | 0.503 | 0.542 | 0.001 | 0.73 |
| F x A | 0.337 | 0.597 | 0.777 | 0.002 | 0.194 | -0.214 | -0.096 | 0.001 | 0.25 |
| F x T | 0.320 | 0.345 | 0.596 | 0.371 | 0.191 | -0.025 | 0.233 | 0.001 | 0.61 |
| D x A |  |  |  |  |  |  |  | ns | 0.07 |
| D x T | 0.165 | -0.163 | 0.786 | 0.491 | -0.122 | 0.341 | 0.558 | 0.001 | 0.84 |
| A x T | 0.211 | -0.094 | 0.462 | 0.721 | 0.014 | -0.191 | 0.621 | 0.001 | 0.51 |
| F x D x A | -0.085 | 1.579 | -0.053 | -1.670 | 0.515 | -0.127 | -1.175 | 0.001 | 0.22 |
| F x D x T | 0.199 | -0.355 | 0.680 | 0.790 | -0.098 | 0.191 | 0.646 | 0.001 | 0.87 |
| F x A x T |  |  |  |  |  |  |  | ns | 0.11 |
| D x A x T | 0.065 | 1.261 | 0.079 | -1.661 | -0.662 | -0.135 | 0.028 | 0.05 | 0.15 |
| F x D x A x T |  |  |  |  |  |  |  | ns | 0.10 |
