# Supplementary material for: Competition and growth among Aedes aegypti larvae: Effects of distributing food inputs over time
Source: PLoS One. 2020 Oct 2;15(10):e0234676. doi: 10.1371/journal.pone.0234676 (PMC7531853; doi:10.1371/journal.pone.0234676)
Supplement: S5 Table — Explanation of the meaning of the single df contrasts. (DOCX) [file pone.0234676.s046.docx]

S5 Table. Experiment 1. Explanation of the meaning of the single df contrasts.

| Contrast | Explanation of the abbreviation and the comparison of treatments |
| --- | --- |
| Food | There are two food levels, 16 mg per test tube and 32 mg per test tube. This contrast compares the value of the dependent variables on each of the food levels. |
| Density | There are two densities, 4 larvae per test tube and 8 larvae per test tube. This contrast compares the value of the dependent variables on each of the densities. |
| Aliquots | There are two aliquot treatments; the total food per test tube is divided equally into 2 aliquots or 4 aliquots and added according to the appropriate timespan treatment. The two aliquot treatments affect the rate of delivery of the total food: 50% of the food, followed by the second 50%; or 25% of the food delivered four times. The initial delivery of food is always on day 0 at the start of the experiment. This contrast compares the value of the dependent variables on each of the aliquot treatments. |
| Timespan | There are two timespan treatments; the total food per test tube is delivered over 3 days or 6 days (in the number of aliquots according to the appropriate aliquot treatment). The two timespan treatments affect the rate of delivery of the total food; either over 3 days or 6 days. The initial delivery of food is always on day 0 at the start of the experiment. This contrast compares the value of the dependent variables on each of the timespan treatments. |
| F x D | Food x density. This interaction examines the two food levels crossed with the two densities. The four combinations result in four different competitive intensities within the test tubes. The high food level (32 mg) with the low density (4 larvae) produces the least competition in the test tubes corresponding to 8 mg of food/larvae. The low food level (16 mg) with the high density (8 larvae) produces the most competition in the test tubes, corresponding to 2 mg of food/larva. The high food (32 mg) with the high density (8 larvae) produces an intermediate level of competition, corresponding to 4 mg of food/larva. The low food (16 mg) with the low density (4 larvae) also produces an intermediate level of competition, also corresponding to 4 mg of food/larva. |
| F x A | Food x aliquot. This interaction examines the two food levels crossed with the two aliquot treatments. The four combination are: low food (16 mg) crossed with 2 aliquots (each aliquot is 8 mg); low food (16 mg) crossed with 4 aliquots (each aliquot is 4 mg); high food (32 mg) crossed with 2 aliquots (each aliquot is 16 mg); high food (32 mg) crossed with 4 aliquots (each aliquot is 8 mg). These are both characteristics of the food supply. There is no density in this interaction, so no competition. Any deviation from the main effects of food and aliquot indicates the joint effect of these factors on the growth of the larvae. |
| F x T | Food x timespan. This interaction examines the two food levels crossed with the two timespan treatments. The four combination are: low food (16 mg) crossed with the 3 day timespan; low food (16 mg) crossed with the 6 day timespan; high food (32 mg) crossed with the 3 day timespan; high food (32 mg) crossed with the 6 day timespan. These are both characteristics of the food supply. There is no density in this interaction, so no competition. Any deviation from the main effects of food and timespan indicates the joint effect of these factors on the growth of the larvae. |
| D x A | Density x aliquot. This interaction examines the two densities crossed with the two aliquot treatments. There are four combinations: low density (4 larvae) with 2 aliquots; low density (4 larvae) with 4 aliquots; high density (8 larvae) with 2 aliquots; high density (8 larvae) with 4 aliquots. Aliquot is a characteristic of the food supply, so this could indicate competitive interactions among the larvae that are not related to the total food level or timespan. |
| D x T | Density x timespan. This interaction examines the two densities crossed with the two timespan treatments. There are four combinations: low density (4 larvae) with the 3 day timespan; low density (4 larvae) with 6 day timespan; high density (8 larvae) with 3 day timespan; high density (8 larvae) with 6 day timespan. Timespan is a characteristic of the food supply, so this could indicate competitive interactions among the larvae that are not related to the total food level or aliquot. |
| A x T | Aliquot x timespan. This interaction examines the two aliquot treatments crossed with the two timespan treatments. There are four combinations: 2 aliquots with the 3 day timespan (the first on day 0, the second on day 3); 2 aliquots with the 6 day timespan (the first on day 0, the second on day 6); 4 aliquots with the 3 day timespan (on day 0, day 1, day 2, day 3); 4 aliquots with the 6 day timespan (on day 0, day 2, day 4, day 6). These are both characteristics of the food supply. There is no density in this interaction, so no competition. Any deviation from the main effects of aliquot and timespan indicates the joint effect of these factors on the growth of the larvae. This would mean an effect of the distribution of the food supply that is independent of the total food per test tube. |
| F x D x A | Food x density x aliquot. This interaction examines the four competition treatments (see F x D above) crossed with the two aliquot treatments. This is an assay for the effect of the aliquot treatment on competition for each of the dependent variables. All three of the main factors have an effect on most of the dependent variables, and the levels of food and density were pre-selected to produce different levels of competition, so any interaction indicates that there is an effect of aliquot that differs from these effects. Aliquot is a characteristic of the food supply and affects the amount of food in the test tubes on different days during the experiment. |
| F x D x T | Food x density x timespan. This interaction examines the four competition treatments (see F x D above) crossed with the two timespan treatments. This is an assay for the effect of the timespan treatment on competition for each of the dependent variables. All three of the main factors have an effect on most of the dependent variables, and the levels of food and density were pre-selected to produce different levels of competition, so any interaction indicates that there is an effect of timespan that differs from these effects. Timespan is a characteristic of the food supply and affects the amount of food in the test tubes on different days during the experiment. |
| F x A x T | Food x aliquot x timespan. This interaction examines the two food levels crossed with the two aliquot treatments and the two timespan treatments (see also A x T above). This is a test for the effect of A x T (the joint effect of aliquot and timespan) on food level for each of the dependent variables. All three factors, food level, aliquot and timespan are characteristics of the food supply. There is no density in this interaction, so no competition. Any deviation from the main effects of food level, aliquot, and timespan indicates the joint effect of these factors on the growth of the larvae. |
| D x A x T | Density x aliquot x timespan. This interaction examines the two density treatments crossed with the two aliquot treatments and the two timespan treatments (see also A x T above). This is a test for the effect of A x T (the joint effect of aliquot and timespan) on density for each of the dependent variables. Both aliquot and timespan are characteristics of the food supply, so an interaction among these three variables could indicate competitive interactions that are not related to the total food level. |
| F x D x A x T | Food x density x aliquot x timespan. This interaction examines the four competition treatments (see F x D above) crossed with the two aliquot treatments and the two timespan treatments (see also A x T above). This is an assay for the effect of A x T (the joint effect of aliquot and timespan) on competition for each of the dependent variables. All four of the main factors have an effect on most of the dependent variables, and the levels of food and density were pre-selected to produce different levels of competition, so any interaction indicates that there is an effect of A x T that differs from these effects. Aliquot and timespan are both characteristics of the food supply and affect the amount of food in the test tubes on different days during the experiment. |
