# Supplementary material for: Competition and growth among Aedes aegypti larvae: Effects of distributing food inputs over time
Source: PLoS One. 2020 Oct 2;15(10):e0234676. doi: 10.1371/journal.pone.0234676 (PMC7531853; doi:10.1371/journal.pone.0234676)
Supplement: S6 Table — MANOVA contrasts for competition interactions (food, density, aliquot, timespan: FxDxT, FxDxA, FxD). R squared values, significance levels and discriminant function coefficients by dependent variable for the three interactions. (DOCX) [file pone.0234676.s047.docx]

S6 Table. MANOVA contrasts for competition interactions (food, density, aliquot, timespan: FxDxT, FxDxA, FxD). R squared values, significance levels and discriminant function coefficients by dependent variable for the three interactions.

| Contrast | Survival | Prime male mass at pupation | Prime male age at pupation | Average male mass at pupation | Prime female mass at pupation | Prime female age at pupation | Average female mass at pupation | MANOVA P< | R squared |
| --- | --- | --- | --- | --- | --- | --- | --- | --- | --- |
| F x D x T | 0.199 | -0.355 | 0.68 | 0.79 | -0.098 | 0.191 | 0.646 | 0.001 | 0.87 |
| F x D x A | -0.085 | 1.579 | -0.053 | -1.67 | 0.515 | -0.127 | -1.175 | 0.001 | 0.22 |
| F x D | 0.126 | -0.097 | 0.857 | 0.322 | -0.45 | 0.503 | 0.542 | 0.001 | 0.73 |
