# Supplementary material for: Competition and growth among Aedes aegypti larvae: Effects of distributing food inputs over time
Source: PLoS One. 2020 Oct 2;15(10):e0234676. doi: 10.1371/journal.pone.0234676 (PMC7531853; doi:10.1371/journal.pone.0234676)
Supplement: S7 Table — MANOVA contrasts for interactions involving only attributes of the food supply (food, aliquot, timespan: FxA, FxT, AxT). R squared values, significance levels and discriminant function coefficients by dependent variable for the three interactions. (DOCX) [file pone.0234676.s048.docx]

S7 Table. MANOVA contrasts for interactions involving only attributes of the food supply (food, aliquot, timespan: FxA, FxT, AxT). R squared values, significance levels and discriminant function coefficients by dependent variable for the three interactions.

| Contrast | Survival | Prime male mass at pupation | Prime male age at pupation | Average male mass at pupation | Prime female mass at pupation | Prime female age at pupation | Average female mass at pupation | MANOVA P< | R squared |
| --- | --- | --- | --- | --- | --- | --- | --- | --- | --- |
| F x A | 0.337 | 0.597 | 0.777 | 0.002 | 0.194 | -0.214 | -0.096 | 0.001 | 0.25 |
| F x T | 0.32 | 0.345 | 0.596 | 0.371 | 0.191 | -0.025 | 0.233 | 0.001 | 0.61 |
| A x T | 0.211 | -0.094 | 0.462 | 0.721 | 0.014 | -0.191 | 0.621 | 0.001 | 0.51 |
