# Supplementary material for: Competition and growth among Aedes aegypti larvae: Effects of distributing food inputs over time
Source: PLoS One. 2020 Oct 2;15(10):e0234676. doi: 10.1371/journal.pone.0234676 (PMC7531853; doi:10.1371/journal.pone.0234676)
Supplement: S8 Table — MANOVA contrasts for interactions between density and the non-food level attributes of the food supply (density, aliquot, timespan: DxAxT, DxT). R squared values, significance levels and discriminant function coefficients by dependent variable for the three interactions. (DOCX) [file pone.0234676.s049.docx]

S8 Table. MANOVA contrasts for interactions between density and the non-food level attributes of the food supply (density, aliquot, timespan: DxAxT, DxT). R squared values, significance levels and discriminant function coefficients by dependent variable for the three interactions.

| Contrast | Survival | Prime male mass at pupation | Prime male age at pupation | Average male mass at pupation | Prime female mass at pupation | Prime female age at pupation | Average female mass at pupation | MANOVA P< | R squared |
| --- | --- | --- | --- | --- | --- | --- | --- | --- | --- |
| D x A x T | 0.065 | 1.261 | 0.079 | -1.661 | -0.662 | -0.135 | 0.028 | 0.05 | 0.15 |
| D x T | 0.165 | -0.163 | 0.786 | 0.491 | -0.122 | 0.341 | 0.558 | 0.001 | 0.84 |
