# Supplementary material for: Competition and growth among Aedes aegypti larvae: Effects of distributing food inputs over time
Source: PLoS One. 2020 Oct 2;15(10):e0234676. doi: 10.1371/journal.pone.0234676 (PMC7531853; doi:10.1371/journal.pone.0234676)
Supplement: S9 Table — ANOVA significant r squared values for the main treatments and significant interaction contrasts from the MANOVA for the 7 dependent variables. The row labelled “Subtotal r squared” includes only the r squared values for the contrasts listed in this table; the row labelled “Total r squared” includes all of the r squared values in the respective ANOVAs. (DOCX) [file pone.0234676.s050.docx]

S9 Table. ANOVA significant r squared values for the main treatments and significant interaction contrasts from the MANOVA for the 7 dependent variables. The row labelled “Subtotal r squared” includes only the r squared values for the contrasts listed in this table; the row labelled “Total r squared” includes all of the r squared values in the respective ANOVAs.

| Contrast | Survival | Prime male mass at pupation | Prime male age at pupation | Average male mass at pupation | Prime female mass at pupation | Prime female age at pupation | Average female mass at pupation |
| --- | --- | --- | --- | --- | --- | --- | --- |
| Main treatments: F, D, A, T | 0.12 | 0.56 | 0.45 | 0.48 | 0.54 | 0.54 | 0.56 |
| F x D |  |  | 0.11 | 0.01 |  | 0.08 | 0.01 |
| F x A |  | 0.01 | 0.01 | 0.01 |  |  |  |
| F x T | 0.08 | 0.09 | 0.02 | 0.08 | 0.03 |  | 0.02 |
| D x T | 0.02 | 0.03 | 0.17 | 0.06 | 0.10 | 0.11 | 0.10 |
| A x T |  | 0.05 | 0.01 | 0.06 | 0.03 |  | 0.03 |
| F x D x A |  |  |  |  | 0.00 |  | 0.01 |
| F x D x T | 0.05 | 0.10 | 0.16 | 0.16 | 0.16 | 0.05 | 0.16 |
| D x A x T |  |  |  | 0.01 | 0.01 |  |  |
| Subtotal r squared | 0.27 | 0.84 | 0.93 | 0.87 | 0.87 | 0.78 | 0.89 |
| Total r squared | 0.27 | 0.84 | 0.93 | 0.87 | 0.88 | 0.79 | 0.90 |
