# Supplementary material for: Competition and growth among Aedes aegypti larvae: Effects of distributing food inputs over time
Source: PLoS One. 2020 Oct 2;15(10):e0234676. doi: 10.1371/journal.pone.0234676 (PMC7531853; doi:10.1371/journal.pone.0234676)
Supplement: S10 Table — ANOVA mean squares, (r squared), and significance [*] for each single df contrast across the 7 dependent variables. (DOCX) [file pone.0234676.s051.docx]

S10 Table. Experiment 1. ANOVA mean squares, (r squared), and significance [*} for each single df contrast across the 7 dependent variables.

| Contrast | Survival | Prime male mass at pupation | Prime male age at pupation | Average male mass at pupation | Prime female mass at pupation | Prime female age at pupation | Average female mass at pupation |
| --- | --- | --- | --- | --- | --- | --- | --- |
| Food | 0.29 | 10.94 (0.20) *** | 0.92 (0.00) ** | 7.18 (0.16) *** | 39.85 (0.26) *** | 76.33 (0.11) *** | 37.62 (0.26) *** |
| Density | 0.41 | 0.41 (0.01) * | 57.16 (0.31) *** | 0.22 (0.00) * | 0.31 | 228.21 (0.32) *** | 0.10 |
| Aliquots | 2.81 (0.12) *** | 0.75 (0.01) *** | 13.79 (0.07) *** | 0.88 (0.02) *** | 2.84 (0.02) *** | 68.58 (0.10) *** | 1.21 (0.01) ** |
| Timespan | 0.40 | 18.56 (0.34) *** | 12.50 (0.07) *** | 13.32 (0.30) *** | 40.14 (0.26) *** | 5.39 (0.01) * | 42.44 (0.29) *** |
| F x D | 0.01 | 0.14 | 20.10 (0.11) *** | 0.47 (0.01) ** | 0.42 | 57.94 (0.08) *** | 1.21 (0.01) ** |
| F x A | 0.40 | 0.67 (0.01) ** | 1.82 (0.01) *** | 0.46 (0.01) ** | 0.06 | 2.72 | 0.02 |
| F x T | 1.77 (0.08) *** | 4.79 (0.09) *** | 4.27 (0.02) *** | 3.65 (0.08) *** | 4.67 (0.03) *** | 0.02 | 3.40 (0.02) *** |
| D x A | 0.05 | 0.02 | 0.09 | 0 | 0.09 | 5.09 (0.01) * | 0.16 |
| D x T | 0.58 (0.02) * | 1.72 (0.03) *** | 31.62 (0.17) *** | 2.56 (0.06) *** | 15.21 (0.10) *** | 78.67 (0.11) *** | 13.87 (0.10) *** |
| A x T | 0.22 | 2.80 (0.05) *** | 1.85 (0.01) *** | 2.52 (0.06) *** | 4.00 (0.03) *** | 3.48 | 4.77 (0.03) *** |
| F x D x A | 0.02 | 0 | 0.04 | 0.16 | 0.71 (0.00) * | 1.20 | 1.32 (0.01) *** |
| F x D x T | 1.08 (0.05) ** | 5.19 (0.10) *** | 29.75 (0.16) *** | 7.41 (0.16) *** | 23.89 (0.16) *** | 37.97 (0.05) *** | 23.62 (0.16) *** |
| F x A x T | 0.01 | 0.01 | 0.04 | 0.08 | 0.79 (0.01) * | 2.40 | 0.84 (0.01) ** |
| D x A x T | 0 | 0.06 | 0 | 0.29 (0.01) * | 1.08 (0.01) * | 2.20 | 0.36 |
| F x D x A x T | 0.43 | 0.13 | 0 | 0.12 | 0.24 | 0.02 | 0.53 (0.00) * |
| Residual (df=115) | 0.13 | 0.07 | 0.10 | 0.05 | 0.17 | 1.16 | 0.12 |
| Total r squared | 0.27 | 0.84 | 0.93 | 0.87 | 0.88 | 0.79 | 0.90 |

*** =P<.001, **=P<.01, *=P<.05.
