# Supplementary material for: Competition and growth among Aedes aegypti larvae: Effects of distributing food inputs over time
Source: PLoS One. 2020 Oct 2;15(10):e0234676. doi: 10.1371/journal.pone.0234676 (PMC7531853; doi:10.1371/journal.pone.0234676)
Supplement: S11 Table — Means (SE) for the main effects: food, density, aliquot and timespan; for the 7 dependent variables. (DOCX) [file pone.0234676.s052.docx]

| Main effects | Survival (arcsine transformed percent survival) | Prime male mass at pupation (mg) | Prime male age at pupation (days) | Average male mass at pupation (mg) | Prime female mass at pupation (mg) | Prime female age at pupation (days) | Average female mass at pupation (mg) |
| --- | --- | --- | --- | --- | --- | --- | --- |
| **Food level** |  |  |  |  |  |  |  |
| High food | 1.23 (0.17) | 2.64 (0.23) | 5.02 (0.05) | 2.53 (0.25) | 4.46 (0.46) | 5.79 (0.56) | 4.23 (0.58) |
| Low food | 1.19 (0.18) | 2.13 (0.47) | 5.23 (0.31) | 2.11 (0.41) | 3.44 (0.70) | 7.33 (1.56) | 3.24 (0.72) |
| **Density** |  |  |  |  |  |  |  |
| Low density | 1.30 (0.13) | 2.59 (0.27) | 5.05 (0.07) | 2.53 (0.29) | 4.33 (0.55) | 6.04 (0.81) | 4.17 (0.61) |
| High density | 1.12 (0.16) | 2.17 (0.50) | 5.20 (0.33) | 2.10 (0.38) | 3.57 (0.80) | 7.08 (1.68) | 3.30 (0.79) |
| **Aliquot** |  |  |  |  |  |  |  |
| 2 aliquots | 1.27 (0.16) | 2.28 (0.51) | 5.10 (0.18) | 2.23 (0.43) | 3.81 (0.79) | 6.84 (1.80) | 3.56 (0.84) |
| 4 aliquots | 1.15 (0.17) | 2.48 (0.37) | 5.15 (0.31) | 2.41 (0.35) | 4.09 (0.79) | 6.28 (0.82) | 3.91 (0.80) |
| **Timespan** |  |  |  |  |  |  |  |
| 3 day timespan | 1.17 (0.15) | 2.56 (0.28) | 5.07 (0.08) | 2.45 (0.29) | 4.21 (0.76) | 5.93 (0.66) | 4.02 (0.78) |
| 6 day timespan | 1.25 (0.18) | 2.20 (0.52) | 5.17 (0.34) | 2.18 (0.45) | 3.69 (0.74) | 7.18 (1.66) | 3.44 (0.78) |

S11 Table. Experiment 1. Means (SE) for the main effects: food, density, aliquot and timespan; for the 7 dependent variables.
