# Supplementary material for: Competition and growth among Aedes aegypti larvae: Effects of distributing food inputs over time
Source: PLoS One. 2020 Oct 2;15(10):e0234676. doi: 10.1371/journal.pone.0234676 (PMC7531853; doi:10.1371/journal.pone.0234676)
Supplement: S12 Table — Number of replicates (N). (DOCX) [file pone.0234676.s053.docx]

S12 Table. Experiment 1. Number of replicates (N).

| Aliquot x Timespan=> | 2 aliquots, 3 days | 2 aliquots, 6 days | 4 aliquots, 3 days | 4 aliquots, 6 days |
| --- | --- | --- | --- | --- |
| Food x Density |  |  |  |  |
| Low food, low density (4 mg/larva) | 9 | 5 | 8 | 5 |
| Most competition (2 mg/larva) | 10 | 10 | 9 | 9 |
| Least competition (8 mg/larva) | 9 | 8 | 7 | 7 |
| High food, high density (4 mg/larva) | 10 | 9 | 8 | 8 |
