# Supplementary material for: Competition and growth among Aedes aegypti larvae: Effects of distributing food inputs over time
Source: PLoS One. 2020 Oct 2;15(10):e0234676. doi: 10.1371/journal.pone.0234676 (PMC7531853; doi:10.1371/journal.pone.0234676)
Supplement: S13 Table — Means (SD) of arcsin transformed percent Survival. (DOCX) [file pone.0234676.s054.docx]

S13 Table. Experiment 1. Means (SD) of arcsin transformed percent Survival.

| Aliquot x Timespan => | 2 aliquots, 3 days | 2 aliquots, 6 days | 4 aliquots, 3 days | 4 aliquots, 6 days | Mean of means [SE] |
| --- | --- | --- | --- | --- | --- |
| Food x Density |  |  |  |  |  |
| Low food, low density (4 mg/larva) | 1.41 (0.32) | 1.28 (0.40) | 1.21 (0.39) | 1.43 (0.32) | 1.33 [0.11] |
| Most competition (2 mg/larva) | 1.12 (0.35) | 1.03 (0.22) | 1.04 (0.33) | 0.96 (0.43) | 1.04 [0.07] |
| Least competition (8 mg/larva) | 1.13 (0.43) | 1.48 (0.26) | 1.16 (0.39) | 1.26 (0.39) | 1.26 [0.16] |
| High food, high density (4 mg/larva) | 1.33 (0.33) | 1.35 (0.33) | 0.93 (0.48) | 1.20 (0.32) | 1.20 [0.19] |
| Mean of means [SE] | 1.25 [0.15] | 1.29 [0.19] | 1.09 [0.13] | 1.21 [0.19] |  |
