# Supplementary material for: Competition and growth among Aedes aegypti larvae: Effects of distributing food inputs over time
Source: PLoS One. 2020 Oct 2;15(10):e0234676. doi: 10.1371/journal.pone.0234676 (PMC7531853; doi:10.1371/journal.pone.0234676)
Supplement: S14 Table — Means (SD) Prime female mass at pupation (mg). (DOCX) [file pone.0234676.s055.docx]

S14 Table. Experiment 1. Means (SD) Prime female mass at pupation (mg).

| Aliquot x Timespan => | 2 aliquots, 3 days | 2 aliquots, 6 days | 4 aliquots, 3 days | 4 aliquots, 6 days | Mean of means [SE] |
| --- | --- | --- | --- | --- | --- |
| Food x Density |  |  |  |  |  |
| Low food, low density (4 mg/larva) | 4.44 (0.28) | 3.27 (0.76) | 4.40 (0.21) | 3.88 (0.53) | 4.00 [0.55] |
| Most competition (2 mg/larva) | 2.96 (0.20) | 2.75 (0.20) | 3.04 (0.27) | 2.80 (0.23) | 2.89 [0.14] |
| Least competition (8 mg/larva) | 4.89 (0.45) | 4.16 (0.17) | 4.75 (0.80) | 4.86 (0.38) | 4.67 [0.34] |
| High food, high density (4 mg/larva) | 4.47 (0.40) | 3.54 (0.72) | 4.69 (0.27) | 4.28 (0.31) | 4.25 [0.50] |
| Mean of means [SE] | 4.19 [0.85] | 3.43 [0.59] | 4.22 [0.80] | 3.96 [0.87] |  |
