# Supplementary material for: Competition and growth among Aedes aegypti larvae: Effects of distributing food inputs over time
Source: PLoS One. 2020 Oct 2;15(10):e0234676. doi: 10.1371/journal.pone.0234676 (PMC7531853; doi:10.1371/journal.pone.0234676)
Supplement: S15 Table — Means (SD) Prime female age at pupation (days). (DOCX) [file pone.0234676.s056.docx]

S15 Table. Experiment 1. Means (SD) Prime female age at pupation (days).

| Aliquot x Timespan => | 2 aliquots, 3 days | 2 aliquots, 6 days | 4 aliquots, 3 days | 4 aliquots, 6 days | Mean of means [SE] |
| --- | --- | --- | --- | --- | --- |
| Food x Density |  |  |  |  |  |
| Low food, low density (4 mg/larva) | 5.89 (0.60) | 7.80 (1.10) | 5.88 (0.99) | 6.60 (0.89) | 6.54 [0.90] |
| Most competition (2 mg/larva) | 7.30 (1.83) | 10.60 (1.27) | 6.44 (0.53) | 8.11 (1.17) | 8.11 [1.79] |
| Least competition (8 mg/larva) | 5.22 (0.67) | 5.50 (0.76) | 5.71 (0.49) | 5.71 (0.49) | 5.54 [0.23] |
| High food, high density (4 mg/larva) | 5.40 (0.70) | 7.00 (2.06) | 5.63 (0.74) | 6.13 (0.84) | 6.04 [0.71] |
| Mean of means [SE] | 5.95 [0.94] | 7.73 [2.14] | 5.92 [0.37] | 6.64 [1.05] |  |
