# Supplementary material for: Competition and growth among Aedes aegypti larvae: Effects of distributing food inputs over time
Source: PLoS One. 2020 Oct 2;15(10):e0234676. doi: 10.1371/journal.pone.0234676 (PMC7531853; doi:10.1371/journal.pone.0234676)
Supplement: S16 Table — Means (SD) Average female mass at pupation (mg). (DOCX) [file pone.0234676.s057.docx]

S16 Table. Experiment 1. Means (SD) Average female mass at pupation (mg).

| Aliquot x Timespan => | 2 aliquots, 3 days | 2 aliquots, 6 days | 4 aliquots, 3 days | 4 aliquots, 6 days | Mean of means [SE] |
| --- | --- | --- | --- | --- | --- |
| Food x Density |  |  |  |  |  |
| Low food, low density (4 mg/larva) | 4.22 (0.26) | 2.97 (0.37) | 4.28 (0.23) | 3.69 (0.42) | 3.79 [0.61] |
| Most competition (2 mg/larva) | 2.77 (0.18) | 2.49 (0.25) | 2.83 (0.30) | 2.64 (0.16) | 2.68 [0.15] |
| Least competition (8 mg/larva) | 4.76 (0.41) | 4.03 (0.21) | 4.71 (0.79) | 4.66 (0.39) | 4.54 [0.34] |
| High food, high density (4 mg/larva) | 4.20 (0.22) | 3.00 (0.38) | 4.42 (0.27) | 4.06 (0.43) | 3.92 [0.63] |
| Mean of means [SE] | 3.99 [0.85] | 3.12 [0.65] | 4.06 [0.84] | 3.76 [0.85] |  |
