# Supplementary material for: Competition and growth among Aedes aegypti larvae: Effects of distributing food inputs over time
Source: PLoS One. 2020 Oct 2;15(10):e0234676. doi: 10.1371/journal.pone.0234676 (PMC7531853; doi:10.1371/journal.pone.0234676)
Supplement: S17 Table — Means (SD) Prime male mass at pupation (mg). (DOCX) [file pone.0234676.s058.docx]

S17 Table. Experiment 1. Means (SD) Prime male mass at pupation (mg).

| Aliquot x Timespan => | 2 aliquots, 3 days | 2 aliquots, 6 days | 4 aliquots, 3 days | 4 aliquots, 6 days | Mean of means [SE] |
| --- | --- | --- | --- | --- | --- |
| Food x Density |  |  |  |  |  |
| Low food, low density (4 mg/larva) | 2.66 (0.18) | 1.99 (0.19) | 2.61 (0.16) | 2.51 (0.13) | 2.44 [0.31] |
| Most competition (2 mg/larva) | 2.08 (0.17) | 1.28 (0.20) | 2.15 (0.20) | 1.73 (0.10) | 1.81 [0.40] |
| Least competition (8 mg/larva) | 2.76 (0.24) | 2.54 (0.20) | 2.77 (0.39) | 2.88 (0.69) | 2.74 [0.14] |
| High food, high density (4 mg/larva) | 2.79 (0.14) | 2.17 (0.13) | 2.67 (0.31) | 2.51 (0.22) | 2.54 [0.27] |
| Mean of means [SE] | 2.57 [0.33] | 2.00 [0.53] | 2.55 [0.27] | 2.41 [0.48] |  |
