# Supplementary material for: Competition and growth among Aedes aegypti larvae: Effects of distributing food inputs over time
Source: PLoS One. 2020 Oct 2;15(10):e0234676. doi: 10.1371/journal.pone.0234676 (PMC7531853; doi:10.1371/journal.pone.0234676)
Supplement: S18 Table — Means (SD) Prime male age at pupation (days). (DOCX) [file pone.0234676.s059.docx]

S18 Table. Experiment 1. Means (SD) Prime male age at pupation (days).

| Aliquot x Timespan => | 2 aliquots, 3 days | 2 aliquots, 6 days | 4 aliquots, 3 days | 4 aliquots, 6 days | Mean of means [SE] |
| --- | --- | --- | --- | --- | --- |
| Food x Density |  |  |  |  |  |
| Low food, low density (4 mg/larva) | 5.11 (0.33) | 5.00 (0.00) | 5.13 (0.35) | 5.00 (0.00) | 5.06 [0.07] |
| Most competition (2 mg/larva) | 5.20 (0.42) | 5.50 (0.71) | 5.00 (0.00) | 5.89 (0.60) | 5.40 [0.39] |
| Least competition (8 mg/larva) | 5.00 (0.00) | 5.00 (0.00) | 5.14 (0.38) | 5.00 (0.00) | 5.04 [0.07] |
| High food, high density (4 mg/larva) | 5.00 (0.00) | 5.00 (0.00) | 5.00 (0.00) | 5.00 (0.00) | 5.00 [0.00] |
| Mean of means [SE] | 5.08 [0.10] | 5.13 [0.25] | 5.07 [0.08] | 5.22 [0.45] |  |
