# Supplementary material for: Competition and growth among Aedes aegypti larvae: Effects of distributing food inputs over time
Source: PLoS One. 2020 Oct 2;15(10):e0234676. doi: 10.1371/journal.pone.0234676 (PMC7531853; doi:10.1371/journal.pone.0234676)
Supplement: S19 Table — Means (SD) Average male mass at pupation (mg). (DOCX) [file pone.0234676.s060.docx]

S19 Table. Experiment 1. Means (SD) Average male mass at pupation (mg).

| Aliquot x Timespan => | 2 aliquots, 3 days | 2 aliquots, 6 days | 4 aliquots, 3 days | 4 aliquots, 6 days | Mean of means [SE] |
| --- | --- | --- | --- | --- | --- |
| Food x Density |  |  |  |  |  |
| Low food, low density (4 mg/larva) | 2.61 (0.16) | 1.88 (0.20) | 2.61 (0.19) | 2.50 (0.10) | 2.40 [0.35] |
| Most competition (2 mg/larva) | 1.98 (0.16) | 1.52 (0.11) | 2.01 (0.15) | 1.77 (0.12) | 1.82 [0.23] |
| Least competition (8 mg/larva) | 2.70 (0.24) | 2.50 (0.17) | 2.59 (0.23) | 2.87 (0.69) | 2.67 [0.16] |
| High food, high density (4 mg/larva) | 2.59 (0.08) | 2.03 (0.12) | 2.54 (0.26) | 2.38 (0.11) | 2.39 [0.25] |
| Mean of means [SE] | 2.47 [0.33] | 1.98 [0.41] | 2.44 [0.29] | 2.38 [0.46] |  |
