# Supplementary material for: Competition and growth among Aedes aegypti larvae: Effects of distributing food inputs over time
Source: PLoS One. 2020 Oct 2;15(10):e0234676. doi: 10.1371/journal.pone.0234676 (PMC7531853; doi:10.1371/journal.pone.0234676)
Supplement: S20 Table — Means (SE) for FxDxT for arcsin transformed percent Survival. (DOCX) [file pone.0234676.s061.docx]

S20 Table. Means (SE) for arcsin transformed percent Survival for the interaction FxDxT

| Food x Density | Timespan | Survival |
| --- | --- | --- |
| Low food, low density (4 mg/larva) | 3 days | 1.31 (0.14) |
|  | 6 days | 1.36 (0.11) |
| Most competition (2 mg/larva) | 3 days | 1.08 (0.06) |
|  | 6 days | 1.00 (0.05) |
| Least competition (8 mg/larva) | 3 days | 1.15 (0.02) |
|  | 6 days | 1.37 (0.16) |
| High food, high density (4 mg/larva) | 3 days | 1.13 (0.28) |
|  | 6 days | 1.28 (0.11) |
