# Supplementary material for: Competition and growth among Aedes aegypti larvae: Effects of distributing food inputs over time
Source: PLoS One. 2020 Oct 2;15(10):e0234676. doi: 10.1371/journal.pone.0234676 (PMC7531853; doi:10.1371/journal.pone.0234676)
Supplement: S21 Table — Means (SE) for FxDxT for Prime female mass and age, and Average female mass. Estimated growth rate and difference between the Prime and Average female mass. (DOCX) [file pone.0234676.s062.docx]

S21 Table. Means (SE) for Prime female mass and age at pupation and Average female mass at pupation for the interaction FxDxT

| Food x Density | Timespan | Rank by Prime female mass (a-h) | Prime female mass at pupation (mg) | Prime female age at pupation (days) | Average female mass at pupation (mg) | Estimated Prime female growth rate (mg/day) | Prime female mass MINUS Average female mass (mg) |
| --- | --- | --- | --- | --- | --- | --- | --- |
| Low food, low density (4 mg/larva) | 3 days | d | 4.42 (0.03) | 5.89 (0.01) | 4.25 (0.04) | 0.75 (0.01) | 0.17 (0.05) |
|  | 6 days | f | 3.58 (0.43) | 7.20 (0.85) | 3.33 (0.51) | 0.50 (0.19) | 0.25 (0.67) |
| Most competition (2 mg/larva) | 3 days | g | 3.00 (0.06) | 6.87 (0.61) | 2.80 (0.04) | 0.44 (0.10) | 0.20 (0.07) |
|  | 6 days | h | 2.78 (0.04) | 9.36 (1.76) | 2.57 (0.11) | 0.30 (0.17) | 0.21 (0.12) |
| Least competition (8 mg/larva) | 3 days | a | 4.82 (0.10) | 5.47 (0.35) | 4.74 (0.04) | 0.88 (0.14) | 0.08 (0.11) |
|  | 6 days | c | 4.51 (0.49) | 5.61 (0.15) | 4.35 (0.45) | 0.80 (0.19) | 0.16 (0.67) |
| High food, high density (4 mg/larva) | 3 days | b | 4.58 (0.16) | 5.52 (0.16) | 4.31 (0.16) | 0.83 (0.08) | 0.27 (0.23) |
|  | 6 days | e | 3.91 (0.52) | 6.57 (0.62) | 3.53 (0.75) | 0.60 (0.21) | 0.38 (0.91) |
