# Supplementary material for: Competition and growth among Aedes aegypti larvae: Effects of distributing food inputs over time
Source: PLoS One. 2020 Oct 2;15(10):e0234676. doi: 10.1371/journal.pone.0234676 (PMC7531853; doi:10.1371/journal.pone.0234676)
Supplement: S22 Table — Means (SE) for Prime female mass and age at pupation and Average female mass at pupation for the interaction FxDxT. Total food after day 4 and food/larva after day 4. (DOCX) [file pone.0234676.s063.docx]

S22 Table. Means (SE) for Prime female mass and age at pupation and Average female mass at pupation for the interaction FxDxT with total food after day 4 and food/larva after day 4.

| Food x Density | Timespan | Rank by Prime female mass (a-h) | Prime female mass at pupation (mg) | Prime female age at pupation (days) | Average female mass at pupation (mg) | Total food after day 4 (mg) | Food/larva after day 4 (mg) |
| --- | --- | --- | --- | --- | --- | --- | --- |
| Low food, low density (4 mg/larva) | 3 days | d | 4.42 (0.03) | 5.89 (0.01) | 4.25 (0.04) | 16, 16 | 4, 4 |
|  | 6 days | f | 3.58 (0.43) | 7.20 (0.85) | 3.33 (0.51) | 8, 12 | 2, 3 |
| Most competition (2 mg/larva) | 3 days | g | 3.00 (0.06) | 6.87 (0.61) | 2.80 (0.04) | 16, 16 | 2, 2 |
|  | 6 days | h | 2.78 (0.04) | 9.36 (1.76) | 2.57 (0.11) | 8, 12 | 1, 1.5 |
| Least competition (8 mg/larva) | 3 days | a | 4.82 (0.10) | 5.47 (0.35) | 4.74 (0.04) | 32, 32 | 8, 8 |
|  | 6 days | c | 4.51 (0.49) | 5.61 (0.15) | 4.35 (0.45) | 16, 24 | 4, 6 |
| High food, high density (4 mg/larva) | 3 days | b | 4.58 (0.16) | 5.52 (0.16) | 4.31 (0.16) | 32, 32 | 4, 4 |
|  | 6 days | e | 3.91 (0.52) | 6.57 (0.62) | 3.53 (0.75) | 16, 24 | 4, 6 |
