# Supplementary material for: Competition and growth among Aedes aegypti larvae: Effects of distributing food inputs over time
Source: PLoS One. 2020 Oct 2;15(10):e0234676. doi: 10.1371/journal.pone.0234676 (PMC7531853; doi:10.1371/journal.pone.0234676)
Supplement: S23 Table — Means (SE) for FxDxT for Prime male mass and age, and Average male mass. (DOCX) [file pone.0234676.s064.docx]

S23 Table. Means (SE) for Prime male mass and age at pupation and Average male mass at pupation for the interaction FxDxT

| Food x Density | Timespan | Rank by Prime male mass (a-h) | Prime male mass at pupation (mg) | Prime male age at pupation (days) | Average male mass at pupation (mg) | Estimated Prime male growth rate (mg/day) | Prime male mass MINUS Average male mass (mg) |
| --- | --- | --- | --- | --- | --- | --- | --- |
| Low food, low density (4 mg/larva) | 3 days | d | 2.64 (0.04) | 5.12 (0.01) | 2.61 (0.00) | 0.52 (0.01) | 0.03 (0.04) |
|  | 6 days | f | 2.25 (0.37) | 5.00 (0.00) | 2.19 (0.44) | 0.45 (0.11) | 0.06 (0.57) |
| Most competition (2 mg/larva) | 3 days | g | 2.12 (0.05) | 5.10 (0.14) | 2.00 (0.02) | 0.42 (0.03) | 0.12 (0.05) |
|  | 6 days | h | 1.51 (0.32) | 5.70 (0.28) | 1.65 (0.18) | 0.26 (0.07) | -0.14 (0.37) |
| Least competition (8 mg/larva) | 3 days | a | 2.77 (0.01) | 5.07 (0.10) | 2.65 (0.08) | 0.55 (0.02) | 0.12 (0.08) |
|  | 6 days | c | 2.71 (0.24) | 5.00 (0.00) | 2.69 (0.26) | 0.54 (0.08) | 0.02 (0.35) |
| High food, high density (4 mg/larva) | 3 days | b | 2.73 (0.08) | 5.00 (0.00) | 2.57 (0.04) | 0.55 (0.03) | 0.16 (0.09) |
|  | 6 days | e | 2.34 (0.24) | 5.00 (0.00) | 2.21 (0.25) | 0.47 (0.07) | 0.13 (0.35) |
