# Supplementary material for: Competition and growth among Aedes aegypti larvae: Effects of distributing food inputs over time
Source: PLoS One. 2020 Oct 2;15(10):e0234676. doi: 10.1371/journal.pone.0234676 (PMC7531853; doi:10.1371/journal.pone.0234676)
Supplement: S24 Table — Means (SD) Average female mass for FxDxAxT. (DOCX) [file pone.0234676.s065.docx]

S24 Table. The means (standard errors) of the Average female mass for the separate 2 aliquot and 4 aliquot treatments presented alongside the 8 mean values for the FxDxT interaction.

| Food x Density | Timespan | FxDxT Means (SE) (mg) | FxDxAxT, 2 aliquots Means (SD) (mg) | FxDxAxT, 4 aliquots Means (SD) (mg) |
| --- | --- | --- | --- | --- |
| Low food, low density (4 mg/larva) | 3 days | 4.25 (0.04) | 4.22 (0.26) | 4.28 (0.23) |
|  | 6 days | 3.33 (0.51) | 2.97 (0.37) | 3.69 (0.42) |
| Most competition (2 mg/larva) | 3 days | 2.80 (0.04) | 2.77 (0.18) | 2.83 (0.30) |
|  | 6 days | 2.57 (0.11) | 2.49 (0.25) | 2.64 (0.16) |
| Least competition (8 mg/larva) | 3 days | 4.74 (0.04) | 4.76 (0.41) | 4.71 (0.79) |
|  | 6 days | 4.35 (0.45) | 4.03 (0.21) | 4.66 (0.39) |
| High food, high density (4 mg/larva) | 3 days | 4.31 (0.16) | 4.20 (0.22) | 4.42 (0.27) |
|  | 6 days | 3.53 (0.75) | 3.00 (0.38) | 4.06 (0.43) |
