# Supplementary material for: Competition and growth among Aedes aegypti larvae: Effects of distributing food inputs over time
Source: PLoS One. 2020 Oct 2;15(10):e0234676. doi: 10.1371/journal.pone.0234676 (PMC7531853; doi:10.1371/journal.pone.0234676)
Supplement: S26 Table — Means (SE) for FxDxA for Prime female mass and age, and Average female mass. Total food and food/larva after day 4. (DOCX) [file pone.0234676.s067.docx]

S26 Table. Means (SE) for FxDxA for Prime female mass and age, and Average female mass. Total food and food/larva after day 4.

| Food x Density | Aliquot | Rank by Prime female mass (a-h) | Prime female mass at pupation (mg) | Prime female age at pupation (days) | Average female mass at pupation (mg) | Total food after day 4 (mg) | Food/larva after day 4 (mg) |
| --- | --- | --- | --- | --- | --- | --- | --- |
| Low food, low density (4 mg/larva) | 2 aliquots | f | 3.86 (0.83) | 6.85 (1.35) | 3.60 (0.88) | 12, 16 | 3, 4 |
|  | 4 aliquots | d | 4.14 (0.37) | 6.24 (0.51) | 3.99 (0.42) | 14, 16 | 3.5, 4 |
| Most competition (2 mg/larva) | 2 aliquots | h | 2.86 (0.15) | 8.95 (2.33) | 2.63 (0.20) | 12, 16 | 1.5, 2 |
|  | 4 aliquots | g | 2.92 (0.17) | 7.28 (1.18) | 2.74 (0.13) | 14, 16 | 1.75, 2 |
| Least competition (8 mg/larva) | 2 aliquots | b | 4.53 (0.52) | 5.36 (0.20) | 4.40 (0.52) | 24, 32 | 6, 8 |
|  | 4 aliquots | a | 4.81 (0.08) | 5.71 (0.00) | 4.69 (0.04) | 28, 32 | 7, 8 |
| High food, high density (4 mg/larva) | 2 aliquots | e | 4.01 (0.66) | 6.20 (1.13) | 3.60 (0.85) | 24, 32 | 3, 4 |
|  | 4 aliquots | c | 4.49 (0.29) | 5.88 (0.35) | 4.24 (0.25) | 28, 32 | 3.5, 4 |
