# Supplementary material for: Competition and growth among Aedes aegypti larvae: Effects of distributing food inputs over time
Source: PLoS One. 2020 Oct 2;15(10):e0234676. doi: 10.1371/journal.pone.0234676 (PMC7531853; doi:10.1371/journal.pone.0234676)
Supplement: S27 Table — Means (SE) for FxD for Prime female mass and age, and Average female mass. Expected mean values for Prime female age and Average female mass. (DOCX) [file pone.0234676.s068.docx]

S27 Table. Means (SE) for FxD for Prime female mass and age at pupation and Average female mass. Expected mean values for Prime female age and Average female mass.

| Food x Density | Prime female mass at pupation (mg) | Prime female age at pupation (days) | Average female mass at pupation (mg) | Estimated Prime female growth rate (mg/day) | Prime female mass MINUS Average female mass (mg) | Expected mean values for Prime female age at pupation (days) | Expected mean values for Average female mass at pupation (mg) |
| --- | --- | --- | --- | --- | --- | --- | --- |
| Low food, low density (4 mg/larva) | 4.00 (0.55) | 6.54 (0.90) | 3.79 (0.61) | 0.61 (0.32) | 0.21 (0.41) | 6.69 (1.38) | 3.71 (0.81) |
| Most competition (2 mg/larva) | 2.89 (0.14) | 8.11 (1.79) | 2.68 (0.15) | 0.36 (0.32) | 0.21 (0.10) | 7.21 (1.38) | 3.27 (0.81) |
| Least competition (8 mg/larva) | 4.67 (0.34) | 5.54 (0.23) | 4.54 (0.34) | 0.84 (0.17) | 0.13 (0.24) | 5.92 (1.38) | 4.20 (0.81) |
| High food, high density (4 mg/larva) | 4.25 (0.50) | 6.04 (0.71) | 3.92 (0.63) | 0.70 (0.31) | 0.33 (0.40) | 6.44 (1.38) | 3.77 (0.81) |
