# Supplementary material for: Competition and growth among Aedes aegypti larvae: Effects of distributing food inputs over time
Source: PLoS One. 2020 Oct 2;15(10):e0234676. doi: 10.1371/journal.pone.0234676 (PMC7531853; doi:10.1371/journal.pone.0234676)
Supplement: S28 Table — Means (SE) for FxD for Prime male mass and age, and Average male mass. Expected mean values for Prime male age and Average male mass. (DOCX) [file pone.0234676.s069.docx]

S28 Table. Means (SE) for Prime male mass and age at pupation and Average male mass at pupation for the interaction FxD.

| Food x Density | Prime male mass at pupation (mg) | Prime male age at pupation (days) | Average male mass at pupation (mg) | Estimated Prime male growth rate (mg/day) | Prime male MINUS Average male mass (mg) | Expected mean values for Prime male age at pupation (days) | Expected mean values for Average male mass at pupation (mg) |
| --- | --- | --- | --- | --- | --- | --- | --- |
| Low food, low density (4 mg/larva) | 2.44 (0.31) | 5.06 (0.07) | 2.40 (0.35) | 0.48 (0.08) | 0.04 (0.23) | 5.14 (0.24) | 2.32 (0.39) |
| Most competition (2 mg/larva) | 1.81 (0.40) | 5.40 (0.39) | 1.82 (0.23) | 0.34 (0.09) | -0.01 (0.23) | 5.22 (0.24) | 2.11 (0.39) |
| Least competition (8 mg/larva) | 2.74 (0.14) | 5.04 (0.07) | 2.67 (0.16) | 0.54 (0.04) | 0.07 (0.11) | 5.04 (0.24) | 2.53 (0.39) |
| High food, high density (4 mg/larva) | 2.54 (0.27) | 5.00 (0.00) | 2.39 (0.25) | 0.51 (0.07) | 0.15 (0.18) | 5.11 (0.24) | 2.32 (0.39) |
