# Supplementary material for: Competition and growth among Aedes aegypti larvae: Effects of distributing food inputs over time
Source: PLoS One. 2020 Oct 2;15(10):e0234676. doi: 10.1371/journal.pone.0234676 (PMC7531853; doi:10.1371/journal.pone.0234676)
Supplement: S29 Table — Means (SE) for FxAxT for Prime female mass and age, and Average female mass. Estimated growth rate and difference between the Prime and Average female mass. (DOCX) [file pone.0234676.s070.docx]

S29 Table. Means (SE) for Prime female mass and age at pupation and Average female mass at pupation for the interaction FxAxT. Estimated growth rate and difference between the Prime and Average female mass.

| Food x Aliquot | Timespan | Rank by Prime female mass (a-h) | Prime female mass at pupation (mg) | Prime female age at pupation (days) | Average female mass at pupation (mg) | Estimated growth rate for Prime female (mg/day) | Prime female mass MINUS Average female mass (mg) |
| --- | --- | --- | --- | --- | --- | --- | --- |
| Low food, 2 aliquots | 3 days | f | 3.70 (1.05) | 6.60 (1.00) | 3.50 (1.03) | 0.56 (0.57) | 0.20 (1.04) |
|  | 6 days | h | 3.01 (0.37) | 9.20 (1.98) | 2.73 (0.34) | 0.33 (0.47) | 0.28 (0.36) |
| Low food, 4 aliquots | 3 days | e | 3.72 (0.96) | 6.16 (0.40) | 3.56 (1.03) | 0.60 (0.44) | 0.16 (1.00) |
|  | 6 days | g | 3.34 (0.76) | 7.36 (1.07) | 3.17 (0.74) | 0.45 (0.42) | 0.17 (0.75) |
| High food, 2 aliquots | 3 days | b | 4.68 (0.30) | 5.31 (0.13) | 4.48 (0.40) | 0.88 (0.20) | 0.20 (0.35) |
|  | 6 days | d | 3.85 (0.44) | 6.25 (1.06) | 3.52 (1.06) | 0.62 (0.50) | 0.33 (0.60) |
| High food, 4 aliquots | 3 days | a | 4.72 (0.04) | 5.67 (0.06) | 4.57 (0.21) | 0.83 (0.04) | 0.15 (0.15) |
|  | 6 days | c | 4.57 (0.41) | 5.92 (0.30) | 4.36 (0.42) | 0.77 (0.28) | 0.21 (0.42) |
