# Supplementary material for: Competition and growth among Aedes aegypti larvae: Effects of distributing food inputs over time
Source: PLoS One. 2020 Oct 2;15(10):e0234676. doi: 10.1371/journal.pone.0234676 (PMC7531853; doi:10.1371/journal.pone.0234676)
Supplement: S30 Table — Means (SE) for FxAxT for Prime female mass and age, and Average female mass. Total food and food/larva after day 4. (DOCX) [file pone.0234676.s071.docx]

S30 Table. Means (SE) for Prime female mass and age at pupation and Average female mass at pupation for the interaction FxAxT. Total food and food/larva after day 4.

| Food x Aliquot | Timespan | Rank by Prime female mass (a-h) | Prime female mass at pupation (mg) | Prime female age at pupation (days) | Average female mass at pupation (mg) | Total food after day 4 (mg) | Food/larva after day 4 (mg) |
| --- | --- | --- | --- | --- | --- | --- | --- |
| Low food, 2 aliquots | 3 days | f | 3.70 (1.05) | 6.60 (1.00) | 3.50 (1.03) | 16 | 2, 4 |
|  | 6 days | h | 3.01 (0.37) | 9.20 (1.98) | 2.73 (0.34) | 8 | 1, 2 |
| Low food, 4 aliquots | 3 days | e | 3.72 (0.96) | 6.16 (0.40) | 3.56 (1.03) | 16 | 2, 4 |
|  | 6 days | g | 3.34 (0.76) | 7.36 (1.07) | 3.17 (0.74) | 12 | 1.5, 3 |
| High food, 2 aliquots | 3 days | b | 4.68 (0.30) | 5.31 (0.13) | 4.48 (0.40) | 32 | 4, 8 |
|  | 6 days | d | 3.85 (0.44) | 6.25 (1.06) | 3.52 (1.06) | 16 | 2, 4 |
| High food, 4 aliquots | 3 days | a | 4.72 (0.04) | 5.67 (0.06) | 4.57 (0.21) | 32 | 4, 8 |
|  | 6 days | c | 4.57 (0.41) | 5.92 (0.30) | 4.36 (0.42) | 24 | 3, 6 |
