# Supplementary material for: Competition and growth among Aedes aegypti larvae: Effects of distributing food inputs over time
Source: PLoS One. 2020 Oct 2;15(10):e0234676. doi: 10.1371/journal.pone.0234676 (PMC7531853; doi:10.1371/journal.pone.0234676)
Supplement: S31 Table — Means (SE) for FxA for Prime male mass and age, and Average male mass. Expected mean values for the Prime male mass and age and the Average male mass. (DOCX) [file pone.0234676.s072.docx]

S31 Table. Means (SE) for Prime male mass and age at pupation and Average male mass at pupation for the interaction FxA. Expected mean values for the Prime male mass and age and the Average male mass.

| Food x Aliquot | Prime male mass at pupation (mg) | Prime male age at pupation (days) | Average male mass at pupation (mg) | Prime male growth rate (mg/day) | Prime male mass MINUS Average male mass (mg) | Expected mean value for Prime male mass at pupation (mg) | Expected mean value for Prime male age at pupation (days) | Expected mean value for Average male mass at pupation (mg) |
| --- | --- | --- | --- | --- | --- | --- | --- | --- |
| 16 mg, 2 aliquots | 2.00 (0.57) | 5.20 (0.21) | 2.00 (0.45) | 0.38 (0.17) | 0.00 (0.36) | 2.21 (0.44) | 5.17 (0.24) | 2.17 (0.39) |
| 16 mg, 4 aliquots | 2.25 (0.40) | 5.26 (0.43) | 2.22 (0.40) | 0.43 (0.23) | 0.03 (0.28) | 2.31 (0.44) | 5.19 (0.24) | 2.26 (0.39) |
| 32 mg, 2 aliquots | 2.57 (0.29) | 5.00 (0.00) | 2.46 (0.29) | 0.51 (0.07) | 0.11 (0.21) | 2.46 (0.44) | 5.06 (0.24) | 2.38 (0.39) |
| 32 mg, 4 aliquots | 2.71 (0.16) | 5.04 (0.07) | 2.60 (0.20) | 0.54 (0.05) | 0.11 (0.13) | 2.56 (0.44) | 5.09 (0.24) | 2.47 (0.39) |
