# Supplementary material for: Competition and growth among Aedes aegypti larvae: Effects of distributing food inputs over time
Source: PLoS One. 2020 Oct 2;15(10):e0234676. doi: 10.1371/journal.pone.0234676 (PMC7531853; doi:10.1371/journal.pone.0234676)
Supplement: S32 Table — Means (SE) for Prime female mass and Average female mass at pupation for the interaction FxA. Differences between the Prime female mass and the Prime male mass and between the Average female mass and the Average male mass. (DOCX) [file pone.0234676.s073.docx]

S32 Table. Means (SE) for Prime female mass and Average female mass at pupation for the interaction FxA. Differences between the Prime female mass and the Prime male mass and between the Average female mass and the Average male mass.

| Food x Aliquot | Prime female mass at pupation (mg) | Prime female age at pupation (days) | Average female mass at pupation (mg) | Prime female mass MINUS Prime male mass (mg) | Average female mass MINUS Average male mass (mg) |
| --- | --- | --- | --- | --- | --- |
| 16 mg, 2 aliquots | 3.36 (0.75) | 7.90 (1.97) | 3.11 (0.76) | 1.36 (0.47) | 1.11 (0.44) |
| 16 mg, 4 aliquots | 3.53 (0.74) | 6.76 (0.95) | 3.36 (0.76) | 1.38 (0.42) | 1.14 (0.43) |
| 32 mg, 2 aliquots | 4.27 (0.57) | 5.78 (0.82) | 4.00 (0.73) | 1.70 (0.32) | 1.54 (0.39) |
| 32 mg, 4 aliquots | 4.65 (0.25) | 5.80 (0.23) | 4.46 (0.30) | 1.94 (0.15) | 1.86 (0.18) |
