# Supplementary material for: Competition and growth among Aedes aegypti larvae: Effects of distributing food inputs over time
Source: PLoS One. 2020 Oct 2;15(10):e0234676. doi: 10.1371/journal.pone.0234676 (PMC7531853; doi:10.1371/journal.pone.0234676)
Supplement: S33 Table — Means (SE) for arcsin transformed percent Survival for the interaction FxT. (DOCX) [file pone.0234676.s074.docx]

S33 Table. Means (SE) for arcsin transformed percent Survival for the interaction FxT.

| Food x Timespan | Survival |
| --- | --- |
| 16 mg, 3 days | 1.20 (0.16) |
| 16 mg, 6 days | 1.18 (0.22) |
| 32 mg, 3 days | 1.14 (0.16) |
| 32 mg, 6 days | 1.32 (0.12) |
