# Supplementary material for: Competition and growth among Aedes aegypti larvae: Effects of distributing food inputs over time
Source: PLoS One. 2020 Oct 2;15(10):e0234676. doi: 10.1371/journal.pone.0234676 (PMC7531853; doi:10.1371/journal.pone.0234676)
Supplement: S34 Table — Means (SE) for Prime female mass and age and Average female mass for the interaction FxT. Total food, expected values, growth rates and the differences between Prime and Average female masses. (DOCX) [file pone.0234676.s075.docx]

S34 Table. Means (SE) for Prime female mass and age and Average female mass for the interaction FxT. Total food, expected values, growth rates and the differences between Prime and Average female masses.

| Food x Timespan | Prime female mass at pupation (mg) | Prime female age at pupation (days) | Average female mass at pupation (mg) | Estimated growth rate (mg/day) | Prime female mass MINUS Average female mass (mg) | Total food after day 4 (mg) | Expected mean values for Prime female mass (mg) | Expected mean values for Average female mass (mg) |
| --- | --- | --- | --- | --- | --- | --- | --- | --- |
| 16 mg, 3 days | 3.71 (0.82) | 6.38 (0.67) | 3.53 (0.84) | 0.58 (0.56) | 0.18 (0.59) | 16 | 3.83 (0.77) | 3.63 (0.81) |
| 16 mg, 6 days | 3.18 (0.53) | 8.28 (1.68) | 2.95 (0.53) | 0.38 (0.95) | 0.23 (0.37) | 8, 12 | 3.57 (0.77) | 3.34 (0.81) |
| 32 mg, 3 days | 4.70 (0.17) | 5.49 (0.22) | 4.52 (0.26) | 0.86 (0.14) | 0.18 (0.16) | 32 | 4.34 (0.77) | 4.13 (0.81) |
| 32 mg, 6 days | 4.21 (0.54) | 6.09 (0.66) | 3.94 (0.69) | 0.69 (0.46) | 0.27 (0.44) | 16, 24 | 4.08 (0.77) | 3.84 (0.81) |
