# Supplementary material for: Competition and growth among Aedes aegypti larvae: Effects of distributing food inputs over time
Source: PLoS One. 2020 Oct 2;15(10):e0234676. doi: 10.1371/journal.pone.0234676 (PMC7531853; doi:10.1371/journal.pone.0234676)
Supplement: S35 Table — Means (SE) for Prime male mass and age and Average male mass for the interaction FxT. Expected values, growth rates and the differences between Prime and Average female masses. (DOCX) [file pone.0234676.s076.docx]

S35 Table. Means (SE) for Prime male mass and age and Average male mass for the interaction FxT. Expected values, growth rates and the differences between Prime and Average female masses.

| Food x Timespan | Prime male mass at pupation (mg) | Prime male age at pupation (days) | Average male mass at pupation (mg) | Estimated growth rate (mg/day) | Prime male mass MINUS Average male mass (mg) | Expected mean values for Prime male mass (mg) | Expected mean values for Prime male age (days) | Expected mean values for Average male mass (mg) |
| --- | --- | --- | --- | --- | --- | --- | --- | --- |
| 16 mg, 3 days | 2.38 (0.30) | 5.11 (0.08) | 2.30 (0.36) | 0.47 (0.16) | 0.08 (0.23) | 2.35 (0.44) | 5.15 (0.24) | 2.28 (0.39) |
| 16 mg, 6 days | 1.88 (0.51) | 5.35 (0.43) | 1.92 (0.42) | 0.35 (0.33) | -0.04 (0.33) | 2.17 (0.44) | 5.20 (0.24) | 2.15 (0.39) |
| 32 mg, 3 days | 2.75 (0.05) | 5.04 (0.07) | 2.61 (0.07) | 0.55 (0.05) | 0.14 (0.04) | 2.60 (0.44) | 5.05 (0.24) | 2.49 (0.39) |
| 32 mg, 6 days | 2.53 (0.29) | 5.00 (0.00) | 2.45 (0.35) | 0.51 (0.15) | 0.08 (0.23) | 2.42 (0.44) | 5.10 (0.24) | 2.36 (0.39) |
