# Supplementary material for: Competition and growth among Aedes aegypti larvae: Effects of distributing food inputs over time
Source: PLoS One. 2020 Oct 2;15(10):e0234676. doi: 10.1371/journal.pone.0234676 (PMC7531853; doi:10.1371/journal.pone.0234676)
Supplement: S36 Table — Means (SE) for Prime male mass and age and Average male mass for the interaction FxT. Differences between the Prime female mass and Prime male mass and between the Average female mass and the Average male mass. (DOCX) [file pone.0234676.s077.docx]

S36 Table. Means (SE) for Prime male mass and age and Average male mass for the interaction FxT. Differences between the Prime female mass and Prime male mass and between the Average female mass and the Average male mass.

| Food x Timespan | Prime male mass at pupation (mg) | Prime male age at pupation (days) | Average male mass at pupation (mg) | Prime female mass MINUS Prime male mass (mg) | Average female mass MINUS Average male mass (mg) |
| --- | --- | --- | --- | --- | --- |
| 16 mg, 3 days | 2.38 (0.30) | 5.11 (0.08) | 2.30 (0.36) | 1.33 (0.44) | 1.23 (0.46) |
| 16 mg, 6 days | 1.88 (0.51) | 5.35 (0.43) | 1.92 (0.42) | 1.30 (0.37) | 1.03 (0.34) |
| 32 mg, 3 days | 2.75 (0.05) | 5.04 (0.07) | 2.61 (0.07) | 1.95 (0.09) | 1.91 (0.13) |
| 32 mg, 6 days | 2.53 (0.29) | 5.00 (0.00) | 2.45 (0.35) | 1.68 (0.31) | 1.49 (0.39) |
