# Supplementary material for: Competition and growth among Aedes aegypti larvae: Effects of distributing food inputs over time
Source: PLoS One. 2020 Oct 2;15(10):e0234676. doi: 10.1371/journal.pone.0234676 (PMC7531853; doi:10.1371/journal.pone.0234676)
Supplement: S37 Table — Means (SE) for Prime female mass and age at pupation and Average female mass at pupation for the interaction AxT. Expected values, growth rates and the differences between the Prime female mass and the Average female mass. (DOCX) [file pone.0234676.s078.docx]

S37 Table. Means (SE) for Prime female mass and age at pupation and Average female mass at pupation for the interaction AxT. Expected values, growth rates and the differences between the Prime female mass and the Average female mass.

| Aliquot x Timespan | Prime female mass at pupation (mg) | Prime female age at pupation (days) | Average female mass at pupation (mg) | Estimated growth rate for Prime females (mg/day) | Prime female mass MINUS Average female mass (mg) | Expected mean values for Prime female mass at pupation (mg) | Expected mean values for Average female mass at pupation (mg) |
| --- | --- | --- | --- | --- | --- | --- | --- |
| 2 aliquots, 3 days | 4.19 (0.85) | 5.95 (0.94) | 3.99 (0.85) | 0.70 (0.09) | 0.20 (0.63) | 4.01 (0.77) | 3.79 (0.81) |
| 2 aliquots, 6 days | 3.43 (0.59) | 7.73 (2.14) | 3.12 (0.65) | 0.44 (0.07) | 0.31 (1.11) | 3.75 (0.77) | 3.50 (0.81) |
| 4 aliquots, 3 days | 4.22 (0.80) | 5.92 (0.37) | 4.06 (0.84) | 0.71 (0.07) | 0.16 (0.44) | 4.15 (0.77) | 3.97 (0.81) |
| 4 aliquots, 6 days | 3.96 (0.87) | 6.64 (1.05) | 3.76 (0.85) | 0.60 (0.08) | 0.20 (0.68) | 3.89 (0.77) | 3.68 (0.81) |
