# Supplementary material for: Competition and growth among Aedes aegypti larvae: Effects of distributing food inputs over time
Source: PLoS One. 2020 Oct 2;15(10):e0234676. doi: 10.1371/journal.pone.0234676 (PMC7531853; doi:10.1371/journal.pone.0234676)
Supplement: S38 Table — Means (SE) for Prime male mass and age at pupation and Average male mass at pupation for the interaction AxT. Expected values, growth rates and the differences between the Prime male mass and the Average male mass. (DOCX) [file pone.0234676.s079.docx]

S38 Table. Means (SE) for Prime male mass and age at pupation and Average male mass at pupation for the interaction AxT. Expected values, growth rates and the differences between the Prime male mass and the Average male mass.

| Aliquot x Timespan | Prime male mass at pupation (mg) | Prime male age at pupation (days) | Average male mass at pupation (mg) | Estimated growth rate for Prime males (mg/day) | Prime male mass MINUS Average male mass (mg) | Expected mean values for Prime male mass at pupation (mg) | Expected mean values for Prime male age at pupation (days) | Expected mean values for Average male mass at pupation (mg) |
| --- | --- | --- | --- | --- | --- | --- | --- | --- |
| 2 aliquots, 3 days | 2.57 (0.33) | 5.08 (0.10) | 2.47 (0.33) | 0.51 (0.03) | 0.10 (0.17) | 2.42 (0.44) | 5.09 (0.24) | 2.34 (0.39) |
| 2 aliquots, 6 days | 2.00 (0.53) | 5.13 (0.25) | 1.98 (0.41) | 0.39 (0.05) | 0.02 (0.29) | 2.24 (0.44) | 5.14 (0.24) | 2.21 (0.39) |
| 4 aliquots, 3 days | 2.55 (0.27) | 5.07 (0.08) | 2.44 (0.29) | 0.50 (0.03) | 0.11 (0.14) | 2.52 (0.44) | 5.11 (0.24) | 2.43 (0.39) |
| 4 aliquots, 6 days | 2.41 (0.48) | 5.22 (0.45) | 2.38 (0.46) | 0.46 (0.05) | 0.03 (0.33) | 2.34 (0.44) | 5.16 (0.24) | 2.30 (0.39) |
