# Supplementary material for: Competition and growth among Aedes aegypti larvae: Effects of distributing food inputs over time
Source: PLoS One. 2020 Oct 2;15(10):e0234676. doi: 10.1371/journal.pone.0234676 (PMC7531853; doi:10.1371/journal.pone.0234676)
Supplement: S39 Table — Means (SE) for Prime female mass for the interaction AxT. Differences between Prime female mass and Average female mass, Prime male mass and Average male mass, Prime female mass and Prime male mass, and Average female mass and Average male mass. Total food after day 4 and food/larva after day 4. (DOCX) [file pone.0234676.s080.docx]

S39 Table. Means (SE) for Prime female mass for the interaction AxT. Differences between Prime female mass and Average female mass, Prime male mass and Average male mass, Prime female mass and Prime male mass, and Average female mass and Average male mass. Total food after day 4 and food/larva after day 4.

| Aliquot x Timespan | Prime female mass at pupation (mg) | Prime female mass MINUS Average female mass (mg) | Prime male mass MINUS Average male mass (mg) | Prime female mass MINUS Prime male mass (mg) | Average female mass MINUS Average male mass (mg) | Total food after day 4 (mg) | Food/larva after day 4 (mg) | Average food/larva after day 4 (mg) |
| --- | --- | --- | --- | --- | --- | --- | --- | --- |
| 2 aliquots, 3 days | 4.19 (0.85) | 0.20 (0.63) | 0.10 (0.17) | 1.62 (0.46) | 1.52 (0.46) | 16, 32 | 4, 2, 8, 4 | 4.5 |
| 2 aliquots, 6 days | 3.43 (0.59) | 0.31 (1.11) | 0.02 (0.29) | 1.43 (0.40) | 1.14 (0.38) | 8, 16 | 2, 1, 4, 2 | 2.25 |
| 4 aliquots, 3 days | 4.22 (0.80) | 0.16 (0.44) | 0.11 (0.14) | 1.67 (0.42) | 1.62 (0.44) | 16, 32 | 4, 2, 8, 4 | 4.5 |
| 4 aliquots, 6 days | 3.96 (0.87) | 0.20 (0.68) | 0.03 (0.33) | 1.55 (0.50) | 1.38 (0.48) | 12, 24 | 3, 1.5, 6, 3 | 3.375 |
