# Supplementary material for: Competition and growth among Aedes aegypti larvae: Effects of distributing food inputs over time
Source: PLoS One. 2020 Oct 2;15(10):e0234676. doi: 10.1371/journal.pone.0234676 (PMC7531853; doi:10.1371/journal.pone.0234676)
Supplement: S40 Table — Means (SE) for Prime female mass and age at pupation and Average female mass at pupation for the interaction DxAxT. Estimated growth rates and the differences between the Prime female mass and the Average female mass. Total food after day 4 and food/larva after day 4. (DOCX) [file pone.0234676.s081.docx]

S40 Table. Means (SE) for Prime female mass and age at pupation and Average female mass at pupation for the interaction DxAxT. Estimated growth rates and the differences between the Prime female mass and the Average female mass. Total food after day 4 and food/larva after day 4.

| Density x Aliquot | Timespan | Rank by Prime female mass | Prime female mass at pupation (mg) | Prime female age at pupation (days) | Average female mass at pupation (mg) | Estimated growth rate of Prime female (mg/day) | Prime female mass MINUS Average female mass (mg) | Total food after day 4 (mg) | Food/larva after day 4 (mg) |
| --- | --- | --- | --- | --- | --- | --- | --- | --- | --- |
| 4 larvae, 2 aliquots | 3 days | a | 4.67 (0.32) | 5.56 (0.47) | 4.49 (0.38) | 0.84 (0.34) | 0.18 (0.35) | 16, 32 | 4, 8 |
|  | 6 days | ef | 3.72 (0.63) | 6.65 (0.63) | 3.50 (0.75) | 0.56 (0.69) | 0.22 (0.69) | 8, 16 | 2, 4 |
| 4 larvae, 4 aliquots | 3 days | b | 4.58 (0.25) | 5.80 (0.12) | 4.50 (0.30) | 0.79 (0.15) | 0.08 (0.28) | 16, 32 | 4, 8 |
|  | 6 days | c | 4.37 (0.69) | 6.16 (0.63) | 4.18 (0.69) | 0.71 (0.47) | 0.19 (0.69) | 12, 24 | 3, 6 |
| 8 larvae, 2 aliquots | 3 days | ef | 3.72 (1.07) | 6.35 (1.34) | 3.49 (1.01) | 0.59 (0.71) | 0.23 (1.04) | 16, 32 | 2, 4 |
|  | 6 days | h | 3.15 (0.56) | 8.80 (2.55) | 2.75 (0.36) | 0.36 (0.66) | 0.40 (0.47) | 8, 16 | 1, 2 |
| 8 larvae, 4 aliquots | 3 days | d | 3.87 (1.17) | 6.04 (0.57) | 3.63 (1.12) | 0.64 (0.59) | 0.24 (1.15) | 16, 32 | 2, 4 |
|  | 6 days | g | 3.54 (1.05) | 7.12 (1.40) | 3.35 (1.00) | 0.50 (0.62) | 0.19 (1.03) | 12, 24 | 1.5, 3 |
