# Supplementary material for: Competition and growth among Aedes aegypti larvae: Effects of distributing food inputs over time
Source: PLoS One. 2020 Oct 2;15(10):e0234676. doi: 10.1371/journal.pone.0234676 (PMC7531853; doi:10.1371/journal.pone.0234676)
Supplement: S41 Table — Means (SE) for Prime male mass and age at pupation and Average male mass at pupation for the interaction DxAxT. Estimated growth rates and the differences between the Prime male mass and the Average male mass. (DOCX) [file pone.0234676.s082.docx]

S41 Table. Means (SE) for Prime male mass and age at pupation and Average male mass at pupation for the interaction DxAxT. Estimated growth rates and the differences between the Prime male mass and the Average male mass.

| Density x Aliquot | Timespan | Rank by Prime male mass | Prime male mass at pupation (mg) | Prime male age at pupation (days) | Average male mass at pupation (mg) | Estimated growth rate of Prime male (mg/day) | Prime male mass MINUS Average male mass (mg) |
| --- | --- | --- | --- | --- | --- | --- | --- |
| 4 larvae, 2 aliquots | 3 days | a | 2.71 (0.07) | 5.06 (0.08) | 2.66 (0.06) | 0.54 (0.04) | 0.05 (0.07) |
|  | 6 days | f | 2.27 (0.39) | 5.00 (0.00) | 2.19 (0.44) | 0.45 (0.13) | 0.08 (0.42) |
| 4 larvae, 4 aliquots | 3 days | c | 2.69 (0.11) | 5.14 (0.01) | 2.60 (0.01) | 0.52 (0.04) | 0.09 (0.08) |
|  | 6 days | b | 2.70 (0.26) | 5.00 (0.00) | 2.69 (0.26) | 0.54 (0.10) | 0.01 (0.26) |
| 8 larvae, 2 aliquots | 3 days | d | 2.44 (0.50) | 5.10 (0.14) | 2.29 (0.43) | 0.48 (0.18) | 0.15 (0.47) |
|  | 6 days | h | 1.73 (0.63) | 5.25 (0.35) | 1.78 (0.36) | 0.33 (0.17) | -0.05 (0.51) |
| 8 larvae, 4 aliquots | 3 days | e | 2.41 (0.37) | 5.00 (0.00) | 2.28 (0.37) | 0.48 (0.13) | 0.13 (0.37) |
|  | 6 days | g | 2.12 (0.55) | 5.45 (0.63) | 2.08 (0.43) | 0.39 (0.23) | 0.04 (0.49) |
