# Supplementary material for: Competition and growth among Aedes aegypti larvae: Effects of distributing food inputs over time
Source: PLoS One. 2020 Oct 2;15(10):e0234676. doi: 10.1371/journal.pone.0234676 (PMC7531853; doi:10.1371/journal.pone.0234676)
Supplement: S42 Table — Means (SE) for Prime female mass and Average male mass for the interaction DxAxT. Prime female mass MINUS Average female mass, Prime female mass MINUS Prime male mass, Prime male mass MINUS Average male mass, Average female mass MINUS Average male mass. (DOCX) [file pone.0234676.s083.docx]

S42 Table. Means (SE) for Prime female mass and Average male mass for the interaction DxAxT. Prime female mass MINUS Average female mass, Prime female mass MINUS Prime male mass, Prime male mass MINUS Average male mass, Average female mass MINUS Average male mass.

| Density x Aliquot | Timespan | Prime female mass at pupation (mg) | Average male mass at pupation (mg) | Prime female mass MINUS Average female mass (mg) | Prime female mass MINUS Prime male mass (mg) | Prime male mass MINUS Average male mass (mg) | Average female mass MINUS Average male mass (mg) |
| --- | --- | --- | --- | --- | --- | --- | --- |
| 4 larvae, 2 aliquots | 3 days | 4.67 (0.32) | 2.66 (0.06) | 0.18 (0.35) | 1.96 (0.23) | 0.05 (0.07) | 1.83 (0.27) |
|  | 6 days | 3.72 (0.63) | 2.19 (0.44) | 0.22 (0.69) | 1.45 (0.52) | 0.08 (0.42) | 1.31 (0.61) |
| 4 larvae, 4 aliquots | 3 days | 4.58 (0.25) | 2.60 (0.01) | 0.08 (0.28) | 1.89 (0.19) | 0.09 (0.08) | 1.90 (0.21) |
|  | 6 days | 4.37 (0.69) | 2.69 (0.26) | 0.19 (0.69) | 1.67 (0.52) | 0.01 (0.26) | 1.49 (0.52) |
| 8 larvae, 2 aliquots | 3 days | 3.72 (1.07) | 2.29 (0.43) | 0.23 (1.04) | 1.28 (0.84) | 0.15 (0.47) | 1.20 (0.78) |
|  | 6 days | 3.15 (0.56) | 1.78 (0.36) | 0.40 (0.47) | 1.42 (0.60) | -0.05 (0.51) | 0.97 (0.36) |
| 8 larvae, 4 aliquots | 3 days | 3.87 (1.17) | 2.28 (0.37) | 0.24 (1.15) | 1.46 (0.87) | 0.13 (0.37) | 1.35 (0.83) |
|  | 6 days | 3.54 (1.05) | 2.08 (0.43) | 0.19 (1.03) | 1.42 (0.84) | 0.04 (0.49) | 1.27 (0.77) |
