# Supplementary material for: Competition and growth among Aedes aegypti larvae: Effects of distributing food inputs over time
Source: PLoS One. 2020 Oct 2;15(10):e0234676. doi: 10.1371/journal.pone.0234676 (PMC7531853; doi:10.1371/journal.pone.0234676)
Supplement: S43 Table — Means (SE) for Prime female mass and age at pupation and Average female mass at pupation for the interaction DxA. Estimated growth rates, differences between Prime and Average female masses, expected mean values for Prime female age, and food levels after day 4 and on day 0. (DOCX) [file pone.0234676.s084.docx]

S43 Table. Means (SE) for Prime female mass and age at pupation and Average female mass at pupation for the interaction DxA. Estimated growth rates, differences between Prime and Average female masses, expected mean values for Prime female age, and food levels after day 4 and on day 0.

| Density x Aliquot | Prime female mass at pupation (mg) | Prime female age at pupation (days) | Average female mass at pupation (mg) | Estimated growth rate (mg/day) | Prime female mass MINUS Average female mass (mg) | Expected mean values for Prime female age (days) | Total food after day 4 (mg) | Food/larva after day 4 (mg) | Food/larva on day 0 (mg) |
| --- | --- | --- | --- | --- | --- | --- | --- | --- | --- |
| 4 larvae, 2 aliquots | 4.19 (0.68) | 6.10 (1.16) | 4.00 (0.75) | 0.69 (0.46) | 0.19 (0.51) | 6.44 (1.38) | 8, 16, 16, 32 | 2, 4, 4, 8 | 2, 4 |
| 4 larvae, 4 aliquots | 4.47 (0.44) | 5.98 (0.42) | 4.34 (0.47) | 0.75 (0.23) | 0.13 (0.32) | 6.16 (1.38) | 12, 24, 16, 32 | 3, 6, 4, 8 | 1, 2 |
| 8 larvae, 2 aliquots | 3.43 (0.77) | 7.58 (2.18) | 3.12 (0.75) | 0.45 (0.52) | 0.31 (0.54) | 6.96 (1.38) | 8, 16, 16, 32 | 1, 2, 2, 4 | 1, 2 |
| 8 larvae, 4 aliquots | 3.70 (0.92) | 6.58 (1.07) | 3.49 (0.88) | 0.56 (0.40) | 0.21 (0.64) | 6.68 (1.38) | 12, 24, 16, 32 | 1.5, 3, 2, 4 | 0.5, 1 |
