# Supplementary material for: Competition and growth among Aedes aegypti larvae: Effects of distributing food inputs over time
Source: PLoS One. 2020 Oct 2;15(10):e0234676. doi: 10.1371/journal.pone.0234676 (PMC7531853; doi:10.1371/journal.pone.0234676)
Supplement: S44 Table — Means (SE) for arcsin transformed percent Survival for the interaction DxT. Expected values. (DOCX) [file pone.0234676.s085.docx]

S44 Table. Means (SE) for arcsin transformed percent Survival for the interaction DxT. Expected values.

| Density x Timespan | Survival | Expected mean values of Survival |
| --- | --- | --- |
| 4 larvae, 3 days | 1.23 (0.13) | 1.24 (0.17) |
| 4 larvae, 6 days | 1.36 (0.11) | 1.28 (0.17) |
| 8 larvae, 3 days | 1.11 (0.17) | 1.15 (0.17) |
| 8 larvae, 6 days | 1.14 (0.18) | 1.19 (0.17) |
