# Supplementary material for: Competition and growth among Aedes aegypti larvae: Effects of distributing food inputs over time
Source: PLoS One. 2020 Oct 2;15(10):e0234676. doi: 10.1371/journal.pone.0234676 (PMC7531853; doi:10.1371/journal.pone.0234676)
Supplement: S45 Table — Means (SE) for Prime female mass and age at pupation and Average female mass at pupation for the interaction DxT. Expected mean values, growth rates, differences between Prime and Average female masses, food levels. (DOCX) [file pone.0234676.s086.docx]

S45 Table. Means (SE) for Prime female mass and age at pupation and Average female mass at pupation for the interaction DxT. Expected mean values, growth rates, differences between Prime and Average female masses, food levels.

| Density x Timespan | Prime female mass at pupation (mg) | Prime female age at pupation (days) | Average female mass at pupation (mg) | Estimated Prime female growth rate (mg/day) | Prime female mass MINUS Average female mass (mg) | Expected mean values for Prime female mass at pupation (mg) | Expected mean values for Prime female age at pupation (days) | Expected mean values for Average female mass at pupation (mg) | Total food after day 4 (mg) | Food/larva after day 4 (mg) |
| --- | --- | --- | --- | --- | --- | --- | --- | --- | --- | --- |
| 4 larvae, 3 days | 4.62 (0.24) | 5.68 (0.31) | 4.49 (0.28) | 0.81 (0.16) | 0.13 (0.18) | 4.27 (0.77) | 5.99 (1.38) | 4.10 (0.81) | 16, 32 | 4, 8 |
| 4 larvae, 6 days | 4.04 (0.66) | 6.40 (1.05) | 3.84 (0.70) | 0.63 (0.39) | 0.20 (0.48) | 4.01 (0.77) | 6.61 (1.38) | 3.81 (0.81) | 8, 12, 16, 24 | 2, 3, 4, 6 |
| 8 larvae, 3 days | 3.79 (0.92) | 6.19 (0.86) | 3.56 (0.88) | 0.61 (0.39) | 0.23 (0.64) | 3.89 (0.77) | 6.51 (1.38) | 3.66 (0.81) | 16, 32 | 2, 4 |
| 8 larvae, 6 days | 3.34 (0.72) | 7.96 (1.94) | 3.05 (0.71) | 0.42 (0.43) | 0.29 (0.51) | 3.63 (0.77) | 7.13 (1.38) | 3.37 (0.81) | 8, 12, 16, 24 | 1, 1.5, 2, 3 |
