# Supplementary material for: Competition and growth among Aedes aegypti larvae: Effects of distributing food inputs over time
Source: PLoS One. 2020 Oct 2;15(10):e0234676. doi: 10.1371/journal.pone.0234676 (PMC7531853; doi:10.1371/journal.pone.0234676)
Supplement: S46 Table — Means (SE) for Prime male mass and age at pupation and Average male mass at pupation for the interaction DxT. Expected mean values, growth rates, differences between Prime and Average male masses. (DOCX) [file pone.0234676.s087.docx]

S46 Table. Means (SE) for Prime male mass and age at pupation and Average male mass at pupation for the interaction DxT. Expected mean values, growth rates, differences between Prime and Average male masses.

| Density x Timespan | Prime male mass at pupation (mg) | Prime male age at pupation (days) | Average male mass at pupation (mg) | Estimated Prime male growth rate (mg/day) | Prime male mass MINUS Average male mass (mg) | Expected mean values of Prime male mass at pupation (mg) | Expected mean values of Prime male age at pupation (days) | Expected mean values of Average male mass at pupation (mg) |
| --- | --- | --- | --- | --- | --- | --- | --- | --- |
| 4 larvae, 3 days | 2.70 (0.08) | 5.10 (0.06) | 2.63 (0.05) | 0.53 (0.01) | 0.07 (0.01) | 2.58 (0.44) | 5.06 (0.24) | 2.49 (0.39) |
| 4 larvae, 6 days | 2.48 (0.37) | 5.00 (0.00) | 2.44 (0.41) | 0.50 (0.14) | 0.04 (0.31) | 2.40 (0.44) | 5.11 (0.24) | 2.36 (0.39) |
| 8 larvae, 3 days | 2.42 (0.36) | 5.05 (0.10) | 2.28 (0.33) | 0.48 (0.14) | 0.14 (0.24) | 2.37 (0.44) | 5.14 (0.24) | 2.28 (0.39) |
| 8 larvae, 6 days | 1.92 (0.53) | 5.35 (0.43) | 1.93 (0.37) | 0.36 (0.47) | -0.01 (0.42) | 2.19 (0.44) | 5.19 (0.24) | 2.14 (0.39) |
