# Supplementary material for: Competition and growth among Aedes aegypti larvae: Effects of distributing food inputs over time
Source: PLoS One. 2020 Oct 2;15(10):e0234676. doi: 10.1371/journal.pone.0234676 (PMC7531853; doi:10.1371/journal.pone.0234676)
Supplement: S47 Table — Means (SE) for Prime female mass at pupation for the interaction DxT. Differences between Prime and Average female masses, Prime and Average male masses, Prime female and Prime male masses, and Average female and male masses. Food/larva. (DOCX) [file pone.0234676.s088.docx]

S47 Table. Means (SE) for Prime female mass at pupation for the interaction DxT. Differences between Prime and Average female masses, Prime and Average male masses, Prime female and Prime male masses, and Average female and male masses. Food/larva.

| Density x Timespan | Prime female mass at pupation (mg) | Prime female mass MINUS Average female mass (mg) | Prime male mass MINUS Average male mass (mg) | Prime female mass MINUS Prime male mass (mg) | Average female mass MINUS Average male mass (mg) | Food/larva after day 4 (mg) |
| --- | --- | --- | --- | --- | --- | --- |
| 4 larvae, 3 days | 4.62 (0.24) | 0.13 (0.18) | 0.07 (0.01) | 1.92 (0.13) | 1.86 (0.14) | 4, 8 |
| 4 larvae, 6 days | 4.04 (0.66) | 0.20 (0.48) | 0.04 (0.31) | 1.56 (0.38) | 1.40 (0.41) | 2, 3, 4, 6 |
| 8 larvae, 3 days | 3.79 (0.92) | 0.23 (0.64) | 0.14 (0.24) | 1.37 (0.49) | 1.28 (0.47) | 2, 4 |
| 8 larvae, 6 days | 3.34 (0.72) | 0.29 (0.51) | -0.01 (0.42) | 1.42 (0.45) | 1.12 (0.40) | 1, 1.5, 2, 3 |
