# Supplementary material for: Competition and growth among Aedes aegypti larvae: Effects of distributing food inputs over time
Source: PLoS One. 2020 Oct 2;15(10):e0234676. doi: 10.1371/journal.pone.0234676 (PMC7531853; doi:10.1371/journal.pone.0234676)
Supplement: S48 Table — Survival of larvae across low densities showing sex ratio distortion at the lowest density. (DOCX) [file pone.0234676.s089.docx]

S48 Table. Survival of larvae across low densities showing sex ratio distortion at the lowest density.

| Larvae/vial | Total larvae | Number of Survivors | % Survival | Number of females | % Females (of Survivors) |
| --- | --- | --- | --- | --- | --- |
| 1 | 20 | 10 | 50 | 2 | 20 |
| 2 | 40 | 27 | 68 | 14 | 52 |
| 3 | 60 | 48 | 80 | 27 | 56 |
