# Supplementary material for: Competition and growth among Aedes aegypti larvae: Effects of distributing food inputs over time
Source: PLoS One. 2020 Oct 2;15(10):e0234676. doi: 10.1371/journal.pone.0234676 (PMC7531853; doi:10.1371/journal.pone.0234676)
Supplement: S49 Table — Mass at pupation and age at pupation for males and females at low densities. (DOCX) [file pone.0234676.s090.docx]

S49 Table. Mass at pupation and age at pupation for males and females at low densities.

| Larvae/vial | Number of males | Male mass at pupation mg (SD) | Male age at pupation days (SD) | Number of females | Female mass at pupation mg (SD) | Female age at pupation days (SD) |
| --- | --- | --- | --- | --- | --- | --- |
| 1 | 8 | 1.66 (0.51) | 7.00 (2.51) | 2 | 2.78 (0.66) | 6.00 (0.00) |
| 2 | 13 | 2.18 (0.38) | 5.38 (0.65) | 14 | 3.03 (0.88) | 6.57 (1.50) |
| 3 | 21 | 2.04 (0.31) | 5.50 (0.61) | 27 | 3.09 (0.70) | 7.26 (1.32) |
