# Supplementary material for: Competition and growth among Aedes aegypti larvae: Effects of distributing food inputs over time
Source: PLoS One. 2020 Oct 2;15(10):e0234676. doi: 10.1371/journal.pone.0234676 (PMC7531853; doi:10.1371/journal.pone.0234676)
Supplement: S50 Table — Means (SD) for mass and age at pupation by treatment factors. (DOCX) [file pone.0234676.s091.docx]

S50 Table. Means (SD) for mass and age at pupation by treatment factors.

| Second food input (mg) | Day of second food input | Sex | Number of observations | Mean mass (mg) | Mass (SD) | Mean age (days) | Age (SD) |
| --- | --- | --- | --- | --- | --- | --- | --- |
| 1 mg | day 6 | M | 10 | 1.652 | 0.276 | 9.500 | 1.080 |
| 1 mg | day 6 | F | 10 | 2.086 | 0.347 | 11.900 | 1.370 |
| 1 mg | day 8 | M | 6 | 1.682 | 0.271 | 12.167 | 0.408 |
| 1 mg | day 8 | F | 6 | 2.097 | 0.275 | 13.667 | 1.033 |
| 2 mg | day 6 | M | 10 | 1.947 | 0.438 | 9.200 | 1.135 |
| 2 mg | day 6 | F | 8 | 3.034 | 0.225 | 10.625 | 0.916 |
| 2 mg | day 8 | M | 9 | 2.272 | 0.124 | 11.889 | 0.782 |
| 2 mg | day 8 | F | 6 | 2.787 | 0.165 | 13.333 | 0.816 |
| 3 mg | day 6 | M | 10 | 2.265 | 0.268 | 9.300 | 0.823 |
| 3 mg | day 6 | F | 5 | 3.744 | 0.178 | 10.400 | 0.548 |
| 3 mg | day 8 | M | 5 | 2.262 | 0.259 | 12.200 | 1.095 |
| 3 mg | day 8 | F | 5 | 3.488 | 0.281 | 13.400 | 0.894 |
