# Supplementary material for: Competition and growth among Aedes aegypti larvae: Effects of distributing food inputs over time
Source: PLoS One. 2020 Oct 2;15(10):e0234676. doi: 10.1371/journal.pone.0234676 (PMC7531853; doi:10.1371/journal.pone.0234676)
Supplement: S51 Table — Counts and totals for larval deaths by treatment and time period. (DOCX) [file pone.0234676.s092.docx]

S51 Table. Counts and totals for larval deaths by treatment and time period.

| Second food input (mg) | Day of second food input | Number of dead larvae before second food input | Number of dead larvae after second food input | Number of dead larvae at end of experiment | Total number of dead larvae |
| --- | --- | --- | --- | --- | --- |
| 1 mg | day 6 | 1 | 4 | 0 | 5 |
| 1 mg | day 8 | 0 | 2 | 5 | 7 |
| 2 mg | day 6 | 1 | 8 | 1 | 10 |
| 2 mg | day 8 | 3 | 2 | 3 | 8 |
| 3 mg | day 6 | 3 | 0 | 5 | 8 |
| 3 mg | day 8 | 1 | 6 | 5 | 12 |
|  |  | 9 | 22 | 19 | 50 |
