# Supplementary material for: Competition and growth among Aedes aegypti larvae: Effects of distributing food inputs over time
Source: PLoS One. 2020 Oct 2;15(10):e0234676. doi: 10.1371/journal.pone.0234676 (PMC7531853; doi:10.1371/journal.pone.0234676)
Supplement: S52 Table — Mean (SD) age at death (days) of larvae by treatment. (DOCX) [file pone.0234676.s093.docx]

S52 Table. Mean (SD) age at death (days) of larvae by treatment.

| Second food input (mg) | Day of second food input | Number of dead larvae | Mean age at death | SD |
| --- | --- | --- | --- | --- |
| 1 mg | day 6 | 5 | 10.20 | 2.49 |
| 1 mg | day 8 | 2 | 11.00 | 2.83 |
| 2 mg | day 6 | 9 | 8.56 | 2.07 |
| 2 mg | day 8 | 5 | 8.60 | 3.71 |
| 3 mg | day 6 | 3 | 6.00 | 0.00 |
| 3 mg | day 8 | 7 | 11.17 | 2.14 |
|  |  |  |  |  |
