# Supplementary material for: Competition and growth among Aedes aegypti larvae: Effects of distributing food inputs over time
Source: PLoS One. 2020 Oct 2;15(10):e0234676. doi: 10.1371/journal.pone.0234676 (PMC7531853; doi:10.1371/journal.pone.0234676)
Supplement: S53 Table — Means (SE) for mass and age at pupation for main effects: food input amount, day of second food input, and sex. (DOCX) [file pone.0234676.s094.docx]

S53 Table. Experiment 3. Means (SE) for mass and age at pupation for main effects: food input amount, day of second food input, and sex.

| Main effects | Mean mass (SE) (mg) | Mean age (SE) (days) |
| --- | --- | --- |
| Second food input |  |  |
| 1 mg | 1.88 (0.25) | 4.81 (1.16) |
| 2 mg | 2.51 (0.49) | 4.26 (0.92) |
| 3 mg | 2.94 (0.79) | 4.33 (0.86) |
| Day of second food input |  |  |
| day 6 | 2.45 (0.78) | 4.15 (1.04) |
| day 8 | 2.43 (0.63) | 4.78 (0.77) |
| Sex |  |  |
| M | 2.01 (0.30) | 3.71 (0.44) |
| F | 2.87 (0.69) | 5.22 (0.59) |
