# Supplementary material for: Competition and growth among Aedes aegypti larvae: Effects of distributing food inputs over time
Source: PLoS One. 2020 Oct 2;15(10):e0234676. doi: 10.1371/journal.pone.0234676 (PMC7531853; doi:10.1371/journal.pone.0234676)
Supplement: S54 Table — MANOVA discriminant function coefficients, R squared values and P values for the single df contrasts. (DOCX) [file pone.0234676.s095.docx]

S54 Table. MANOVA discriminant function coefficients, R squared values and P values for the single df contrasts.

| Contrast | df | MANOVA Mass discriminant function coefficients | MANOVA Age discriminant function coefficients | MANOVA R squared | MANOVA P < |
| --- | --- | --- | --- | --- | --- |
| Food 1 | 1 | 0.991 | -0.388 | 0.56 | 0.001 |
| Food 2 | 1 | -0.159 | 1.015 | 0.37 | 0.001 |
| Delay day 6 vs day 8 | 1 | 0.956 | 0.173 | 0.70 | 0.001 |
| Sex M vs F | 1 | 0.845 | 0.409 | 0.28 | 0.001 |
| Food 1 x Delay | 1 | 0.819 | 0.450 | 0.34 | 0.001 |
| Food 1 x Sex | 1 | 0.311 | 0.898 | 0.17 | 0.001 |
| Food 2 x Delay | 1 | 1.012 | -0.099 | 0.08 | 0.039 |
| Food 2 x Sex | 1 | -0.350 | 0.999 | 0.17 | 0.001 |
| Delay x Sex | 1 |  |  |  | ns |
| Food 1 x Delay x Sex | 1 | 1.015 | -0.150 | 0.16 | 0.001 |
| Food 2 x Delay x Sex | 1 | 0.850 | 0.400 | 0.44 | 0.001 |
| Residual | 78 |  |  |  |  |
