# Supplementary material for: Competition and growth among Aedes aegypti larvae: Effects of distributing food inputs over time
Source: PLoS One. 2020 Oct 2;15(10):e0234676. doi: 10.1371/journal.pone.0234676 (PMC7531853; doi:10.1371/journal.pone.0234676)
Supplement: S55 Table — Mean squares (MS), r squared values and P values for the ANOVAs for the two variables mass and age at pupation for each single df contrast. (DOCX) [file pone.0234676.s096.docx]

S55 Table. Mean squares (MS), r squared values and P values for the ANOVAs for the two variables mass and age at pupation for each single df contrast.

| Contrast | df | ANOVA Mass MS | ANOVA Mass r squared | ANOVA Mass P < | ANOVA Age MS | ANOVA Age r squared | ANOVA Age P < |
| --- | --- | --- | --- | --- | --- | --- | --- |
| Food 1 | 1 | 6.70 | 0.18 | 0.001 | 4.45 | 0.02 | 0.034 |
| Food 2 | 1 | 0.00 |  | ns | 42.02 | 0.20 | 0.001 |
| Delay day 6 vs day 8 | 1 | 14.07 | 0.37 | 0.001 | 19.83 | 0.10 | 0.001 |
| Sex M vs F | 1 | 2.03 | 0.05 | 0.001 | 8.92 | 0.04 | 0.003 |
| Food 1 x Delay | 1 | 2.57 | 0.07 | 0.001 | 13.43 | 0.06 | 0.001 |
| Food 1 x Sex | 1 | 0.27 |  | ns | 13.61 | 0.07 | 0.001 |
| Food 2 x Delay | 1 | 0.54 | 0.01 | 0.011 | 0.04 |  | ns |
| Food 2 x Sex | 1 | 0.04 |  | ns | 13.65 | 0.07 | 0.001 |
| Delay x Sex | 1 | 0.00 |  | ns | 0.21 |  | ns |
| Food 1 x Delay x Sex | 1 | 1.18 | 0.03 | 0.001 | 0.01 |  | ns |
| Food 2 x Delay x Sex | 1 | 4.16 | 0.11 | 0.001 | 17.65 | 0.08 | 0.001 |
| Residual | 78 | 0.08 |  |  | 0.96 |  |  |
| Totals |  |  | 0.83 |  |  | 0.64 |  |
