# Supplementary material for: Competition and growth among Aedes aegypti larvae: Effects of distributing food inputs over time
Source: PLoS One. 2020 Oct 2;15(10):e0234676. doi: 10.1371/journal.pone.0234676 (PMC7531853; doi:10.1371/journal.pone.0234676)
Supplement: S56 Table — Means (SE) for mass (mg) for the interaction food 2 x delay x sex. (DOCX) [file pone.0234676.s097.docx]

S56 Table. Means (SE) for mass (mg) for the interaction food 2 x delay x sex.

| Second food input (Food 2) | Delay (day 6 or day 8) | Mass (SE) of males (mg) | Mass (SE) of females (mg) |
| --- | --- | --- | --- |
| 1 mg + 2 mg | day 6 | 1.80 (0.21) | 2.56 (0.67) |
|  | day 8 | 1.98 (0.42) | 2.44 (0.49) |
| 3 mg | day 6 | 2.27 (0.27) | 3.74 (0.18) |
|  | day 8 | 2.26 (0.26) | 3.49 (0.28) |
