# Supplementary material for: Competition and growth among Aedes aegypti larvae: Effects of distributing food inputs over time
Source: PLoS One. 2020 Oct 2;15(10):e0234676. doi: 10.1371/journal.pone.0234676 (PMC7531853; doi:10.1371/journal.pone.0234676)
Supplement: S57 Table — Means (SE) for age (days) for the interaction food 2 x delay x sex. (DOCX) [file pone.0234676.s098.docx]

S57 Table. Means (SE) for age (days) for the interaction food 2 x delay x sex.

| Second food input (Food 2) | Delay (day 6 or day 8) | Age (SE) of males (days) | Age (SE) of females (days) |
| --- | --- | --- | --- |
| 1 mg + 2 mg | day 6 | 3.35 (0.21) | 5.26 (0.90) |
|  | day 8 | 4.03 (0.20) | 5.50 (0.24) |
| 3 mg | day 6 | 3.30 (0.82) | 4.40 (0.55) |
|  | day 8 | 4.20 (1.10) | 5.40 (0.89) |
