# Supplementary material for: Competition and growth among Aedes aegypti larvae: Effects of distributing food inputs over time
Source: PLoS One. 2020 Oct 2;15(10):e0234676. doi: 10.1371/journal.pone.0234676 (PMC7531853; doi:10.1371/journal.pone.0234676)
Supplement: S58 Table — Means (SE) for estimated growth rates (mg/day) for the interaction food 2 x delay x sex. (DOCX) [file pone.0234676.s099.docx]

S58 Table. Means (SE) for estimated growth rates (mg/day) for the interaction food 2 x delay x sex.

| Second food input (Food 2) | Delay (day 6 or day 8) | Estimated growth rate (SE) of males (mg/day) | Estimated growth rate (SE) of females (mg/day) |
| --- | --- | --- | --- |
| 1 mg + 2 mg | day 6 | 0.54 (0.07) | 0.49 (0.15) |
|  | day 8 | 0.49 (0.11) | 0.44 (0.09) |
| 3 mg | day 6 | 0.69 (0.19) | 0.85 (0.11) |
|  | day 8 | 0.54 (0.15) | 0.65 (0.12) |
