# Supplementary material for: Competition and growth among Aedes aegypti larvae: Effects of distributing food inputs over time
Source: PLoS One. 2020 Oct 2;15(10):e0234676. doi: 10.1371/journal.pone.0234676 (PMC7531853; doi:10.1371/journal.pone.0234676)
Supplement: S59 Table — Means (SE) for mass (mg) for the interaction food 1 x delay x sex. (DOCX) [file pone.0234676.s100.docx]

S59 Table. Means (SE) for mass (mg) for the interaction food 1 x delay x sex.

| Second food input (Food 1) | Delay (day 6 or day 8) | Mass (SE) of males (mg) | Mass (SE) of females (mg) |
| --- | --- | --- | --- |
| 1 mg | day 6 | 1.65 (0.28) | 2.09 (0.35) |
|  | day 8 | 1.68 (0.27) | 2.10 (0.28) |
| 2 mg | day 6 | 1.95 (0.44) | 3.03 (0.23) |
|  | day 8 | 2.27 (0.12) | 2.79 (0.17) |
