# Supplementary material for: Competition and growth among Aedes aegypti larvae: Effects of distributing food inputs over time
Source: PLoS One. 2020 Oct 2;15(10):e0234676. doi: 10.1371/journal.pone.0234676 (PMC7531853; doi:10.1371/journal.pone.0234676)
Supplement: S60 Table — Means (SE) for age (days) for the interaction food 1 x delay x sex. (DOCX) [file pone.0234676.s101.docx]

S60 Table. Means (SE) for age (days) for the interaction food 1 x delay x sex.

| Second food input (Food 1) | Delay (day 6 or day 8) | Age (SE) of males (days) | Age (SE) of females (days) |
| --- | --- | --- | --- |
| 1 mg | day 6 | 3.50 (1.08) | 5.90 (1.37) |
|  | day 8 | 4.17 (0.41) | 5.67 (1.03) |
| 2 mg | day 6 | 3.20 (1.14) | 4.63 (0.92) |
|  | day 8 | 3.89 (0.78) | 5.33 (0.82) |
