# Supplementary material for: Competition and growth among Aedes aegypti larvae: Effects of distributing food inputs over time
Source: PLoS One. 2020 Oct 2;15(10):e0234676. doi: 10.1371/journal.pone.0234676 (PMC7531853; doi:10.1371/journal.pone.0234676)
Supplement: S61 Table — Means (SE) for estimated growth rates (mg/day) for the interaction food 1 x delay x sex. (DOCX) [file pone.0234676.s102.docx]

S61 Table. Means (SE) for estimated growth rates (mg/day) for the interaction food 1 x delay x sex.

| Second food input (Food 1) | Delay (day 6 or day 8) | Estimated growth rate (SE) of males (mg/day) | Estimated growth rate (SE) of females (mg/day) |
| --- | --- | --- | --- |
| 1 mg | day 6 | 0.47 (0.17) | 0.35 (0.10) |
|  | day 8 | 0.40 (0.08) | 0.37 (0.08) |
| 2 mg | day 6 | 0.61 (0.26) | 0.66 (0.14) |
|  | day 8 | 0.58 (0.12) | 0.52 (0.09) |
