# Supplementary material for: Competition and growth among Aedes aegypti larvae: Effects of distributing food inputs over time
Source: PLoS One. 2020 Oct 2;15(10):e0234676. doi: 10.1371/journal.pone.0234676 (PMC7531853; doi:10.1371/journal.pone.0234676)
Supplement: S62 Table — Means (SE), expected values and differences for mass (mg) for the interaction food 1 x delay. (DOCX) [file pone.0234676.s103.docx]

S62 Table. Means (SE), expected values and differences for mass (mg) for the interaction food 1 x delay.

| Second food input | Delay | Mass (SE) (mg) | Expected value of mass (SE) (mg) | Difference between observed and expected values (SE) (mg) |
| --- | --- | --- | --- | --- |
| 1 mg | day 6 | 1.87 (0.31) | 2.15 (0.68) | -0.28 (0.37) |
|  | day 8 | 1.89 (0.29) | 2.14 (0.68) | -0.25 (0.37) |
| 2 mg | day 6 | 2.49 (0.77) | 2.48 (0.68) | 0.01 (0.51) |
|  | day 8 | 2.53 (0.36) | 2.47 (0.68) | 0.06 (0.39) |
