# Supplementary material for: Competition and growth among Aedes aegypti larvae: Effects of distributing food inputs over time
Source: PLoS One. 2020 Oct 2;15(10):e0234676. doi: 10.1371/journal.pone.0234676 (PMC7531853; doi:10.1371/journal.pone.0234676)
Supplement: S63 Table — Means (SE), expected values and differences for age (days) for the interaction food 1 x delay. (DOCX) [file pone.0234676.s104.docx]

S63 Table. Means (SE), expected values and differences for age (days) for the interaction food 1 x delay.

| Second food input | Delay | Age (SE) (days) | Expected value of age (SE) (days) | Difference between observed and expected values (SE) (mg) |
| --- | --- | --- | --- | --- |
| 1 mg | day 6 | 4.70 (1.70) | 4.47 (0.93) | 0.23 (0.97) |
|  | day 8 | 4.92 (1.06) | 4.79 (0.93) | 0.13 (0.71) |
| 2 mg | day 6 | 3.91 (1.01) | 4.21 (0.93) | -0.30 (0.69) |
|  | day 8 | 4.61 (1.02) | 4.51 (0.93) | 0.10 (0.69) |
