# Supplementary material for: Competition and growth among Aedes aegypti larvae: Effects of distributing food inputs over time
Source: PLoS One. 2020 Oct 2;15(10):e0234676. doi: 10.1371/journal.pone.0234676 (PMC7531853; doi:10.1371/journal.pone.0234676)
Supplement: S64 Table — Means (SE), expected values and differences for mass (mg) for the interaction food 2 x delay. (DOCX) [file pone.0234676.s105.docx]

S64 Table. Means (SE), expected values and differences for mass (mg) for the interaction food 2 x delay.

| Second food input | Delay | Mass (SE) (mg) | Expected value of mass (SE) (mg) | Difference between observed and expected values (SE) (mg) |
| --- | --- | --- | --- | --- |
| 1 mg + 2 mg | day 6 | 2.18 (0.60) | 2.32 (0.68) | -0.14 (0.45) |
|  | day 8 | 2.21 (0.46) | 2.31 (0.68) | -0.10 (0.41) |
| 3 mg | day 6 | 3.00 (1.05) | 2.69 (0.68) | 0.32 (0.62) |
|  | day 8 | 2.88 (0.87) | 2.67 (0.68) | 0.20 (0.55) |
