# Supplementary material for: Competition and growth among Aedes aegypti larvae: Effects of distributing food inputs over time
Source: PLoS One. 2020 Oct 2;15(10):e0234676. doi: 10.1371/journal.pone.0234676 (PMC7531853; doi:10.1371/journal.pone.0234676)
Supplement: S65 Table — Means (SE) for age (days) for the interaction food 2 x delay (not significant in the ANOVA). (DOCX) [file pone.0234676.s106.docx]

S65 Table. Means (SE) for age (days) for the interaction food 2 x delay (not significant in the ANOVA).

| Second food input | Delay | Age (SE) (days) |
| --- | --- | --- |
| 1 mg + 2 mg | day 6 | 4.31 (1.23) |
|  | day 8 | 4.76 (0.87) |
| 3 mg | day 6 | 3.85 (0.78) |
|  | day 8 | 4.80 (0.85) |
