# Supplementary material for: Competition and growth among Aedes aegypti larvae: Effects of distributing food inputs over time
Source: PLoS One. 2020 Oct 2;15(10):e0234676. doi: 10.1371/journal.pone.0234676 (PMC7531853; doi:10.1371/journal.pone.0234676)
Supplement: S66 Table — Means (SE), expected values and differences for age (days) for the interaction food 1 x sex. (DOCX) [file pone.0234676.s107.docx]

S66 Table. Means (SE), expected values and differences for age (days) for the interaction food 1 x sex.

| Second food input | Sex | Age (SE) (days) | Expected value of age (SE) (days) | Difference between observed and expected values (SE) (days) |
| --- | --- | --- | --- | --- |
| 1 mg | M | 3.83 (0.47) | 4.22 (0.93) | -0.39 (0.52) |
|  | F | 5.78 (0.16) | 5.01 (0.93) | 0.77 (0.47) |
| 2 mg | M | 3.54 (0.49) | 3.98 (0.93) | -0.44 (0.53) |
|  | F | 4.98 (0.50) | 4.72 (0.93) | 0.26 (0.53) |
