# Supplementary material for: Competition and growth among Aedes aegypti larvae: Effects of distributing food inputs over time
Source: PLoS One. 2020 Oct 2;15(10):e0234676. doi: 10.1371/journal.pone.0234676 (PMC7531853; doi:10.1371/journal.pone.0234676)
Supplement: S68 Table — Means (SE) for estimated growth rates (mg/day) for the interaction food 1 x sex. (DOCX) [file pone.0234676.s109.docx]

S68 Table. Means (SE) for estimated growth rates (mg/day) for the interaction food 1 x sex.

| Second food input | Sex | Estimated growth rate (mg/day) |
| --- | --- | --- |
| 1 mg | M | 0.43 (0.05) |
|  | F | 0.36 (0.01) |
| 2 mg | M | 0.60 (0.10) |
|  | F | 0.58 (0.07) |
