# Supplementary material for: Competition and growth among Aedes aegypti larvae: Effects of distributing food inputs over time
Source: PLoS One. 2020 Oct 2;15(10):e0234676. doi: 10.1371/journal.pone.0234676 (PMC7531853; doi:10.1371/journal.pone.0234676)
Supplement: S69 Table — Means (SE), expected values and differences for age (days) for the interaction food 2 x sex. (DOCX) [file pone.0234676.s110.docx]

S69 Table. Means (SE), expected values and differences for age (days) for the interaction food 2 x sex.

| Second food input | Sex | Age (SE) (days) | Expected value of age (SE) (days) | Difference between observed and expected values (SE) (days) |
| --- | --- | --- | --- | --- |
| 1 mg + 2 mg | M | 3.69 (0.43) | 4.10 (0.93) | -0.41 (0.51) |
|  | F | 5.38 (0.56) | 4.87 (0.93) | 0.51 (0.54) |
| 3 mg | M | 3.75 (0.64) | 4.01 (0.93) | -0.26 (0.56) |
|  | F | 4.90 (0.71) | 4.75 (0.93) | 0.15 (0.59) |
