# Supplementary material for: Competition and growth among Aedes aegypti larvae: Effects of distributing food inputs over time
Source: PLoS One. 2020 Oct 2;15(10):e0234676. doi: 10.1371/journal.pone.0234676 (PMC7531853; doi:10.1371/journal.pone.0234676)
Supplement: S70 Table — Means (SE) for mass (mg) for the interaction food 2 x sex (not significant in the ANOVA). (DOCX) [file pone.0234676.s111.docx]

S70 Table. Means (SE) for mass (mg) for the interaction food 2 x sex (not significant in the ANOVA).

| Second food input | Sex | Mass (SE) (mg) |
| --- | --- | --- |
| 1 mg + 2 mg | M | 1.89 (0.29) |
|  | F | 2.50 (0.48) |
| 3 mg | M | 2.26 (0.00) |
|  | F | 3.62 (0.18) |
