# Supplementary material for: Competition and growth among Aedes aegypti larvae: Effects of distributing food inputs over time
Source: PLoS One. 2020 Oct 2;15(10):e0234676. doi: 10.1371/journal.pone.0234676 (PMC7531853; doi:10.1371/journal.pone.0234676)
Supplement: S1 Text — (DOCX) [file pone.0234676.s113.docx]

**S1 Text. Detailed methods**

**First experiment**

Eggs were obtained from fifth generation female *A. aegypti* in a colony derived from collections in tires near the Dade County Public Works Department (Florida). Eggs were hatched in distilled water and larvae were counted into test tubes (new, disposable, round bottomed, approximately 25 x 150 mm) containing 20 ml distilled water. Two density treatments, 4 or 8 larvae/test tube, were crossed with two total food levels treatments, 16 or 32 mg baker’s yeast/test tube. Two further factors were added to investigate the distribution of food input over time. For the aliquot factor, the total food was divided into 2 or 4 aliquots. The first aliquot was always presented on the day of hatching (day 0). The subsequent aliquots were distributed across a timespan of either 3 or 6 days. Each additional input was suspended in 0.5 ml distilled water. The amount and distribution of aliquots across the 16 treatments of this 4-factor experiment are listed in S1 Table. Ten biological replicates of each treatment combination were initiated. 960 1st instar larvae were counted into 160 test tubes according to the 16 treatments. Each test tube was then provided with the appropriate amount of suspended yeast cells for that treatment. The test tubes were stored in randomized sequence and kept at ambient conditions throughout the experiment. Additional food was provided according to treatment on the appropriate days. The test tubes were examined for pupae from day 4 until day 35 when the last larva died. Pupae were removed, identified to sex, and weighed to the nearest 0.01 mg. On day 6, pupae were removed before the final aliquots of food were added. [The detailed, step-by-step methodology is presented in dx.doi.org/10.17504/protocols.io.bddhi236].

This is a 2x2x2x2 experimental design. The experiment crosses two additional factors, aliquot and timespan, with the four competition treatments to look at how the rate and timing of food inputs affect larval competition among females, among males and between the sexes. There are four main effects, six 2-way interactions, four 3-way interactions and one 4-way interaction. Some of the interactions describe competition, others describe growth, and others could describe competition or growth. Results from the prior experiment [1] suggest the outcome of competitive interactions. Females dominate males in larval competition. Females should be largest and pupate earliest in the treatments with the least competition and smallest and pupate latest in the treatments with the most competition. The two intermediate competition treatments offer the same food per larva at different densities (so the total food will be higher at the higher density). Females in the intermediate competition treatments should be larger at the higher density than at the lower density because of the higher total food despite the same food per larva in both treatments. Males respond differently to the competition treatments and are also affected by competition with females. Males and females may extend their larval growth in response to competition and to take advantage of food abundance.

In addition to the interactions involving competition (food and density), there are also interactions involving only aspects of the food supply (food, aliquot and timespan), and interactions between density, aliquot and timespan. The interactions involving only food, aliquot and timespan describe the effects of these attributes of the food supply on growth, not competition. The interactions involving density, aliquot and timespan may describe competitive effects that are independent of the total food level, and due only to the distribution of the food inputs in time. Alternatively, these interactions may describe the effects of these factors on growth, not competition.

At the conclusion of the experiment, 7 dependent variables were calculated for each replicate: Survival, Prime male mass and age at pupation, Average male mass, Prime female mass and age at pupation, and Average female mass. The Prime male was the male with the greatest growth rate; this is the first male to pupate, or the largest of the first males to pupate. The Prime female was the largest female to pupate. The distinction between the two Prime individuals reflects differences in the growth and pupation of the two sexes [1] [34-36]. Test tubes with survivors of only one sex were excluded from analysis. The data were analyzed as a multivariate, four-way analysis of variance (MANOVA) [37,38].

**Second experiment**

Eggs were obtained and hatched as previously described, to produce 120 1^st^ instar larvae. 60 flat-bottomed shell vials (25 mm diameter x 95 mm tall) were numbered and filled with 20 ml distilled water containing a concentration of baker’s yeast to produce the food level treatments (2 mg, 3 mg, 4 mg, and 5 mg dry weight of yeast per larva). 1, 2 or 3 larvae were added to each vial according to randomized treatment. 5 replicates of each treatment were initiated. S2 Table lists the treatments and replicates. The test tubes were examined for pupae from day 4 until the last larva died. Pupae were removed, identified to sex, and weighed to the nearest 0.01 mg. [The detailed, step-by-step methodology is presented in dx.doi.org/10.17504/protocols.io.bddhi236].

**Third experiment**

Eggs were obtained and hatched as previously described, to produce 150 1^st^ instar larvae. 150 test tubes (new, disposable, round bottomed, approximately 25 x 150 mm) were numbered, then assigned to a treatment using a random number table. There were 6 treatments with 25 physical replicates each. S3 Table lists the treatments with the incremental food and delay. [The detailed, step-by-step methodology is presented in dx.doi.org/10.17504/protocols.io.bddhi236].

Each test tube received 20 ml distilled water with 1 mg Baker’s yeast and a single 1^st^ instar larva on day 0. Test tubes were examined for pupa beginning on day 4, the day before any pupa were expected. On day 6, after examining for dead larvae and pupae, treatments 1-3 received 1 mg, 2 mg, or 3 mg additional yeast (dry weight) respectively. On day 7, test tubes were examined for dead larvae and pupae. On day 8, after examining for dead larvae and pupae, treatments 4-6 received 1 mg, 2 mg, or 3 mg additional yeast (dry weight) respectively. On subsequent days, test tubes were examined for dead larvae and pupae. Each pupa was blotted on paper toweling to remove excess water and weighed to the nearest 0.01 mg. The weight (mg), sex (M/F), and age at pupation (days) were recorded for each tube with a pupa. Larvae that died before pupating are also recorded by day at which death is observed.

The mosquito larvae are not expected to be able to pupate on the initial amount of food (1 mg), but they should be able to survive until the second food input. The amount of food at the second food input (1 mg, 2 mg or 3 mg) should allow pupation and the size and age at pupation should improve with increasing amounts. The shorter delay should be better for both size and age than the longer delay. Better outcomes mean larger masses and earlier ages at pupation. Males and females may delay pupation in response to abundant food, so there are possible interactions between the mass and age variables, which should show up in the MANOVA. There is no competition in this experiment because larvae are alone in their test tubes, so sex is treated as a factor rather than as covarying dependent variables.

The data are analyzed as a multivariate, three-way analysis of variance (MANOVA) [37,38] with 3 factors: amount of incremental food (1 mg, 2 mg, or 3 mg); delay (6 or 8 days between the start of the experiment and the input of the incremental food); and sex (male or female). The two dependent variables are: mass at pupation (mg) and days to pupation after the second input of food. The age at pupation is calculated as: day of pupation MINUS the delay, 6 days or 8 days, depending on the treatment. The transformation of the age by subtracting the delay removes the numerical effect of the delay from the biological effect of the delay.

**Note on the mosquito colony maintenance**

The colony was maintained in an insectary with access to sugar water, and fed on a mouse or a chick approximately once a week over several months. There was always an oviposition site available and the filter papers were changed once or twice a week. These filter papers with eggs attached were stored in an insectary until it was time to regenerate the colony or to conduct an experiment. Filter papers with the appropriate number of eggs were immersed in distilled water to hatch the eggs. The larvae were counted into the experimental treatments and the extra larvae were raised in large enamel trays. Pupae from the batch trays and from the experiment were released into a new colony cage. The procedure was to make sure that there were enough eggs for each experiment, but it effectively resulted in synchronizing the colony.
