# Supplementary material for: Competition and growth among Aedes aegypti larvae: Effects of distributing food inputs over time
Source: PLoS One. 2020 Oct 2;15(10):e0234676. doi: 10.1371/journal.pone.0234676 (PMC7531853; doi:10.1371/journal.pone.0234676)
Supplement: S3 Text — First experiment results analysis by interaction. (DOCX) [file pone.0234676.s115.docx]

**S3 Text. First experiment results analysis by interaction**

**The FxDxT interaction (R squared = 0.87)**

The interaction between food and density is expected to produce 4 different competitive environments: least competition (high food, low density); most competition (low food, high density) and two intermediate levels of competition (high food, high density, and low food, low density). The least competition test tubes receive 32 mg of food or 8 mg food/larva. The most competition test tubes receive only 16 mg of food or 2 mg food/larva. The two intermediate competition treatments receive 32 mg (for 8 larvae) or 16 mg (for 4 larvae) of food resulting in 4 mg food/larva. Mosquito larvae should grow larger, faster, and pupate earlier in the least competition treatment, followed by the intermediate competition treatments. Pupae should be smallest, grow slowest and pupate latest in the most competition treatment. The high food, high density treatment is expected to be better for mosquito larval growth than the low food, low density treatment because the total food is greater. These four treatments are crossed with the two timespan treatments (3 days or 6 days) for a total of 8 treatment combinations. The 3 day timespan is expected to be better than the 6 day timespan because more food is offered earlier in the larval period. The interaction between the FxD treatments and the timespan treatment reveals how timespan affects the competition for food among the larvae.

This interaction is significant for all seven dependent variables: Survival, Prime male mass and age at pupation, Average male mass, Prime female mass and age at pupation, and Average female mass. It is the most significant interaction in the MANOVA and for the mass variables in the ANOVAs, and one of two interactions that are significant in the ANOVAs for all seven dependent variables. The r squared values from the seven ANOVAs are: 0.05, 0.10, 0.16, 0.16, 0.16, 0.45, and 0.16, respectively (S9 Table). The MANOVA discriminant function coefficients correspond roughly to the r squared values with the exception of Prime female mass, which is close to zero compared to its r squared value of 0.16. Prime male mass and Prime female mass have negative discriminant function coefficients indicating that higher values of the two Prime masses are associated with lower values of Prime ages, Average masses, and Survival. Larger Prime individuals pupate earlier and are more dominant over the non-Prime individuals of both sexes. Large Prime individuals occur in test tubes that have lower survival.

S20 Table shows the means and standard errors for the food x density x timespan interaction for Survival. A heuristic 3D plot of the means is presented in S5 Fig.

1. This contrast explains 5% of the variability in Survival across the experiment.
2. Survival is affected by competition; the Survival is similar across the six least competition and intermediate competition treatments, and lower in both most competition treatments.
3. Survival is better with the 6 day timespan than with the 3 day timespan except in the test tubes with the most competition.
4. Survival appears to improve with the gradual addition of food (6 days versus 3 days) except at the lowest food level where the 6 day timespan may increase the food stress and competitive interactions.

S20 Table. Means (SE) for arcsin transformed percent Survival for the interaction FxDxT

| Food x Density | Timespan | Survival |
| --- | --- | --- |
| Low food, low density (4 mg/larva) | 3 days | 1.31 (0.14) |
|  | 6 days | 1.36 (0.11) |
| Most competition (2 mg/larva) | 3 days | 1.08 (0.06) |
|  | 6 days | 1.00 (0.05) |
| Least competition (8 mg/larva) | 3 days | 1.15 (0.02) |
|  | 6 days | 1.37 (0.16) |
| High food, high density (4 mg/larva) | 3 days | 1.13 (0.28) |
|  | 6 days | 1.28 (0.11) |

**Prime female mass**

S21 Table shows the means and standard errors for the food x density x timespan interaction for the Prime female mass at pupation, the Prime female age at pupation and the Average female mass at pupation. The treatments are labelled a-h, ranked from largest to smallest by Prime female mass. Heuristic 3D plots of the means for Prime female mass, Prime female age, estimated growth rates, Average female mass and the difference between the Prime female mass and the Average female mass are presented in S6 Fig-S10 Fig. S22 Table shows the same means and standard errors for Prime female mass and age, and Average female mass, with the same ranking, alongside two additional columns: total food after day 4, and food/larva after day 4. Aliquot is not a factor in this interaction, but it does affect the amount of food after day 4; the 4 aliquot treatment receives more food by day 4 than the 2 aliquot treatment.

1. The r squared values for these three variables (Prime female mass and age, Average female mass) for this contrast are 0.16, 0.05, and 0.16, out of a total r squared of 0.88, 0.79, and 0.90, which explain 16%, 5%, and 16% of the variability across the experiment, respectively.
2. The Prime female mass is affected by competition; the masses are greater in the least competition treatments and the two intermediate competition treatments than in the most competition treatments.
3. Across the food x density treatments that create different levels of competition, the Prime female mass is always greater with the 3 day timespan compared to the 6 day timespan in each competitive environment.
4. The largest mass is in the least competition, 3 day timespan treatment combination and the smallest mass is in the most competition, 6 day timespan.
5. Timespan has a small effect on Prime females in the most competition treatments (0.22 mg difference between the Prime females in the 3 day and 6 day timespan test tubes, g and h).
6. It has a slightly larger effect on the those in the least competition test tubes (0.31 mg difference between the Prime females in the 3 day and 6 day timespan test tubes, a and c).
7. It has a much larger effect on the Prime females in the two intermediate competition treatments (0.67 mg and 0.84 mg; b and e, d and f). This is a likely cause for the interaction FxDxT for the Prime female mass.
8. Within the 2 mg span of size for Prime females across these 8 treatment combinations, there are four at the high end (4.42 mg to 4.82 mg; a-d), two in the middle (3.58 mg and 3.91 mg; e and f) and two at the low end (2.78 mg and 3.00 mg; g and h); these correspond to the least competition treatment plus the two intermediate competition treatments at the 3 day timespan (a-d), then the two intermediate treatments at the 6 day timespan (e and f), and finally the two most competition treatments (g and h).
9. The four treatments with the largest Prime females all have at least 4 mg food/larva by day 3 or day 4. The two treatments in the middle have 2 mg food/larva or 3 mg food/larva by day 3 or 4, and the two smallest Prime females are in test tubes with only 1 mg to 2 mg food/larva by day 3 or 4. However, for the four treatments with the largest Prime females (a-d), the total food on day 4 better explains the mass at pupation than does the food/larva. Prime females in a and b have 32 mg total food, c has either 16 or 24 mg, and d has 16 mg. For the four treatments with the smallest Prime females, the food/larva on day 4 explains the masses better, with the total food affecting the order when the food/larva is equal: e—2 mg or 3 mg food/larva, 16 mg or 24 mg total food; f—2 mg or 3 mg food/larva, 8 mg or 12 mg total food; g—2 mg food/larva, 16 mg total food; and h—1 mg or 1.5 mg food/larva, 8 mg or 12 mg total food. The difference in the relative importance of food/larva and total food may also contribute to the FxDxT interaction for the Prime female mass.
10. The number of aliquots is not a factor in this interaction so the difference between the delivery by day 3 or 4 is not important here. The reason for calculating the amount of food in the test tubes by day 3 or 4 is that most of the Prime females pupated before the addition of the final aliquot of food on day 6 (in the 6 day timespan treatment). Total food per test tube and food/larva (by day 4) jointly explain the Prime female mass in this interaction. The effect of FxDxT is to reduce the food/larva and the total amount of food in the day 6 timespan treatments for Prime females.

S21 Table. Means (SE) for Prime female mass and age at pupation and Average female mass at pupation for the interaction FxDxT

| Food x Density | Timespan | Rank by Prime female mass (a-h) | Prime female mass at pupation (mg) | Prime female age at pupation (days) | Average female mass at pupation (mg) | Estimated Prime female growth rate (mg/day) | Prime female mass MINUS Average female mass (mg) |
| --- | --- | --- | --- | --- | --- | --- | --- |
| Low food, low density (4 mg/larva) | 3 days | d | 4.42 (0.03) | 5.89 (0.01) | 4.25 (0.04) | 0.75 (0.01) | 0.17 (0.05) |
|  | 6 days | f | 3.58 (0.43) | 7.20 (0.85) | 3.33 (0.51) | 0.50 (0.19) | 0.25 (0.67) |
| Most competition (2 mg/larva) | 3 days | g | 3.00 (0.06) | 6.87 (0.61) | 2.80 (0.04) | 0.44 (0.10) | 0.20 (0.07) |
|  | 6 days | h | 2.78 (0.04) | 9.36 (1.76) | 2.57 (0.11) | 0.30 (0.17) | 0.21 (0.12) |
| Least competition (8 mg/larva) | 3 days | a | 4.82 (0.10) | 5.47 (0.35) | 4.74 (0.04) | 0.88 (0.14) | 0.08 (0.11) |
|  | 6 days | c | 4.51 (0.49) | 5.61 (0.15) | 4.35 (0.45) | 0.80 (0.19) | 0.16 (0.67) |
| High food, high density (4 mg/larva) | 3 days | b | 4.58 (0.16) | 5.52 (0.16) | 4.31 (0.16) | 0.83 (0.08) | 0.27 (0.23) |
|  | 6 days | e | 3.91 (0.52) | 6.57 (0.62) | 3.53 (0.75) | 0.60 (0.21) | 0.38 (0.91) |

S22 Table. Means (SE) for Prime female mass and age at pupation and Average female mass at pupation for the interaction FxDxT with total food after day 4 and food/larva after day 4.

| Food x Density | Timespan | Rank by Prime female mass (a-h) | Prime female mass at pupation (mg) | Prime female age at pupation (days) | Average female mass at pupation (mg) | Total food after day 4 (mg) | Food/larva after day 4 (mg) |
| --- | --- | --- | --- | --- | --- | --- | --- |
| Low food, low density (4 mg/larva) | 3 days | d | 4.42 (0.03) | 5.89 (0.01) | 4.25 (0.04) | 16, 16 | 4, 4 |
|  | 6 days | f | 3.58 (0.43) | 7.20 (0.85) | 3.33 (0.51) | 8, 12 | 2, 3 |
| Most competition (2 mg/larva) | 3 days | g | 3.00 (0.06) | 6.87 (0.61) | 2.80 (0.04) | 16, 16 | 2, 2 |
|  | 6 days | h | 2.78 (0.04) | 9.36 (1.76) | 2.57 (0.11) | 8, 12 | 1, 1.5 |
| Least competition (8 mg/larva) | 3 days | a | 4.82 (0.10) | 5.47 (0.35) | 4.74 (0.04) | 32, 32 | 8, 8 |
|  | 6 days | c | 4.51 (0.49) | 5.61 (0.15) | 4.35 (0.45) | 16, 24 | 4, 6 |
| High food, high density (4 mg/larva) | 3 days | b | 4.58 (0.16) | 5.52 (0.16) | 4.31 (0.16) | 32, 32 | 4, 4 |
|  | 6 days | e | 3.91 (0.52) | 6.57 (0.62) | 3.53 (0.75) | 16, 24 | 4, 6 |

**Prime female age**

1. Prime female age at pupation is also affected by the level of competition and by timespan, but the effect of timespan is much greater relative to the effect of competition as compared to Prime female mass. With the 3 day timespan, the Prime females pupate earliest in the least competition treatment, a, followed by the high food, high density, b, then the low food, low density, d, and finally the most competition treatment, g. The 6 day timespan shows the same order (c, e, f, then h), but pupation is delayed relative to the 3 day treatments.
2. The earliest Prime females to pupate are in the least competition, 3 day timespan, a, followed by the high food, high density, 3 day timespan, b, then the least competition, 6 day timespan, c, and the low food, low density, 3 day timespan, d. These correspond to the four treatments with the largest Prime females (in the same order). Almost all these Prime females pupate by day 6 (before the final aliquot is added in the 6 day timespan treatment, e.g. for the Prime females in c). These treatments have 4 mg food/larva by day 4 (see #13 and #14 above). At high relative food/larva levels and greater total food levels, mass at pupation and age at pupation are inversely related; Prime females are larger in mass and pupate earlier with more food, delivered earlier in the larval period. Total food seems to be more important that food/larva for these four treatments (see #13).
3. The four treatments associated with the lower pupal masses do not pupate in the same order as their relative weights. The Prime females in the high food, high density, 6 day timespan test tubes pupate, e, followed by those in the most competition, 3 day timespan test tubes, g, then the low food, low density, 6 day timespan test tubes, f, and finally the most competition, 6 day timespan, h. This is likely one source of the FxDxT interaction for Prime female age at pupation. In contrast to the relative effects of total food and food/larva on the masses of Prime females (e-h), the ages at pupation for these Prime females appear to be affected more by the total food on day 4 than by the food/larva on day 4: e—16 mg or 24 mg total food, 2 mg or 3 mg food/larva; g—16 mg total food, 2 mg food/larva; f—8 mg or 12 mg total food; 2 mg or 3 mg food/larva; and h—8 mg or 12 mg total food, 1 mg or 1.5 mg food/larva.
4. About half the Prime females in the high food, high density, 6 day timespan test tubes, e, pupate on or before day 6 (before the final aliquot of food is added); the remainder pupate on day 7 or later, after the addition of food. They delay pupation, perhaps in response to the additional food, but do not grow as large as in any of the treatment combinations that pupate earlier.
5. The next in pupation sequence, the treatment combination with most competition and the 3 day timespan, g, does not receive an aliquot of food on day 6; these Prime females pupate almost a day later (6.87 days) and 1.42 mg smaller than any other 3 day timespan treatment. Greater competition for food in g delays Prime female pupation more than in two of the 6 day timespan treatments (c and e). The Prime females in g have 16 mg total food and 2 mg food/larva after day 3. The Prime females in c pupate earlier and larger on 16 mg or 24 mg total food and 4 mg or 6 mg food/larva (before the final food input on day 6). The Prime females in e have 16 mg or 24 mg total food and 2 mg or 3 mg food/larva after day 4 and about half those Prime females pupate before the final input. The Prime females in e that pupate after day 6 have 32 mg total food and 4 mg food/larva. For age at pupation, the total food seems to be more important than the food/larva.
6. The next in sequence is the low food, low density, 6 day timespan treatment, f; these Prime female take longer (7.20 days, 0.33 days longer), but grow larger (0.58 mg larger) than the most competition, 3 day timespan females, g. They delay pupation in response to the low food level until after the final aliquot on day 6, then grow for just over a day before pupating. These treatments receive 1 mg or 2 mg food/larva on day 6 relative to the 2 mg or 3 mg of food/larva before day 6. Compare this to the equivalent food/larva, but greater total food in #18 above. The greater total food allows the Prime females in e to pupate both earlier and larger than those in f, despite equivalent food/larva in both. Prime females dominate competition and disproportionately benefit from both higher food/larva levels and greater total food.
7. The latest to pupate is the most competition, 6 day timespan treatment, h. These females also delay pupation in response to the low food level and grow for 3.36 days after the final aliquot of food. Despite the delay and the equivalent food (both total food and food/larva after day 6), they do not grow as large as the Prime females in the most competition, 3 day timespan treatment (g; equivalent food, delivered earlier). Late additions of food do not result in equivalent growth of Prime females compared to earlier additions of food.
8. Another source of the FxDxT interaction for the Prime female age at pupation appears to be the tight distribution of ages of the earliest four treatments (a-d; corresponding also to the largest masses) compared to the broad spread of the later four treatments (e-h).

**Estimated growth rates (Prime female mass divided by Prime female age)**

Age and mass at pupation are not expected to vary independently; the negative discriminant function for Prime female age suggests that age and mass are inversely related. The estimated growth rates for the Prime females in the 8 treatments of this interaction allow us to combine the two measures in a biologically meaningful way and to look for other potential sources of the interaction.

1. The highest estimated growth rate (0.88) is for the least competition, 3 day timespan treatment (a); this is expected to be the most optimal microcosm across this interaction.
2. The estimated growth rate for the high food, high density treatment with the 3 day timespan (b) is higher than that of the least competition at the 6 day timespan (c), as also observed for the independent mass and age at pupation variables.
3. Across the four food x density treatments, the estimated growth rate for the treatments with the 6 day timespan is always lower than that for the treatments with the 3 day timespan.
4. The growth rates for the least competition treatments (a and c) are both high, and the growth rate estimates for the most competition treatments (g and h) are lower than all the other treatments.
5. The difference between the growth rates for the test tubes with the 6 day timespan and those of the 3 day timespan is much larger for the two intermediate levels of competition (b versus e, d versus f) than for either the most competition or the least competition. This reflects the observations in #9-11.
6. Timespan appears to have a greater effect on the growth rate of the Prime females at intermediate levels of competition than at the extremes. This is another potential source of the 3-way interaction.
7. For this interaction, the highest growth rates correspond to the four treatments with the largest and earliest pupating Prime females (a-d). For these four, the mass and age are inversely related; the highest mass is associated with the lowest age. This is the relationship indicated by the sign of the discriminant function coefficients for these two variables for this contrast in the MANOVA.
8. For the remaining four treatments, the growth rate corresponds to the mass of the female rather than to the age at pupation (the masses and ages are ordered differently from one another across these four treatments). The differences in mass are greater than the differences in age at pupation. The r squared value for the Prime female mass is 3 times that of the Prime female age for this interaction.
9. Timespan translates into less available food for the Prime females in some treatments where the Prime female age at pupation is 6 days or less because the final aliquot of food in the 6 day timespan was added after the pupae were removed.
10. However, for the four treatments where some of the females pupated after day 6, the Prime females did not grow as large as those in the corresponding 3 day timespan despite the equivalent food.
11. The late addition of food is not as useful to the Prime female as the same amount of food earlier in the larval lifespan.
12. Competition for food is increased by the longer timespan; the delay in adding the last aliquot of food affects both the mass and age at pupation of the Prime female, but the effect of the timespan on Prime female mass at pupation is smallest in the test tubes with the most competition and greatest in the test tubes with intermediate levels of competition.
13. The effect of timespan on Prime female age at pupation is smallest in the test tubes with the least competition and greatest in those with the most competition, with the intermediate levels of competition being intermediate. The estimated growth rates for Prime females indicates that timespan also affects the intermediate levels of competition more than the extremes; it resembles the pattern of Prime female mass more than age, again reflecting the relative r squared values.

Prime females dominate the competition in the test tubes. Both mass and age at pupation respond to the factors: food, density and timespan. Food and density jointly set up four different competitive environments, and timespan modifies those environments by delaying the input of food. There are two aspects of the food supply that affect competition among females; the total food in the test tube, and the food/larva. Timespan affects both of these, while density affects primarily the total food. The Prime female mass at pupation follows the total food (affected by the density and timespan) at high levels of food/larva (4 mg or greater). The mass at pupation follows the food/larva at levels less than 4 mg food/larva, and the total food influences the mass when the food/larva is equal across treatments (e.g. high food, high density versus low food, low density for the 6 day timespan). In contrast, the Prime female age at pupation follows the total food per test tube with the food/larva influencing the age at pupation when the total food is equal across treatments. The Prime female mass at pupation is affected by this interaction 3 times as much as the Prime female age at pupation, and this is reflected in the estimated growth rate for the Prime female. The estimated growth rate conforms to the Prime female mass rather than the Prime female age at pupation.

**Average female mass**

1. The Average female mass at pupation is similar to the Prime female mass at pupation. The letters, a-h, refer to the order of the Prime female mass at pupation; the Prime female dominates the competition with the non-Prime females.
2. The Average female mass is affected by competition (food x density); the highest values are in the least competition treatments (a and c), followed by the two intermediate competition treatments (b, d, e, and f, which overlap). The lowest values of Average female mass are in the most competition treatments (g and h).
3. The highest value is in the test tubes with the least competition and the 3 day timespan (a). The lowest value is in the test tubes with the most competition and the 6 day timespan (h).
4. The Average females in the test tubes with the least competition (a and c) are larger than those in all other treatments. The Average female mass in the test tubes with the least competition and the 3 day timespan (a) is larger than any of the Prime female masses in the other treatments (b-h), suggesting that this is the optimal environment for all females.
5. The Average females in the test tubes with the most competition are smaller than those in all other treatments (g and h).
6. The Average females in the test tubes with intermediate levels of competition (b, d, e, and f) are intermediate and overlapping in mass. The Average females in the test tubes corresponding to the four largest Prime females (a-d) are larger than the four smaller Prime females (e-h). These Average females (in a-d) have 4 mg food/larva or more, while the Average females in e-h have less than 4 mg food/larva.
7. The Average females in the test tubes with the 3 day timespan are larger than those in the corresponding test tubes with the 6 day timespan.
8. This is not the same pattern as for the Prime female mass. The Average females in the least competition, 6 day timespan treatment are the second largest in this interaction, which is different from the Prime females in that treatment. The Average female mass follows the food/larva on day 4 rather than the total food, with the total food affecting the Average female mass when the food/larva is equal. This pattern holds for all 8 treatment combinations.
9. Across the four competition (food x density) treatments, the Average female mass is always higher with the 3 day timespan than with the 6 day timespan.
10. Analogous to the Prime female mass and estimated growth rate, the difference between Average female mass with the 3 day timespan and that with the 6 day timespan is much larger for the intermediate levels of competition than for either the least competition or the most competition. Both the least competition treatments have more than 4 mg food/larva on day 4, and both the most competition treatments have less than 4 mg food/larva on day 4. The intermediate competition treatments with the 3 day timespan both have 4 mg food/larva on day 4, while the intermediate competition treatments with the 6 day timespan both have less than 4 mg food/larva on day 4.
11. For the Average female, the longer timespan increases competition for food; the delay in adding the last aliquot of food decreases the Average female mass, but the effect of the timespan is smallest in the test tubes with the most competition and greatest in the test tubes with intermediate levels of competition. This is likely the source of the 3-way interaction for the Average female mass.
12. As for the Prime females, the Average females in the 6 day timespan treatments do not catch up to the mass of their counterparts in the 3 day timespan treatments. The late addition of food is not as beneficial as receiving it earlier in the larval lifespan.

**Prime female mass MINUS Average female mass**

1. The difference between the Prime female mass and the Average female mass is an indication of the size distribution, or the relative advantage of the Prime female over the non-Prime females. Interference competition results in smaller Prime and Average females, with a larger difference between them. For each competition treatment (food x density) the 6 day timespan results in a larger difference between the two masses than the 3 day timespan. There is less food in the test tubes with the 6 day timespan until after the 6th day, so competition for food should be more intense prior to that.
2. The Prime female dominates the competition and is relatively larger than the Average female in the 6 day timespans versus the 3 day timespan, although there is only 0.01 mg difference between the two most competition treatments (see S21 Table). These are the treatments where interference competition should occur, and the masses of both Prime and Average females are smallest overall here, but the difference between the Prime and Average female masses are in the middle of the range, suggesting that there is no interference competition.
3. Most of the differences between the Prime female mass and the Average female mass lie in the range 0.16 mg to 0.27 mg, with two outliers at the top and bottom.
4. The smallest difference is in the least competition, 3 day timespan test tubes (0.08 mg), where we expect the competition to be least because the amount of food is high and the shorter timespan delivers the food earlier in the larval lifespan.
5. The largest difference between Prime and Average female masses is in the high food, high density, 6 day timespan test tubes (0.38 mg). The Prime females in these test tubes pupate on day 6 or day 7, so they do not greatly extend their larval life to take advantage the additional food added at the end of day 6. The non-Prime females do not grow larger on either the release from competition after the Prime females pupate or on the increment of food at the end of day 6.
6. There is some aspect of competition (food x density) in these test tubes that limits the growth of the non-Prime females so that the final aliquot of food, combined with the release from competition with the Prime female at her pupation, does not result in further growth of the non-Prime females.
7. Interference competition could account for this observation, but it seems unlikely that interference competition would occur at intermediate levels of competition only. A simpler explanation would be that the non-Prime female larvae are limited by their size in the molt between the 3rd and 4th instars. They can grow only as large as the size of their head capsule allows, or they are limited by the size of their mouthparts (affecting their feeding rate), or some other aspect that is determined by that molt.

**Prime female mass and age, and Average female mass**

1. For these three variables (Prime female mass and age, Average female mass) across the food x density treatments that create different levels of competition, masses are always greater and the ages at pupation are earlier with the 3 day timespan compared to the 6 day timespan in each competitive environment.
2. The best outcome (largest masses and the earliest pupation) for females in this interaction is in the test tubes with the least competition and the 3 day timespan.
3. The test tubes with the least competition produce larger females than the test tubes with intermediate levels of competition with one exception: the Prime female mass at the high food, high density treatment with the 3 day timespan is larger than the Prime female mass at the least competition with the 6 day timespan.
4. More generally, the effect of timespan on competition is greater at the intermediate levels of competition for both mass variables; this is likely the source of the 3-way interaction for these variables.
5. The worst outcome for females (smallest masses and the latest pupation) is in the test tubes with the most competition and the 6 day timespan. The test tubes with the most competition produce females with smaller masses than the test tubes with the least competition and the test tubes with intermediate levels of competition regardless of the timespan.
6. Prime female age at pupation is inversely related to mass overall, with one exception; Prime females in the low food, low density, 6 day timespan test tubes delay pupation longer than those in the most competition, 3 day timespan, presumably to grow larger.
7. Female larvae modify both mass and age at pupation, independently under certain circumstances, to respond to the environmental conditions presented to them. Prime females increase in mass in response to total food at high levels of food/larva (4 mg or greater on day 4), but follow the food/larva at lower levels of food/larva (less than 4 mg on day 4) with the total food affecting the outcome for equivalent levels of food/larva. Prime female age at pupation follows the total food in the test tube at all levels of food/larva. The food/larva affects the age at pupation when the total food is equal across treatments. Average female mass at pupation follows the food/larva, pupating at larger masses with higher levels of food/larva.

Females dominate competition in the test tubes. Prime females increase in mass in response to the total food in the test tubes at levels of food/larva of 4 mg or greater on day 4, but in response to the food/larva at lower levels on day 4. Prime females pupate earlier in response to higher levels of total food on day 4 at all levels of food/larva. Average females are larger at higher levels of food/larva with total food affecting mass when the food/larva is equal across treatments. That Prime females grow in response to total food at high levels of food/larva while Average females do not suggests that the non-Prime females are diminished in equal measure to the success of the Prime female (causing the Average to follow the food/larva despite the larger size of the Prime female at higher total food levels. Competition is affected by total food (density and timespan) and by food/larva (density). The outcome follows the total food and/or food/larva on day 4 because many Prime females pupate before the final food input on day 6 (for the 6 day timespan treatment). Average females do not appear to benefit from the late addition of food on day 6 or any release from competition after the pupation of the Prime female. No evidence of interference competition is apparent.

**Prime male mass**

S23 Table shows the means and standard errors for the food x density x timespan interaction for the Prime male mass at pupation, the Prime male age at pupation and the Average male mass at pupation. The treatments are labelled a-h, ranked from largest to smallest by Prime male mass. Heuristic 3D plots of the means for Prime male mass, Prime male age, Average male mass and the difference between the Prime male mass and the Average male mass are presented in S11 Fig-S14 Fig.

1. The r squared values for these three variables for this contrast are 0.10, 0.16, and 0.16, respectively, out of a total r squared for each of 0.84, 0.93, and 0.87. This contrast explains 10%, 16% and 16% of the variability in Prime male mass, Prime male age and Average male mass across the experiment.
2. The Prime male mass is greatest in the test tubes with the least competition, somewhat less in the test tubes with intermediate competition, and lowest in the test tubes with the most competition.
3. Within each level of competition, the test tubes with the 3 day timespan produce larger Prime males than the test tubes with the 6 day timespan.
4. The largest value of the Prime male mass is in the least competition, 3 day timespan treatment (a).
5. The smallest value of the Prime male mass is in the most competition, 6 day timespan treatment (h).
6. Similar to the situation with the Prime females, the Prime male mass in the high food, high density, 3 day timespan (b) is larger than that in the least competition, 6 day timespan (c).
7. Also similar to the situation with the Prime females, the effect of the timespan treatment is greater at the intermediate levels of competition (b versus e and d versus f) than at the least competition (a versus c). The difference between the Prime male mass in the 3 day timespan treatment (b and d) and that in the 6 day timespan treatment (e and f) is 0.39 mg for both the high food, high density test tubes and the low food, low density test tubes. The difference between the Prime male mass across the two timespan treatments is only 0.06 mg for the least competition test tubes (a and c).
8. In contrast to the Prime females, the effect of the timespan treatment is greatest in the test tubes with the most competition (g and h, 0.61 mg).
9. The four treatments in which the Prime female grew largest also produced the largest Prime males (a-d); however, these males are more similar in size than the females in the same treatments. These Prime males range from 2.64 mg to 2.77 mg (0.13 mg difference, about 5% of the largest mass), while the females range from 4.42 mg to 4.82 mg (0.40 mg difference, about 8%). Between the four largest Prime males (a-d) and the next group (e-g), there is a 0.30 mg difference in size. These three Prime males range from 2.12 mg to 2.34 mg in size, a difference of 0.22 mg. Furthermore, 7 of the 8 Prime males are in the range from 2.12 mg to 2.77 mg (0.65 mg), with the last Prime male reaching only 1.51 mg (0.61 mg below the lowest of the other 7). While the order of the Prime male masses reflects the total food and food/larva that corresponds with the Prime female mass and age, and Average female mass in this interaction, the magnitude of the differences among Prime males does not reflect the differences in size among females.
10. The 3 factors of this interaction, likely reinforced by the effect of competition with the females, result in Prime males that grow best in the test tubes where competition is least intense and food is most abundant, but result in Prime males that are very small in the test tubes where competition is most intense and the food is least abundant (h; most competition, 6 day timespan). The small range of sizes of the Prime males in the suggest that Prime males reach an upper limit in size based on the total food, food/larva, and density and pupate.
11. All these asymmetries likely contribute to the significance of this interaction for the Prime male mass.

S23 Table. Means (SE) for Prime male mass and age at pupation and Average male mass at pupation for the interaction FxDxT

| Food x Density | Timespan | Rank by Prime male mass (a-h) | Prime male mass at pupation (mg) | Prime male age at pupation (days) | Average male mass at pupation (mg) | Estimated Prime male growth rate (mg/day) | Prime male mass MINUS Average male mass (mg) |
| --- | --- | --- | --- | --- | --- | --- | --- |
| Low food, low density (4 mg/larva) | 3 days | d | 2.64 (0.04) | 5.12 (0.01) | 2.61 (0.00) | 0.52 (0.01) | 0.03 (0.04) |
|  | 6 days | f | 2.25 (0.37) | 5.00 (0.00) | 2.19 (0.44) | 0.45 (0.11) | 0.06 (0.57) |
| Most competition (2 mg/larva) | 3 days | g | 2.12 (0.05) | 5.10 (0.14) | 2.00 (0.02) | 0.42 (0.03) | 0.12 (0.05) |
|  | 6 days | h | 1.51 (0.32) | 5.70 (0.28) | 1.65 (0.18) | 0.26 (0.07) | -0.14 (0.37) |
| Least competition (8 mg/larva) | 3 days | a | 2.77 (0.01) | 5.07 (0.10) | 2.65 (0.08) | 0.55 (0.02) | 0.12 (0.08) |
|  | 6 days | c | 2.71 (0.24) | 5.00 (0.00) | 2.69 (0.26) | 0.54 (0.08) | 0.02 (0.35) |
| High food, high density (4 mg/larva) | 3 days | b | 2.73 (0.08) | 5.00 (0.00) | 2.57 (0.04) | 0.55 (0.03) | 0.16 (0.09) |
|  | 6 days | e | 2.34 (0.24) | 5.00 (0.00) | 2.21 (0.25) | 0.47 (0.07) | 0.13 (0.35) |

**Prime male age**

1. The Prime male age at pupation differs from the Prime male mass in that seven of the eight treatments pupate early (a-g; 5 days-5.12 days) and only one pupates relatively late (h; 5.70 days), but still before the last aliquot of food was added on the 6th day.
2. This is a different pattern from the Prime female age at pupation.
3. The latest pupation is in the test tubes with the most competition and the 6 day timespan, h; these are the test tubes with the least food/larva during the male larval period.
4. Two of the 3 day timespan treatments pupate later than their corresponding 6 day treatments; these are least competition, 3 day timespan, a, and low food, low density, 3 day timespan, d. In both cases, these 3 day timespan treatments have more food after day 4, suggesting that the Prime male delays pupation to grow larger.
5. Males may delay pupation when food is scarce, but also may delay pupation when food is available, presumably to grow larger.
6. Males pupate earlier than females under the best conditions, and are able to pupate on less food than females as demonstrated by their consistent earlier pupation and smaller size at pupation, as well as their more similar masses and ages at pupation across all the treatment combinations in this interaction.

**Estimated growth rate (Prime male mass divided by Prime male age)**

1. The Prime male age at pupation does not vary greatly across the treatments, with the exception of the most competition, 6 day timespan treatment, so the estimated growth rates largely reflect the Prime male mass.
2. The test tubes with the most competition and 6 day timespan (h) produced the smallest males as well as the latest to pupate, so the estimated growth rate for those Prime males is about half of that of the larger ones.

Prime males grow and pupate as though they are affected by the total food and food/larva in the same way as the Prime females (see S22 Table), but the Prime males pupate at much smaller sizes and earlier, and they are more similar in size across treatments except for the most competition, 6 day timespan treatment. Competition with females may account for some of the compression in size, especially for the Prime males in h, but the early, largely simultaneous pupation on day 5 suggests that the males minimize the time to pupation rather than maximizing size at pupation. However, in two cases, Prime males with greater access to food delayed pupation slightly, perhaps to grow larger (or to improve some internal measure of health).

**Average male mass**

1. The Average male mass is affected by competition and timespan, but in a different manner from the Prime male mass. The Average male mass is highest in the two least competition treatments (corresponding to Prime male mass a and c), intermediate and overlapping in the four intermediate competition treatments (b, d, e, and f), and lowest in the two most competition treatments (g and h). In contrast to the Prime male mass, the size order of the Average male mass is: c, a, d, b, e, f, g, h. The Prime male mass followed the same relationship(s) between total food and food/larva as did the Prime female mass (see S22 Table). The Average male mass deviates from this pattern at the higher levels of total food and food/larva (Prime males a-d). Competition is more important than timespan in the least competition and most competition treatments, but timespan is more important than competition in the intermediate competition treatments.
2. In three of the four competition treatments the 3 day timespan produces larger Average males than the 6 day timespan. However, in the least competition, 6 day timespan test tubes (c), the Average male mass is larger than that in the least competition, 3 day timespan test tubes (a). The Average male should grow larger from the additional food in the last aliquot delivered on the 6th day, both because of a release from competition with the Prime male after it pupates and from the extra food. The least competition treatments provide the best growing environment for Prime and Average females and for Prime males, but only the Average male grows larger in the 6 day timespan test tubes (c) than in the 3 day timespan ones (a). This is likely to be part of the source of the 3-way interaction for the Average male mass.
3. The difference between the Average male mass in these two treatments is also small (0.04 mg) relative to the difference between the 3 day timespan and the 6 day timespan in the other three competition treatments (0.35 mg-0.42 mg). This suggests that the least competition treatments both provide sufficient food for all the larvae to grow to optimum size regardless of the timespan treatment.
4. Another difference between the size order of the Prime male masses and that of the Average male masses is the two intermediate levels of competition with the 3 day timespan (b and d). In contrast to the Prime male mass, the Average males in d grow larger than those in b. The Average male mass in d is 2.61 mg, which is almost as large as the Prime male mass in d (2.64 mg). B and d are both 3 day timespan treatments so the larvae receive all the food by the end of day 3. D has the same food/larva as b (4 mg food/larva), but only half the total food (16 mg in d versus 32 mg in b). The food treatment alone doesn’t explain this difference, and there is no late addition of food due to the timespan treatment, so the anomalous difference in size between the two Average male masses and relative to each of the respective Prime male masses must be due to competition. The only factor in this comparison is the release from competition for the non-Prime males after the Prime male pupates. In d, the Prime male pupates at a smaller mass because of the lower total food relative to b, and the non-Prime males benefit from this. In b, the Prime male grows faster than in d and pupates earlier and at a larger size, leaving less available food for the non-Prime males. Competition among males happens against the background of competition among females, and there are 8 larvae in b compared to 4 larvae in d, so the effect of competition on the outcome for males is greater at the higher density.
5. Another potential source of this interaction is that the Average male grows larger than the Prime male in the treatment combination most competition, 6 day timespan (h). This is the worst outcome for both Prime and Average male mass, but the Average male mass is 0.14 mg larger than the Prime male mass; one or more non-Prime males grew larger than the Prime male on the last aliquot of food on day 6. The reason that Average males in the most competition, 6 day timespan, grow larger than the Prime male is likely due to the release from competition after the Prime male pupates, as well as the final aliquot of food.
6. Similarly, the release from competition with the Prime male and the additional food from the last aliquot of food on day 6 allow the Average males to attain a size almost equal to the Prime male in the least competition, 6 day timespan treatment (c).
7. It is likely that the two intermediate competition treatments with the 6 day timespan (e and f) also experience a release of competition after the pupation of the Prime male, and benefit from the food input on day 6. The Prime males in e are larger than those in f despite similar levels of food/larva (0.09 mg larger, about 4% of the larger male mass); the Average males in e are also larger than those in f, but the difference between the two is 0.02 mg (less than 1% of the larger mass). This suggests that the non-Prime males grow larger on the reduced competition and increased food in all of the 6 day timespan treatments, even though they do not grow as large as the Prime male except in h, the environment with the most extreme competition across this interaction.

The Average male mass is affected by competition and by timespan. The two least competition treatments produce the largest Average male masses. In contrast to Prime and Average females, and Prime males, the Average males grow larger in the 6 day timespan treatment than in the 3 day timespan treatment. The Average male masses are also more similar across these two treatments than the corresponding Prime male masses. The least competition treatments provide the optimal growth environment for the Prime and Average females and the Prime males. Despite this, only the Average male mass is larger in the 6 day timespan, suggesting that only the non-Prime males take advantage of the large final food input on day 6 and the release from competition when the Prime male pupates. It is likely that the non-Prime males grow larger on the reduced competition and increased food after day 6 in all of the 6 day timespan treatments. In treatment h, the test tubes with the most extreme competition across this interaction, the non-Prime males grow larger than the Prime males on the final food input.

Another difference from the Prime and Average females, and the Prime males, is the size order of the two intermediate competition treatments with the 3 day timespan (b and d). For the females and Prime males, the masses in the high food, high density treatment, b, are larger than those in the low food, low density treatment, d. For the Average males, the order is reversed. In both treatments all the food is added by the end of day 3, so there is no late input of food; the difference between these treatments must be due to competition. The food/larva is the same in both treatments (4 mg food/larva), and the total food is higher in b, where the Average male mass is lower. The increased density results in increased competition which benefits the Prime male over the non-Prime males; the Prime males grow faster and larger on the higher total food and the non-Prime males are smaller than the non-Prime males in the low food, low density, 3 day timespan treatment, d. This suggests that males compete differently or for a different aspect of the food supply than females. Average females appear to experience more competition in the high food, high density, 3 day timespan (b) treatment than in the low food, low density, 3 day timespan treatment (d), but the Average female mass is greater in b than d, as it is for Prime females and for Prime males.

**Prime male mass MINUS Average male mass**

1. The difference between the Prime male mass and the Average male mass is an indication of the relative advantage of the Prime male over the non-Prime males. (See S22 Table, Prime male mass MINUS Average male mass.)
2. In the test tubes with the least competition (a and c), both the Prime male and the Average male mass are large, but the Prime male grows largest (2.77 mg) across this interaction and is 0.12 mg larger than the Average male in the 3 day timespan treatment (a).
3. The Prime male is only 2.73 mg (0.04 mg smaller than the largest Prime male) in the high food, high density, 3 day timespan treatment (b; with the same amount of food, but higher density) but is 0.16 mg larger than the Average male in that treatment.
4. Density (or total food) has a larger effect on the Average male mass than on the Prime male mass.
5. Comparing the high food, high density, 3 day timespan treatment (b) to the low food, low density, 3 day timespan treatment (d; same 4 mg food/larva), the Prime male mass is higher at the high food level (2.73 mg versus 2.64 mg), but the Average male mass is higher at the low density (2.57 mg versus 2.61 mg).
6. Increased density (or total food) again has a larger effect on the Average male mass than on the Prime male mass.
7. Comparing the high food, high density, 6 day timespan treatment (e) with the low food, low density 6 day timespan treatment (f; same food/larva as the previous comparison, but with the longer timespan), the Prime males grow larger at high food (2.34 mg versus 2.25 mg), but the Average males are almost the same size in both treatments (2.21 mg versus 2.19 mg), and the Average males are larger than the Prime males in both most competition treatments (g and h; 0.09 mg and 0.68 mg larger for e versus g and f versus h, respectively).
8. This suggests that the non-Prime males in the intermediate competition treatments do experience a release of competition after the pupation of the Prime male, and grow larger on the additional food on the 6th day.
9. Density, food/larva, total food, release from competition and the last aliquot jointly affect the Average mass of males.
10. Overall, food inputs earlier in the larval period are better for both Prime and Average males.
11. Average males can grow further on food added after the Prime male pupates, and in some cases can surpass the size of the Prime male, but except for the least competition treatment, they do not catch up to treatments with earlier food inputs (3 day timespan).

Average male mass shows the effect of these factors on the non-Prime males. The Prime male dominates competition among males, and females dominate competition in general through their larger size and retention of particles, so the non-Prime males are most affected by competition and the food levels that mediate competition. Prime males appear to minimize their age at pupation, reaching a minimum size determined by environmental conditions, and pupating. Non-prime males may adopt a different life history strategy. It is possible that after missing the early pupation target, these male larvae grow to a larger size on the increased food in order to benefit from being a larger adult rather than just being another small male.

**Males versus females**

Males are affected by the food, density, and timespan treatments, but also by the competition with females. Males should grow larger and pupate earlier when food is abundant and competition with females is least. The size of females is an indication of the outcome of competition, so larger sizes indicate less competition. Females grow largest in the test tubes with the least competition and those with intermediate competition and the 3 day timespan (a-d). Males also grow largest in these test tubes. Females are smaller in the test tubes with intermediate competition and the 6 day timespan (e and f), and smallest in the test tubes with the most competition (g and h). Males are also smaller in the test tubes with intermediate competition and the 6 day timespan, and smallest in the test tubes with the most competition. However, the FxDxT treatments affect male and female growth as well as competition among females, so the effect of competition among females on the competition among males is confounded.

1. For all four mass variables, there are four treatment combinations where masses are highest suggesting lower levels of competition: a) least competition with 3 day timespan; b) least competition with 6 day timespan; c) high food, high density with 3 day timespan; d) low food, low density with 3 day timespan.
2. For males, these masses are similar and overlapping (2.64 mg to 2.77 mg for Prime male mass, 0.13 mg or 5% of the largest Prime male mass, and 2.57 mg to 2.69 mg for Average male mass, 0.12 mg or 4% of the largest Average male mass).
3. For females, these masses are larger and more dispersed (4.42 mg to 4.82 mg for Prime female mass, 0.40 mg or 8% of the largest Prime female mass, and 4.25 mg to 4.74 mg for Average female mass, 0.49 mg or 10% of the largest Average female mass).
4. There is a large gap (0.30 mg or greater—more than 10% of the respective masses) between the lowest of these values and the rest of the values for both the Prime male and Average male masses at pupation and an even larger gap for the female masses (0.51 mg and 0.72 mg for Prime and Average females, but still just over 10% of the respective masses).
5. The corresponding female masses are higher (1.78 mg to 2.05 mg greater for the Prime female, and 1.64 mg to 2.09 mg greater for the Average female) and more spread out (8% of the mass of the largest Prime female, 10% of the mass of the largest Average female).
6. In these four treatment combination where competition appears to be lowest, the range of masses of Prime and Average males is compressed relative to the distribution of Prime and Average female masses.
7. These four treatments (a-d) have similar Prime male ages at pupation and so have the highest estimated Prime male growth rates across the eight treatment combinations.
8. In two of the treatments, a, least competition with 3 day timespan, and d, low food, low density with 3 day timespan, Prime males appear to have delayed pupation slightly to grow larger (5.07 days, 5.12 days), while in the other two they pupated at the earliest opportunity (5 days).
9. In these four treatments the Prime female growth rates are higher (0.75 mg/day to 0.88 mg/day) compared to the Prime male growth rate (0.52 mg/day to 0.55 mg/day), and they are also more spread out.
10. Prime males pupate about a half day earlier than the Prime females in these four treatments.
11. All the Prime males and most Prime females pupated on or before day 6.
12. The only treatment combination among these four that did not receive the full complement of food by day 3 is the least competition, 6 day timespan (b); pupation of these Prime individuals occurs before the addition of food on day 6, but the food level is as high or higher in these test tubes, b, than in the test tubes for the low food, low density, 3 day timespan treatment, d. Prime female and male growth rates are compared in the heuristic 3D plot in S15 Fig.
13. For these four treatment combinations (a-d) where competition appears to be lowest across the interaction, males and females grow fastest and largest and pupate earliest.
14. Even at this low level of competition, there are differences across the FxD treatments and across the timespan treatment that may contribute to the interaction. For the Prime female and the Prime male, the largest masses occur in the test tubes with the least competition and the 3 day timespan, a; these are the test tubes with the most food per larva, delivered earliest.
15. The second largest masses occur in the test tubes with the high food, high density treatment and the 3 day timespan, b; these have the same amount of food, also delivered early, but a higher density.
16. The difference between the Prime female masses in these two treatments is 0.24 mg (5% of the mass of the largest female); the difference between the Prime male masses is 0.04 mg (1% of the mass of the largest male). Prime females are relatively more affected by density (or total food) than Prime males.
17. The next largest masses are in the test tubes with least competition and the 6 day timespan, c. The difference between the Prime female masses in b and c is 0.07 mg (1%), while the same difference for the Prime males is 0.02 mg (also 1%).
18. The difference between the Prime female masses in c and d is 0.09 mg (2%), while the difference for the Prime males is 0.07 mg (3%).
19. The increments are additive, so the comparison between the Prime females in b and those in d, both with 4 mg/food per larva is 0.16 mg (3%), while that for the Prime males is 0.09 mg (also 3%). Prime males are more tightly clustered than Prime females and this numerical comparison indicates that Prime females in the best environment, a, grow relatively much larger than other Prime females, while Prime males do not reflect this extra growth.
20. This pattern is exaggerated for the Average female mass: the difference between treatments (a and b) is 0.43 mg, 9% of the largest Average female mass. However, the Average female mass in c is greater than b, and this difference is 0.39 mg, 8%, so the advantage of the Average females in a is greater than the Average females in either b or c. This is greater than the advantage of the Prime females in a over the Prime females in either b or c (see above, 5% and 6% respectively). The differences between the Average female mass in b or c compared to that in d are 0.06 mg or 1% and 0.10 mg or 2% of the larger Average female mass, respectively. The Average female mass is disproportionately greater in a than either the Prime female mass or the Prime male mass.
21. The Average male masses are not in the same order as the Prime male masses (or the female masses), but they are also tightly compressed (the four largest Prime male masses, a-d, range from 2.64 mg to 2.77 mg, 0.13 mg, and the four largest Average male masses range from 2.57 mg to 2.69 mg, 0.12 mg). The largest Average male mass occurs in b and the second largest in a; the difference between these two is 0.04 mg or 1% of the larger Average male mass. The differences between the Average male masses in a and d, and in d and c, are both 0.04 mg (also 1%). The three largest Prime male masses are also separated by 1% of the largest mass, but the Prime male mass in d is 3% smaller.
22. Both Prime and Average females grow much larger in the test tubes with the least competition and 3 day timespan, a; neither Prime nor Average males grow as disproportionately large. This suggests that males are limited by some maximum size or minimum age regardless of the abundance of food.
23. The remaining four treatment combinations are: e, high food, high density, 6 day timespan; f, low food, low density, 6 day timespan; g, most competition, 3 day timespan; and h, most competition, 6 day timespan. Competition appears to be higher for these four treatments as evidenced by smaller masses, lower growth rates, and in some cases, later pupation.
24. These treatments (e-h) show greater differences across male and female endpoints (both pupal mass and age at pupation) than a-d.
25. Both males and females exhibit the same grouping: a gap between a-d and the two intermediate competition, 6 day timespan treatments (e and f), followed by a gap, then the two most competition treatments (g and h).
26. For males the two intermediate competition, 6 day timespan treatments (e and f) are similar in size (2.25 mg and 2.34 mg for Prime males, the difference is 0.09 mg, 3% of the mass of the largest Prime male; 2.19 mg and 2.21 mg for the Average males, the difference is 0.02 mg, 1% of the mass of the largest Average male).
27. For females these treatments are more different (3.58 mg and 3.91 mg for the Prime female, the difference is 0.33 mg, 7% of the mass of the largest Prime female; 3.33 mg and 3.53 mg for the Average female, the difference is 0.20 mg, 4% of the mass of the largest Average female).
28. The gap between these treatments (e and f) and the most competition treatments (g and h) reveals an even larger difference between males and females.
29. For males, the gap is 0.13 mg for Prime males and 0.19 mg for Average males (5% of the largest Prime male mass; 7% of the largest Average male mass), but for females the gaps are 0.58 mg and 0.53 mg (12% of the largest Prime female mass; 11% of the largest Average female mass).
30. Finally, the difference in mass between the two most competition treatments is much greater for the males than for the females.
31. Males in the most competition test tubes with the 3 day timespan, g, are a little smaller than those in the group above, but males in the most competition test tubes with the 6 day timespan, h, are much smaller (0.61 mg for the Prime male, 22% of the largest Prime male mass; 0.35 mg for the Average male, 13% of the largest Average male mass).
32. In contrast, the females are more similar in size to each other and much smaller than the group above (2.78 mg and 3.00 mg for the Prime female, 5% of the largest Prime female mass; 2.57 mg and 2.80 mg for the Average female, 5% of the largest Average female mass).
33. Within these four treatments the males, especially the Prime males, show a distinctly different pattern from the females.
34. Three of the treatments, the two intermediate competition treatments with the 6 day timespan, e and f, and the most competition treatment with the 3 day timespan, g, are smaller in accordance with the lower food, higher density and increased competition, but the males in the most competition, 6 day timespan treatment, h, are much smaller, indicating much greater competitive stress.
35. In contrast, females show a larger difference between the two intermediate competition treatments with the 6 day timespan (e and f) and the two most competition treatments (g and h), and a much smaller difference between the two most competition treatments.
36. Across these four treatments (e-h) both Prime and Average females appear to be affected by competition and timespan incrementally.
37. Prime and Average males are less affected by competition and timespan than the females with the exception of the most competition, 6 day timespan (h).
38. The ages at pupation for the Prime males and females in these higher competition treatments (e-h) also show different patterns.
39. Prime males in the intermediate competition, 6 day timespan treatments (e and f) pupate on day 5, as early as possible.
40. Prime males in the most competition, 3 day timespan treatment (g) also pupate primarily on day 5, but more than half of those in the most competition, 6 day timespan (h) pupate on day 6.
41. All Prime males pupate before the addition of the last aliquot of food on day 6.
42. Some Prime females pupate on day 6 as well, but most of the Prime females in these higher competition treatments delay pupation until after day 6, and some of these delay much further after the addition of the final aliquot of food.
43. For this subset of Prime females the timing of pupation is positively related to the total food and the food/larva in the test tubes on day 5: e, high food, high density, 6 day timespan—16 mg or 24 mg total food, 2 mg/larva or 3 mg/larva; g, most competition, 3 day timespan—16 mg total food, 2 mg/larva; f, low food, low density, 6 day timespan—8 mg or 12 mg total food, 2 mg/larva or 3 mg/larva; h, most competition, 6 day timespan—8 mg or 12 mg total food, 1 mg/larva or 1.5 mg/larva.
44. In these higher competition treatments, Prime males pupate at a size dependent on the total food, food/larva, density and competition, but pupate on or shortly after day 5.
45. Prime females also pupate at a size dependent on these factors, but appear not to be able to pupate with less than 16 mg total food, thus extending their larval lifespan until additional food inputs arrive.
46. Another way to look at these variables is through the estimated growth rates. The Prime female growth rates for these four higher competition treatments (e-h; 0.30 mg/day to 0.60 mg/day) are lower than those for the group of four lower competition treatments (a-d; 0.75 mg/day to 0.88 mg/day).
47. The same is true for the Prime males (0.26 mg/day to 0.47 mg/day for the four higher competition treatments, e-h, compared to 0.52 mg/day to 0.55 mg/day for the four lower competition treatments, a-d).
48. Similarly to the pattern for masses, the pattern of growth rates for the Prime male groups the intermediate competition, 6 day timespan treatments, e and f, and the most competition, 3 day timespan, g, closer together, with a larger difference between those and the most competition, 6 day timespan, h.
49. Equally interesting, the differences between the Prime female growth rates and the Prime male growth rates in the low food, low density, 6 day timespan treatment, f, and the two most competition treatments, g and h, are very small (0.02 mg/day to 0.05 mg/day).
50. In the test tubes with the most competition Prime males and females both grow slowly, but Prime males sacrifice size and pupate on day 5 or 6, while Prime females extend their larval lifespan to grow larger. Prime female and male growth rates are compared in the heuristic 3D plot in S15 Fig.
51. The difference between the Prime female mass and the Prime male mass is another way to examine the relative advantage of the Prime female over the Prime male across treatments. The largest differences, indicating the largest female advantage, are in the test tubes with the lower levels of competition (a-d). These differences range from 1.78 mg to 2.05 mg, and correspond to the size of both Prime female and Prime male.
52. Where the Prime female is largest, the difference between the two is also largest, despite the large size of the Prime male. These relationships are reasonable because they are based on exponential growth processes in response to varying levels of food (and density and competition).
53. This pattern continues through the high food, high density, 6 day timespan, e, and low food, low density, 6 day timespan, f, treatments (both intermediate levels of competition).
54. However, in the most competition test tubes the difference between the Prime female and the Prime male in the 3 day timespan treatment, g, is smaller than that in the 6 day timespan treatment, h, despite the larger size of both Prime female and Prime male in the 3 day timespan treatment. A heuristic plot of the differences is presented in S16 Fig.
55. In the six treatments: least competition for both timespans (a and c); high food, high density for both timespans (b and e); and low food, low density for both timespans (d and f); the masses of the Prime female and the Prime male and the difference between the two are always greater for the 3 day timespan than for the 6 day timespan. The larvae in these 3 day timespan treatments had more food, earlier in their lifespan, than the larvae in the corresponding 6 day timespan.
56. Across these 6 treatments the Prime female outcompetes the Prime male for food, and the relative advantage of the Prime female over the male increases as the food level increases and with the shorter timespan (also an increase in food level).
57. This relationship also holds true for the Average female mass compared to the Average male mass, so all females outcompete the males for food and the female advantage increases with increasing food level and the shorter timespan.
58. This should also be true of the larvae in the most competition test tubes; the 3 day timespan treatment provides all the food by day 3 while the 6 day timespan adds the final aliquot of food at the end of day 6, after the Prime males pupate. In the most competition test tubes with the 6 day timespan, h, the Prime males pupate at the smallest mass across the interaction, but the non-Prime males, and the Prime and Average females grow larger on the addition of the final aliquot of food. The Prime females delay pupation for 3.36 days after the last aliquot of food is added on day 6.
59. This appears to be a different mechanism than the size-related competitive advantage of the females in the other six treatments; the additional food in the last aliquot combined with the early pupation of the Prime male provides a release from competition for the remaining larvae. The Prime females in the most competition, 6 day timespan, h, grow relatively larger than the Prime males, but they don’t grow as large as the Prime females in the most competition, 3 day timespan test tubes, g, with the equivalent amount of food delivered earlier. The Average females are also relatively larger than the Average males in the 6 day timespan, h, compared to the 3 day timespan, g. Despite this additional increment of growth, the Prime females in the most competition, 6 day timespan test tubes, h, are 0.22 mg smaller than those in the most competition, 3 day timespan test tubes, g; the Average females in the most competition, 6 day timespan, h, are 0.23 mg smaller than those in the most competition, 3 day timespan, g. The Average male mass, although larger than the Prime male mass in the most competition, 6 day timespan test tubes, h, is 0.35 mg smaller than the Average male mass in the most competition, 3 day timespan test tubes, g. This is an indication of the difference in growth that food added at different times in the larval lifespan can cause. Food added early in the larval lifespan contributes more to growth and the final pupal mass than equivalent amounts of food later in the lifespan. The Prime males in h are smallest due to the highest level of competition and the least food. The Average males in h are also smallest, due to the same factors, and the release from competition after the Prime male pupates along with the late addition of food after day 6 allows the non-Prime males to grow larger than the Prime male, but does not support growth to the same size as the Average males in g, which receive the food earlier.

In addition to the well-known differences between males and females in pupal size and timing of pupation, there are differences in the way the two sexes respond to variations in the abundance of food. For both sexes the 8 treatments fall into three groups: the four treatments with the most food and less competition (a-d), the two intermediate competition treatments with the 6 day timespan (e and f), and the two most competition treatments (g and h). There are distinct gaps in the masses at pupation between these groups.

Prime females and Prime males grow largest and pupate earliest in the first group. Mass at pupation follows the total food per test tube (after day 4), with the food/larva affecting the outcome when the total food is equal across treatments. The distribution of Prime male masses is compressed relative to Prime female masses, supporting the idea that they grow to a minimum size and pupate early rather than growing to the maximum size allowed by the food supply (as females appear to do). The Average females in these 4 treatments appear to grow in response to the food/larva level with the total food affecting the outcome when food/larva is equal across treatments. This has the effect of favoring the least competition, 6 day timespan treatment over both intermediate competition 3 day timespan treatments. The Prime female benefits from the total food and the non-Prime females are correspondingly diminished (causing the Average female mass to track the food/larva rather than the total food). The effect may be due to lower density, higher food/larva, or the final food input on day 6. The Average male mass also deviates from the pattern followed by the Prime females and Prime males. The two least competition treatments result in the largest Average male masses, but the 6 day treatment is larger than the 3 day treatment, suggesting that release from competition with the Prime male and the extra food from the last food input contribute to the growth of the non-Prime males. The two intermediate competition treatments with the 3 day timespan also switch order compared to the Prime male masses; the Average males in the low food, low density, 3 day timespan treatment grow larger than those in the high food, high density, 3 day timespan. This suggests that density increases competition for the non-Prime males more than the higher total food offsets competition.

The masses of Prime females and Prime males follow the food/larva level (after day 4) in the treatments e-h, with the total food affecting the outcome when the food/larva level is equal across treatments. Prime female masses are more dispersed across these four treatments than across the previous four. The two intermediate competition treatments with the 6 day timespan are separated from the two most competition treatments by a large gap in size, and there are gaps between the two intermediate competition treatments and the two most competition treatments. Prime male masses are also more dispersed across these four treatments than across the previous four. There are gaps between the two intermediate competition treatments with the 6 day timespan, between the intermediate competition treatments and the most competition treatments, and between the two most competition treatments, but the largest gap is between the most competition treatment with the 3 day timespan and the most competition treatment with the 6 day timespan. In contrast to the Prime female masses, where the two most competition treatments are relatively similar compared to the other 6 treatments, the Prime male mass in the most competition treatment with the 6 day timespan is 0.61 mg smaller than that of the most competition treatment with the 3 day timespan. The Prime male mass in the other 7 treatments span a range of 0.59 mg, so this level of food/larva (1 mg or 1.5 mg by day 4) is much harder on the Prime male than on the Prime female. The Average female mass also follows the food/larva level (after day 4) in the treatments e-h, with the total food affecting the outcome when the food/larva is equal across treatments.

The Average male mass nominally follows the food/larva level (after day 4) in treatments e-h, with total food affecting the outcome when the food/larva is equal across treatments. However, the non-Prime males grow larger after the Prime male pupates, and the final increment of food also allows them to grow larger, and the size of the Average male is affected. In treatment h, the Average male grows larger than the Prime male. In treatments e and f, the Average male grows almost as large as the Prime male, and larger than the Prime males in treatments g and h (most competition). Males may pupate earlier and at a smaller size than females, but the relationship between timing, size and food level is flexible for non-Prime males, so that they grow larger than the Prime males on the day 6 food input.

Females are not affected by the pupation of the Prime male. Females benefit from additional food, but only in treatment e is there an anomalous difference between the Prime and Average females. The Average female mass is about 0.20 mg less than the Prime female mass in the most competition treatments, where both Prime and Average female masses are small relative to the other treatments. The difference between the Prime and Average female masses is 0.25 mg for the low food, low density treatment with the 6 day timespan, f, but the difference is 0.38 mg for the high food, high density treatment (e, also intermediate competition, and 2 mg or 3 mg food/larva) with the 6 day timespan. The higher total food in the high density treatment benefits the Prime female over the non-Prime females in this treatment. This may also be the case between the Prime female and the Prime male, although the effect of competition for food and the propensity for males to reach a minimum size and pupate early is difficult to separate. For Prime females, Average females and Prime males, growth and size at pupation appear to follow the food/larva (at day 4), and the differences between the Prime female and the Average female, and the Prime female and the Prime male, are greater in treatment e than in treatment f (equivalent food/larva, but lower total food).

**FxDxT summary**

Timespan interacts with competition for all seven dependent variables. The timespan treatments alter the total food and food/larva within the competition treatments and both these factors have been shown to affect the outcome of competition for males and females. The results are consistent with the prior study on competition [1]. The primary effect of timespan on competition is through changing the total food and food/larva over time. Males and females respond differently to competitive stress; Prime males pupate consistently early and respond to competition by pupating at a lower mass. Prime females require more total food (16 mg in this experiment) in order to pupate at all, and extend their larval lifespan to grow larger in response to additional food late in the lifespan in those treatments where the food was insufficient to pupate. At the same time, Prime and Average females and non-Prime males do not grow as large on the equivalent food provided late in the lifespan compared to that provided earlier. There may be physical constraints (size of body parts at the molt from 3rd instar) that cause this observation in addition to possible physiological constraints. Prime males, Average males, Prime females and Average females all do relatively well at the lower levels of competition (a-d), but there are large differences across the sexes and smaller differences within the sexes when competition is more intense (e-f). The Prime male is most affected by competition (h-most competition, 6 day timespan) pupating at the smallest size and latest across the interaction. The Average male grows larger than the Prime male in this treatment due to the release of competition after the pupation of the Prime male and also the effect of the additional food on day 6. The Prime and Average females are also smallest in this treatment combination, but they are more similar to the most competition, 3 day timespan treatment. They are less affected by timespan when competition is intense and more affected by timespan when competition is intermediate than the males. Females may change the way that they feed (filtering versus retention of particles) as the total food and/or food/larva changes and this may explain the difference between the 3 day timespan and 6 day timespan treatments at the intermediate levels of competition. Finally, Survival appears to improve with the gradual addition of food (6 days versus 3 days) except at the lowest food level where the 6 day timespan may increase the food stress and competitive interactions.

**The FxDxAxT interaction (not significant in the MANOVA)**

The 4-way interaction would indicate that both the number of aliquots of food (2 or 4) and the timespan of delivery of those aliquots (over 3 days or over 6 days) affect the competition among the larvae in the test tubes. Aliquot and timespan both affect the growth of the larvae (S4 Table MANOVA: main effects, FxA, FxT, DxT, AxT and DxAxT interactions) and separately interact with competition (S4 Table, MANOVA: FxDxA and FxDxT interactions), but they do not jointly interact with food and density in the MANOVA or in the ANOVAs with the exception of the Average female mass (S24 Table).

1. The Average female mass at pupation is significantly affected by the 4-way interaction in the ANOVA (r squared<0.005, P<0.05). The values of the means for the separate 2 aliquot and 4 aliquot treatments are presented against the 8 means for the FxDxT interaction for this variable in S23 Table.
2. In 7 of the 8 treatment combinations (FxDxT combinations), the Average female mass is greater with 4 aliquots rather than 2 aliquots, but in the treatment with the least competition and the 3 day timespan, the Average female mass is greater with 2 aliquots rather than 4.
3. The Average females in the least competition, 2 aliquot, 3 day timespan combination grow larger than the Average females in any other of the 16 treatment combinations. This treatment has 16 mg of food delivered on day 0 and another 16 mg of food delivered on day 3. The least competition 4 aliquot, 3 day timespan has 8 mg of food delivered on day 0, day 1, day 2, and day 3. The difference between these two treatments could be the larger amount of food on day 0, or the larger amount of food on day 3. For all the other treatments, the 4 aliquot treatment is better than the 2 aliquot treatment, but this pair of treatments has the most food per test tube, and also the most food per larva, and the shortest timespan, so it is the most food rich environment among the 8 combinations of food, density and timespan.
4. Four treatments also have the large input of food on day 0 (16 mg), but only the high food, low density, 3 day timespan treatment and the high food, high density, 3 day timespan treatment have the second large input on day 3. Only the low density treatment shows an advantage for the Average female mass on the 2 aliquot versus 4 aliquot treatment.
5. The four way interaction suggests that the large addition of food on day 3 enhances the growth of the females, perhaps by providing so much food on day 3 that there is effectively no competition for a period of time.
6. Furthermore, because this affects the Average female mass and not the Prime female mass, this is primarily due to the growth of the non-Prime females in the test tubes. Very high food levels on day 3 benefit the non-Prime females (compared to the non-Prime females in the other treatments).
7. No other variables are affected in this way in either the ANOVAs or the MANOVA, so this apparent release of competition by a large bolus of food on day 3 in this one treatment only affects the non-Prime females.

S24 Table. The means (standard errors) of the Average female mass for the separate 2 aliquot and 4 aliquot treatments presented alongside the 8 mean values for the FxDxT interaction.

| Food x Density | Timespan | FxDxT Means (SE) (mg) | FxDxAxT, 2 aliquots Means (SD) (mg) | FxDxAxT, 4 aliquots Means (SD) (mg) |
| --- | --- | --- | --- | --- |
| Low food, low density (4 mg/larva) | 3 days | 4.25 (0.04) | 4.22 (0.26) | 4.28 (0.23) |
|  | 6 days | 3.33 (0.51) | 2.97 (0.37) | 3.69 (0.42) |
| Most competition (2 mg/larva) | 3 days | 2.80 (0.04) | 2.77 (0.18) | 2.83 (0.30) |
|  | 6 days | 2.57 (0.11) | 2.49 (0.25) | 2.64 (0.16) |
| Least competition (8 mg/larva) | 3 days | 4.74 (0.04) | 4.76 (0.41) | 4.71 (0.79) |
|  | 6 days | 4.35 (0.45) | 4.03 (0.21) | 4.66 (0.39) |
| High food, high density (4 mg/larva) | 3 days | 4.31 (0.16) | 4.20 (0.22) | 4.42 (0.27) |
|  | 6 days | 3.53 (0.75) | 3.00 (0.38) | 4.06 (0.43) |

A large input of food on day 3 effectively eliminates competition among females for a period of time, allowing the non-Prime females to grow larger relative to the non-Prime females that received equivalent food earlier in the life cycle (the 4 aliquot treatment). A similar large input of food on day 6 does not have the same effect. For females, food early in the larval period is more beneficial than food later, and multiple inputs are better than single inputs, but a large input of food on day 3 benefits the non-Prime females by eliminating competition at a critical point in the larval growth.

Female larvae outcompete male larvae, but neither the Prime male mass nor the Average male mass is affected by this interaction and the elimination of competition due to the large food input on day 3. The lack of effect of this specific food input suggests that either the food level is already so high in this treatment that males do not respond to the additional food, or that the final mass of males is already determined by the food level before day 3 (at this high food level, some non-Prime males grow larger than the Prime male in treatments at low food levels).

**The FxDxA interaction (R squared = 0.22)**

Similar to the FxDxT interaction, the interaction between food and density is expected to produce 4 different competitive environments: least competition (high food, low density); most competition (low food, high density) and two intermediate levels of competition (high food, high density, and low food, low density). The least competition test tubes receive 32 mg of food or 8 mg food/larva. The most competition test tubes receive only 16 mg of food or 2 mg food/larva. The two intermediate competition treatments receive 32 mg (for 8 larvae) or 16 mg (for 4 larvae) of food resulting in 4 mg food/larva. Mosquito larvae should grow larger, faster, and pupate earlier in the least competition treatment, followed by the intermediate competition treatments. Pupae should be smallest, grow slowest and pupate latest in the most competition treatment. The high food, high density treatment is expected to be better for mosquito larval growth than the low food, low density treatment because the total food is greater. These four treatments are crossed with the two aliquot treatments (the total food is divided into 2 aliquots or 4 aliquots) for a total of 8 treatment combinations. The interaction between the food x density treatments and the aliquot treatment reveals how the aliquot treatment affects the competition for food among the larvae. This is one of the least significant contrasts in the MANOVA (R squared = 0.22, P<0.001). The discriminant function scores were large for all four mass variables, with the Prime masses having positive scores and the Average masses having negative scores. However, this contrast was only significant for the Prime female mass and the Average female mass in the ANOVAs. Neither variable has a large r squared value (Prime female mass: r squared<0.005, P<0.05; Average female mass: r squared=0.01, P<0.001). Nevertheless, the interaction indicates that the aliquot treatment does have an effect on competition among female larvae (in addition to the FxDxAxT interaction described above).

S25 Table shows the means and standard errors for the Prime female mass at pupation, the Prime female age at pupation and the Average female mass at pupation for the FxDxA interaction. Only the two mass variables were significantly affected by the interaction in the ANOVAs. The Prime female age is included in the table to estimate the growth rate of the Prime females across the treatments. The treatments are labelled a-h based on the size of the Prime female in each treatment combination; the ranks are not the same as for the FxDxT interaction above. Heuristic 3D plots of the Prime female mass and the Average female mass are in S17 Fig and S18 Fig, respectively.

1. The Prime and Average females in the two least competition treatments were larger than their counterparts in all the other treatments and those in the two most competition treatments were smaller than their counterparts, so competition (food x density) is more important than aliquot in this interaction.
2. Prime females and Average females grow larger with 4 aliquots than with 2 aliquots in all competition (food x density) treatment combinations.
3. However, both the Prime and Average females form three groups of similar masses across the 8 treatments. There is a group of three with the largest females (a-c), another group of three in the middle (d-f), and then the smallest females in the two most competition treatments (g and h). The largest Prime females (a-c) range in size from 4.49 mg to 4.81 mg (0.32 mg, 7% of the mass of the largest Prime female). There is a gap of 0.35 mg (also 7%) followed by the next larger group of Prime females (d-f) with a range from 3.86 mg to 4.14 mg (0.28 mg, 6%). There is a larger gap of 0.92 mg (19%) followed by the smallest Prime females (g and h) with a range from 2.86 mg to 2.92 mg (0.06 mg, 1%). The Average female masses follow a similar pattern. These three groups indicate the interaction of the competition and aliquot treatments. The largest females are in the least competition treatments plus the high food, high density treatment with 4 aliquots. The middle group of females are in the low food, low density treatments plus the high food, high density treatment with 2 aliquots. The smallest females are in the most competition group.
4. The aliquot treatment affects competition most noticeably in the high food, high density treatments (intermediate competition) with the 4 aliquot treatment producing females similar in size to the least competition treatments and the 2 aliquot treatment producing females similar in size to the low food, low density treatments (same 4 mg food/larva, but lower total food).
5. Although the ranking (a-h) is based on the Prime female mass, the Average female mass follows it closely. The Prime female mass (4.81 mg) and the Average female mass (4.69 mg) are largest in the least competition treatment with 4 aliquots, a. The next largest females (both Prime and Average) are in the test tubes with the least competition and 2 aliquots, b. The third largest females (both Prime and Average) are in the high food, high density treatment with 4 aliquots, c. The Prime females in the high food, high density, 4 aliquot treatment, c, are only 0.04 mg smaller than the Prime females in the least competition, 2 aliquot treatment, b. The Average females in the high food, high density, 4 aliquot treatment, c, are 0.16 mg smaller than the Average females in the least competition, 2 aliquot treatment, b. In all three of these treatment combinations the Prime females pupate before day 6.
6. The timespan treatment is not a factor in this interaction, so for these high food treatments with 4 aliquots (a and c), the 3 aliquots (8 mg of food on days 0, 2, 4 for the 6 day timespan) and 4 aliquots (8 mg of food on days 0, 1, 2, 3 for the 3 day timespan) are equivalent in promoting the rapid growth of the Prime females. Similarly, in the least competition, 2 aliquot treatment, b, the single aliquot (16 mg of food on day 0 for the 6 day timespan) is equivalent to the 2 aliquots (16 mg each on days 0 and 3 for the 3 day timespan). Within each of the competition levels the females in the 4 aliquot treatment grow larger than those in the 2 aliquot treatment, so multiple food inputs are better for growth than a large input on day 0 followed by another large input on day 3 or day 6 (in this case after the Prime female pupates).
7. The absence of an effect of timespan on the Prime female mass suggests that early food abundance is more important to competition among female larvae than later additions. The effect of the timespan treatment is to delay the final aliquots of food; this has a very significant effect on competition independent of the aliquot treatment, but no 4-way interaction. (See the exception to this above in the description of the FxDxAxT interaction for the Average female mass.)
8. The high food, high density, 2 aliquot treatment, e, and the two low food, low density treatments (d and f) are similar in size, and about midway between the three treatment combinations that produced the largest females (a-c), and the two most competition treatment combinations that produced the smallest females (g and h). The Prime females in these 3 treatments (d-f) all pupated on the 6th day or just afterwards, in contrast to the larger females that pupated a day earlier and the smaller females (most competition) that pupated more than a day later.
9. Within this group of 3 intermediate sized females, the largest Prime females (4.14 mg) are in the low food, low density, 4 aliquot treatment, d. The Prime females in these test tubes have the same food/larva but less total food than the females in the high food, high density, 2 aliquot ones, e, yet they grow larger (0.13 mg larger). The Prime females in the low food, low density, 4 aliquot treatments, d, receive 12 mg or 16 mg of food by day 4 (in 4 mg aliquots) while the females in the high food, high density, 2 aliquot treatments, e, receive 16 mg or 32 mg of food (in 16 mg aliquots). This suggests that multiple food inputs promote growth better than a larger, single initial input (even followed by an additional large input).
10. The Average female mass parallels the Prime female mass as described above, with one exception. The low food, low density, 2 aliquot treatment, f, produced smaller Prime females (3.86 mg) than the other two treatments in this group, but the Average females in both the 2 aliquot treatments (e and f) are the same size (3.60 mg). This suggests that the total food/test tube has less effect on the Average females than the food/larva does (4 mg/larva in both treatments), a difference between the Prime and Average females. (See also the discussion of the interaction FxDxT above.)
11. The Prime and Average females in the two most competition treatments are about 2 mg smaller than the largest females and about 1 mg smaller than the next larger females. The females in the most competition, 4 aliquot test tubes, g, are larger than those in the most competition, 2 aliquot test tubes, h, but the difference between them is small (0.06 mg for the Prime females and 0.11 mg for the Average females) relative to the larger differences between the females in the other competition treatments in this contrast); these range from 0.28 to 0.48 for the Prime females and from 0.29 to 0.64 for the Average females. This indicates that the aliquot treatment has a smaller effect on the growth of females when competition is most intense. If the females in the two most competition treatments are retaining food at an earlier point in their larval life, they would grow more slowly, pupate later and reach a smaller mass, and the difference between the Prime and Average female masses would be smaller across the two aliquot treatments.
12. The largest effect of the aliquot treatment across the competition treatments in this contrast is in the high food, high density treatments. The Prime females are 0.48 mg larger (10% of the largest Prime female mass) in the 4 aliquot treatment compared to the 2 aliquot treatment, and the Average females are 0.64 mg larger (14% of the largest Average female mass). For the low food, low density treatment, the Prime females are 0.28 mg larger (6%) in the 4 aliquot treatment and the Average females are 0.39 mg larger (8%). For the least competition treatment, the Prime females in the 4 aliquot treatment are 0.28 mg larger (6%) and the Average females are 0.29 mg larger (also 6%). The effect of aliquot on the Prime and Average female mass is much greater in the high food, high density treatments than in the other competition treatments.
13. In the high food, high density treatment the 4 aliquot treatment allows the females to grow almost as well as in the least competition treatment, while the 2 aliquot treatment causes the females to grow only as well as the low food, low density treatment.
14. Three or four aliquots of food reduce the effect of competition (food x density) on the growth of females as compared to one or two aliquots. This effect is greater for the Average females than for the Prime females. This suggests that the Prime female (or the larger females) dominates the initial food input (in the 2 aliquot treatment) and outcompetes the smaller females for the food.
15. The size difference between the Prime female and the Average female in each treatment is a measure of the relative advantage of the Prime female over the non-Prime females. Comparing the difference in size between the Prime female and the Average female across the 8 treatment combinations, the difference is always greater in the 2 aliquot treatment compared to the 4 aliquot treatment within each of the competition treatments (food x density). The Prime female has a greater advantage over the non-Prime females in the 2 aliquot treatment. Aliquot affects the competition among females independently of timespan.
16. Furthermore, the smallest effects of aliquot on the difference between the Prime female and the Average female are in the least competition treatments (0.12 mg and 0.13 mg). The Prime female dominates the competition; the Prime female grows largest and pupates earliest in the least competition treatments. The Average female also grows largest in the least competition treatments. Within the least competition treatment, the Prime female grows larger and delays pupation in the 4 aliquot treatment compared to the 2 aliquot treatment. The Average female also grows larger in the 4 aliquot treatment. At the highest food levels (total food and food/larva) competition among females is reduced resulting in the largest masses and least differences in size between the Prime and Average females. Despite the lower competition in these test tubes, the 4 aliquot treatment increases the size of both Prime and Average females.
17. The largest effect of aliquot on the difference between the Prime female and the Average female occurs in the high food, high density, 2 aliquot treatment (e, 0.41 mg difference), indicating the greatest dominance of the Prime female over the Average female. The differences between the Prime female and the Average female in the other treatments range from 0.15 mg to 0.26 mg. This suggests that the high total food may be affecting the competition among females, but the size of the Average female in e is the same as in f, so total food doesn’t explain this outcome by itself (e has twice the total food as f). A heuristic 3D plot of these differences is presented in S19 Fig.
18. This asymmetry in the impact of aliquot on competition is the likely source of this interaction. It appears to be different from the effect of aliquots seen previously in the FxDxAxT interaction for the Average female mass, where the large input of food on day 3 in the least competition, 2 aliquot treatment results in an apparent release of competition for the non-Prime females.
19. The initial food input in the test tube is greater for the 2 aliquot treatment (8 mg or 16 mg). This does not seem to affect competition since the 2 aliquot treatment produces smaller females than the 4 aliquot treatment at all levels of competition.
20. The second aliquot in the 4 aliquot treatment (on day 1 or day 2) brings the level of food in all test tubes up to the same level (8 mg or 16 mg). This doesn’t seem likely to explain the positive effect of the 4 aliquot treatment on the growth and competition of females.
21. The third aliquot (on day 2 or day 4) would increase the amount food in the test tubes with the 4 aliquot treatment, and thus could affect both competition and growth. The three treatment combinations that grow largest (Prime and Average females) and pupate earliest (Prime female) in this interaction all pupate before the addition of the final aliquot in the 6 day timespan, so this interaction seems to be about the third aliquot during the mid-larval period when females are actively growing. (This is the same time in the larval growth period that the food input affected the growth of non-Prime females in the FxDxAxT interaction above.)
22. The addition of food reduces competition among females and possibly changes the nature of competition from retaining food particles to actively filtering particles. At the lower food level, and especially in the test tubes with the most competition, the effect of the additional food due to the third aliquot is not as large as in the high food, high density treatment, probably because the third aliquot is also not as large in these test tubes.
23. The Prime female age at pupation is not significantly affected by this interaction, but the ages at pupation are affected by all three main effects (S4 Table MANOVA and S9 Table ANOVA). The mean ages range from 5.36 days to 8.95 days and correspond roughly to the Prime female masses. The three largest Prime females (a-c) pupate earliest, on day 5 or 6. The next, d and e, pupate mostly on day 6, with a minority pupating on day 7. The next largest, f, begins pupating on day 6, but the majority pupate on day 7. The Prime females in g pupate on day 7 with a minority pupating on day 8, and those in h pupate mostly on day 9.
24. The 4 aliquot treatments pupate earlier than the 2 aliquot treatments except for the least competition treatment. In those test tubes, the Prime females in the 4 aliquot treatment grow larger and pupate later, probably because there is more food from the third aliquot (day 2 and day 3 for the 3 day timespan, or day 4 for the 6 day timespan).
25. As a result of the differences between the Prime female mass and age at pupation across the treatments, the estimated growth rates are grouped differently from the masses: b and a are similar, then c, then d and e are similar, then f, then g and h. The difference between the order of the growth rates and the Prime female masses is the overlap between the high food, high density and low food, low density treatments. Across these four intermediate competition treatments, the high food, high density, 4 aliquot treatment (c) has the highest growth rate (0.76 mg/day), the low food, low density, 4 aliquot treatment (d) has the next highest (0.66 mg/day), which is almost the same as the high food, high density, 2 aliquot treatment (e) at 0.65 mg/day, followed by the low food, low density, 2 aliquot (f) at 0.56 mg/day. The high food, high density, 2 aliquot treatment (e) pupates slightly earlier and at a smaller size than the low food, low density, 4 aliquot treatment (d). A heuristic 3D plot of the estimated growth rates for the Prime females is presented in S20 Fig.
26. Analogous to the relationship between mass at pupation and the amount of food after day 4 (both total food and food/larva) for the interaction FxDxT, there is a relationship in this interaction between the mass at pupation for both Prime females and Average females and the amount of food after day 4. The mass at pupation follows the food/larva at the end of day 4, with the total food at the end of day 4 affecting the outcome where the food/larva is equal across treatments. This explains the order (a-h) for both mass variables. This relationship differs from that observed for the FxDxT interaction for Prime females where the total food was more important above 4 mg food/larva and food/larva was more important below 4 mg food/larva. In the FxDxA interaction all 8 treatments include the 3 day timespan and the 6 day timespan treatments, so only a and b (both least competition treatments) have food/larva levels 4 mg or greater for all of their replicates.

S25 Table. Means (SE) for Prime female mass and age at pupation and Average female mass at pupation for the interaction FxDxA. Estimated growth rate and difference between the Prime and Average female mass.

| Food x Density | Aliquot | Rank by Prime female mass (a-h) | Prime female mass at pupation (mg) | Prime female age at pupation (days) | Average female mass at pupation (mg) | Estimated Prime female growth rate (mg/day) | Prime female mass MINUS Average female mass (mg) |
| --- | --- | --- | --- | --- | --- | --- | --- |
| Low food, low density (4 mg/larva) | 2 aliquots | f | 3.86 (0.83) | 6.85 (1.35) | 3.60 (0.88) | 0.56 (0.37) | 0.26 (1.21) |
|  | 4 aliquots | d | 4.14 (0.37) | 6.24 (0.51) | 3.99 (0.42) | 0.66 (0.18) | 0.15 (0.56) |
| Most competition (2 mg/larva) | 2 aliquots | h | 2.86 (0.15) | 8.95 (2.33) | 2.63 (0.20) | 0.32 (0.25) | 0.23 (0.25) |
|  | 4 aliquots | g | 2.92 (0.17) | 7.28 (1.18) | 2.74 (0.13) | 0.40 (0.18) | 0.18 (0.21) |
| Least competition (8 mg/larva) | 2 aliquots | b | 4.53 (0.52) | 5.36 (0.20) | 4.40 (0.52) | 0.85 (0.22) | 0.13 (0.74) |
|  | 4 aliquots | a | 4.81 (0.08) | 5.71 (0.00) | 4.69 (0.04) | 0.84 (0.03) | 0.12 (0.09) |
| High food, high density (4 mg/larva) | 2 aliquots | e | 4.01 (0.66) | 6.20 (1.13) | 3.60 (0.85) | 0.65 (0.36) | 0.41 (1.08) |
|  | 4 aliquots | c | 4.49 (0.29) | 5.88 (0.35) | 4.24 (0.25) | 0.76 (0.15) | 0.25 (0.38) |

Multiple food inputs (the 4 aliquot treatment) result in larger sizes at pupation for Prime and Average females. Multiple food inputs appear to reduce competition among females, but have no effect on competition among males. Prime females and Average females grow larger on multiple food inputs even when the food/larva is the same, and the total food is lower in the 4 aliquot treatment than in the 2 aliquot treatment (comparing the Prime and Average females in d, the low food, low density, 4 aliquot treatment, with those in e, the high food, high density, 2 aliquot treatment). This anomaly, along with the pupation of some of the Prime females before the addition of the final aliquot on day 6, suggests that the total food and the food/larva on day 4 is more indicative of the appropriate food supply for these larvae. Food/larva is affected by the timespan treatment, so it is represented by two numbers in S26 Table, one for the 3 day timespan and the smaller one for the 6 day timespan. Food/larva describes the size order of the 8 treatments (a-h) for both the Prime and Average females, with the total food affecting the outcome when the food/larva is the same across treatments. In the comparison between the larger females in d, low food, low density, 4 aliquot treatment relative to e, the high food, high density, 2 aliquot treatment above, the first treatment has a higher food/larva than the second (3 mg, 4 mg, food/larva in the low food, low density, 4 aliquot treatment versus 2 mg, 4 mg, food/larva in the high food, high density, 2 aliquot treatment) which is more important than the total food in each treatment. Food/larva on day 4 is more important to the mass at pupation of females in this interaction. Early food inputs are more important than later ones for females (see the exception in FxDxAxT above).

S26 Table. Means (SE) for FxDxA for Prime female mass and age, and Average female mass. Total food and food/larva after day 4.

| Food x Density | Aliquot | Rank by Prime female mass (a-h) | Prime female mass at pupation (mg) | Prime female age at pupation (days) | Average female mass at pupation (mg) | Total food after day 4 (mg) | Food/larva after day 4 (mg) |
| --- | --- | --- | --- | --- | --- | --- | --- |
| Low food, low density (4 mg/larva) | 2 aliquots | f | 3.86 (0.83) | 6.85 (1.35) | 3.60 (0.88) | 12, 16 | 3, 4 |
|  | 4 aliquots | d | 4.14 (0.37) | 6.24 (0.51) | 3.99 (0.42) | 14, 16 | 3.5, 4 |
| Most competition (2 mg/larva) | 2 aliquots | h | 2.86 (0.15) | 8.95 (2.33) | 2.63 (0.20) | 12, 16 | 1.5, 2 |
|  | 4 aliquots | g | 2.92 (0.17) | 7.28 (1.18) | 2.74 (0.13) | 14, 16 | 1.75, 2 |
| Least competition (8 mg/larva) | 2 aliquots | b | 4.53 (0.52) | 5.36 (0.20) | 4.40 (0.52) | 24, 32 | 6, 8 |
|  | 4 aliquots | a | 4.81 (0.08) | 5.71 (0.00) | 4.69 (0.04) | 28, 32 | 7, 8 |
| High food, high density (4 mg/larva) | 2 aliquots | e | 4.01 (0.66) | 6.20 (1.13) | 3.60 (0.85) | 24, 32 | 3, 4 |
|  | 4 aliquots | c | 4.49 (0.29) | 5.88 (0.35) | 4.24 (0.25) | 28, 32 | 3.5, 4 |

Competition among females does not seem to be affected by the different initial inputs (day 0) or by the second input (on day 1 or day 2 depending on the timespan treatment), but rather by the third input (on day 2 or day 4 depending on the timespan treatment). The third aliquot of food changes the competitive environment among females, perhaps causing them to switch from retention to active filtering. This effect is more pronounced in the test tubes with the least competition and those with the higher food level, probably because the third aliquot is larger in these treatments. In contrast, the females in the most competition treatments are smaller and the Prime females grow slowest across the interaction. The Prime females in these test tubes are more similar in size (across the aliquot treatment) than the Prime females in the test tubes with the least competition and the intermediate levels of competition. The Average females in these test tubes are also closer in size (across the aliquot treatment) than the Average females in the test tubes with the least competition and the intermediate levels of competition. This suggests that at low food/larva levels females retain particles and differences in outcome are caused by differential rates of absorption rather than differential filtering ability. Large females probably have faster rates of absorption, but the difference is not as great as size differences and growth rates due to filtration ability.

**FxDxA summary**

The Prime and Average female masses are affected by competition and the aliquot treatment. The other dependent variables: the masses of males, the ages of the Prime males and females at pupation, and survival; are not affected by this interaction. Competition among females for food is different from that among males.

The 4 aliquot treatment allows females to grow larger than the 2 aliquot treatment for each level of competition. The difference between the aliquot treatments is likely the third aliquot that increases the food level during the larval growth period. The Prime female dominates competition among females. For each level of competition the Prime female is smaller and the difference between the Prime and Average female is greater in the test tubes with the 2 aliquot treatment compared to the 4 aliquot treatment. This is possibly explained by interference competition, but the largest difference is at the intermediate level of competition (e), so this is more likely explained by the Prime female switching to retention from active filtering and then switching back to active filtering on the subsequent inputs of yeast particles (the third and fourth aliquots). The Average females in the 2 aliquot treatments may experience a release from competition with the addition of the last aliquot on day 3 or day 6, but they do not grow as large as the Average females in the 4 aliquot treatments (for each level of competition). Late additions of food are not of equal value to the larvae as early food inputs. This may be due to constraints created during the molt to the fourth instar larva limiting either the growth in size directly or through the smaller sizes of feeding structures.

The opposite signs of the discriminant function coefficients for these two variables in this contrast in the MANOVA appears to be due to the relative sizes of the Prime female and the Average female. As the Prime female mass decreases (a-h), the difference between the Prime female and the Average female roughly increases.

Males are affected by competition with females in the interactions FxDxT (above) and FxD (below) as well as in other sources (see [1]). That they are not affected by competition with females in this interaction suggests that the third aliquot of food that is driving this effect among females is too late to affect the outcome of competition among males (see the FxDxAxT above). One possibility is that there is already surplus food at the higher food level for the males to grow and pupate, and that at lower food levels the females monopolize the smaller incremental food input so that males do not benefit. Another possibility is that males determine their final size before day 2 or day 4 and subsequent inputs of food don’t affect that target size. A third possibility is that the molt to the third or fourth instar limits the final size of the male pupa due to some physical constraint or some aspect of the feeding apparatus.

**The FxD interaction (R squared = 0.73)**

This interaction represents residual effects of competition on the seven dependent variables (the primary effects being in the higher level interactions, FxDxT, FxDxAxT and FxDxA, described previously). It is the third largest interaction in the MANOVA, but it is only significant for four of the dependent variables in the ANOVAs: Prime male age, Average male mass, Prime female age and Average female mass. For the mass and age at pupation variables, the main effects are: higher food level increases mass and decreases age, and higher density reduces mass and increases age. Prime male age, Average male mass, Prime female age and Average female mass have r squared values of 0.11, 0.01, 0.08, and 0.01, respectively. This contrast is much more important for the two age variables than for the two Average mass variables. The MANOVA coefficients for these 4 variables are large and positive; the MANOVA coefficient for Prime female mass is also large, but negative. Increased competition decreases the Average masses and also increases the Prime ages at pupation. Because this interaction affects the Average masses and not the Prime masses, competition is likely affecting the mass of the non-Prime individuals. Competition (food x density) appears to have a residual effect on the Prime male and female ages at pupation and the Average male and female masses at pupation that is independent of the aliquot or timespan treatments.

S27 Table shows the means and standard errors for the Prime female mass at pupation, the Prime female age at pupation and the Average female mass at pupation; the Prime female mass is included because it is useful to understand the effect on the other variables in relation to the Prime female mass (e.g. growth rate and the difference between the Prime and Average masses). The Prime female age and the Average female mass are presented together in the heuristic 3D plot in S21 Fig. This table presents two new columns: the expected mean values for Prime female age at pupation and the expected mean values for the Average female mass at pupation. These values are calculated by averaging the means for the main effects for each variable. The standard error is the standard error for each variable across the 16 cells. This is a heuristic comparison to indicate which cells in the 2x2 comparison deviate most and least from the combined main effects. There is no statistical test implied.

1. The earliest Prime female age at pupation is 5.54 days in the test tubes with the least competition. The latest Prime female age is 8.11 days in the test tubes with the most competition. The two intermediate competition treatments (6.04 days for the high food, high density, and 6.54 days for the low food, low density) are more similar to the least competition treatment than to the most competition treatment. The Prime female age at pupation is disproportionately late in the test tubes with the most competition compared to the other treatments. This is likely the source of this interaction.
2. The largest Average female mass is 4.54 mg in the test tubes with the least competition. The smallest Average female mass is 2.68 mg in the test tubes with the most competition. The two intermediate competition treatments (3.92 mg for the high food, high density, and 3.79 mg for the low food, low density) are more similar to the least competition treatment than to the most competition treatment. The Average female mass is disproportionately small in the test tubes with the most competition compared to the other treatments. This is likely the source of this interaction.
3. The two columns, “Expected mean values for Prime female age at pupation” and “Expected mean values for Average female mass at pupation,” represent the expected means based on the main effects for this interaction. For the Prime female age at pupation, the least competition treatment pupates almost a half day earlier than expected, but the most competition pupates almost a full day later. The two intermediate competition treatments also pupate earlier than the projections based on the main effects.
4. Similarly, for the Average female mass, the size of the Average females in the least competition test tubes is 0.34 mg larger than expected, but the size of the Average females in the most competition test tubes is 0.59 mg smaller. The two intermediate competition treatments are also larger than the projections.
5. This 2-way interaction is significant because the growth of larvae in the most competition treatment is much worse than predicted based on the main effects for both Prime female age and Average female mass. The most competition treatment appears to have even less food than expected for these two variables. The Prime female age at pupation is affected by this interaction 8 times more than the Average female mass (r squared values).
6. Competition increases from the least competition test tubes, to the intermediate competition test tubes, to the most competition test tubes. The factors, food and density, double, from 16 mg to 32 mg of food, and from 4 larvae to 8 larvae per test tube, but the age at pupation increases by a half day from least competition to high food, high density, then by another half day from high food, high density to low food, low density, then by just over a day and a half to most competition. The increase in competition has a disproportionate effect on age at pupation in the most competition test tubes. The larvae in the least competition test tubes have the highest total food (32 mg of food) and the highest food/larva (8 mg food/larva). Those in the high food, high density test tubes also have the highest total food (32 mg of food) and 4 mg food/larva. The larvae in the low food, low density test tubes have 16 mg total food and 4 mg food/larva. Those in the most competition test tubes have 16 mg total food and 2 mg food/larva. In the FxDxT interaction above, the 4 mg food/larva level marked a change in the way that Prime and Average females (masses) responded to the two aspects of the food supply (total food and food/larva). In this interaction Prime female age increases by half a day for each increase in competition until the food/larva falls below 4 mg, and the Prime female age increases by a day and a half. The pattern is similar for the Average female mass; the Average females are disproportionately smaller in the most competition treatment.
7. The Prime female mass is not significantly affected by this interaction in the ANOVA, so the values of the Prime female mass are consistent with the main effects, food and density. The estimated growth rate combines the Prime female mass with the Prime female age in a biologically meaningful number. The greatest growth rate is in the least competition treatment (0.84 mg/day) and the smallest is in the most competition treatment (0.36 mg/day). The growth rates for the two treatments with intermediate levels of competition have growth rates closer to the least competition treatment than to the most competition treatment. This is consistent with the results from the age at pupation alone.
8. The difference between the Prime female mass and the Average female mass indicates the relative advantage of the Prime female over the non-Prime females. The smallest difference is in the least competition treatment (0.13 mg); however, the largest difference is not in the most competition treatment, but in the high food, high density treatment (0.33 mg). The low food, low density treatment and the most competition treatment are intermediate, both at 0.21 mg difference.
9. Competition among females in the test tubes with the least competition results in large Prime females and large Average females. The increased competition in the test tubes with high food and high density (4 additional larvae per test tube) results in smaller Prime females (0.42 mg smaller) and much smaller Average females (0.62 mg smaller). Comparing the low food, low density treatment to the high food, high density treatment (16 mg food per test tube versus 32 mg food, but 4 mg food/larva in both treatments), the Prime and Average females are both smaller in the low food, low density treatment, but the difference between them is also smaller. There is a large difference in size between the low food, low density treatment and the most competition treatment for both Prime and Average females (1.11 mg for both), but the difference between the Prime and Average females is 0.21 mg in each case.
10. Because the Prime female mass is not significantly affected by this interaction, the non-Prime females are growing larger than expected in the least competition treatment and not growing as large as expected in the high food, high density treatments. Density has an effect on competition beyond total food and food/larva. The additional 4 larvae in the test tubes between the low food, low density treatment and the high food, high density treatment increase the effect of competition on the non-Prime females and reduce their mass at pupation. The same increase in density from low food, low density to most competition does not produce the same decrease in Average female mass. The relative advantage of the Prime female over the non-Prime female is greater at the higher food level only. It is likely that at the lower food levels, the Prime females switch from filtering particles and passing them rapidly through their guts to retaining food earlier than at higher food levels, resulting in reduced growth and a compressed distribution of sizes. In the least competition treatment females grow as though food were unlimited, while in the high food, high density treatment females compete exploitatively for longer and establish a broader size distribution. The effect of this is on the non-Prime females only, and only shows up in the Average female mass.

S27 Table. Means (SE) for FxD for Prime female mass and age at pupation and Average female mass. Expected mean values for Prime female age and Average female mass.

| Food x Density | Prime female mass at pupation (mg) | Prime female age at pupation (days) | Average female mass at pupation (mg) | Estimated Prime female growth rate (mg/day) | Prime female mass MINUS Average female mass (mg) | Expected mean values for Prime female age at pupation (days) | Expected mean values for Average female mass at pupation (mg) |
| --- | --- | --- | --- | --- | --- | --- | --- |
| Low food, low density (4 mg/larva) | 4.00 (0.55) | 6.54 (0.90) | 3.79 (0.61) | 0.61 (0.32) | 0.21 (0.41) | 6.69 (1.38) | 3.71 (0.81) |
| Most competition (2 mg/larva) | 2.89 (0.14) | 8.11 (1.79) | 2.68 (0.15) | 0.36 (0.32) | 0.21 (0.10) | 7.21 (1.38) | 3.27 (0.81) |
| Least competition (8 mg/larva) | 4.67 (0.34) | 5.54 (0.23) | 4.54 (0.34) | 0.84 (0.17) | 0.13 (0.24) | 5.92 (1.38) | 4.20 (0.81) |
| High food, high density (4 mg/larva) | 4.25 (0.50) | 6.04 (0.71) | 3.92 (0.63) | 0.70 (0.31) | 0.33 (0.40) | 6.44 (1.38) | 3.77 (0.81) |

The FxD interaction shows a residual effect on competition among female larvae after accounting for the higher order interactions (FxDxT, FxDxA, FxDxAxT). The most significant effect is the delay in the Prime female age at pupation in the test tubes with the most competition. The mass of the Prime female is affected by the main effects of food and density, but not by the interaction between food and density. The age of the Prime female increases disproportionately with increasing competition, so the Prime female grows to a size dependent on the main effects of food and density, but takes longer to reach that size and pupate as competition increases. There is a similar effect on the mass at pupation for the non-Prime females in the most competition treatment. Since the mass of the Prime female is not affected by the interaction, and the mass of the Average female is affected, the masses of the non-Prime females across these treatment combinations must be the reason for the interaction on the mass of the Average female. The non-Prime females in the most competition treatment are disproportionately smaller than their counterparts in the other treatments. There is also an effect on the relative advantage of Prime females over the non-Prime females in the high food, high density, intermediate competition treatment. The size of the Prime female is reduced relative to the least competition treatment, but the non-Prime females are even smaller, indicating that the increased density/competition primarily affects the non-Prime females. This anomaly is likely due to the different competitive mechanisms (active filtering versus retention) at different levels of total food per test tube.

S28 Table shows the means and standard errors for the Prime male mass at pupation, the Prime male age at pupation and the Average male mass at pupation. Only the Prime male age and the Average male mass were significantly affected by this contrast in the ANOVAs; the Prime male mass is included because it is useful to understand the effect of the interaction on the other variables. This contrast explains 11 times as much variance for the Prime male age as it does for the Average male mass (r squared values). The expectation is that the males will grow best (largest size, earliest pupation) in the test tubes with the least competition, and do worst (smallest size, latest pupation) in the test tubes with the most competition. A heuristic 3D plot of the means for these two variables are presented in S22 Fig.

1. The highest value (5.40 days) of the Prime male age occurs in the test tubes with the most competition. The lowest value (5.00 days) is in the test tubes with high food and high density (intermediate competition), but the other two treatments are almost the same (5.04 days and 5.06 days for least competition and low food, low density, respectively). The disproportionately late pupation of Prime males in the most competition treatment is likely a source of the interaction for Prime male age.
2. The highest value (2.67 mg) of the Average male mass is in the test tubes with the least competition. The lowest value (1.82 mg) is in the test tubes with the most competition. The Average male masses in the test tubes with intermediate levels of competition are also intermediate (2.39 mg and 2.40 mg for high food, high density, and low food, low density, respectively), but closer to the least competition test tubes than to the most competition ones. The disproportionately small size of the Average males in the test tubes with the most competition is likely the source of the interaction for Average male mass.
3. The column, “Expected mean values for Prime male age at pupation,” represents the mean values based on the main effects of food and density. Unlike the case for Prime female age at pupation, the expected mean Prime male ages do not correspond to the magnitude or order of the observed means. The Prime males in the high food, high density treatment pupate earliest, while the main effects project that the Prime males in the least competition treatment should pupate earliest. This is likely one of the asymmetries that causes this interaction for Prime male age.
4. Another asymmetry is that the observed Prime male age at pupation in the most competition treatment is later (0.18 day) than the projected age at pupation, while the other three treatments are earlier or the same as the projection. (This asymmetry is similar to the one observed for Prime female age at pupation.)
5. These asymmetries likely cause the interaction and the high r squared value for Prime male age at pupation.
6. The column, “Expected mean values for Average male mass at pupation,” represents the mean values based on the main effects of food and density. The Average male mass is similar to the Average female mass; the least competition test tubes produce larger than expected Average males and the most competition test tubes produce much smaller than expected Average males. The intermediate competition treatments also produce larger than expected Average males.
7. The most competition treatment is worse than expected for both Prime male age and Average male mass, while the other treatments are better than expected.
8. Prime male mass is affected by both food and density, but not by the interaction between food and density. The estimated growth rates for the treatments: least competition; high food, high density; and low food, low density; are similar despite the differences in age at pupation, but the estimated growth rate for the Prime males in the most competition treatment is much lower reflecting the smaller mass and the later pupation.
9. The least competition treatment supports the fastest growth rate across the four treatments. These Prime males delay their pupation slightly (to 5.04 days) to grow larger (2.74 mg), but still attain the fastest growth rate (0.54 mg/day). The Prime males in the high food, high density treatment have the second highest growth rate (0.51 mg/day). They grow almost as large (2.54 mg) and pupate slightly earlier (5.00 days). The Prime males in the low food, low density treatment are smaller (2.44 mg) and pupate later (5.06 days) and attain a growth rate of 0.48 mg/day. The Prime males in the most competition treatment are smallest (1.81 mg), latest to pupate (5.40 days) and have the lowest growth rate (0.34 mg/day).
10. Prime males pupate earliest in the high food, high density treatment combination, but they do not grow to the largest size or attain the fastest estimated growth rate. Prime males may extend their larval period slightly in the least competition test tubes (more food/larva); they grow largest and have the highest growth rate in these test tubes, and this is expected to be the most favorable competitive environment for males and females. Prime males also appear to extend their larval period in the low food, low density test tubes (same food/larva as the high food, high density treatment, but less total food), but they do not grow as large or as fast as in the high food, high density treatment, so this does not appear to be taking advantage of abundant food, but rather extending the larval period to grow incrementally larger. Prime males in the test tubes with the most competition are smallest and have the lowest estimated growth rate in addition to the latest age at pupation. Prime males may extend their larval period to grow larger under favorable food conditions and also under unfavorable food conditions.
11. The Average male mass is largest in the test tubes with least competition and smallest in those with the most competition. The Average male masses in the test tubes with intermediate levels of competition are similar to each other and closer to that in the least competition treatment than to the most competition treatment. The Prime male mass at pupation is not affected by this interaction while the Average male mass is affected. This means that the non-Prime male masses are causing this interaction.
12. The difference between the Prime male mass and the Average male mass indicates the relative advantage of the Prime male over the non-Prime males. There are two quantitative differences between the Prime male mass and the Average male mass: first, the Average male mass is larger than the Prime male mass in the most competition treatment (1.82 mg for the Average male, 1.81 for the Prime male); second, the Prime male mass is higher in the high food, high density treatment (2.54 mg) than in the low food, low density treatment (2.44 mg), but the Average male mass is slightly lower in the high food, high density treatment (2.39 mg) than in the low food, low density treatment (2.40 mg).
13. The column “Prime male mass MINUS Average male mass” (S26 Table) shows the relative advantage of the Prime male. In the test tubes with the least competition, where the Prime male growth rate was highest, the Prime and Average males both grow largest and the difference between them is 0.07 mg.
14. Comparing these numbers to those of the test tubes with the most competition, the Prime male growth rate is the lowest, and both Prime and Average males are smallest, but the Average male is larger than the Prime male. The Prime male is the largest of the males that pupate earliest, so at least one of the non-Prime males benefits from the reduction in competition after the Prime male pupates and grows larger than the Prime male. (The FxDxT interaction shows a clear effect of the incremental food added on the 6th day on the growth of the non-Prime males. Since this interaction does not involve timespan, this is likely an effect of the reduction in competition due to the pupation of the Prime male. Comparing the r squared value for the FxDxT interaction, 0.16, to this one, 0.01, it seems clear that the effect of incremental food due to the timespan treatment is 16 times larger than the effect of release from competition due to the pupation of the Prime male.)
15. The second comparison involves the two intermediate levels of competition. The relative advantage of the Prime male over the Average male is twice as big for the high food, high density treatment (0.15) as for the least competition treatment (0.07 mg). On the other hand, the relative advantage of the Prime male is half as big for the low food, low density treatment (0.04) as for the least competition treatment (0.07). In the high food, high density test tubes there is the same amount of food as in the least competition ones, but 4 more larvae. Competition among the larvae is more intense and this appears as a reduction in the amount of food for the non-Prime males. At the higher food level the nature of competition is exploitative (active filtering and rapid passing of the particles through the gut by the female larvae) and the distribution of sizes is broad. In the low food, low density test tubes, the total amount of food is half of that in the high food treatments, but the lower density means that the food/larva is the same across the two intermediate competition treatments (4 mg/larva). In this case, the lower number of particles causes the females to switch to retaining particles in their guts, further reducing the number of particles available for the males and compressing the size distribution. The Prime male is reduced in size and the Average male mass is similar to that in the high food, high density treatment, so the non-Prime males do relatively better in the low food, low density treatment than in the high food, high density one (both relative to the Prime male in the low food, low density treatment and to the non-Prime males in the high food, high density treatment). This may also be due to the release from competition after the Prime male pupates.

S28 Table. Means (SE) for Prime male mass and age at pupation and Average male mass at pupation for the interaction FxD.

| Food x Density | Prime male mass at pupation (mg) | Prime male age at pupation (days) | Average male mass at pupation (mg) | Estimated Prime male growth rate (mg/day) | Prime male MINUS Average male mass (mg) | Expected mean values for Prime male age at pupation (days) | Expected mean values for Average male mass at pupation (mg) |
| --- | --- | --- | --- | --- | --- | --- | --- |
| Low food, low density (4 mg/larva) | 2.44 (0.31) | 5.06 (0.07) | 2.40 (0.35) | 0.48 (0.08) | 0.04 (0.23) | 5.14 (0.24) | 2.32 (0.39) |
| Most competition (2 mg/larva) | 1.81 (0.40) | 5.40 (0.39) | 1.82 (0.23) | 0.34 (0.09) | -0.01 (0.23) | 5.22 (0.24) | 2.11 (0.39) |
| Least competition (8 mg/larva) | 2.74 (0.14) | 5.04 (0.07) | 2.67 (0.16) | 0.54 (0.04) | 0.07 (0.11) | 5.04 (0.24) | 2.53 (0.39) |
| High food, high density (4 mg/larva) | 2.54 (0.27) | 5.00 (0.00) | 2.39 (0.25) | 0.51 (0.07) | 0.15 (0.18) | 5.11 (0.24) | 2.32 (0.39) |

The FxD interaction shows a residual effect on competition among male larvae after accounting for the higher order interaction (only FxDxT). The most significant effect is the delay in the Prime male age at pupation in the test tubes with the most competition. The mass of the Prime male is affected by the main effects of food and density, and the FxDxT interaction, but not by the interaction between food and density. The age of the Prime male increases disproportionately with increasing competition, so the Prime male grows to a size dependent on the main effects of food and density, but takes longer to reach that size and pupate as competition increases. There is a similar effect on the mass at pupation for the non-Prime males in the most competition treatment. Since the mass of the Prime male is not affected by the interaction, and the mass of the Average male is affected, the masses of the non-Prime males across these treatment combinations must be the reason for this interaction for the Average male mass. The non-Prime males in the most competition treatment are disproportionately smaller than their counterparts in the other treatments, but they are larger than the Prime male in the most competition treatment. This is likely due to the competitive release of non-Prime males after the Prime male pupates. There is also an effect on the relative advantage of Prime males over the non-Prime males in the high food, high density, intermediate competition treatment. The size of the Prime male is reduced relative to the least competition treatment, but the non-Prime males are even smaller, indicating that the increased density/competition primarily affects the non-Prime males.

**Males versus females**

1. Neither the Prime female mass nor the Prime male mass is affected by the FxD interaction, but the Prime female age at pupation and the Prime male age at pupation are affected. For both Prime individuals, an increase in competition delays pupation disproportionately, but the mass at pupation is determined by the independent factors, food and density, rather than the interaction between food and density. This interaction explains 8% of the variability for the Prime female age and 11% of the variability for the Prime male age across the experiment (the second largest interaction r squared value for the Prime female age and the third largest for the Prime male age).
2. Females dominate competition for food in the test tubes, and the Prime male outcompetes the non-Prime males, so the Average male mass represents the outcome for the least competitive individuals. The Average males in both the low food treatments grow larger than expected compared to the Prime male masses. At low food levels, Prime males pupate and this event either increases the available particles for the non-Prime males, or reduces competition, or both. This appears to be one source of the interaction for the Average male mass at pupation.
3. The difference between the Average female mass and the Average male mass across this interaction decreases as competition increases. The Average female is largest in the least competition treatment. The Average male is also largest in this treatment, and the difference between the two masses is largest (1.87 mg). The Average female in the high food, high density treatment is next in size. The Average female in the low food, low density treatment is slightly smaller. The Average males in both these intermediate competition treatments are about the same size; the difference between the Average females and the Average males corresponds to the Average female size (1.53 mg and 1.39 mg). The Average female is smallest in the most competition treatment, as is the Average male. The difference between the two is also smallest (0.86 mg). The non-Prime males grow larger than the Prime male in this treatment apparently experiencing a release from competition when the Prime male pupates. Non-Prime females do not appear to benefit similarly.
4. Males pupate earlier and at a smaller size than females, and the compressed distribution of sizes of Prime males across treatments suggests that they reach a maximum size (depending on food and density and other factors) and pupate. The Average males across this interaction pupate at masses from 1.82 mg to 2.67 mg (0.85 mg range), while the Average females pupate at masses from 2.68 mg to 4.54 mg (1.86 mg range, more than double the range of male masses). Furthermore, the size of the Average male as a percent of the Average female increases as competition increases (59%, 61%, 63% and 68% corresponding to decreasing Average female mass in S25 Table). Females are larger than males within each treatment and dominate competition, but in the most competition treatment males, especially the non-Prime males, grow relatively larger than the non-Prime females.

This is one of the most consequential interactions for Prime female age at pupation and Prime male age at pupation. Both Prime individuals grow to a size determined by the factors food and density, and by other interactions, but the age at pupation is delayed by increasing competition. This effect of competition is independent of the two other factors, aliquot and timespan.

This interaction also describes the competition among the non-Prime males and females (see also the FxDxAxT interaction). Non-Prime males and non-Prime females are disproportionately affected by the increase in density and competition in the high food, high density treatment (relative to the least competition treatment). The Prime individuals have the largest advantage over the Average individuals in these test tubes. The non-Prime females are also disproportionately smaller in the most competition treatment, probably reflecting a switch in feeding behavior among the females from active filtering to retention. In contrast, the non-Prime males in both the low food treatments (low food, low density and most competition) grow larger than expected. This probably reflects a release from competition after the Prime male pupates, but may also reflect the compressed size distribution of males at pupation. There may be two alternative paths for males, either to grow as fast as possible to a target size and pupate as early as possible, or to grow to a larger size in response to late additions of food and pupate as a larger adult.

FxD summary. For the Prime female age at pupation and the Average female mass, the test tubes with the most competition produce the worst outcomes, disproportionately worse than the other three treatments and also worse than projected based on the main effects. Greater competitive intensity looks like a reduced food level. The effect of the interaction on Prime female growth rate is consistent with the effect on age at pupation alone. The difference between the Prime and Average female masses suggests that competition in the high food, high density test tubes is qualitatively different from that in the other treatments. In the least competition test tubes, growth appears to be optimal for this interaction. In the two treatments at the lower food level, competition appears to result in smaller sizes and a similar (smaller) size distribution. In the high food, high density treatment, the Prime female grows larger, but the non-Prime females are smaller than in the low food, low density treatment (same food/larva, but less total food). This is suggestive of different competitive mechanisms among the females in the different treatments (actively filtering particles and passing them rapidly through the gut versus retaining the particles within the gut to extract more nutrients on each transit). The active filtering at the higher total food level results in larger Prime females and smaller non-Prime females compared to retention at the same food/larva level.

For the Prime male age at pupation, there are multiple asymmetries that probably cause the interaction. Prime males pupate at a disproportionately late age in the test tubes with the most competition, and this is also later than expected based on the main effects. However, the Prime males in the high food, high density test tubes pupate earliest; the main effects projected the Prime males in the least competition test tubes to pupate earliest. The effect of the interaction on the Prime male estimated growth rate differs from that of the Prime female above. The least competition treatment supports the fastest growth rate and the largest Prime mass at pupation, but not the earliest age at pupation. These Prime males delay their pupation perhaps in response to abundant food and grow larger than those in the high food, high density treatment (second fastest growth rate and earliest age at pupation). The Prime males in the low food, low density treatment also delay their pupation, but they are smaller than the high food, high density Prime males and also have a slower growth rate. The Prime males in the most competition treatment are smallest, latest to pupate and have the lowest growth rate. The Prime males pupate in a small window of time, but delay pupation in response to abundant food, and to competition at lower food levels (retention by females). Prime males pupate earliest in those test tubes where the competitive mechanism among females is predominately active filtering. Prime males respond to food and density (and competition with females) differently than Prime females. The Prime female pupates earliest in the test tubes with the least competition; the Prime male delays pupation in response to abundant food in those test tubes. The Prime females’ estimated growth rates reflect their ages at pupation; the Prime males’ estimated growth rates reflect their masses rather than their ages at pupation. However, the Prime males pupate in a much tighter window of time than the Prime females, so there is more variability in the masses than in the ages at pupation. Prime males pupate earliest in the test tubes where the Prime females appear to dominate the non-Prime females to the greatest degree, the high food, high density treatment. The more intense competition among females in these test tubes compared to the least competition test tubes appears to cause the Prime male to pupate sooner and at a smaller size.

The Average male mass resembles the Average female mass; the test tubes with the most competition produce the worst outcomes, disproportionately worse than the other three treatments and also worse than projected based on the main effects. Also similar to the Average females, because the Prime male mass is not significant for this interaction, the affected individuals are the non-Prime males. There are two quantitative differences in the relationship between the Prime male mass and the Average male mass that are different from the females. First, the Average male mass is greater than the Prime male mass in the most competition treatment; at least one non-Prime male grows larger than the Prime male after the Prime male pupates. Second, the Prime male mass is larger in the high food, high density treatment than in the low food, low density treatment (same food/larva, but more total food), but the Average male mass is larger in the low food, low density treatment. In the high food, high density test tubes there is the same amount of food as in the least competition ones, but 4 more larvae. Competition among the larvae is more intense and this appears as a reduction in the amount of food. Because there is a high food level the nature of competition among the females is active filtering and rapid passing of the particles through the gut, the distribution of sizes is broad and the relative advantage of the Prime male over the non-Prime males is large. In the low food, low density test tubes, the total amount of food is half of that in the high food treatments, but the lower density means that the food/larva is the same across the two intermediate competition treatments (4 mg/larva). In this case, the lower number of particles causes the females to switch to retaining particles in their guts, further reducing the number of particles available for the males and compressing the size distribution. The Prime male is reduced in size and the Average male mass is similar to that in the high food, high density treatment, so the non-Prime males do better in the low food, low density treatment than in the high food, high density one (both relative to their Prime male and absolutely against the non-Prime males in other treatments). The Average male mass is larger than expected at both low food treatments. The non-Prime males grow larger after the Prime male pupates in the treatments where the females are retaining food because of the low number of particles. The Prime male pupates, releasing food particles and reducing competition; this benefits the non-Prime males and allows them to grow larger. Timespan and aliquot are not involved in this interaction, so this effect (competitive release) is not affected by those factors.

The Prime males and females pupate in response to different cues in the test tubes. Prime females respond to food, density and competition (the interaction between food and density). Prime females grow best in the test tubes with the least competition and switch from active filtering to retention at the lower food level (two treatments). Prime males respond to the main factors and the interaction as well, but also to the nature of the competition among females. Prime males pupate earliest in the test tubes with the high food, high density treatment where the females are actively filtering, but extend their larval period in the test tubes where the females appear to have abundant food. The males also extend the larval period in the test tubes at the two low food treatments where the females are retaining particles.

The Average male and female masses represent the effect of this interaction on the non-Prime males and females. The Prime females dominate the competition in these test tubes; they are larger (better at filtering) and switch from active filtering to retaining particles in their guts in response to lower food levels. In the test tubes with the least competition, both Prime and Average females grow largest. In the high food, high density test tubes, the difference between the Prime and Average females is greatest, indicating that the active filtering competitive mechanism creates a broad size distribution. The non-Prime females do relatively and absolutely less well compared to those in the least competition test tubes and compared to those in the low food, low density test tubes. The higher density compared to the least competition test tubes increases competition for particles. This reduces the size of the Prime female, but affects the non-Prime females to a larger degree. The different competitive mechanism at the lower food level (retention) also reduces the size of the Prime female, but does not reduce the size of the non-Prime females as much as in the high food, high density test tubes. There is a similar effect on the non-Prime males. Males compete more intensely with each other and are affected by the competition with females (but females are unaffected by competition with males, see [1]). In addition to the larger size distribution of males in the high food, high density test tubes, the non-Prime males in the two low food level treatments grow larger than expected. They experience a release from competition after the Prime male pupates. The difference between the Average female mass and the Average male mass is smallest in the most competition treatment where the non-Prime males grow larger than the Prime male. The difference is also small in the low food, low density treatment where the non-Prime males grow larger than the non-Prime males in the high food, high density treatment. At the high food levels, both the Average masses increase in size, but the Average female mass is increasingly larger than the Average male mass. The size advantage of larger females actively filtering and outcompeting smaller females also means that they are outcompeting the males.

**Interactions involving competition (FxD set of interactions)**

Four interactions describe competition among mosquito larvae in this experiment: FxDxT; FxDxAxT; FxDxA; and FxD. FxDxT, FxDxA, and FxD are all significant in the MANOVA; the 4-way interaction, FxDxAxT, is not significant in the MANOVA. FxDxT is significant in the ANOVAs for each of the seven dependent variables. FxDxAxT is significant only for the ANOVA for Average female mass. FxDxA is significant for the Prime female mass and the Average female mass. FxD is significant for Prime male and female ages at pupation and for Average male and female masses. Together these interactions describe a complex relationship between the factors: food, density, aliquot and timespan; and the survival, growth and pupation of male and female mosquito larvae. These interactions cross food (total food per test tube) with density (4 or 8 larvae per test tube) to compare the outcomes across different competitive environments.

Survival is only affected by the FxDxT interaction (among these four). Survival is lower in the most competition treatments. Survival is higher with the 6 day timespan treatment than with the 3 day timespan treatment except in the most competition treatment, where the longer timespan may increase the food stress. Survival appears to be better when the food additions are spread out in time. (See also the results of the third experiment.)

The four mass variables decrease with increasing competition and the two age variables increase with increasing competition, so they are frequently negatively correlated in the MANOVA. The Prime female dominates competition in the test tubes and affects the size of the Average females and the size of the males as well as the age at pupation of the Prime male. The Prime female mass is significant in the FxDxT and FxDxA interactions (in the ANOVA). The Prime female age is significant in the FxDxT and FxD interactions. The Average female mass is significant in all four interactions. When the Average female mass is significantly affected by a contrast, but the Prime female mass is not affected, the presumption is that the interaction reflects competition by the non-Prime females (FxDxAxT and FxD).

The Prime male dominates competition among the male larvae. The Prime male mass is significant only in the FxDxT interaction. The Prime male age is significant in the FxDxT and FxD interactions. The Average male mass is significant in the FxDxT and FxD interactions as well. When the Average male mass is significantly affected by a contrast, but the Prime male mass is not affected, the presumption is that the interaction reflects competition by the non-Prime males (FxD). This is also the case when the Average male mass is larger than the Prime male mass (the non-Prime males grow larger than the Prime male).

Prime females: The interaction FxDxT shows that the Prime female mass at pupation follows the total food level (on day 4 of the experiment) when the food/larva is 4 mg or greater, but follows the food/larva level at lower levels. This is probably related to the mechanism of feeding (active filtering versus retention) associated with the abundance or scarcity of food particles. Late additions of food do not have the same value as equal earlier inputs. The Prime female age at pupation follows the total food level across all treatments. In the interaction FxDxA, the Prime female mass follows the food/larva (on day 4 of the experiment) across all treatments (only the two least competition treatments have food/larva levels that are all greater than 4 mg, and the total food levels are in the same order as the food/larva for these two treatments). Late additions of food do not have the same value as equal earlier inputs in this interaction either. The Prime female age at pupation is not affected by the FxDxA interaction. In the FxDxA interaction, the third aliquot of food (only in the 4 aliquot treatment) appears to positively affect the mass of the Prime females. The Prime female mass is not affected by the FxD interaction, but the Prime female age is affected. Prime females pupate at masses determined by other factors, but competition (FxD) affects how long they take to pupate. For the Prime female, the timespan treatment reveals that competition changes in response to the highest levels of total food at high levels of food/larva, but that competition is different at lower levels of food/larva. The timing of pupation is related to the total food at all levels of food/larva. The aliquot treatment reveals that multiple inputs of food are beneficial and that the third aliquot results in additional growth, probably by providing more food at a critical time in the larval lifespan. The FxD interaction shows that competition affects the timing of pupation while other factors (and other interactions) affect the mass at pupation. The mass at pupation of the Prime female is determined by one set of environmental parameters, and the age at pupation is determined by another set, which partially overlaps the first set. Mass and age at pupation are partially independent, but both respond to some of the same environmental cues (e.g. food/larva). When these cues change during the larval life, early changes have a greater affect than later changes. This could be because of biochemical and physiological characteristics of the larvae and/or physical characteristics such as the size of the head capsule or the feeding apparatus.

Average females: The interaction FxDxT shows that the Average female mass at pupation follows the food/larva level (on day 4) across all treatments. Average females do not appear to benefit from the late addition of food on day 6 or any release from competition after the pupation of the Prime female. The Prime female controls the food supply and benefits exclusively from the high total food, while the non-Prime females grow according to the food/larva level. The interaction FxDxAxT reveals that a large input of food on day 3 effectively eliminates competition among females for a period of time, allowing the non-Prime females to grow larger relative to non-Prime females that received equivalent food earlier in the life cycle. The specific timing of this food input makes food particles available when the Prime female would be switching from active filtering to retention, and allows the non-Prime females to grow larger (increasing the Average female mass). The FxDxA interaction reveals that multiple food inputs result in larger masses for Average females. Food/larva (on day 4) describes the size order of the mass at pupation of the Average females. Early food inputs are more important than later ones for the Average females (but see FxDxAxT above). The FxD interaction reveals that the masses of the non-Prime females are affected by competition after the higher order interactions are removed. The effect of food and density jointly on the masses of the non-Prime females is independent of the two other factors: aliquot and timespan. Non-Prime females grow similarly to their Prime females in all treatments. The exception to this is the large input of food on day 3 in the FxDxAxT interaction which allows non-Prime females to grow larger than their peers in test tubes with the same amount of food, but delivered in multiple aliquots, earlier. Non-Prime females are also affected by competition after the higher order interactions are removed. Only the age at pupation of Prime females is affected by the residual FxD interaction.

The Prime female dominates competition in the test tube and solely benefits from the high total food level. The non-Prime females grow in response to the food/larva, although they can benefit from a large input of food (on day 3 in this experiment) that coincides with the switch from active filtering to retention by the Prime female as the relative abundance of particles decreases. Food is probably unlimited for 1^st^ instar larvae and similarly abundant for 2^nd^ instar larvae. As the larvae molt from the 2^nd^ instar to the 3^rd^ instar, their demand for particles increases and their ability to filter particles increases, reducing the availability of particles. During the 3^rd^ or 4^th^ instar, the demand for particles exceeds the availability and the females switch from actively filtering particles and passing them rapidly through their guts to retaining the particles to extract more nutrients. This is further exacerbated because the quality of the particles decreases over time as well. An input of food in the 3^rd^ instar would offset the relative decrease in particles and allow the females to continue to actively filter. Multiple such inputs would result in larger females by reducing competition as well as increasing food availability. The timing of a food addition in the third instar may offset multiple earlier inputs (of equal food) because the non-Prime females escape competition at the time when the Prime female would normally switch to retention, reducing the growth rate of all the females and reducing their eventual mass at pupation. Individual females grow as fast as possible in response to environmental conditions. As 1^st^ instars, food may be unlimited, but the initial size of the larva (or egg size, or some other uncontrolled factor in the experiment) and chance determine how fast the larva grows. At some point, this larva molts to the 2^nd^ instar. The larva may delay molting in order to grow larger because food is available, but there is also an advantage to molting in order to use the larger feeding apparatus of the 2^nd^ instar. This trade off between growing larger and molting should also be true for the 3^rd^ and 4^th^ instar molts. Other trade offs may be important for the timing of pupation. In this experiment, the total food and food/larva in the test tubes change depending on the aliquot and timespan treatments. Both total food and food/larva affect competition among females; timespan is more important than aliquot, and their effects are independent (with the exception of non-Prime females in the FxDxAxT interaction). It appears that the environmental parameters on day 4 of the larval lifespan are the most important to determining the final size and age at pupation of females, although at lower total food levels females do not pupate until there is 16 mg total food in their test tubes (after the final food input for the 6 day timespan treatment).

Prime males: Males are affected by competition with females (but females are not affected by the males). The Prime male benefits from reduced competition with females (at higher food levels, for instance) usually at the expense of non-Prime males. This suggests that males compete more intensely among themselves than with females. The Prime male mass at pupation is affected by total food and food/larva in the same way as the Prime females (on day 4 of the larval period), but Prime males are smaller and more similar in size to each other than are the Prime females. Competition with females may account for some of the compression of the size distribution, but the early, largely simultaneous pupation on day 5 suggests that Prime males minimize the time to pupation rather than maximizing size at pupation. Prime male age at pupation is affected by food and competition. In two treatments (a and d in FxDxT) Prime males pupate slightly after day 5 apparently because food is abundant (3 day timespan); both of these pupate at larger masses than their 6 day timespan peers. In two other treatments, the two most competition treatments, Prime males pupate slightly after day 5, with the 6 day timespan treatment pupating mostly on day 6, but still before the addition of food at the end of day 6. The mass of Prime males is determined by food, but can be modified by delaying pupation to take advantage of abundant food, or to continue growing because food is scarce. The aliquot treatment does not interact with competition for Prime males; there is no effect of dividing the total food into 2 or 4 aliquots. In the FxD interaction, the mass of the Prime male is determined by other factors, but the age at pupation is affected by the residual competition after the higher order interactions have been removed. Prime male age at pupation is earliest with the least competition treatment, a little later in the intermediate competition treatments and disproportionately delayed in the most competition treatment.

Average males: The Average male mass responds to food level, density, competition and timespan, but is not affected by the interactions between competition (FxD) and aliquot. The Average male mass deviates from the pattern shown by the Prime male and female mass variables and by the Average female mass. Non-Prime males experience a release from competition after the Prime male pupates, and in the 6 day timespan treatments, they receive additional food and appear to grow larger as a result. Prime males outcompete non-Prime males in the high food, high density, 3 day timespan treatment resulting in larger Prime males and smaller non-Prime males compared to the equivalent food/larva treatment, low food, low density, 3 day timespan. Despite the same food/larva (4 mg food/larva) the Prime males are smaller and the Average male mass is larger at the low density than at the high density. There is also a residual effect of competition on the size of non-Prime males (in the FxD interaction). Non-Prime males decrease in size in the treatments with increased competition, but they are disproportionately small in the most competition treatment (FxD). Similarly to the FxDxT treatment h, most competition with the 6 day timespan, the non-Prime males grow larger that the Prime males in the FxD most competition treatment. There is no timespan treatment here, so the increase in size of the non-Prime male over the Prime males must be due to the release from competition rather than additional food. There is also evidence that competition among males is more intense in the high food, high density treatment than in the low food, low density treatment (with the same food/larva). The Prime male is larger, the Average male mass is smaller and the difference between them is larger in the high food, high density test tubes compared to the low food, low density test tubes.

In the FxDxT and FxD interactions, Prime and Average males are largest in the least competition treatments, and smallest in the most competition treatments. The high density intermediate competition treatments (4 mg food/larva) produce larger Prime males and smaller Average males than the low density intermediate competition treatments (also 4 mg food/larva). The largest difference between Prime and Average males is in the high density intermediate competition treatments. Non-Prime males grow larger than the Prime males in the most competition treatment. In the FxD interaction, this increment is 0.01 mg larger than the Prime males. In the FxDxT interaction, the increment is 0.14 mg larger. The FxD increment represents only the release of competition after the Prime male pupates, while the FxDxT increment represents both the release of competition and the additional food at the end of day 6 in the 6 day timespan treatment. The effect of the release from competition with the Prime male is much smaller than the effect of the additional food after day 6.

In the FxDxT interaction, the total food after day 4 explains the size order of Prime males when the food/larva is 4 mg or above. The food/larva after day 4 explains the size order of Prime males below 4 mg food/larva. The outcome of competition for the Prime males is directly related to the availability of food after day 4. This is not true of the Average male mass. The outcome of competition among males in the FxDxT interaction resembles that of the FxD interaction for the 3 day timespan treatment. Prime males are smaller in the 6 day timespan for each treatment. The non-Prime males grow larger in the 6 day timespan treatment than in the 3 day timespan treatment in the least competition test tubes. The non-Prime males grow larger than the Prime males in the most competition test tubes. Both are due to the release from competition and the additional food after day 6. In the intermediate competition treatments (4 mg food/larva), the effect of the 6 day timespan is to reduce the size of both Prime and Average males, and to reduce the difference between them at the low density, but to increase that difference at the high density.

Competition among males is affected by females, but males appear to be competing more intensely among themselves than directly with females. Males grow to a size determined by various environmental factors and pupate earlier and at a smaller size than females. However, males that fail to pupate early may delay pupation and grow larger, suggesting that adult longevity and other benefits of larger size may be viable alternatives to early emergence.

**Interactions not involving FxD competition**

The previous four interactions describe competition (FxD) and the way that the factors timespan and aliquot affect competition (FxDxT, FxDxA, and FxDxAxT). Timespan and aliquot change the amount of food in the test tubes over time and this influences the nature and intensity of the competition among the larvae in those test tubes. The remaining 7 interactions describe the separate effects of food and density on timespan and aliquot (FxT, DxT, FxA, DxA), the interaction between timespan and aliquot (AxT) and the separate effects of food and density on that interaction (FxAxT, DxAxT).

**FxAxT, then FxA, FxT and AxT**

These four interactions only involve factors that affect the food supply. They describe the growth of larvae, not competition between larvae. The larvae are competing with each other in the test tubes, but without density as a factor in the interaction, the effect of competition is averaged out across the densities.

**The FxAxT interaction (not significant in the MANOVA)**

The interaction of food, aliquot and timespan is significant only for the Prime female mass and the Average female mass in the ANOVAs, and not significant at all in the MANOVA. The r squared values for both variables are 0.01, so this 3-way interaction does not explain much of the variance for either. However, the 2-way interactions between these three variables were significant in the MANOVA and the r squared values for Prime female mass and Average female mass are larger in the 2-way interactions. It is necessary to account for the 3-way interaction before considering the 2-way ones. This interaction is between three characteristics of the food supply. No density is involved, so the interaction describes growth rather than competition. The behavior of the female larvae may still be affected, switching from active filtering to retaining particles, but the end points, mass and age at pupation indicate the nature of growth on different characteristics of the food, not different competitive environments.

S29 Table shows the means and standard errors for FxAxT for Prime female mass and age, and Average female mass. Only the two mass variables are significant for this contrast in the ANOVAs; the Prime female age is included because the estimated growth rate combines the two mass and age in a biologically relevant number. The main effects are: higher food results in larger mass; 4 aliquots result in better growth than 2 aliquots; and the 3 day timespan results in better growth than the 6 day timespan. The test tubes with high food, 4 aliquots and 3 day timespan should produce the largest masses and the test tubes with low food, 2 aliquots and 6 day timespan should produce the smallest masses. This is true for both the Prime female mass and the Average female mass. The eight treatments are ranked by Prime female mass (a-h); these are not the same rankings as in earlier descriptions. Heuristic 3D plots of the means are presented in S23 Fig-S24 Fig.

1. Across the eight treatments, the Prime females are larger in each of the high food treatments (a-d) than in any of the low food treatments (e-h); however aliquot and timespan jointly affect the Prime female mass so that the values overlap within each food level.
2. Within each food level, the Prime female mass for the 3 day timespan treatment is similar regardless of the number of aliquots. For the low food treatments, the 3 day timespan produces Prime female masses of 3.70 mg (f, 2 aliquots) and 3.72 mg (e, 4 aliquots). For the high food treatments, the 3 day timespan produces Prime female masses of 4.68 mg (b, 2 aliquots) and 4.72 mg (a, 4 aliquots).
3. At each food level, the 6 day timespan interacts with the aliquot treatment. For the low food treatments, the 6 day timespan produces Prime female masses of 3.01 mg (h, 2 aliquots) and 3.34 mg (g, 4 aliquots), a difference of 0.33 mg, 8 times greater than that for the 3 day timespan. For the high food treatments, the 6 day timespan produces Prime female masses of 3.85 mg (d, 2 aliquots) and 4.57 mg (c, 4 aliquots), a difference of 0.72, 18 times greater than for the 3 day timespan.
4. Not only is the difference between the 2 and 4 aliquot treatments with the 6 day timespan much greater at the high food level (c versus d) than at the low food level (g versus h), the 4 aliquot treatment, c, grows almost as large as the high food, 3 day timespan treatments (a and b) and the 2 aliquot treatment, d, grows only a little larger than the low food, 3 day timespan treatments (e and f). The 2 aliquot, 6 day timespan treatments at both food levels are disproportionately small, but the difference is exaggerated at the higher food level. This asymmetry is likely the source of the interaction for the Prime female mass.
5. There are three Prime female masses that are similar and large (a-c), followed by another group of three that are similar and smaller (d-f), followed by the smallest two (g and h).
6. The best outcome for the Prime female mass is in a, the high food, 4 aliquot, 3 day timespan treatment. The next best is in b, the high food, 2 aliquot, 3 day timespan treatment; the difference between the two is 0.04 mg. The third best is in c, the high food, 4 aliquot, 6 day timespan treatment; the difference between b and c is 0.11 mg, so the three span a range of 0.15 mg. There is a gap of 0.72 mg between c and d, the high food, 2 aliquot, 6 day treatment. The next two treatments are 0.13 mg and 0.15 mg smaller than d; these are: e, the low food, 4 aliquot, 3 day timespan treatment and f, the low food, 2 aliquot, 3 day timespan treatment. There is another gap of 0.36 mg to g, the low food, 4 aliquot, 6 day timespan treatment, and another gap of 0.33 mg to h, the low food, 2 aliquot, 6 day timespan treatment.
7. S29 Table and the 3D plot in S23 Fig indicate that the two 3 day timespan treatments are similar across aliquot treatments for each food level, while the two 6 day timespan treatments differ by aliquot treatment, and that the effect of aliquot was more extreme at the high food level (both better and worse) than at the low food level. The difference between the largest Prime female mass, a, and that in the high food, 4 aliquot, 6 day treatment, c, is 0.15 mg (3% of the largest Prime female mass). The comparable difference between the 3 day timespan, b, and the 6 day timespan, d, for the high food, 2 aliquot treatment is 0.83 mg (18% of the largest Prime female mass). The comparable differences for the low food treatments are 0.38 mg (e and g, 8%) and 0.69 mg (f and h, 15 %). The Prime female mass is greater at the high food level than at the low food level. The effect of aliquot is primarily on the 6 day timespan treatments rather than the 3 day timespan treatments, and this effect is greater at high food than at low food.
8. This grouping of Prime female masses is clearly related to the amount of food and the early delivery of food in the test tubes (see S30 Table). The larvae in a, the high food, 4 aliquot, 3 day timespan test tubes, receive 8 mg of food on day 0, 8 mg of food on day 1, 8 mg of food on day 2, 8 mg of food on day 3. The larvae in b receive 16 mg of food on day 1 and 16 mg of food on day 3. 4 aliquots are better than 2 aliquots at the high food level and 3 day timespan, but the difference is small (0.04 mg).
9. The larvae in c receive 8 mg of food on day 0, 8 mg on day 2, 8 mg on day 4 and then the Prime females pupate before the final input of food on day 6. They have only three-fourths of the total food (24 mg of food) that the larger Prime females (a and b) receive, but still grow to almost as large (0.13 mg smaller than b).
10. The larvae in d, the high food, 2 aliquot, 6 day timespan test tubes, receive 16 mg of food on day 0 and most of the Prime females pupate before the final input of food on day 6. They have only 16 mg of food and are 0.72 mg smaller than the other high food level Prime females.
11. The next two Prime females, e and f, both are in test tubes that receive 16 mg of food over 3 days. Comparing these treatments, 4 aliquots are better than 2 aliquots, so the early delivery of food increases the size of the Prime female. Notably, the initial input of 16 mg of food on day 0 (treatment d) increases the size of the Prime female by 0.13 mg, more than the difference between e and f (0.02 mg).
12. All three of these treatments (d-f) pupate on or after day 6, although both e and f receive all their allotted food by day 3; it appears that 16 mg of food is enough for the Prime female to reach a size sufficient to trigger pupation.
13. The smallest two treatments, g and h, do not receive 16 mg of food until the end of day 6; they both extend the larval period by more than a day after the final input, but do not catch up to any of the other Prime females in mass despite equivalent total food.
14. Early addition of food is more important to the growth of Prime females than later additions, although Prime females grow larger on 4 aliquots than 2 aliquots despite the larger initial input (but see growth rates below).
15. This interaction arises because the high food, 4 aliquot, 6 day timespan is almost as good an environment as the two high food, 3 day timespan treatments, but the high food, 2 aliquot, 6 day timespan treatment is only a little better than the two low food, 3 day timespan treatments. The difference between the two low food, 6 day timespan treatments is not as extreme.
16. In the competition interactions, the total food and food/larva jointly affected the size at pupation for the Prime female. In this interaction, food/larva is obscured because both densities are included in each treatment, but total food explains the size order of the Prime female. In the two treatments that receive 32 mg total food by the end of day 4 (a and b), the test tubes receiving 4 aliquots grow slightly larger than those receiving 2 aliquots. The third largest treatment receives 24 mg total food. There are 3 treatments that receive 16 mg total food: d receives 16 mg on day 0; e receives 4 aliquots by day 3; and f receives 2 aliquots by day 3. Receiving all the food on day 0 results in larger Prime females than either aliquot treatment, but 4 aliquots are better than 2. More food, earlier in the larval period is better.
17. The Prime female age at pupation is affected by the main effects: food, aliquot, and timespan, but not by the 3-way interaction. The treatment that results in the earliest age at pupation is b, high food, 2 aliquots, and 3 day timespan. This treatment produces the second largest Prime female (4.68 mg), but has the highest growth rate (0.88 mg/day) because these Prime females pupate earlier than the ones in a. This underscores the earlier statement that this interaction describes growth processes rather than competitive processes. All the competitive (food x density) interactions associated the largest individual with the fastest growth rate.
18. The treatment with the largest Prime female (4.72 mg), a, high food, 4 aliquots, and 3 day timespan, has the second earliest age at pupation and the second highest growth rate (0.83 mg/day). Treatment b receives 16 mg of food on day 0 and another 16 mg of food on day 3. Treatment a receives 8 mg of food on day 0, another 8 mg on day 1, another 8 mg on day 2 and the final 8 mg on day 3. Larvae probably experience an unlimited food supply as 1st instars and perhaps also as 2nd instars, especially at these high food levels. Treatment a receives an increment of food on day 2 and another on day 3, but treatment b receives a larger increment on day 3 only. This results in a slightly earlier pupation at a slightly smaller size, but a faster growth rate.
19. The number or size of the aliquots, in the middle of the larval period, appears to alter the timing of pupation and the size of the pupa. The changing abundance of food (both increasing and decreasing) may be involved in triggering pupation for Prime females.
20. The rest of the growth rates follow the mass of the Prime female.
21. The third place for Prime female mass, age, and growth rate is c, high food, 4 aliquots, and 6 day timespan. These Prime females pupate at a large size (4.57 mg) and fast growth rate (0.77 mg/day) despite pupating before the final aliquot of food is added on day 6. They pupate after 5.92 days and the final aliquot is added after the pupae are collected on day 6. 24 mg total food is clearly sufficient for fast growth, large size and early pupation.
22. The fourth ranked growth rate (0.62 mg/day) is in d, the high food, 2 aliquot, 6 day timespan. The pupal mass and the growth rate are ranked fourth, but the age at pupation is fifth, indicating that these larvae delayed pupation to grow larger. Some of these Prime females pupate after the addition of the second aliquot of food on day 6. The fifth ranked growth rate, 0.60 mg/day for e, is associated with a smaller Prime female mass, but an earlier age at pupation. The Prime female with the sixth ranked growth rate, 0.56 mg/day for f, is almost the same size as the fifth ranked, but the age at pupation is later. These three treatments pupate on 16 mg total food, the apparent minimum required for Prime females to pupate (see the competition interactions). The earliest Prime females to pupate are in the 4 aliquot treatment, e, followed by the Prime females that receive 16 mg on day 0 (treatment d) followed by the 2 aliquot treatment (f). The Prime females that receive 16 mg total food on day 0 appear to experience more food than either of the other two; they grow larger and extend their larval life by a fraction of a day.
23. The seventh and eighth ranked growth rates (0.45 mg/day and 0.33 mg/day, g and h) are both associated with small Prime females and later ages at pupation. These two correspond with the treatments that did not have 16 mg of food before the final food input on day 6.
24. The two smallest Prime females, g and h, both delay pupation significantly (1.36 days and 3.20 days after day 6). They are 1.35 mg and 1.71 mg smaller than the largest Prime female (71% and 64% of treatment a), but the growth rates are relatively much smaller (one-half and one-third of the fastest growth rate, treatment b, because of the extended age at pupation of these smallest Prime females.
25. The Average female mass is similar to the Prime female mass.
26. The largest Average female mass is in a, the high food, 4 aliquot, 3 day timespan treatment.
27. The smallest is in h, the low food, 2 aliquot, 6 day timespan treatment.
28. The three largest treatments (a-c) are similar in size (4.36 mg to 4.57 mg, a range of 0.21 mg). There is also a gap (0.84 mg) between these and the next group.
29. The middle three (d-f) are also similar in size (3.50 mg to 3.56 mg, a range of 0.06 mg), but the Average female mass in d, the high food, 2 aliquot, 6 day treatment is between the Average female masses for e and f, the two low food, 3 day timespan treatments. The Prime female in d appeared to have the most food and to grow the largest and fastest despite delaying pupation. The Average female does not grow as large as those in e and f, despite receiving an addition 16 mg of food on day 6. Early food benefits the Prime female more than the Average female in this comparison.
30. The two smallest Average female masses (g and h) are also separated by a gap (0.33 mg) from the middle three, but the smallest (h) is much smaller than g (0.44 mg smaller).
31. The overall range in sizes is greater for the Average female mass (1.84 mg between the largest and smallest, a-h) than for the Prime female mass (1.71 mg), and the gap between the largest three (a-c) and the middle three (d-f) is also larger (0.84 mg for the Average female mass compared to 0.72 for the Prime female mass). The grouping of the middle three (d-f) is tighter (0.06 mg for the Average female mass compared to 0.15 mg for the Prime female mass). The gap between the middle three (d-f) and the two smaller ones (g and h) is about the same as for the Prime females (0.33 mg and 0.36 mg). The difference between g and h is greater for the Average female mass (0.44 mg) than for the Prime female mass (0.33 mg). The Prime females are expected to be larger than the Average females, but the difference in the ranges from the largest to the smallest suggests that some conditions favor the growth of Prime females over that of the non-Prime females, affecting the Average mass of females.
32. The differences between the Prime female mass and the Average female mass for each treatment combination are shown in S31 Table (column “Prime female mass MINUS Average female mass).” This difference is always smaller for the 3 day timespan than for the 6 day timespan for each of the food x aliquot combinations. The Prime female is relatively larger in the 6 day timespan despite the additional food that is delivered on day 6, after some of the Prime females pupate. This means that the additional food does not enhance the growth of the non-Prime females. The distribution of sizes is determined earlier in the larval growth period and doesn’t change even with a large increment of food (8 mg at the low food level, or 16 mg at the high food level).
33. The difference between the Prime female mass and the Average female mass in the high food, 4 aliquot, 3 day timespan treatment (a) is the smallest (0.15 mg) across all the treatments. The low food, 4 aliquot, 3 day timespan treatment (e) is also small, suggesting that the 4 aliquot, 3 day timespan combination is good for the non-Prime females and raises the Average female mass relative to the Prime female mass.
34. The difference between the Prime female mass and the Average female mass in the 2 aliquot, 6 day timespan treatments (d and h, 0.33 mg and 0.28 mg respectively) are the largest across the interaction. At both food levels, the 2 aliquot, 6 day timespan results in the smallest Prime and Average female masses, but the Average females are affected more than the Prime females.
35. The gap between the largest females (a-c) and the middle group (d-f) is larger for the Average female than for the Prime female. This appears to be due to the greater advantage of the Prime female over the Average female in d) the high food, 2 aliquot, 6 day timespan.
36. The Prime females in d grow larger than those in e and f with the same amount of food (16 mg); the 16 mg on day 0 provides better growing conditions than the two 8 mg inputs in f or the four 4 mg inputs in e. However, the Average female in e grows larger than the Average female in d and the Average female in f grows almost as large.
37. The dynamics of food level have different effects on the Prime and non-Prime females. Prime females grow best on a single large input of food on day 0 (d); non-Prime females grow better on the same amount of food divided into 4 aliquots (e). This is despite the addition of more food after day 6 in treatment d.
38. The smallest females occur at the low food level and the 6 day timespan (g and h). Both Prime and Average females are larger with 4 aliquots than with 2 aliquots, but the difference between the Prime and Average females is smaller at 4 aliquots (0.17 mg, among the smallest of the differences) and larger at 2 aliquots (0.28 mg, the second largest difference).
39. In both g and h, the Prime female delays pupation, probably because there is insufficient food before the final input on day 6.
40. The gap in size between treatments f and g is about the same for both the Prime female and the Average female. The non-Prime females in g grow better on the 4 aliquots of food relative to the Prime female; this similar to e above.
41. The gap between treatments g and h is greater for the Average female mass than for the Prime female mass. The initial input of food is only 8 mg and is not supplemented until day 6, when the second 8 mg is added. The Prime female is small (3.01 mg, the smallest across this interaction), but the non-Prime females are even smaller (reducing the size of the Average female mass to 2.73, causing the 0.44 mg gap between g and h, and contributing to the 1.84 mg range between the largest Average female mass and the smallest).

S29 Table. Means (SE) for Prime female mass and age at pupation and Average female mass at pupation for the interaction FxAxT. Estimated growth rate and difference between the Prime and Average female mass.

| Food x Aliquot | Timespan | Rank by Prime female mass (a-h) | Prime female mass at pupation (mg) | Prime female age at pupation (days) | Average female mass at pupation (mg) | Estimated growth rate for Prime female (mg/day) | Prime female mass MINUS Average female mass (mg) |
| --- | --- | --- | --- | --- | --- | --- | --- |
| Low food, 2 aliquots | 3 days | f | 3.70 (1.05) | 6.60 (1.00) | 3.50 (1.03) | 0.56 (0.57) | 0.20 (1.04) |
|  | 6 days | h | 3.01 (0.37) | 9.20 (1.98) | 2.73 (0.34) | 0.33 (0.47) | 0.28 (0.36) |
| Low food, 4 aliquots | 3 days | e | 3.72 (0.96) | 6.16 (0.40) | 3.56 (1.03) | 0.60 (0.44) | 0.16 (1.00) |
|  | 6 days | g | 3.34 (0.76) | 7.36 (1.07) | 3.17 (0.74) | 0.45 (0.42) | 0.17 (0.75) |
| High food, 2 aliquots | 3 days | b | 4.68 (0.30) | 5.31 (0.13) | 4.48 (0.40) | 0.88 (0.20) | 0.20 (0.35) |
|  | 6 days | d | 3.85 (0.44) | 6.25 (1.06) | 3.52 (1.06) | 0.62 (0.50) | 0.33 (0.60) |
| High food, 4 aliquots | 3 days | a | 4.72 (0.04) | 5.67 (0.06) | 4.57 (0.21) | 0.83 (0.04) | 0.15 (0.15) |
|  | 6 days | c | 4.57 (0.41) | 5.92 (0.30) | 4.36 (0.42) | 0.77 (0.28) | 0.21 (0.42) |

S30 Table. Means (SE) for Prime female mass and age at pupation and Average female mass at pupation for the interaction FxAxT. Total food and food/larva after day 4.

| Food x Aliquot | Timespan | Rank by Prime female mass (a-h) | Prime female mass at pupation (mg) | Prime female age at pupation (days) | Average female mass at pupation (mg) | Total food after day 4 (mg) | Food/larva after day 4 (mg) |
| --- | --- | --- | --- | --- | --- | --- | --- |
| Low food, 2 aliquots | 3 days | f | 3.70 (1.05) | 6.60 (1.00) | 3.50 (1.03) | 16 | 2, 4 |
|  | 6 days | h | 3.01 (0.37) | 9.20 (1.98) | 2.73 (0.34) | 8 | 1, 2 |
| Low food, 4 aliquots | 3 days | e | 3.72 (0.96) | 6.16 (0.40) | 3.56 (1.03) | 16 | 2, 4 |
|  | 6 days | g | 3.34 (0.76) | 7.36 (1.07) | 3.17 (0.74) | 12 | 1.5, 3 |
| High food, 2 aliquots | 3 days | b | 4.68 (0.30) | 5.31 (0.13) | 4.48 (0.40) | 32 | 4, 8 |
|  | 6 days | d | 3.85 (0.44) | 6.25 (1.06) | 3.52 (1.06) | 16 | 2, 4 |
| High food, 4 aliquots | 3 days | a | 4.72 (0.04) | 5.67 (0.06) | 4.57 (0.21) | 32 | 4, 8 |
|  | 6 days | c | 4.57 (0.41) | 5.92 (0.30) | 4.36 (0.42) | 24 | 3, 6 |

**FxAxT summary**

This 3-way interaction describes the effect of three attributes of the food supply on the growth of females. The food level is the most important factor. Within each food level there is an interaction between aliquot and timespan; aliquot has little effect at the 3 day timespan, but a large effect at the 6 day timespan, and a larger effect at the high food level than at the low food level. The Prime female masses are arranged in 3 groups with large gaps between each group. The largest Prime females are in the high food treatment; the two largest receive 32 mg of food by day 3 and the third largest receives 24 mg of food by day 4. The next group receives 16 mg of food on day 0 or by day 3. The third group doesn’t receive 16 mg of food until day 6. Prime females require 16 mg of food to pupate, so the last group delays pupation compared to the first two groups. The total food is the most important factor, but the early delivery of food (3 day timespan and 4 aliquots, jointly) increases the size of the Prime females.

At the highest food level the Prime females in the 4 aliquot, 3 day timespan treatment grow largest, but those in the 2 aliquot, 3 day timespan treatment grow fastest and pupate earliest. The large input of food on day 3 appears to accelerate the growth of females. The timing of the input and/or the relative abundance of food particles affects the triggers for pupation in the Prime female at high food levels.

Within the middle group of Prime females, the ones that receive 16 mg of food on day 0 grow largest and fastest, followed by the ones in the 4 aliquot treatment (4 mg of food per day on day 0 - day 3). The Prime females in the 2 aliquot treatment (8 mg of food on day 0 and day 3) are smaller and grow more slowly than the other two. Early delivery of food (1 large input, then 4 aliquots, then 2 aliquots) increases the size of Prime females and their growth rates at moderate food levels.

The estimated growth rates of the Prime females in middle group correspond to their masses. There is no acceleration due to the large input on day 3 similar to that observed at the high food level.

At the low food level and the 6 day timespan, the Prime females do not have enough food to pupate until after the last aliquot on day 6. They grow larger and faster on the 4 aliquot treatment than the 2 aliquot treatment. Despite equal food and a longer larval period, they do not grow as large as the Prime females in the 3 day timespan treatments. Food early in the larval period is more important than later additions for both size and growth rate.

The Average female mass is similar to the Prime female mass, but food level is less important and the interaction between aliquot and timespan is more important. The Average female mass is highest at the high food level in 3 of the 4 treatments; the Average females in the high food, 2 aliquot, 6 day timespan treatment are smaller than the largest Average females in the low food treatments. Within each food level there is an interaction between aliquot and timespan; aliquot has little effect at the 3 day timespan, but a large effect at the 6 day timespan, and a larger effect at the high food level than at the low food level. The 6 day timespan treatments are all smaller than the corresponding 3 day timespan treatments across the interaction. The Average females in the 6 day timespan treatments are also relatively smaller than the Prime females in these treatments (again compared to the corresponding 3 day timespan treatments). The additional food in the final aliquot on day 6 does not result in larger non-Prime females; the distribution of sizes among females is determined earlier in the larval growth period and doesn’t change despite the additional food.

The Average female masses are also arranged in 3 groups with large gaps between each group. The largest Average females are in the high food treatment; the two largest receive 32 mg of food by day 3 and the third largest receives 24 mg of food by day 4. The next group receives 16 mg of food on day 0 or by day 3. The third group doesn’t receive 16 mg of food until day 6. The total food is the most important factor, but the early delivery of food (3 day timespan and 4 aliquots, a and e) increases the size of the Average females as it does for the Prime females.

At the highest food level the Average female mass in the 4 aliquot, 3 day timespan is the largest and the closest to that of the Prime female mass. In this treatment with abundant food, the non-Prime females do better than in any other treatment. There is a bigger difference between the Average female mass in this treatment and the next largest Average female, than between the corresponding Prime females, so the non-Prime females do better on the 4 aliquot treatment than on the 2 aliquot treatment (at the high food level and 3 day timespan).

There is a larger gap in size between the 3 largest Average females and the middle group of Average females (compared to the same gap for the Prime females). In part this is due to the smaller size of the Average females in the high food, 2 aliquot, 6 day timespan relative to the Prime female in that treatment and also to the group of largest Average females. The three treatments in this middle group all receive 16 mg of food either on day 0 or by day 3. The Prime females that receive 16 mg of food on day 0 grow larger than the other two, but the Average females in this treatment do not. The Average females in the 4 aliquot treatment do better than the other two, so again the non-Prime females benefit from the 4 aliquot treatment. There is also a smaller difference between the Prime female mass and the Average female mass in the 4 aliquot treatment, compared to the other two in this middle group. However, the difference between the largest and smallest of these three Average female masses in the middle group is smaller than the difference for the middle group of Prime females. This may indicate that the food supply attributes, aliquot and timespan, are less important to the non-Prime females than the Prime females in this middle range of food.

The gap between the middle group of Average female masses and the two smallest Average female masses is about the same as that for the corresponding Prime females. The Average females in the 4 aliquot treatment do better than the ones in the 2 aliquot treatment (both in test tubes with low food, and 6 day timespan). The difference between the Prime and Average masses also indicates that the non-Prime females grow relatively larger in the 4 aliquot treatment. The Prime and Average female masses in the low food, 2 aliquot, 6 day timespan treatment are the smallest across the interaction, and the difference between the Prime and Average female masses is the second largest, so the non-Prime females face the worst food environment in this treatment.

Large initial inputs of food allow the Prime female to grow faster and to dominate the food supply. Smaller, regular inputs (4 aliquot treatment) allow the non-Prime females to grow larger than equivalent amounts of food in larger inputs (2 aliquot treatment). The food level is the most important factor for the Prime females, followed by the interaction between aliquot and timespan. The interaction between aliquot and timespan is relatively more important for the non-Prime females. The final aliquot in the 6 day timespan appears essential to Prime and Average females at the lowest food level, but otherwise does not appear to benefit either the Prime females (because they pupate before the addition of the food) or the non-Prime females (perhaps because the size distribution is fixed by the molt into the fourth instar).

**The FxA interaction (R squared = 0.25)**

The FxA interaction indicates whether the apparent amount of food changes depending on the number of aliquots it is divided into. There is no density involved, so this examines the effect of food and aliquot on growth. In the MANOVA, the R squared for the interaction is one of the smaller ones, and Prime male mass and age have the two highest discriminant function correlation coefficients (both positive), followed by Survival and the Prime female mass and age, but the coefficients for the two Average mass variables were close to zero. This interaction is significant for the Prime male mass and age at pupation and the Average male mass at pupation in the ANOVAs; the interaction explains only 1% of the variation for each of these. The three variables: Prime male mass and age, and Average male mass, are not significant in the FxDxAxT, FxDxA, or FxAxT interactions described earlier. This is the highest order interaction between food and aliquot for males. Survival, and the female mass and age variables are not significant in the ANOVAs for this interaction.

For both mass variables the main effects are: high food is better than low food, and 4 aliquots are better than 2 aliquots. For the Prime male age at pupation high food is also better than low food, but 2 aliquots are slightly better than 4 aliquots (earlier pupation). S31 Table shows the means and standard errors for the Prime male mass and age at pupation and the Average male mass at pupation for the FxA interaction alongside the estimated growth rate and the difference between the Prime and Average male masses and the expected values of the three variables (see the explanation for S27 Table). S25 Fig presents a heuristic 3D plot of the Prime male mass and age at pupation (the Average male mass is similar to the Prime male mass).

1. In S31 Table both the Prime male mass and the Average male mass are highest at 32 mg of food and 4 aliquots (2.71 mg and 2.60 mg, respectively), and both are lowest at 16 mg and 2 aliquots (2.00 mg and 2.00 mg, respectively), as expected based on the main effects of those two factors.
2. At each food level, both Prime and Average males grow larger with 4 aliquots than with 2 aliquots, but the advantage due to the 4 aliquot treatment is greater at the low food level than at the high food level. The Prime male mass is 0.25 mg larger on 4 aliquots versus 2 aliquots at the low food level and only 0.14 mg larger at the high food level. The corresponding numbers for the Average male mass are 0.22 mg and 0.14 mg. This is likely one source of this interaction for the two male mass variables.
3. The columns, “Expected mean value for Prime male mass at pupation” and “Expected mean value for Average male mass at pupation,” represent the projected means based on the main effects of the two factors, food and aliquot. The values of the projected and observed means are in the same order for both Prime and Average male masses, but the range of the observed means is twice that of the projected ones (2.00 mg to 2.71 mg observed for the Prime male compared to 2.21 mg to 2.56 mg projected; 2.00 mg to 2.60 mg observed for the Average male compared to 2.17 mg to 2.47 mg projected).
4. For both Prime and Average male mass, the values for the high food treatment are higher than the projections and the values for the low food treatment are lower than the projections. This is likely another asymmetry causing this interaction.
5. The column, “Expected mean value for Prime male age at pupation,” represents the projected means based on the main effects of food and aliquot as above. The observed values of Prime male age are in the same order as the projections, but also span twice the range (5.00 days to 5.26 days, observed; 5.06 days to 5.19 days, projected).
6. The high food treatments pupate earlier than expected and the low food treatments pupate later than expected.
7. All the Prime males pupate on or shortly after the 5th day with the low food treatments taking slightly longer than the high food treatments and the 2 aliquot treatments pupating slightly earlier than the 4 aliquot treatments.
8. There is no timespan factor here, so the effect of aliquot on food level is separate from the effect of timespan (and whether the larvae pupate before the final aliquot is added). The effect of the aliquot treatment is slightly larger at the low food level than at the high food level; this is likely the source of this interaction for Prime male age.
9. Prime males take longer to pupate at the low food level than at high food; this likely indicates that they are taking longer to increase their final size in a low food environment. However, at both food levels, the 4 aliquot Prime males take slightly longer than the 2 aliquot Prime males to pupate. The larger size of the Prime males coupled with the later pupation suggests that these males also delay pupation to grow larger when food is more available. The 4 aliquot treatment appears to increase the available food for the Prime male.
10. The smaller size of the Prime male at the low food level and the later pupation result in slower growth rates in the low food treatments, with the slowest growth rate at the low food, 2 aliquot treatment combination.
11. However, the Prime males also took slightly longer to pupate in the high food, 4 aliquot treatment, so the growth rates in the high food treatments are more similar than at the low food treatment. The highest growth rate is associated with the high food, 4 aliquot treatment; these are the largest Prime males, but they pupate slightly later (5.04 days) than the Prime males in the high food, 2 aliquot treatment (5.00 days). Males may be delaying pupation at higher food levels in addition to taking longer to pupate at low food levels.
12. The difference between the Prime male mass and the Average male mass is an indication of the distribution of sizes in the population and the relative advantage of the Prime male over the non-Prime males. The difference between the Prime and Average male masses is 0.11 mg at the high food level (for both aliquot treatments), but much smaller (0.0 mg - 0.03 mg) at the low food level.
13. At the high food level, growth of males is largely unrestricted and Prime and non-Prime males grow according to some underlying size distribution that results from exponential growth and results in the 0.11 mg spread between the Prime and Average male masses.
14. At the lower food levels, the growth is restricted by the food level and by the 2 aliquots. The Prime males pupate later and at a smaller size than in the high food treatments; the non-Prime males continue to grow, pupating at a relatively larger size (compared to the Prime male), but still smaller than the high food Average male mass.
15. At low food and 2 aliquots the Prime male mass and the Average male mass are the same. In this treatment combination the Prime male pupates before the final aliquot of food and the Average males grow larger on the release from competition and also the subsequent increase in food on day 6 for some test tubes.
16. The Prime female mass and the Average female mass are not affected by this interaction (S32 Table), but they are affected by the main effects of the factors, and by other interactions involving food and aliquot (FxDxAxT, FxDxA, and FxAxT). The difference between the Prime female mass and the Prime male mass shows the relative advantage of the Prime female over the Prime male, and the difference between the Average female mass and the Average male mass shows the relative advantage of females over males, including the Prime and non-Prime individuals. For both of these comparisons, the difference is larger at the higher food level and larger values of the masses, suggesting that it is caused by exponential growth processes.
17. The Prime female is largest in the test tubes with high food and 4 aliquots, as is the Prime male, the Average female mass and the Average male mass. The difference between the Prime female and the Prime male is also largest in this treatment (1.94 mg). The difference between the Prime female and the Prime male is also large in the high food, 2 aliquot treatment (1.70 mg).
18. However, the smallest difference between the Prime female and the Prime male is in the low food, 4 aliquot treatment (1.28 mg), not the low food, 2 aliquot treatment (1.36 mg), where both Prime female and Prime male are smallest. The Prime males appear to grow larger relative to the Prime females on 4 aliquots than on 2 aliquots at the low food level. The aliquot treatment affects the exponential growth processes at low food, making it appear that the Prime males have access to more food.
19. The difference between the Average females and the Average males is largest at the high food level and 4 aliquots (1.86 mg) and almost as large at the high food level and 2 aliquots (1.54 mg). The difference between the Average females and the Average males is small in both the low food treatments: 1.14 mg in the low food, 4 aliquot treatment and 1.11 mg in the low food, 2 aliquot treatment. The non-Prime males in the low food, 2 aliquot treatment grow as large as the Prime males in this treatment. The non-Prime males in the low food, 4 aliquot treatment grow almost as large as the Prime males. Some of this is due to the release from competition and subsequent increase in food in one treatment (see #276 above), but the Prime females, the Average females, the Prime males and the Average males all grow larger at low food with 4 aliquots than with 2 aliquots. The differences in mass between the low food, 4 aliquot treatment and the low food, 2 aliquot treatment for each of these variables are on the order of 0.20 mg (0.17 mg to 0.25 mg). The differences in mass between the high food, 4 aliquot treatment and the high food, 2 aliquot treatment are lower for the Prime and Average males (both 0.14 mg) and higher for the Prime and Average females (0.38 mg and 0.46 mg, respectively). This underscores the differences between the two sexes with respect to the interaction.
20. There are two different anomalies here: the difference between the Prime female mass and the Prime male mass is larger for the low food, 2 aliquot treatment (where both are smaller) than for the low food, 4 aliquot treatment (see #279 above); and the difference between the Average female mass and the Average male mass is 0.03 mg despite the 0.25 mg and 0.22 mg size differences between the Average females and Average males across the low food treatments. At the low food level the non-Prime females and non-Prime males are equally constrained by the availability of food, and the positive effect of the 4 aliquot treatment is primarily for the Prime males rather than the non-Prime males.
21. The second anomaly is that the Average male mass is the same as the Prime male mass in the low food, 2 aliquot treatment. This suggests that there is a minimum size at pupation for Prime males that is determined before day 3, and that males grow until they reach that size, but can grow larger than the minimum size in response to greater food availability. This observation is due to one cell in the experiment (see #275 above).

S31 Table. Means (SE) for Prime male mass and age at pupation and Average male mass at pupation for the interaction FxA. Expected mean values for the Prime male mass and age and the Average male mass.

| Food x Aliquot | Prime male mass at pupation (mg) | Prime male age at pupation (days) | Average male mass at pupation (mg) | Prime male growth rate (mg/day) | Prime male mass MINUS Average male mass (mg) | Expected mean value for Prime male mass at pupation (mg) | Expected mean value for Prime male age at pupation (days) | Expected mean value for Average male mass at pupation (mg) |
| --- | --- | --- | --- | --- | --- | --- | --- | --- |
| 16 mg, 2 aliquots | 2.00 (0.57) | 5.20 (0.21) | 2.00 (0.45) | 0.38 (0.17) | 0.00 (0.36) | 2.21 (0.44) | 5.17 (0.24) | 2.17 (0.39) |
| 16 mg, 4 aliquots | 2.25 (0.40) | 5.26 (0.43) | 2.22 (0.40) | 0.43 (0.23) | 0.03 (0.28) | 2.31 (0.44) | 5.19 (0.24) | 2.26 (0.39) |
| 32 mg, 2 aliquots | 2.57 (0.29) | 5.00 (0.00) | 2.46 (0.29) | 0.51 (0.07) | 0.11 (0.21) | 2.46 (0.44) | 5.06 (0.24) | 2.38 (0.39) |
| 32 mg, 4 aliquots | 2.71 (0.16) | 5.04 (0.07) | 2.60 (0.20) | 0.54 (0.05) | 0.11 (0.13) | 2.56 (0.44) | 5.09 (0.24) | 2.47 (0.39) |

S32 Table. Means (SE) for Prime female mass and Average female mass at pupation for the interaction FxA. Differences between the Prime female mass and the Prime male mass and between the Average female mass and the Average male mass.

| Food x Aliquot | Prime female mass at pupation (mg) | Prime female age at pupation (days) | Average female mass at pupation (mg) | Prime female mass MINUS Prime male mass (mg) | Average female mass MINUS Average male mass (mg) |
| --- | --- | --- | --- | --- | --- |
| 16 mg, 2 aliquots | 3.36 (0.75) | 7.90 (1.97) | 3.11 (0.76) | 1.36 (0.47) | 1.11 (0.44) |
| 16 mg, 4 aliquots | 3.53 (0.74) | 6.76 (0.95) | 3.36 (0.76) | 1.38 (0.42) | 1.14 (0.43) |
| 32 mg, 2 aliquots | 4.27 (0.57) | 5.78 (0.82) | 4.00 (0.73) | 1.70 (0.32) | 1.54 (0.39) |
| 32 mg, 4 aliquots | 4.65 (0.25) | 5.80 (0.23) | 4.46 (0.30) | 1.94 (0.15) | 1.86 (0.18) |

**FxA summary**

This interaction shows that the growth of males is affected by the interaction of the amount of food and the number of aliquots it is divided into, separately from competition for food (food x density) and timespan. Both Prime and non-Prime males grow better on 4 aliquots than 2 aliquots. The Prime males pupate before the 6th day, but timespan is not a factor in this interaction; the difference between the 3 day timespan and the 6 day timespan is not important. It appears to be the food delivered in the day 2 to day 4 period (the third aliquot) that causes all the larvae to grow larger in the 4 aliquot treatment (the main effect of aliquot). This interaction between food and aliquot arises because males grow larger than expected and take longer in the low food, 4 aliquot treatment. Males are growing rapidly and filtering particles, so the added particles accelerate their growth, and this has a larger effect at low food levels than at high food levels. Prime males defer pupation slightly and extend their growth in response to the third aliquot of food and grow larger than Prime males at the same food level and the 2 aliquot treatment. Although the Prime and Average males are smaller in the low food treatment, the third aliquot has a larger impact on growth at the low food level compared to the high food level.

The low food treatment causes the females to switch from active filtering to retaining particles in their guts, reducing the available particles. This reduces the size and size distribution among the females, and also reduces the size and the size distribution among males. Males are smaller than projected at the low food level, especially in the 2 aliquot treatment, but the size distribution of the males is also compressed. This can be explained by exponential growth processes. At the low food level, Prime males have less of an advantage over the non-Prime males than at the high food level. Prime and Average male masses in the low food, 4 aliquot treatment grow larger than in the 2 aliquot treatment, and are almost as large as their projected values. This is due to the third aliquot of food that is delivered in the middle of the larval growth period (day 2 or day 4, depending on timespan treatment). Prime and non-Prime males grow larger but the relative advantage of the Prime female over the Prime male is smaller, so the Prime male benefits more from this third aliquot food addition than the non-Prime males. Males appear to actively filter at all food levels, unlike females, which appear to switch from active filtering to retaining particles in their guts. In this situation, the males respond sooner to the third aliquot and the Prime male grows larger relative to the Prime female. The Prime male also grows larger relative to the non-Prime males due to exponential growth processes and the initial size distribution before the third aliquot. The Prime males extend their larval period to grow larger on the extra food, and pupate latest in this treatment (across this interaction).

The non-Prime male masses are in the same order as the Prime male masses, so all males appear to benefit from the third aliquot, but the Average male mass in the low food, 2 aliquot treatment is the same as the Prime male mass. The non-Prime males in this treatment grow as large as the Prime males. They don’t grow as large as the non-Prime males in the low food, 4 aliquot treatment; the third aliquot delivers more food earlier in the larval lifespan. The non-Prime males in the low food, 2 aliquot treatment must take advantage of the additional food in the final aliquot (on day 3 or day 6) to grow as large as the Prime male. This suggests that there is a minimum mass at pupation for males that is determined by environmental conditions before day 3. Male larvae grow until they reach that minimum, or grow larger than the minimum, even delaying pupation, in response to greater availability of food.

The main effect of the 4 aliquot treatment increases the size of all larvae compared to the 2 aliquot treatment, but for males at the low food level, it provides a disproportionate benefit. There are two different anomalies: the Prime male mass in the low food, 4 aliquot treatment is disproportionately large relative to both the Average male and to the Prime female; and the Average male mass in the low food, 2 aliquot treatment is the same as the Prime male mass in that treatment. The Prime male grows larger on the third aliquot at the low food level in relation to the Prime female. The non-Prime males grow to the same size as the Prime males at the lowest food level, suggesting a minimum mass at pupation for males depending on the availability of food before day 3.

**The FxT interaction (R squared = 0.61)**

The interaction between food and timespan shows the residual effect after the higher order interactions have been explained (FxDxT for all variables, FxAxT for Prime and Average female mass, and FxDxAxT for Average female mass). The MANOVA coefficients are large and positive for the Prime male age, smaller and positive for the rest of the variables except for Prime female age, which is close to zero. The R squared for this contrast is one of the highest for interaction contrasts across the MANOVA. The ANOVAs are significant for all the variables except for Prime female age. The r squared values for the significant variables are: 0.08, 0.09, 0.02, 0.08, 0.03, and 0.02 (Survival, Prime male mass and age, Average male mass, Prime female mass, Average female mass, respectively). This interaction is about 3 times more important for males than for females. The main effects are: higher food is associated with better Survival, larger mass, and earlier pupation; 3 day timespan is associated with lower Survival, larger mass and earlier pupation. There is no density in this interaction, so the contrast describes the effect of food and timespan on the growth of larvae.

S33 Table shows the means and standard errors for the FxT interaction for arc sine transformed percent Survival. This interaction has the highest r squared value for Survival of any interaction and explains 8% of the variance in the experiment for Survival (27% of the variance in Survival is explained by the experiment, so this interaction accounts for more than a quarter of the explained variance).

1. Survival is best at the high food, 6 day timespan treatment (1.32) and worst at the high food, 3 day timespan (1.14).
2. Survival in both low food treatments is similar (1.18 and 1.20) and closer to the high food, 3 day timespan than to the best outcome.
3. The interaction appears to be due to the asymmetrically higher Survival at high food with the 6 day timespan.
4. The column, “Expected mean values for Survival,” represents the projected values based on the main effects, food and timespan. The best projected Survival is in the high food, 6 day timespan treatment, but it is lower than the observed value.
5. The worst projected Survival is in the low food, 3 day timespan treatment, not the observed high food, 3 day timespan. This asymmetry may also be a cause of the interaction.
6. Also, the range of the observed means is 0.18 (arc sine transformed percent Survival) three times the projected range of 0.06.
7. This is different from the result in the FxDxT interaction described previously. In that interaction the lowest value was in the most competition test tubes with the 6 day timespan, while the other three 6 day timespan treatments were the highest. Competition, not food level, was the most important determinant of Survival. In this contrast, the highest and lowest values of Survival are at the high food level. Survival appears to be improved by the late addition of food at high food levels and possibly compromised by too much food in the high food, 3 day timespan treatment. (see experiment 2).

S33 Table. Means (SE) for arcsin transformed percent Survival for the interaction FxT.

| Food x Timespan | Survival |
| --- | --- |
| 16 mg, 3 days | 1.20 (0.16) |
| 16 mg, 6 days | 1.18 (0.22) |
| 32 mg, 3 days | 1.14 (0.16) |
| 32 mg, 6 days | 1.32 (0.12) |

S34 Table shows the means and standard errors for the FxT interaction for the Prime female mass and age at pupation and the Average female mass at pupation. The Prime female age at pupation is not significantly affected by this interaction, but it is useful to consider as a component of the estimated growth rate of the Prime females. Across the ANOVA for both Prime female mass and Average female mass, this interaction ranks in the middle with respect to the explained variance (3% for Prime female mass and 2% for Average female mass). A heuristic 3D plot of the Prime female mass and age at pupation is presented in S26 Fig.

1. For all three variables the outcomes are better (larger masses, earlier pupation) at the high food level than at the low food level; food is more important than timespan in this interaction.
2. For both female mass variables, the best outcomes are in the test tubes with high food and 3 days timespan (4.70 mg for Prime and 4.52 mg for Average female mass) and the worst outcomes are in the test tubes with low food and 6 days timespan (3.18 mg and 2.95 mg, respectively).
3. The females in the high food, 3 day timespan treatment grow largest and the Prime females pupate earliest. The females in the high food, 6 day timespan are about 0.50 mg smaller than the ones in the high food, 3 day timespan and the Prime females pupate about a half day later, but most of them pupate before the addition of the last aliquot on the 6th day. The females in the low food, 3 day timespan are about 0.5 mg smaller than the ones in the high food, 6 day timespan (1.0 mg smaller than the ones in the high food, 3 day timespan) and the Prime females pupate slightly later. The females in the test tubes with the worst outcomes, low food, 6 day timespan are also 0.5 mg smaller than the next worst treatment, but the Prime females defer pupation until more than two days after the addition of the last aliquot on the 6th day.
4. The columns, “Expected mean values for Prime female mass and Expected mean values for Average female mass,” represent the projected means for each treatment based on the main effects of food and timespan (see the explanation for S27 Table). The highest values and lowest values correspond to the treatment combinations as observed and the sequence is the same for both Prime and Average masses, but the observed ranges of sizes are twice the projected ranges (Prime female mass observed is 3.18 mg to 4.70 mg, a span of 1.52 mg; Prime female mass projected is 3.57 mg to 4.08 mg, a span of 0.77 mg; Average female mass observed is 2.95 mg to 4.52 mg, a span of 1.57 mg; Average female mass observed is 3.34 mg to 4.13 mg, a span of 0.79 mg).
5. The observed means at the high food level are greater than projected and the observed means at the low food level are less than projected for both Prime and Average female masses. The deviation from the projected means are greater in the best (high food, 3 day timespan) and worst (low food, 6 day timespan) treatments. This is likely a source of the interaction.
6. The factors, food and timespan, double between the low value and the high value (16 mg of food versus 32 mg; 3 days versus 6 day). The observed mean values for both Prime and Average female mass increase by about 0.5 mg from the lowest treatment combination to the highest. It appears that adding the 16 mg of food in 3 days rather than 6 days increases the food level so that both Prime and Average masses increase by about 0.5 mg, and that doubling the food to 32 mg over 6 days also increases the food level by the same amount, as does adding the 32 mg in 3 days rather than 6 days. For the Prime female, the age at pupation also decreased (not as regularly) with increasing food.
7. These increments are also evident in the projected means for Prime and Average female mass; they are half the size of the observed ones (0.25 mg approximately), but just as regular.
8. The smallest females are in test tubes that receive: 4 mg or 8 mg of food on day 0; 4 mg or nothing on day 2; 4 mg or nothing on day; and 4 mg or 8 mg on day 6; depending on the aliquot treatment assigned.
9. The next smallest are in test tubes that receive: 4 mg or 8 mg of food on day 0; 4 mg or nothing on day 1; 4 mg or nothing on day 2; and 4 mg or 8 mg on day 3; depending on the aliquot treatment assigned.
10. The first treatment receives 8 mg or 12 mg of food by day 4 and the second treatment receives 16 mg of food by day 3, a clear difference in the apparent food level.
11. The next largest females are in test tubes that receive: 8 mg or 16 mg on day 0; 8 mg or nothing on day 2; 8 mg or nothing on day 4; 8 mg or 16 mg on day 6; depending on the aliquot treatment assigned.
12. The largest females are in test tubes that receive: 8 mg or 16 mg on day 0; 8 mg or nothing on day 1; 8 mg or nothing on day 2; 8 mg or 16 mg on day 3; depending on the aliquot treatment assigned.
13. The third treatment receives 16 or 24 mg of food by day 4 and the fourth (best) treatment receives 32 mg by day 3.
14. The stepwise increments of food are: 8 mg-12 mg, 16 mg, 16 mg-24 mg, and 32 mg. This probably accounts for the stepwise increments in the sizes of Prime and Average females, with the exception of the smallest ones. The Prime females in the low food, 6 day timespan delay pupation until after the last aliquot of food has been added on day 6. Interactions described earlier have suggested that Prime females need 16 mg of food in the test tubes to initiate pupation. Once the last aliquot of food is added, the females grow and pupate; they do not grow as large as the females in the test tubes with the equivalent amount of food delivered in 3 days rather than in 6 days, but they do exploit the 6th day aliquot of food, which none of the other Prime females do.
15. Again, the equivalent amount of food, delivered earlier in the larval period, has a greater effect on the growth of females.
16. The estimated growth rate combines the Prime female mass and age in a biologically meaningful number. The highest growth rate is associated with the largest mass and the earliest age at pupation (high food, 3 day timespan). The remainder of the growth rates are in accord with the Prime female masses. The smallest growth rate is much smaller because the smallest Prime female mass is associated with the latest pupation (2.28 days after the final aliquot of food on the 6th day). There appears to be no asymmetry other than for the Prime female mass; the Prime female age is not significantly affected by this interaction and the main effects that do affect the Prime female age do not change the relationships among the Prime female masses.
17. The difference between the Prime and Average female mass is a measure of the size distribution of the females and the relative advantage of the Prime female over the non-Prime females. The smallest difference is in the two treatments with the 3 day timespan (0.18 mg, both high food and low food treatments) and the largest difference is in the high food, 6 day timespan treatment (0.27 mg). The difference between the two masses in the low food, 6 day timespan treatment is intermediate (0.23 mg).
18. In the test tubes with the 3 day timespan all the food is added by day 3; the Prime females grow to the limit of the available food and pupate, and the non-Prime females grow to the limit of the remaining food and pupate as well. The similar size difference between the Prime and Average females in the high and low food treatments with the 3 day timespan suggests that similar mechanisms of feeding (filtering versus retention) are taking place at both food levels. All females grow as though food is more available in the 3 day timespan treatment than in the 6 day timespan treatment.
19. The larger differences between Prime and Average female masses in the 6 day timespan treatments indicate that Prime females grow better than the non-Prime females when food is limited (6 day timespan), although both Prime and Average female masses are smaller than in the 3 day timespan at the same food level.
20. The Average females are smaller and the difference between Prime and Average is larger despite the additional food available to the non-Prime females after the 6th day. Previous interactions have shown that late additions of food contribute little to the ultimate size of the female pupae.
21. Prime and non-Prime females all grow larger at the higher food level; exponential growth is sufficient to explain the slightly larger difference between Prime and Average female masses at the 6 day timespan treatment at the high food level (compared to the 6 day timespan at the low food level).

S34 Table. Means (SE) for Prime female mass and age and Average female mass for the interaction FxT. Total food, expected values, growth rates and the differences between Prime and Average female masses.

| Food x Timespan | Prime female mass at pupation (mg) | Prime female age at pupation (days) | Average female mass at pupation (mg) | Estimated growth rate (mg/day) | Prime female mass MINUS Average female mass (mg) | Total food after day 4 (mg) | Expected mean values for Prime female mass (mg) | Expected mean values for Average female mass (mg) |
| --- | --- | --- | --- | --- | --- | --- | --- | --- |
| 16 mg, 3 days | 3.71 (0.82) | 6.38 (0.67) | 3.53 (0.84) | 0.58 (0.56) | 0.18 (0.59) | 16 | 3.83 (0.77) | 3.63 (0.81) |
| 16 mg, 6 days | 3.18 (0.53) | 8.28 (1.68) | 2.95 (0.53) | 0.38 (0.95) | 0.23 (0.37) | 8, 12 | 3.57 (0.77) | 3.34 (0.81) |
| 32 mg, 3 days | 4.70 (0.17) | 5.49 (0.22) | 4.52 (0.26) | 0.86 (0.14) | 0.18 (0.16) | 32 | 4.34 (0.77) | 4.13 (0.81) |
| 32 mg, 6 days | 4.21 (0.54) | 6.09 (0.66) | 3.94 (0.69) | 0.69 (0.46) | 0.27 (0.44) | 16, 24 | 4.08 (0.77) | 3.84 (0.81) |

S35 Table shows the means and standard errors for the FxT interaction for the Prime male mass and age at pupation and the Average male mass at pupation. This interaction explains a relatively large amount of the variance in the two mass variables (9% for Prime male mass and 8% for Average male mass; 3-4 times the amount explained by this contrast for the female mass variables). This interaction is significant for Prime male age at pupation and accounts for 2% of the variance in the ANOVA for this variable. A heuristic 3D plot of the Prime male mass and age at pupation is presented in S27 Fig.

1. For all three variables, the outcomes at the high food level are better than the outcomes at the low food level, so food is more important than timespan in this interaction.
2. For the Prime male mass the best outcome is in the high food, 3 day timespan treatment (2.75 mg) and the worst outcome is in the low food, 6 day timespan treatment (1.88 mg). These are the same high and low treatments as for the Prime female mass. The Prime males pupate earliest in the high food, 6 day timespan treatment; this is different from the Prime females. The Prime males pupate latest in the low food, 6 day timespan treatment (the same as the Prime females), but these males still pupate before the final aliquot of food is delivered on day 6.
3. There is very little difference in the Prime male age at pupation across these treatment combinations (0.35 day between the earliest and the latest mean ages), but there are large differences in the Prime male mass at pupation (0.87 mg between the largest and smallest; the largest Prime male is almost half again larger than the smallest).
4. The Prime males in both high food level treatments grow larger and pupate earlier than the Prime males at the low food level.
5. The Prime males at the low food, 3 day timespan grow almost as large and pupate almost as soon as the Prime males at the high food level.
6. There is a larger gap in both mass and age between the Prime males in the low food, 6 day timespan (the worst outcome) and the three other treatments. At the low food level, the Prime male in the 6 day timespan is 0.50 mg smaller and 0.24 days later at pupation compared to the 3 day timespan. The difference between the low food, 3 day timespan and the high food, 3 day timespan is only 0.27 mg and 0.11 day. The Prime males in the low food, 6 day timespan treatment do much worse than the other three treatments. This is likely one asymmetry that causes this interaction.
7. The columns, “Expected mean values for Prime male mass,” “Expected mean values for Prime male age,” and “Expected mean values for Average male mass,” report the projected mean values based on the main effects of food and timespan (see the explanation for S27 Table). For the Prime male mass and the Average male mass, the observed range of values is larger than the projected range (Prime male mass, observed, 1.88 mg to 2.75 mg, projected, 2.17 mg to 2.60 mg; Average male mass, observed, 1.92 mg to 2.61 mg, projected, 2.15 mg to 2.49 mg). This is similar to the pattern for Prime female and Average female masses.
8. Also similar, the stepwise increments between the food levels (explained for females, above) are present for males, although not as regular, and the projected increments are about half of the value of the observed increments, so the observed values are higher at the high food levels and lower at the low food levels, as for the females.
9. The projected ages at pupation for the Prime males don’t match the order of observed ages at pupation for the Prime males. The projected ages for the Prime males are in the same order as the observed ages at pupation for Prime females (S34 Table); the age at pupation for Prime females is not significantly affected by this interaction, suggesting that the mismatch of the order among Prime males is one of the sources of this interaction.
10. The observed ages at pupation for the Prime males deviate in both order and range from the projections. The largest Prime males are in the test tubes with the most food and delay pupation slightly (5.04 days) presumably to grow larger on the abundant food (32 mg of food, see the explanation of food levels for females above).
11. The second largest Prime males are in test tubes with somewhat less food (16 mg-24 mg of food) and pupate on day 5.
12. The third largest Prime males are in test tubes with even less food (16 mg of food); they pupate somewhat later (5.11 days).
13. The smallest Prime males have the least food (8 mg-12 mg of food); they pupate latest (5.35 days), but unlike the Prime females, they are able to pupate on less than 16 mg of food.
14. Prime males may delay pupation at the highest food level (32 mg of food) to grow larger and may delay pupation at low food levels to grow as large as possible. At the lower food levels (16 mg and below) Prime males seem to be food limited; they are smaller and pupate later as the food level decreases.
15. Growth rate combines the Prime male mass and age at pupation into a single biologically meaningful number. The two variables differ in which treatment results in the best outcome, but the growth rate is highest for the high food, 3 day timespan treatment, which matches the Prime male mass. (This interaction explains about 4x more variance in the Prime male mass than in the Prime male age at pupation.) There does not seem to be any asymmetry in the growth rate that is not related to the Prime male mass.
16. For the Average male mass the best outcome is also in the high food, 3 day timespan treatment (2.61 mg) and the worst outcome is in the low food, 6 day timespan treatment (1.92 mg; this is 0.04 mg larger than the Prime male mass in this treatment). Non-Prime males benefit from the final aliquot of food on the 6th day.
17. However, the difference in size between the largest and smallest Average males is 0.69 mg and the difference between the worst outcome and the next smallest is 0.38 mg (more than half of the overall range). The difference between the three best outcomes (low food, 3 day timespan and the two high food treatments) is 0.31 mg. The Average males are more similar in size across the treatments than the Prime males.
18. The range of sizes of Average males is larger than the range of projected sizes based on the main effects, but it is smaller than the range of sizes of the Prime male.
19. The difference between the Prime male and the Average male is a measure of the size distribution of the larvae and also of the relative dominance of the Prime over the non-Prime males.
20. The difference between the Prime and Average in the high food, 3 day timespan treatment (the largest Prime males) is 0.14 mg; this is the largest difference across these four treatments. The food may be abundant for the Prime male, but the non-Prime males are relatively smaller than in the other three treatments.
21. The next largest Prime males (high food, 6 day timespan) are only 0.08 mg larger than the Average male mass. This could be due to the non-Prime males growing further on the additional food on day 6.
22. However, the Prime male in the low food, 3 mg timespan treatment is also 0.08 mg larger than the Average male mass. This can’t be due to a late addition of food, but could be due to the release of food by the pupation of the Prime male, or it could be due to the larger females changing their feeding behavior from filtering to retention because the abundance of particles is low, and retention further reduces the abundance of particles and causes a tighter distribution of sizes among the larvae.
23. Finally, the smallest Prime males in the low food, 6 day timespan treatment are 0.04 mg smaller than the Average male mass. This is clearly due to the additional food on day 6, but could also be affected by the Prime male pupating and/or a change in the behavior of the females (from retention to filtering, after the addition of the final aliquot of food on day 6).
24. For various possible reasons, the range of the Average male masses is more compressed, at both ends, than that of the Prime male mass. The difference between the largest Prime male and the next largest is 0.22 mg; the corresponding difference for the Average male is 0.16 mg. The difference between the smallest Prime male and the next smallest is 0.50 mg. The corresponding difference for the Average male is 0.38 mg. The difference between the middle two Prime male masses is 0.15 mg, and the same for the middle two Average male masses.
25. Prime males experience a food environment that is somewhat different from that experienced by the non-Prime males. The main difference is that the non-Prime males benefit from increased food after the Prime males pupate, and from increased food and a possible change in female feeding behavior after the addition of the final aliquot on day 6.
26. The difference between the Prime female mass and the Prime male mass is a measure of the relative advantage of the Prime female over the Prime male (S36 Table). The difference between the Average female mass and the Average male mass describes the relative advantage of females over males. For both comparisons the difference is greatest in the high food, 3 day timespan where all four mass variables are largest, and least in the low food, 6 day timespan treatment where all four mass variables are smallest.
27. For the difference between the Prime female and the Prime male, the values at the low food level are almost the same (1.33 mg and 1.30 mg, for the 3 day and 6 day timespan, respectively). The values of the difference are higher and divergent at the high food level (1.95 mg and 1.68 mg, for 3 days and 6 days, respectively). This might reflect the feeding behavior of the females at low food level (retention), compressing the size distribution of the males as well as the females.
28. For the difference between the Average female mass and the Average male mass, the values at the low food level are lower at the 6 day timespan treatment (1.03 mg) than at the 3 day timespan treatment (1.23 mg). In this treatment the non-Prime males grow larger than the Prime males after the Prime males pupate, so the smaller difference between the Average female mass and the Average male mass is due to the growth of the non-Prime males. This suggests that the non-Prime females do not grow disproportionately larger on the final aliquot of food.
29. The differences between the Average individuals span a larger range (1.03 mg to 1.91 mg, a span of 0.88 mg) than that of the Prime individuals (1.30 mg to 1.95 mg, a span of 0.65 mg). At high food and the 3 day timespan, all four mass variables are greatest across this interaction, and the difference between the Prime individuals is only a little higher than that between the Averages (1.95 mg compared to 1.91 mg). At low food and the 6 day timespan, all four mass variables are smallest across the interaction, and the differences between the Prime individuals (1.30 mg) and between the Average individuals (1.03 mg) are also smallest, but the Average difference is much smaller than the Prime. The difference in span between the Average individuals and the Prime individuals is primarily due to the low food, 6 day timespan treatment. There are differences in the outcomes between males and females and Prime and non-Prime individuals, but the low food, 6 day timespan treatment is disproportionately worse than the other three treatments for all larvae.

S35 Table. Means (SE) for Prime male mass and age and Average male mass for the interaction FxT. Expected values, growth rates and the differences between Prime and Average female masses.

| Food x Timespan | Prime male mass at pupation (mg) | Prime male age at pupation (days) | Average male mass at pupation (mg) | Estimated growth rate (mg/day) | Prime male mass MINUS Average male mass (mg) | Expected mean values for Prime male mass (mg) | Expected mean values for Prime male age (days) | Expected mean values for Average male mass (mg) |
| --- | --- | --- | --- | --- | --- | --- | --- | --- |
| 16 mg, 3 days | 2.38 (0.30) | 5.11 (0.08) | 2.30 (0.36) | 0.47 (0.16) | 0.08 (0.23) | 2.35 (0.44) | 5.15 (0.24) | 2.28 (0.39) |
| 16 mg, 6 days | 1.88 (0.51) | 5.35 (0.43) | 1.92 (0.42) | 0.35 (0.33) | -0.04 (0.33) | 2.17 (0.44) | 5.20 (0.24) | 2.15 (0.39) |
| 32 mg, 3 days | 2.75 (0.05) | 5.04 (0.07) | 2.61 (0.07) | 0.55 (0.05) | 0.14 (0.04) | 2.60 (0.44) | 5.05 (0.24) | 2.49 (0.39) |
| 32 mg, 6 days | 2.53 (0.29) | 5.00 (0.00) | 2.45 (0.35) | 0.51 (0.15) | 0.08 (0.23) | 2.42 (0.44) | 5.10 (0.24) | 2.36 (0.39) |

S36 Table. Means (SE) for Prime male mass and age and Average male mass for the interaction FxT. Differences between the Prime female mass and Prime male mass and between the Average female mass and the Average male mass.

| Food x Timespan | Prime male mass at pupation (mg) | Prime male age at pupation (days) | Average male mass at pupation (mg) | Prime female mass MINUS Prime male mass (mg) | Average female mass MINUS Average male mass (mg) |
| --- | --- | --- | --- | --- | --- |
| 16 mg, 3 days | 2.38 (0.30) | 5.11 (0.08) | 2.30 (0.36) | 1.33 (0.44) | 1.23 (0.46) |
| 16 mg, 6 days | 1.88 (0.51) | 5.35 (0.43) | 1.92 (0.42) | 1.30 (0.37) | 1.03 (0.34) |
| 32 mg, 3 days | 2.75 (0.05) | 5.04 (0.07) | 2.61 (0.07) | 1.95 (0.09) | 1.91 (0.13) |
| 32 mg, 6 days | 2.53 (0.29) | 5.00 (0.00) | 2.45 (0.35) | 1.68 (0.31) | 1.49 (0.39) |

**FxT summary**

Males and females both grow according to the food level in the test tubes, but this food level is modified by the timespan, and significantly affects the growth of both sexes. Females do not pupate until 16 mg have been added to the test tubes (at the end of the timespan for the low food level); males do pupate on less food. Females grow larger in stepwise increments as the amount of food increases; males also grow larger, but the increments are smaller and less regular. The overall residual effect in this interaction is the almost linear relationship between total food after day 4 and the mass of males and females (S28 Fig). Both females and males are larger on the high food, 3 day timespan than projected by the main effects, and smaller than projected in the low food, 6 day timespan. The range of sizes of the Average females across treatments is slightly larger than that of the Prime females. The range of sizes of the Average males is compressed relative to that of the Prime males. Females may change their feeding behavior in response to the food inputs across the timespan, producing different size distributions. Most Prime females pupate on day 6; the Prime females in the low food, 6 day timespan pupate 2.28 days later. Most Prime males pupate on day 5; none appear to pupate late enough to take advantage of the day 6 food input. Non-Prime males do grow larger after the Prime males pupate; in some cases they grow larger than the Prime males. Females also grow larger on the late addition of food on day 6, and this may increase the size distribution suggesting that the females switch from retention to active filtering when the last aliquot of food is added.

This interaction describes the growth of larvae rather than competition. Males actively filter particles throughout their larval period. Females in the low food levels switch to retention at some point as the number and quality of particles decreases relatively to demand. The Prime male grows at the expense of the non-Prime males and pupates in response to unknown triggers that may include size, physiological status, age, and environmental cues (availability of food particles). In the 6 day timespan treatments, where the worst outcomes occur for all the mass and age variables, the Prime male grows as large as possible on the total food before day 6 and pupates before the addition of the final aliquot. A difference between the low food, 6 day timespan treatment and the other three treatments is that the Prime female pupates 2.28 days after the final aliquot is added on day 6. All the females and the non-Prime males grow larger on this final food input. The females and the non-Prime males experience a benefit when the additional food is added on day 6 and grow larger. Some non-Prime males grow to be larger than the Prime male on the extra food. The females are larger than the males, and also grow larger, but the incremental food is less valuable than the same amount of food earlier in the larval period (for instance, the 3 day timespan). None of the larvae in the low food, 6 day timespan treatment reach the size of corresponding larvae in the low food, 3 day timespan treatment despite the extra food and longer larval periods. The estimated growth rate of the Prime female (0.38 mg/day) is only a little higher than that of the Prime male (0.35 mg/day) despite taking almost 3 days longer to feed and grow.

In contrast to the mass and age variables, Survival is best in the test tubes with high food and the 6 day timespan. This treatment is intermediate for all the mass and age variables with the exception of Prime male age at pupation. Prime males pupate earliest in this treatment. Survival is worst in the test tubes with high food and the 3 day timespan. This is the treatment with the best outcomes for the four mass variables, and the fastest growth rates, indicating that these test tubes promote optimal growth for mosquito larvae across this interaction. The high food level delivered by day 3 may affect Survival by overwhelming the larvae with too much food early in the larval growth period.

**The AxT interaction (R squared = 0.51)**

This interaction between aliquot and timespan represents the residual effect of these factors after the higher level interactions have been resolved (FxAxT for Prime female mass and Average female mass, FxDxAxT for Average female mass, DxAxT for Average male mass and Prime female mass, below). This interaction is significant in the ANOVA for the four mass variables and the Prime male age at pupation. In the MANOVA the coefficients for Prime male age at pupation, Average male mass at pupation and Average female mass at pupation are large and positive, but the Prime male mass and Prime female mass are both close to zero. This contrast ranks in the middle of the significant interaction contrasts, explaining 3% of the variance for the Prime and Average female masses, 1% of the Prime male age at pupation, and 5% and 6% of the Prime and Average male masses, respectively. The main effects of aliquot and timespan are: for the mass variables, 4 aliquots are better than 2 aliquots and the 3 day timespan is better than the 6 day timespan; for the Prime male age at pupation, 2 aliquots are better (earlier) than 4 aliquots and the 3 day timespan is better than the 6 day timespan.

S37 Table shows the means and standard errors for this interaction for the Prime female mass and age at pupation and for the Average female mass at pupation. The interaction is not significant for the Prime female age at pupation variable, but it is useful to consider for estimated growth rates. Overall, the 3 day timespan produces better outcomes than the 6 day timespan for all variables, so timespan is more important that aliquot in this interaction. Heuristic 3D plots of the Prime female mass and age and Prime male mass and age are presented in S29 Fig and S30 Fig, respectively.

1. The largest value of the Prime female mass is in the test tubes with 4 aliquots and the 3 day timespan (4.22 mg), and the smallest value is in those with 2 aliquots and the 6 day timespan (3.43 mg). Both 3 day timespan treatments are similar in size and larger than either of the 6 day timespan treatments. There is only a small difference in size between the 2 aliquot treatment and the 4 aliquot treatment at the 3 day timespan (0.03 mg for the Prime female mass). There is a larger difference between the 2 aliquot treatment and the 4 aliquot treatment at the 6 day timespan (0.53 mg for the Prime female mass).
2. The interaction arises from the disproportionately small masses in the 2 aliquot, 6 day timespan treatment. The Prime females are larger at the 3 day timespan than at the 6 day timespan, and aliquot has little effect on the size of the Prime females at the 3 day timespan, but a very large effect on the size of the Prime females at the 6 day timespan.
3. The Prime female age at pupation is not affected by this interaction, but it is affected by the main factors, aliquot and timespan. The estimated growth rate combines the age and mass variables into a single biologically meaningful number. The same pattern is apparent for the estimated growth rates of the Prime female as evidenced by the Prime female mass; these are similar for the treatments with the 3 day timespan, lower for the 4 aliquot, 6 day timespan and lowest for the 2 aliquot, 6 day timespan. There is no asymmetry other than that already indicated for the Prime female mass.
4. For Average female mass the largest value is in the test tubes with 4 aliquots and the 3 day timespan (4.06 mg), and the smallest value is in those with 2 aliquots and the 6 day timespan (3.13 mg); this is the same as for the Prime female mass.
5. There is only a small difference in size between the 2 aliquot treatment and the 4 aliquot treatment at the 3 day timespan (0.07 mg). There is a larger difference between the 2 aliquot treatment and the 4 aliquot treatment at the 6 day timespan (0.64 mg). As for the Prime female mass, the interaction is caused by the much lower value of the Average female mass at the 2 aliquot, 6 day timespan with respect to the other three treatments.
6. The columns, “Expected mean values for Prime female mass” and “Expected mean values for Average female mass,” report the projected means based on the main effects, aliquot and timespan (see explanation of S27 Table). The expected values for the 3 day timespan treatments are higher than those for the 6 day timespan in the projections, but all of the projected means are lower than the observed means except for the 2 aliquot, 6 day treatment. It is the worst outcome in the projections, but the observed values of the means are much lower than the expected values for both Prime female mass and Average female mass.
7. The observed ranges for both Prime and Average female masses are greater than the projected ones (Prime female mass, observed, 3.43 mg to 4.22 mg, a span of 0.79 mg; projected, 3.75 mg to 4.15 mg, a span of 0.40 mg; Average female mass, observed, 3.12 mg to 4.06 mg, a span of 0.94 mg; projected, 3.50 mg to 3.97 mg, a span of 0.47 mg). However, the difference between the largest observed value and the largest projected value (both at 4 aliquots and the 3 day timespan) is only 0.07 mg for the Prime female and 0.09 mg for the Average female.
8. The asymmetries that cause these interactions are the much lower values in the worst treatment, 2 aliquots and the 6 day timespan: 0.32 mg lower for the Prime female and 0.62 mg lower for the Average female. Both Prime and Average females do much worse at the 2 aliquot, 6 day timespan treatment than expected. The obvious reason for this is that there is less food in these test tubes than in any of the others; the first aliquot of food is delivered on day 0 and no more food is delivered until after pupae are collected on day 6. The Prime females pupate almost 2 days after the final aliquot is delivered, but do not grow as large as any of the Prime females in other treatments. This is an effect of the distribution of food in time independent of the food level, density, or competition (food x density) factors.
9. The size distribution of the females indicates the relative advantage that the Prime female has over the non-Prime females. The difference between the mass of the Prime female and that of the Average female is smallest in the test tubes where the females grow largest (4 aliquots, 3 day timespan) and greatest in the test tubes where the females are smallest (2 aliquots, 6 day timespan). This is the opposite of the effect due to exponential growth processes.
10. The large size of the Prime female corresponds to the fastest growth rate and the most abundant food (more aliquots, delivered in 3 days). The small difference between the Prime and Average female mass (0.16 mg) indicates that all the females benefit from this food delivery scheme.
11. The small size of the Prime female in the 2 aliquot, 6 day timespan treatment corresponds to the slowest growth rate and the least abundant food (one aliquot, delivered on day 0, followed by a second aliquot delivered on day 6). The larger difference between the Prime and Average female mass (0.31 mg) indicates that all females suffer from the low apparent food, but that the non-Prime females suffer more than the Prime.
12. This could be an indication of interference competition (smaller sizes with a larger size distribution), except that density (and competition) is not part of this interaction.
13. Regardless of food level or density or competition (food x density), the temporal distribution of the food inputs (aliquot x timespan) affects the growth of all females similarly.
14. Multiple smaller inputs delivered earlier in the larval life cycle are best. Fewer inputs and later delivery of inputs both reduce the size and increase the distribution of sizes of the females.
15. The temporal distribution of food affects the actual availability of food. Furthermore, the value of identical aliquots of food decreases as the timespan increases, so late additions do not increase the size of females as much as the same input delivered earlier in the larval lifespan.

S37 Table. Means (SE) for Prime female mass and age at pupation and Average female mass at pupation for the interaction AxT. Expected values, growth rates and the differences between the Prime female mass and the Average female mass.

| Aliquot x Timespan | Prime female mass at pupation (mg) | Prime female age at pupation (days) | Average female mass at pupation (mg) | Estimated growth rate for Prime females (mg/day) | Prime female mass MINUS Average female mass (mg) | Expected mean values for Prime female mass at pupation (mg) | Expected mean values for Average female mass at pupation (mg) |
| --- | --- | --- | --- | --- | --- | --- | --- |
| 2 aliquots, 3 days | 4.19 (0.85) | 5.95 (0.94) | 3.99 (0.85) | 0.70 (0.09) | 0.20 (0.63) | 4.01 (0.77) | 3.79 (0.81) |
| 2 aliquots, 6 days | 3.43 (0.59) | 7.73 (2.14) | 3.12 (0.65) | 0.44 (0.07) | 0.31 (1.11) | 3.75 (0.77) | 3.50 (0.81) |
| 4 aliquots, 3 days | 4.22 (0.80) | 5.92 (0.37) | 4.06 (0.84) | 0.71 (0.07) | 0.16 (0.44) | 4.15 (0.77) | 3.97 (0.81) |
| 4 aliquots, 6 days | 3.96 (0.87) | 6.64 (1.05) | 3.76 (0.85) | 0.60 (0.08) | 0.20 (0.68) | 3.89 (0.77) | 3.68 (0.81) |

S38 Table shows the means and standard errors for Prime male mass and age at pupation and Average male mass at pupation for the AxT interaction. The r squared values for the male mass variables are about twice as large as for the female mass variables; this interaction explains 5% of the variance in the experiment for Prime male mass and 6% for the Average male mass. For the Prime male mass and age at pupation, this is the highest level interaction between these two factors. Timespan is more important than aliquot for all three variables; the outcomes at the 3 day timespan are all better than any at the 6 day timespan.

1. The Prime male mass and Average male mass are largest in the 2 aliquot, 3 day timespan treatment. However, the Prime and Average male masses are similar at the 3 day timespan in both aliquot treatments (Prime male mass at 2 aliquots, 3 day timespan is 2.57 mg, at 4 aliquots, 3 day timespan is 2.55 mg; Average male mass at 2 aliquots, 3 day timespan is 2.47 mg, at 4 aliquots, 3 day timespan is 2.44 mg). There is little effect of aliquot on either the Prime male mass or the Average male mass at the 3 day timespan. The 4 aliquot, 6 day timespan treatment produces Prime and Average males that are only a little smaller than the 3 day timespan treatments (2.41 mg and 2.38 mg respectively). The Prime and Average males are smallest in the 2 aliquot, 6 day timespan treatment (2.00 mg and 1.98 mg, respectively).
2. Similarly to the female mass variables, the male mass variables are disproportionately small in the 2 aliquot, 6 day timespan treatment combination, giving rise to the interaction.
3. The Prime male age at pupation is earliest at 4 aliquots and the 3 day timespan, and latest at 4 aliquots and the 6 day timespan. The two 3 day timespan treatments pupate at similar times (5.07 days and 5.08 days) and the 2 aliquot, 6 day timespan treatments pupate next (5.13 days) followed by the 4 aliquot, 6 day timespan treatment (5.22 days). This is a different asymmetry than that of the mass variables.
4. The columns, “Expected mean values for Prime male mass at pupation,” “Expected mean values for the Prime male age at pupation,” and “Expected mean values for the Average male mass at pupation,” represent the projected values based on the main effects of aliquot and timespan (see explanation for S27 Table). The patterns for the projected Prime and Average male mass deviate from the observed in five ways: first, the observed highest values of mass are in the 2 aliquot, 3 day timespan treatment, but the projected highest values are in the 4 aliquot, 3 day timespan; second, the projected values are a close match to the observed values in the 4 aliquot, 3 day timespan treatment, this is the only treatment where the projected and observed masses are close; third, the observed high values are higher than the projected values in the 2 aliquot, 3 day timespan (0.13 mg for both Prime and Average male mass); fourth, the observed range of values for both Prime and Average male mass is twice the projected range (Prime male mass, observed, 2.00 mg to 2.57 mg, a span of 0.57 mg, projected, 2.24 mg to 2.52 mg, a span of 0.28 mg; Average male mass, observed, 1.98 mg to 2.47 mg, a span of 0.49 mg, projected, 2.21 mg to 2.43 mg, a span of 0.22 mg); fifth, the difference between the observed and expected values for the worst outcome (2 aliquots, 6 day timespan) accounts for almost half of the range for both Prime and Average male mass (0.24 mg for the Prime male mass; 0.23 mg for the Average male mass). All of these deviations contribute to the significant interaction, but the last one seems likely to be the largest contributor.
5. For Prime male age at pupation the range of the observed values is also twice the range of the projected values (observed, 5.07 days to 5.22 days, a span of 0.15 days; projected, 5.09 days to 5.16 days, a span of 0.07 days). The two 3 day timespan treatments are the earliest to pupate in the projections and as observed, but the actual age for both is lower than either projection. The latest age at pupation is in the 4 aliquot, 6 day timespan (both observed and projected), but is 0.06 days later than projected (again almost half of the range).
6. The estimated growth rate combines the Prime male mass and age into one biologically meaningful number. Both mass and age are significantly affected by this interaction and the worst outcomes for each occur in different treatments (2 aliquots, 6 day timespan for the Prime male mass; 4 aliquots, 6 day timespan for the Prime male age). The growth rates for the two 3 day timespan treatments are almost the same; the larger mass in the 2 aliquot, 3 day timespan treatment results in the faster growth rate than in the 4 aliquot, 3 day timespan treatment despite the slightly later age at pupation. The third place growth rate is associated with the third place mass despite the latest age at pupation. The smallest mass produces the lowest growth rate despite an earlier age at pupation (third ranked). The growth rates follow the Prime male mass; this interaction affects the mass 5 times more than the Prime male age.
7. The fastest growth rate is associated with the largest mass and a slightly later age at pupation than the second fastest growth rate, but the growth rates are more similar than the masses.
8. All the Prime males pupate before the end of the 6 day timespan, so the ones in the 6 day timespan treatments pupate on the first aliquot (of 2 aliquots) or the first 3 aliquots (of 4 aliquots). The Prime males in both 6 day timespan treatments delay their pupation to grow larger, but the ones with more food (3 aliquots instead of 1 aliquot) delay longer and grow larger.
9. The Prime male age at pupation is earliest in the test tubes with the 3 day timespan (not very different across the aliquot treatments) and the estimated growth rate of the Prime male is also fastest and very similar in these test tubes. The Prime male age at pupation is latest in the test tubes with 4 aliquots and the 6 day timespan, but the estimated growth rate is slowest in the test tubes with 2 aliquots and the 6 day timespan. The much smaller mass of the Prime male in the 2 aliquot, 6 day timespan treatment combination offsets the somewhat earlier age at pupation to reduce the estimated growth rate.
10. The difference between the Prime male mass and the Average male mass indicates changes in the size distribution of males and the relative advantage of the Prime male over the non-Prime males; it is almost entirely affected by the timespan.
11. The difference between the Prime and Average males is larger with the 3 day timespan (0.10 mg to 0.11 mg) than with the 6 day timespan (0.02 mg to 0.03 mg). Both Prime and Average males are larger in the test tubes with the 3 day timespan, so this is possibly due to exponential growth processes. All the Prime males pupate before the addition of the final aliquot on the 6th day. In the 3 day timespan treatments, all the food has been added by the 3rd day and the Prime and Average male masses are larger than in the 6 day timespan treatments. In the 6 day timespan treatments some of the non-Prime males grow larger on the final aliquot and the mass of the Average males is more similar to that of the Prime males.

S38 Table. Means (SE) for Prime male mass and age at pupation and Average male mass at pupation for the interaction AxT. Expected values, growth rates and the differences between the Prime male mass and the Average male mass.

| Aliquot x Timespan | Prime male mass at pupation (mg) | Prime male age at pupation (days) | Average male mass at pupation (mg) | Estimated growth rate for Prime males (mg/day) | Prime male mass MINUS Average male mass (mg) | Expected mean values for Prime male mass at pupation (mg) | Expected mean values for Prime male age at pupation (days) | Expected mean values for Average male mass at pupation (mg) |
| --- | --- | --- | --- | --- | --- | --- | --- | --- |
| 2 aliquots, 3 days | 2.57 (0.33) | 5.08 (0.10) | 2.47 (0.33) | 0.51 (0.03) | 0.10 (0.17) | 2.42 (0.44) | 5.09 (0.24) | 2.34 (0.39) |
| 2 aliquots, 6 days | 2.00 (0.53) | 5.13 (0.25) | 1.98 (0.41) | 0.39 (0.05) | 0.02 (0.29) | 2.24 (0.44) | 5.14 (0.24) | 2.21 (0.39) |
| 4 aliquots, 3 days | 2.55 (0.27) | 5.07 (0.08) | 2.44 (0.29) | 0.50 (0.03) | 0.11 (0.14) | 2.52 (0.44) | 5.11 (0.24) | 2.43 (0.39) |
| 4 aliquots, 6 days | 2.41 (0.48) | 5.22 (0.45) | 2.38 (0.46) | 0.46 (0.05) | 0.03 (0.33) | 2.34 (0.44) | 5.16 (0.24) | 2.30 (0.39) |

**Males and females**

S39 Table presents the Prime female mass at pupation alongside the differences between the Prime female mass and the Average female mass (from S37 Table) and the differences between the Prime male mass and the Average male mass (from S38 Table) with the differences between the Prime female mass and the Prime male mass, and the Average female mass and the Average male mass. The total food after day 4 and the food/larva after day 4 are included as well. The differences between the masses indicate the size distribution of the pairs of variables and the relative advantage of the larger over the smaller. The interaction involves the distribution of food over time (aliquot, timespan) so each contrast includes both food levels (16 mg, 32 mg) and both densities (4 larvae, 8 larvae). This results in two total food levels within each contrast and 4 food/larva levels within each contrast. The 4 food/larva levels are presented as an average in the last column.

1. The difference between the Prime female mass and the Prime male mass reflects the advantage that the Prime female has over the Prime male in the various treatments. The difference between the Average female mass and the Average male mass reflects the analogous advantage that females have over males. The females grow largest in the 4 aliquot, 3 day timespan treatment, while the males grow largest in the 2 aliquot, 3 day timespan treatment, but in both cases the difference between the two 3 day timespan treatments is small.
2. The difference between the Prime female and Prime male masses is largest in the 4 aliquot, 3 day timespan treatment (1.67 mg); this treatment produces the highest value of the Prime female mass and the second highest value of the Prime male mass. The difference between the Prime female and Prime male in the 2 aliquot, 3 day timespan is lower (1.62 mg); this treatment produces the second highest value of the Prime female mass and the highest value of the Prime male mass. The differences between the Average female mass and the Average male mass follow the same pattern (1.62 mg and 1.52 mg, respectively). The values of all four mass variables rank third in the 4 aliquot, 6 day treatment and the difference between the Prime female and Prime male (1.55 mg), and between the Average female and the Average male (1.38 mg), also rank third. The values of all four mass variables are smallest in the 4 aliquot, 6 day treatment and the difference between the Prime female and Prime male (1.43 mg), and between the Average female and the Average male (1.14 mg), also are smallest.
3. The difference between the female and male masses decreases as the size of the females decreases (both Prime and Average females), but the gap between the differences increases as the size decreases. For the difference between the Prime female and the Prime male, the gaps between the four treatments are: 0.05 mg, 0.07 mg, and 0.12 mg. For the difference between the Average female and the Average male, the gaps are: 0.10 mg, 0.14. mg, and 0.24 mg. The gaps and the total span of these differences are double for the Average individuals compared to the Prime. This regularity is unexpected because the difference between the Prime male and Average male is only affected by the timespan, while the difference between the Prime female and Average female is smallest when the females are largest (4 aliquots, 3 day timespan) and largest in the test tubes where the females are smallest (2 aliquots, 6 day timespan).
4. The Prime female grows largest and has the greatest advantage over the Prime male in the 4 aliquot, 3 day timespan treatment. This suggests that the food supply is best in this treatment combination. The Prime female is slightly smaller in the 2 aliquot, 3 day timespan treatment and has a slightly smaller advantage over the Prime male (0.05 mg smaller). The Prime female is even smaller in the 4 aliquot, 6 day timespan treatment and has an even smaller advantage over the Prime male (0.07 mg smaller). The Prime female is much smaller in the 2 aliquot, 6 day timespan treatment and has the smallest relative advantage over the Prime male (0.12 mg smaller). As the aliquot and timespan treatments restrict the food supply (independently of the actual food level or density), both the Prime female and the Prime male get smaller, but the difference between them gets smaller as well. The same relationship is true for the Average female mass and the Average male mass, but the effect is twice that for the Prime individuals. The relative advantage of the females over the males decreases as the food supply is restricted by the factors aliquot and timespan.

S39 Table. Means (SE) for Prime female mass for the interaction AxT. Differences between Prime female mass and Average female mass, Prime male mass and Average male mass, Prime female mass and Prime male mass, and Average female mass and Average male mass. Total food after day 4 and food/larva after day 4.

| Aliquot x Timespan | Prime female mass at pupation (mg) | Prime female mass MINUS Average female mass (mg) | Prime male mass MINUS Average male mass (mg) | Prime female mass MINUS Prime male mass (mg) | Average female mass MINUS Average male mass (mg) | Total food after day 4 (mg) | Food/larva after day 4 (mg) | Average food/larva after day 4 (mg) |
| --- | --- | --- | --- | --- | --- | --- | --- | --- |
| 2 aliquots, 3 days | 4.19 (0.85) | 0.20 (0.63) | 0.10 (0.17) | 1.62 (0.46) | 1.52 (0.46) | 16, 32 | 4, 2, 8, 4 | 4.5 |
| 2 aliquots, 6 days | 3.43 (0.59) | 0.31 (1.11) | 0.02 (0.29) | 1.43 (0.40) | 1.14 (0.38) | 8, 16 | 2, 1, 4, 2 | 2.25 |
| 4 aliquots, 3 days | 4.22 (0.80) | 0.16 (0.44) | 0.11 (0.14) | 1.67 (0.42) | 1.62 (0.44) | 16, 32 | 4, 2, 8, 4 | 4.5 |
| 4 aliquots, 6 days | 3.96 (0.87) | 0.20 (0.68) | 0.03 (0.33) | 1.55 (0.50) | 1.38 (0.48) | 12, 24 | 3, 1.5, 6, 3 | 3.375 |

Females change their feeding behavior from actively filtering particles to retaining particles in their gut in response to the food level. In this interaction, both food levels are represented in each treatment combination, so this behavior does not explain the change in relative advantage of the females over the males.

Prime males pupate earlier and at a smaller size and a more compressed size distribution than Prime females, and three of these Prime males pupate at sizes ranging from 2.41 mg to 2.57 mg (0.16 mg), but the three largest Prime females pupate at 3.96 mg to 4.22 mg (0.24 mg), so this doesn’t explain the difference in relative advantage either. The ranges for the Average females and Average males are similar.

The difference between the Prime female and the Average female is greatest at 2 aliquots and the 6 day timespan where they are both smallest. This is where the Prime male and the Average male are also smallest, but the difference between them is least. The Prime female monopolizes the initial food input on day 0 and reduces the size of the non-Prime females and all the males, then the non-Prime males grow larger release after the Prime male pupates and on the day 6 food input.

The difference between the Prime and Average female masses is similar across the other three treatments, although it is slightly lower for the 4 aliquot, 3 day timespan treatment where both Prime and Average female masses are highest, indicating the best food supply for this interaction. The Prime and Average male masses are higher in the 3 day timespan treatment and the difference between the Prime and Average male masses is similar across the aliquot treatments. This suggests that males grow better with a larger input on day 3 (2 aliquots and 3 day timespan) and females grow better with 4 aliquots (also the 3 day timespan). This explains the larger masses of males on the 2 aliquot, 3 day timespan and the larger masses of females on the 4 aliquot, 3 day timespan.

The larvae in the 4 aliquot, 6 day timespan treatment grow more slowly and attain a smaller final mass because the food supply is delivered over more time. The size distribution of females in the 4 aliquot, 6 day timespan treatment (the difference between the Prime and Average female masses) is similar to the size distributions of females in both the 3 day timespan treatments, although the Prime and Average masses are smaller in the 4 aliquot, 6 day timespan treatment. Prime males in the 4 aliquot, 6 day timespan treatment are smaller than the Prime males in both the 3 day timespan treatments, but the non-Prime males benefit from the early pupation of the Prime males and the additional food on day 6 as do the non-Prime males in the 2 aliquot, 6 day timespan treatment.

Males do better with a large input of food on day 3 while females do better with more smaller inputs. For males, the addition of half the food on the 3rd day increases the size of all males. This effect is small, but is opposite of the effect on females in this interaction. Females do better with multiple smaller inputs early in the larval period. Males do better when there is an influx of food later in the larval period. This difference is independent of food level, density, or competition (food x density).

The difference between the Prime female and the Average female, between the Prime female and the Prime male, and between the Average female and the Average male, are all smallest in the 4 aliquot, 3 day timespan treatment. The difference between the Prime male and Average male is largest in this treatment. The difference between the Prime female and the Average female, between the Prime female and the Prime male, and between the Average female and the Average male, are all largest in the 2 aliquot, 6 day timespan treatment. The difference between the Prime male and Average male is smallest in this treatment. Food level is not part of this interaction, but two attributes of the food supply are, and these affect the availability of food in the test tubes. 4 aliquots and the 3 day timespan provides the most food, earliest in the larval period. 2 aliquots and the 6 day timespan provides the least food early in the larval period; the final aliquot is added after the Prime males pupate, and the Prime females delay pupation for more than a day after the next latest treatment. At the highest food availability (4 aliquots, 3 day timespan) the females grow largest and fastest, the non-Prime females grow almost as large as the Prime females, the Prime males grow large (second largest and second fastest growth rate) and there is a large difference between the Prime and non-Prime males, suggesting exponential growth due to active filtering for abundant particles. At the next highest food availability (2 aliquots, 3 day timespan) the females grow almost as large, almost as fast, the non-Prime females are a little smaller relative to the Prime females, the males grow largest, the Prime males grow fastest, and there is also a large difference between the Prime and non-Prime males. The males do better relative to the females because of the large input of food on day 3 (compared to the multiple inputs that favor female growth). At the third highest food availability (4 aliquots, 6 day timespan) the females are smaller, grow more slowly, the non-Prime females are the same relative to the Prime females as in the second highest, the males are smaller, the Prime males pupate latest, and there is a small difference between the Prime and Average males. The change in the size distribution of the males, along with the smaller size of the females suggests that the females switch to retention from active filtering and reduce the availability of food even further than the aliquot and timespan treatment. The Prime males delay pupation to grow larger (on the third aliquot of food on day 4), but still pupate before the final aliquot on day 6. At the lowest food availability (2 aliquots, 6 day timespan) the females are smallest, grow slowest, pupate much later, the difference between the Prime and Average females is larger, the males are smaller and the difference between the Prime and Average males is smallest. The change in the size distribution of the males, along with the smaller size of the females again suggests that the females switch to retention from active filtering and reduce the availability of food even further than the aliquot and timespan treatment. The increase in the size distribution of the females suggests that they switch back to active filtering after the final aliquot is added. The non-Prime males may also grow larger on this added food. None of the females or non-Prime males grow as large as their counterparts in other treatments. This describes the significant interaction of the two attributes of food availability affecting the mass and age at pupation of males and females independently of food level, density, or competition (food x density).

**Density, aliquot and timespan**

The remaining interactions cross density with aliquot and timespan. Aliquot and timespan are aspects of the food supply independent of each other and of the food level. In the previous interactions, both aliquot and timespan, and their interaction, AxT, appear to alter the availability of food for the larvae. The interaction of density (4 larvae or 8 larvae per test tube) and food level (16 mg food per test tube and 32 mg food per test tube) produces 4 different competitive environments. These three interactions between density, aliquot and timespan may also result in different competitive environments, or they may reveal additional information about growth. Competition among larvae alters the mass and age of the Prime individuals, the growth rate of the Prime individual, the Average mass, and the difference between the Prime and the Average mass. Furthermore, the specific differences are sex-dependent. For females, the 4 aliquot, 3 day timespan appears to provide the largest amount of food, earliest in the larval period (at both food levels) and represents the best growth conditions in the AxT interaction (above). Also for females, the 2 aliquot, 6 day timespan provides the least amount of food early in the larval period and represents the worst growth conditions across this interaction. The 2 aliquot, 3 day timespan and the 4 aliquot, 6 day timespan are intermediate, but the expectation is that the 3 day timespan treatment will provide more food earlier than the 6 day timespan. High density should increase competition for food and it should have a disproportionate effect at the lower food supply (both food levels are present in each treatment combination, so this would be competition independent of the food level).

The 3-way interaction, DxAxT, has the smallest R squared value of any significant contrast in the MANOVA, but is the highest order interaction for these factors for the two dependent variables, Average male mass and Prime female mass. DxT is the second most significant interaction in the MANOVA (after FxDxT). It is significant for all 7 dependent variables in the ANOVAs (also like FxDxT). DxA is not significant in the MANOVA, but is significant for Prime female age at pupation in the ANOVA.

**The DxAxT interaction (R squared = 0.15)**

This interaction looks at the effect of density on the joint effect of aliquot and timespan; both aliquot and timespan are attributes of the distribution of food over time. The interaction of density, aliquot and timespan is significant only for Average male mass and Prime female mass in the ANOVAs, but it is significant (P<0.05) in the MANOVA (R squared = 0.15). The discriminant function correlation for Prime male mass is large and positive and the correlations for Average male mass and Prime female mass are large and negative. Correlations for the other dependent variables are close to zero. The interaction explains only 1% of the variance for the Prime female mass and the same for the Average male mass, but the AxT and DxT interactions are among the most significant across the ANOVAs and the 3-way interactions have to be addressed before the 2-way interactions. (The AxT interaction was addressed before the DxAxT interaction because it does not involve density as a factor and therefore describes the growth of larvae regardless of competition among them.)

S40 Table shows the means and standard errors for the DxAxT interaction for Prime female mass and age at pupation and the Average female mass at pupation. The Prime female age and the Average female mass are not significant for this interaction, but useful to compare against the Prime female mass. The main effects of density, aliquot and timespan for the Prime female mass are: low density is better than high density, 4 aliquots are better than 2 aliquots, and the 3 day timespan is better than the 6 day timespan. There is a heuristic 3D plot of the Prime female mass in S31 Fig. The treatment combinations are labelled a-h in order of decreasing Prime female mass; treatments e and f are tied at 3.72 mg. This ranking differs from the rankings in other interactions.

1. The largest value of the Prime female mass is in a, the low density, 2 aliquot, 3 day timespan treatment (4.67 mg). The smallest value is in h, the high density, 2 aliquot, 6 day treatment (3.15 mg).
2. The three largest Prime females occur at the low density, a-c, and the two smallest Prime females occur at the high density, g and h, so density appears to be more important than either aliquot or timespan in this interaction, but both aliquot and timespan impact the size of the Prime female.
3. Within each aliquot and timespan treatment, the low density produces larger females than the high density.
4. At the low density, the Prime female mass is largest in a, the 2 aliquot, 3 day timespan treatment (4.67 mg), followed by b, the 4 aliquot, 3 day timespan (4.58 mg), then c, the 4 aliquot, 6 day timespan (4.37 mg). The Prime female mass is smallest in e/f, the 2 aliquot, 6 day timespan treatment (3.72 mg).
5. At the high density, the Prime female mass is largest in d, the 4 aliquot, 3 day timespan treatment (3.87 mg), followed by e/f, the 2 aliquot, 3 day timespan (3.72 mg), then g, the 4 aliquot, 6 day timespan (3.54 mg). The Prime female mass at the high density is smallest in h, the 2 aliquot, 6 day timespan treatment (3.15 mg).
6. The highest value of the Prime female mass at the two densities occur in different aliquot x timespan treatment combinations, although both are 3 day timespan treatments.
7. The lowest values at each density are in the 2 aliquot, 6 day timespan treatment, and they are much lower than the other 3 values for that density.
8. At the low density, the three largest Prime females range from 4.37 mg to 4.67 mg (a-c, 0.30 mg); the difference between the smallest of these and e/f, the 2 aliquot, 6 day treatment is 0.65 mg (14% of 4.67 mg). At the high density, the three largest Prime females range from 3.54 mg to 3.87 mg (d, e/f, g, 0.33 mg); the difference between the smallest of these and h, the 2 aliquot, 6 day treatment is 0.39 mg (10% of 3.87 mg and 8% of 4.67 mg).
9. There is an effect of density on the masses of Prime females, but there is also an effect of density on the relationship between aliquot and timespan. At low density the largest Prime female mass is in a, the 2 aliquot, 3 day timespan treatment, but at high density, the largest Prime female mass is in d, the 4 aliquot, 3 day timespan treatment. This is likely a source of the interaction.
10. The 3 day timespan treatments produce the largest Prime females at both densities. The difference between the Prime female mass in the 3 day timespan treatments across the two densities is 0.09 mg (a and b, low density) and 0.15 mg (d and e/f, high density); this is a significant fraction of the range of the three largest Prime female masses at each density (0.30 mg and 0.33 mg, respectively). The optimum environment for the Prime female is 4 aliquots over 3 days at the high density, but 2 aliquots over 3 days at the low density. In other interactions, the third aliquot increased the available food for the larvae, while the large addition of food on day 3 (2 aliquot treatment) also increased the growth of larvae. Density appears to alter the effects of these treatments on growth (or competition) of Prime females.
11. The third largest Prime female is in the 4 aliquot, 6 day timespan treatment at both low and high densities. The smallest Prime female is in the 2 aliquot, 6 day timespan treatment at both low and high densities.
12. The Prime female age at pupation is not affected by this interaction, but is affected by the three main factors: density, aliquot and timespan. The estimated growth rate combines the Prime female mass and age into a single biologically meaningful number. The estimated growth rates follow the same pattern within each density as the Prime female mass. The larger masses are associated with earlier ages at pupation. The three highest growth rates are at the low density, the next two are at the high density, followed by the slowest growth rate at the low density, then the next two at the high density. The order of the growth rates matches the Prime female mass rather than the age at pupation. The largest Prime females, a and b, have the fastest growth rates and the earliest ages at pupation. The smallest Prime females, g and h, have the slowest growth rates and the latest ages at pupation. The four treatments with intermediate sizes and ages at pupation overlap. The largest Prime female in this group, c, pupates after the second largest, d, perhaps extending the larval period to grow larger (at low density, 4 aliquots, 6 day timespan) while d pupates earlier, but at a smaller size (at high density, 4 aliquots 6 day timespan). The smallest Prime females in this intermediate group, e/f, pupate at the same size, but the high density, 2 aliquot, 3 day timespan grows faster than the low density, 2 aliquot, 6 day timespan.
13. Average female mass is also not affected by this interaction, but is affected by two of the three main factors: aliquot and timespan. The difference between the Prime female mass and the Average female mass is an indication of the relative advantage of the Prime female over the non-Prime females. Because the Prime female mass is significantly affected by this interaction and the Average female is not affected, the non-Prime females must be increasing or decreasing in mass (collectively) so as to offset the changes in the Prime female mass and balance the effect of the Prime female on the Average female mass.
14. The smallest difference between the Prime and Average females is in b, the low density, 4 aliquot, 3 day timespan treatment (0.08 mg, 2% of the largest Prime female mass). This is not the treatment with the largest Prime female, although it is the one with the largest Average female.
15. The largest difference is in h, the high density, 2 aliquot, 6 day timespan treatment (0.40 mg, 9% of the largest Prime female mass). This corresponds to the smallest Prime female, the smallest Average female, the latest pupation, and the slowest growth rate.
16. The others form a tight range (0.18 mg to 0.24 mg, a span of 0.06 mg). Within this tight range, two of the lowest values are in the low density treatments and the two highest values are in the high density treatments.
17. In the prior interactions, the total food and/or food/larva after day 4 corresponds to the order of the Prime female masses at pupation. In this interaction, both food levels (16 mg and 32 mg per test tube) occur in each treatment, so there are two levels listed in S40 Table (e.g. 16 mg, 32 mg total food and 4 mg, 8 mg food/larva). Only the two largest Prime females, a and b, are associated with food/larva values greater than 4 mg; this is the point at which the total food amount influences the size of the Prime females rather than the food/larva value. Both treatments a and b have the same total food amounts, and they are both at low density and the 3 day timespan, so the difference between the two appears to be the number of aliquots. The 2 aliquot treatment results in larger females than the 4 aliquot treatment.
18. The third largest Prime females, c, are associated with the next largest food/larva value.
19. The next three treatments, d-f, are associated with the next largest food/larva value, but the values are all equal (2 mg or 4 mg, depending on the 16 mg or 32 mg food level). Treatment d, (3.87 mg, high density, 4 aliquots and the 3 day timespan) receives more food earlier than treatments e/f (3.72 mg, high density, 2 aliquots, 3 day timespan and low density, 2 aliquots, 6 day timespan). The two e/f treatments either receive all the food on day 0 or half on day 0 and half on day 3 (the 6 day timespan treatment also receives a food input at the end of day 6); there is a difference in the age at pupation and in the size of the Average females across these treatments, but neither of these variables is significantly affected by this interaction. The two e/f treatments appear to be equal for the growth of the Prime females.
20. The last two treatments, g and h, correspond to the lowest food/larva values.

S40 Table. Means (SE) for Prime female mass and age at pupation and Average female mass at pupation for the interaction DxAxT. Estimated growth rates and the differences between the Prime female mass and the Average female mass. Total food after day 4 and food/larva after day 4.

| Density x Aliquot | Timespan | Rank by Prime female mass | Prime female mass at pupation (mg) | Prime female age at pupation (days) | Average female mass at pupation (mg) | Estimated growth rate of Prime female (mg/day) | Prime female mass MINUS Average female mass (mg) | Total food after day 4 (mg) | Food/larva after day 4 (mg) |
| --- | --- | --- | --- | --- | --- | --- | --- | --- | --- |
| 4 larvae, 2 aliquots | 3 days | a | 4.67 (0.32) | 5.56 (0.47) | 4.49 (0.38) | 0.84 (0.34) | 0.18 (0.35) | 16, 32 | 4, 8 |
|  | 6 days | ef | 3.72 (0.63) | 6.65 (0.63) | 3.50 (0.75) | 0.56 (0.69) | 0.22 (0.69) | 8, 16 | 2, 4 |
| 4 larvae, 4 aliquots | 3 days | b | 4.58 (0.25) | 5.80 (0.12) | 4.50 (0.30) | 0.79 (0.15) | 0.08 (0.28) | 16, 32 | 4, 8 |
|  | 6 days | c | 4.37 (0.69) | 6.16 (0.63) | 4.18 (0.69) | 0.71 (0.47) | 0.19 (0.69) | 12, 24 | 3, 6 |
| 8 larvae, 2 aliquots | 3 days | ef | 3.72 (1.07) | 6.35 (1.34) | 3.49 (1.01) | 0.59 (0.71) | 0.23 (1.04) | 16, 32 | 2, 4 |
|  | 6 days | h | 3.15 (0.56) | 8.80 (2.55) | 2.75 (0.36) | 0.36 (0.66) | 0.40 (0.47) | 8, 16 | 1, 2 |
| 8 larvae, 4 aliquots | 3 days | d | 3.87 (1.17) | 6.04 (0.57) | 3.63 (1.12) | 0.64 (0.59) | 0.24 (1.15) | 16, 32 | 2, 4 |
|  | 6 days | g | 3.54 (1.05) | 7.12 (1.40) | 3.35 (1.00) | 0.50 (0.62) | 0.19 (1.03) | 12, 24 | 1.5, 3 |

In the FxD set of interactions (above), competition among females results in large Prime and Average females in the test tubes with the least competition. These Prime females have the fastest estimated growth rate and a small difference between the Prime female mass and the Average female mass, indicating that all females have sufficient food to grow and pupate. In the test tubes with the most competition, the Prime and Average females are the smallest, the growth rate is the slowest, and there is also a small difference between the Prime female mass and the Average female mass. As the larvae grow, the females switch from actively filtering particles to retaining them in their guts; this compresses the size distribution of the females resulting in the small difference between the Prime and Average females. In the test tubes with intermediate levels of competition the Prime and Average females are intermediate in size, the growth rates are intermediate, but the difference between the Prime and Average females is largest at the high density. There are 8 larvae competing for 4 mg food/larva and the Prime female has an advantage over the smaller larvae. The higher total food per test tube allows the Prime female to grow almost as large as the Prime female in the least competition treatment, while the Average female is only a little larger than the Average female in the low density intermediate competition treatment.

If the DxAxT interaction is the result of competition for food, the females at the highest food/larva (4 mg food/larva or greater) should resemble the females in the least competition treatment, the females in the lowest food/larva should resemble the females in the most competition treatment, and the intermediate females should resemble the females in the intermediate competition treatments. Specifically, the intermediate treatments at high density should allow the Prime females to grow almost as large as those in the least competition treatment while the Average females grow only as large as the Average females in the low density intermediate competition treatments. This is clearly not true. The three largest Prime females occur at the low density (a-c); they have the three fastest estimated growth rates and they are associated with the three largest Average female masses, but only one treatment (b) has a small difference between the Prime and Average masses. The next largest female does occur in the high density treatment (d), but the gap between the Prime and Average females is no larger than any of the other intermediate treatments. Finally, the smallest Prime and Average females occur in the high density, 2 aliquot, 6 day timespan treatment, which has the largest difference between the Prime and Average masses. Overall, the masses of the Prime female correspond to the food availability due to the DxAxT treatments, but the size distributions among the females associated with competition (in the FxD set of interactions) do not appear. For the Prime female, the DxAxT interaction describes the growth of females rather than competition among females. As the Prime female benefits from the aliquot and timespan treatments, the non-Prime females correspondingly decrease in size (collectively) so the Average female mass is not affected by this interaction. For the Prime female at the highest food/larva, receiving the large input of food on day 3 (low density, 2 aliquots, 3 day timespan) increases the mass, decreases the age at pupation and increases the estimated growth rate. At the intermediate food/larva (2 mg or 4 mg after day 4), the Prime female grows largest with 4 aliquots and the 3 day timespan (high density). There are two other treatments that have the same food/larva; these achieve the same mass as each other so receiving the food on day 0 and day 3 has the same effect as receiving it all on day 0. At the intermediate food/larva 4 aliquots are better than 2 or 1. The lowest food/larva treatments grow to a smaller mass and grow more slowly according to the amount of food.

S41 Table shows the means and standard errors for the DxAxT interaction for the Prime male mass and age at pupation and the Average male mass. The Prime male mass and age at pupation are not significant in the ANOVAs but are useful to compare against the Average male mass. The main effects of density, aliquot and timespan for the Average male mass are: low density is better than high density, 4 aliquots are better than 2 aliquots, and the 3 day timespan is better than the 6 day timespan. A heuristic 3D plot of the Average male mass for DxAxT is presented in S32 Fig. The treatment combinations are labelled a-h in order of decreasing Prime male mass. This ranking differs from the rankings in other interactions.

1. The largest Average male is in the low density, 4 aliquot, 6 day timespan (2.69 mg). This corresponds to the second largest Prime male, b.
2. The smallest Average male is in the high density, 2 aliquot, 6 day timespan (1.78 mg). This corresponds to the smallest Prime male, h.
3. In this interaction, Average males grow larger at low density than at high density for each of the aliquot and timespan treatments. Density appears to be more important to the size of the Average male than either aliquot or timespan, but both aliquot and timespan change the size of the Average male significantly.
4. The largest Average male at the low density is in the 4 aliquot, 6 day timespan treatment (b, 2.69 mg). The largest Average male at the high density is in the 2 aliquot, 3 day timespan treatment (d, 2.29 mg). The smallest Average male at the low density is in the 2 aliquot, 6 day timespan treatment (f, 2.19 mg). The smallest Average male at the high density is also in the 2 aliquot, 6 day timespan treatment (h, 1.78 mg).
5. The three largest Average males at the low density form a tight range (a-c, 2.60 mg to 2.69 mg, a span of 0.09 mg); the three largest Average males at the high density are also grouped (d, e, and g, 2.08 mg to 2.29 mg, a span of 0.21 mg).
6. The gap between the group of three largest Average males and the smallest Average male at each density is larger than the ranges of the grouped largest Average males (2.19 mg to 2.60 mg, a gap of 0.41 mg for the low density; 1.78 mg to 2.08 mg, a gap of 0.30 for the high density). Part of the cause of this interaction is the disproportionately small size of the Average males in the 2 aliquot, 6 day timespan treatment at both densities.
7. However, there are other asymmetries between the treatments at different densities. At the low density b, the 4 aliquot, 6 day timespan treatment, grows largest, followed by a, the 2 aliquot, 3 day timespan, and c, the 4 aliquot, 3 day timespan; they are similar in size (2.60 mg to 2.69 mg). At the high density d, the 2 aliquot, 3 day timespan treatment, and e, the 4 aliquot, 3 day timespan treatment, are almost the same size, but are 0.20 mg larger than g, the 4 aliquot, 6 day timespan. Each of these asymmetries contributes to the interaction.
8. Neither the Prime male mass nor the Prime male age is affected by this interaction. Nevertheless, the growth rate of the Prime male is an indication of the growing conditions in these test tubes. At the low density, the three treatments with the largest Average males (a-c) also have the largest Prime males and the fastest growth rates. The low density treatment (f) with the smallest Average male mass also has the smallest Prime male mass and the slowest growth rate.
9. At the high density, the two treatments with the largest Average males have the largest Prime males and the fastest growth rates (d and e, both 3 day timespan treatments). Both these high density Prime male masses and their growth rates rank between the 3 largest/fastest low density treatments (a-c) and the smallest/slowest low density treatment (f, 4 larvae, 2 aliquots, 6 day timespan). The larger of the two Average males, d, is associated with the larger Prime male, but a later age at pupation, so the growth rate is slower than the smaller of the two, in treatment e. These two treatments (d and e, high density, 3 day timespan with 2 or 4 aliquots) are very similar to one another across the three dependent variables: Prime male mass and age, Average male mass; and the two computed measures, estimated growth rate and the difference between Prime and Average male masses (see below). The aliquot treatment has little effect on the growth of males at high density and the 3 day timespan.
10. The two high density treatments with the smallest Average male masses (g and h) also have the smallest Prime male masses and the slowest growth rates (at high density and across the interaction).
11. The difference between the Prime and Average male masses is an indication of the size distribution of the males and also of the relative advantage of the Prime male over the non-Prime males. Because this interaction affects only the Average male mass and not the Prime male mass, the effect is on the non-Prime males.
12. There is no simple pattern organizing the size differences between the Prime and Average male masses. The two largest differences are in treatments d and e (high density, 3 day timespan), but the Average male mass is larger than the Prime male mass in h, with the smallest Prime and Average males. In other interactions, this is because there is a final input of food on day 6 and the non-Prime males grow larger after the Prime male pupates. This advantage to the non-Prime males could also explain the smaller differences between the Prime and Average male masses in b and g, but the difference is larger in f, the 6 day timespan, than in a, the 3 day timespan (both are low density, 2 aliquots treatments).
13. The total food after day 4 and the food/larva after day 4, which explain the size order of females in this interaction (S40 Table), do not explain the size order of the Average male masses. The largest Average male occurs in b, the low density, 4 aliquot, 6 day timespan treatment, where the food/larva after day 4 is 3 mg or 6 mg (depending on the food level, 16 mg or 32 mg). The Prime male pupates on day 5, so the non-Prime males could be growing larger on the additional food after day 6 and the release from competition with the Prime male. They grow almost as large as the Prime male in this treatment (2.70 mg for the Prime male and 2.69 mg for the Average male).
14. The two treatments with the most total food and food/larva after day 4 are a and c, both low density, 3 day timespan treatments. There is not much difference between the Prime male masses, the Average male masses, or the growth rates across these two treatments. Both Prime males delay pupation slightly (5.06 days and 5.14 days). The larger of the two Prime males pupates earlier and grows faster than the second. The larger Prime male is associated with the larger Average male and the smaller difference between them, so 2 aliquots is better for males than 4 aliquots at the low density and high total food (or food/larva). This is another source of the interaction for the Average male mass.
15. The three middle treatments, d-f, all have the same total food and food/larva after day 4 (2 mg or 4 mg, depending on the food levels 16 mg or 32 mg). The Average male masses are more similar than the Prime male masses; the range for the Average males is 2.19 mg to 2.29 mg (0.10 mg) and the range for the Prime males is 2.27 mg to 2.44 mg (0.17 mg). The two 3 day timespan treatments (d and e) are larger and grow faster and the difference between the Prime and Average male masses is larger than in treatment f, the 6 day timespan. The 3 day timespan treatments (d and e) receive all the food by the end of day 3; they are both high density treatments and 2 aliquots results in larger males than 4 aliquots. The 6 day timespan treatment (f) receives half the amount of food on day 0 that d and e receive over 3 days; the Prime male does not grow as large as the Prime males in d and e, but grows almost as fast, pupates on day 5, and the non-Prime males grow larger on the day 6 food input (reducing the difference between the Prime and Average males in f, and also the difference between the Average males across d, e and f).
16. Treatments g and h have the lowest food/larva and the smallest masses and growth rates across the interaction. Both receive food inputs on day 6 and the non-Prime males in both treatments grow after the pupation of the Prime male (5.25 days and 5.45 days, the latest of all the Prime males in this interaction). The difference between the Prime and Average males in g is 0.04 mg, so the non-Prime males grow larger on the additional food, but they do not catch up to treatments with more food earlier in the larval period. The non-Prime males in h are larger than the Prime males (0.05 mg larger), but they are still the smallest across the interaction. The additional food on day 6 and the release from competition with the Prime male allows the non-Prime males to grow larger, but they do not catch up with the treatments that received more food earlier in the larval period.

S41 Table. Means (SE) for Prime male mass and age at pupation and Average male mass at pupation for the interaction DxAxT. Estimated growth rates and the differences between the Prime male mass and the Average male mass.

| Density x Aliquot | Timespan | Rank by Prime male mass | Prime male mass at pupation (mg) | Prime male age at pupation (days) | Average male mass at pupation (mg) | Estimated growth rate of Prime male (mg/day) | Prime male mass MINUS Average male mass (mg) |
| --- | --- | --- | --- | --- | --- | --- | --- |
| 4 larvae, 2 aliquots | 3 days | a | 2.71 (0.07) | 5.06 (0.08) | 2.66 (0.06) | 0.54 (0.04) | 0.05 (0.07) |
|  | 6 days | f | 2.27 (0.39) | 5.00 (0.00) | 2.19 (0.44) | 0.45 (0.13) | 0.08 (0.42) |
| 4 larvae, 4 aliquots | 3 days | c | 2.69 (0.11) | 5.14 (0.01) | 2.60 (0.01) | 0.52 (0.04) | 0.09 (0.08) |
|  | 6 days | b | 2.70 (0.26) | 5.00 (0.00) | 2.69 (0.26) | 0.54 (0.10) | 0.01 (0.26) |
| 8 larvae, 2 aliquots | 3 days | d | 2.44 (0.50) | 5.10 (0.14) | 2.29 (0.43) | 0.48 (0.18) | 0.15 (0.47) |
|  | 6 days | h | 1.73 (0.63) | 5.25 (0.35) | 1.78 (0.36) | 0.33 (0.17) | -0.05 (0.51) |
| 8 larvae, 4 aliquots | 3 days | e | 2.41 (0.37) | 5.00 (0.00) | 2.28 (0.37) | 0.48 (0.13) | 0.13 (0.37) |
|  | 6 days | g | 2.12 (0.55) | 5.45 (0.63) | 2.08 (0.43) | 0.39 (0.23) | 0.04 (0.49) |

Males compete with other males and with females for food; females have the advantage due to their larger size, but males appear to compete among themselves more intensely and for an aspect of the food resource that is different from the females. This interaction affects the growth of Prime females, and collectively diminishes the non-Prime females where it increases the size of the Prime females, but does not appear to affect competition among females. It is possible that this interaction affects the competition among the non-Prime males independently of the effect on Prime females.

In the FxDxT and FxD interactions, Prime and Average males are largest in the least competition treatments, and smallest in the most competition treatments. The high density intermediate competition treatments (4 mg food/larva) produce larger Prime males and smaller Average males than the low density intermediate competition treatments (also 4 mg food/larva). The largest difference between Prime and Average males is in the high density intermediate competition treatments. Non-Prime males grow larger than the Prime males in the most competition treatment. In the FxD interaction, this increment is 0.01 mg larger than the Prime males. In the FxDxT interaction, the increment is 0.14 mg larger. The FxD increment represents only the release of competition after the Prime male pupates, while the FxDxT increment represents both the release of competition and the additional food at the end of day 6 in the 6 day timespan treatment. The effect of the release from competition with the Prime male is much smaller than the effect of the additional food after day 6.

In the FxDxT interaction, the total food after day 4 explains the size order of Prime males when the food/larva is 4 mg or above. The food/larva after day 4 explains the size order of Prime males below 4 mg food/larva. The outcome of competition for the Prime males is directly related to the availability of food after day 4. This is not true of the Average male mass. The outcome of competition among males in the FxDxT interaction resembles that of the FxD interaction for the 3 day timespan treatment. Prime males are smaller in the 6 day timespan for each treatment. The non-Prime males grow larger in the 6 day timespan treatment than the 3 day timespan treatment in the least competition test tubes. The non-Prime males grow larger than the Prime males in the most competition test tubes. Both are due to the release from competition and the additional food after day 6. In the intermediate competition treatments (4 mg food/larva), the effect of the 6 day timespan is to reduce the size of both Prime and Average males, and to reduce the difference between them at the high density, but to increase that difference at the low density.

The Average male mass in the DxAxT interaction is similar to the Average male mass in the FxDxT interaction in several ways (S23 Table FxDxT versus S41 Table DxAxT for Average Male Mass). First, the largest Average male occurs in the 6 day timespan treatment with the most food; these are the least competition treatment in FxDxT and the low density, 4 aliquot treatment in DxAxT. These are not the treatments with the most food after day 4 in either interaction (S22 Table and S40 Table). The Prime males in these treatments are not the largest in the respective interactions, but the differences between the Prime male mass and the Average male mass are the smallest (0.02 mg for FxDxT, 0.01 mg for DxAxT, but see below). The large size of the Average male mass is likely due to the growth of non-Prime males on the final input of food after day 6. Second, the next largest Average males occur in the 3 day timespan treatments with the most food/larva; these are also the treatments with the largest Prime males, but not necessarily in the same order as the Average males. There are 4 treatments in FxDxT where all test tubes have 4 mg food/larva or higher, and 3 treatments in DxAxT where all test tubes have 4 mg food/larva or higher. There is a gap between the 3 or 4 treatments above and the remainder in each interaction (0.36 mg for FxDxT and 0.31 mg for DxAxT). Below this gap the Average male mass and the Prime male mass are in the same order, corresponding to the food/larva after day 4. In both interactions, the smallest Average males grow larger than the Prime males in their respective treatments; both treatments are 6 day timespans, so these non-Prime males grow larger on the release from competition and the additional food on day 6. In each interaction there is one treatment at an intermediate food level that results in a large Prime male mass, a smaller Average male mass, and a large difference between them. In the FxDxT interaction this treatment is the high food, high density, 3 day timespan (Prime male, 2.73 mg; Average male 2.57 mg; difference 0.16 mg). In the DxAxT interaction the treatment is the high density, 2 aliquot, 3 day timespan (Prime male, 2.44 mg; Average male, 2.29 mg; difference 0.15 mg). The relationships between Prime male mass, Average male mass, the difference between the two masses, and the food/larva after day 4 suggest that the non-Prime males are competing in the DxAxT interaction in the same way that they are competing in the FxDxT interaction; aliquot interacts with density and timespan for the non-Prime males similarly to the way that food level does in the FxDxT interaction.

In the FxDxT interaction for Average male mass, the 6 day timespan results in a smaller Average male than the 3 day timespan (with the exception of the largest Average male). Food and density are more important to the order (and to food/larva) than timespan. In the DxAxT interaction for Average males, the 6 day timespan results in a smaller Average male than the 3 day timespan (again with the exception of the largest Average male). The 4 treatments with the 3 day timespan follow the largest Average male (6 day timespan). The 3 smallest Average males are in the other test tubes with the 6 day timespan. Timespan is as important as density and both are more important that aliquot to the size order of the Average males. At both densities the 2 aliquot treatment produces larger Average males with the 3 day timespan, but 4 aliquots are better than 2 aliquots with the 6 day timespan. The simple explanation for the different effects of the 2 or 4 aliquot treatments at high and low food availabilities is that the 2 aliquot, 3 day timespan combination puts a large amount of food in the test tube on day 3 when the larvae are growing fastest and this increases the size of the male larvae. The 4 aliquot, 6 day combination provides more food, earlier than the 2 aliquot, 6 day combination. Both add more food on day 6, which benefits the non-Prime males, but the third input on day 4 has more benefit than a larger input on day 6.

The order of the size of the Average male does not correspond to either the total food after day 4 or the food/larva after day 4. The largest Average males are in the test tubes where the Prime male has the highest estimated growth rate. In this interaction, the Prime male estimated growth rate predicts the Average male mass better than the total food or food/larva or any combination of the independent factors. The Prime male mass and age at pupation are not affected by this interaction, but the growth of the Prime male predicts the mass of the Average male. Growth rate is an indication of the competitive environment; high growth rates occur in the test tubes with the least competition (in the FxD interactions). This suggests that the Prime males are not affected by competition governed by aliquot and timespan, but that the non-Prime males are affected.

**Males versus females, competition or differences in growth**

1. The Prime female mass and the Average male mass are both affected by the interaction DxAxT. Density affects both variables more than either aliquot or timespan; for each combination of aliquot and timespan, the value of the variable at low density is larger than that at high density. There is no similar simple relationship for either aliquot or timespan.
2. For both Prime female mass and Average male mass, the relationship between aliquot and timespan at high density is different from that at low density.
3. For both, at high density, the two 3 day timespan treatments are large and similar, while the two 6 day treatments are smaller and widely separated. The high density, 2 aliquot, 6 day treatment results in much smaller Prime females and Average males.
4. The larger Prime females and Average males differ from each other at the high density: Prime females are larger in the high density, 4 aliquot, 3 day timespan treatment and Average males are larger in the high density, 2 aliquot, 3 day timespan treatment.
5. However, at high density the asymmetries look very similar to those described for Prime female mass and Average male mass in the AxT interaction (above), so they probably aren’t the source of the DxAxT interactions for these two variables.
6. Comparing the Prime female mass to the Average male mass is complicated. It is better to compare the Prime female mass to the Prime male mass, and to compare the Average female mass to the Average male mass. The Prime male mass is not affected by this interaction, but the Average female mass includes the Prime female mass, and the inferred effect on the non-Prime females. S42 Table shows the Prime female mass and Average male mass alongside the: Prime female mass MINUS Average female mass, Prime female mass MINUS Prime male mass, Prime male mass MINUS Average male mass, Average female mass MINUS Average male mass.
7. At low densities the Prime female is largest in the 2 aliquot, 3 day timespan treatment, with the Prime female in the 4 aliquot, 3 day timespan treatment pupating at a somewhat smaller mass. The Prime female in the low density, 4 aliquot, 6 day treatment is smaller still and the one in the low density, 2 aliquot, 6 day timespan treatment is much smaller. This is different from the high density outcome, and appears to be the source of the DxAxT interaction for the Prime female mass.
8. At the low density, in the 3 day timespan treatments, the Prime female benefits from the large input of food on day 3 (2 aliquot treatment) compared to the 3 equal inputs on days 1-3 (4 aliquot treatment). The Average female at low density grows larger on the 4 aliquot treatment, so the large input of food on day 3 benefits only the Prime female.
9. The Prime females are expected to be the best competitor in the test tubes. This suggests that the Prime female experiences an abundance of food and grows larger due to the food input in the middle of the larval period. Because the Average female mass is not affected by this interaction, the non-Prime females are collectively smaller; they are reduced in size in the treatments where the Prime females grow larger. The Prime female grows larger, the non-Prime females also grow large due to the food/larva, but the difference between the Prime and Average females is 0.18 mg (low density, 2 aliquots, 3 day timespan). Comparing this to the 4 aliquot treatment (at low density and 3 day timespan), the Average female mass is about the same, but the Prime female mass is smaller and the difference between the two is the smallest (0.08 mg) across the interaction. The non-Prime females do relatively better against the Prime female with 4 aliquots rather than 2 aliquots.
10. The source of the interaction is due to the asymmetry at the low density. This suggests that the interaction effect for females is due to the abundance of food rather than to competition; competition would result in larger differences at the higher density, not the lower one. In fact, the largest difference between the Prime female mass and the Average female mass occurs in the high density, 2 aliquot, 6 day timespan treatment, but this is the same pattern that is observed in the AxT interaction, which describes growth not competition.
11. The Prime male mass is not significantly affected by this interaction, but is affected by the AxT interaction and by density. Comparing the Prime female mass to the Prime male mass shows the relative advantage of the Prime female over the Prime male. The largest difference between the two Prime masses, indicating the largest relative advantage of the Prime female over the Prime male, is in the test tubes with the low density, 2 aliquot, 3 day timespan treatment (1.96 mg). This corresponds to the largest Prime female and the largest Prime male.
12. The difference between the Prime female and the Prime male masses decreases as the Prime female mass decreases. In three of these treatment the Prime males are almost the same size (2.69 mg to 2.71 mg) while the females range from 2.60 mg to 2.69 mg (a-c). Some of this pattern is likely due to the Prime males having sufficient food to grow to their maximum size (determined by food and other factors) and then pupating, while females grow as large as possible on the available food. The differences between the Prime female mass and the Prime male mass in the remaining treatments are smaller, but consistent (1.42 mg to 1.46 mg) suggesting that at lower availabilities of food females have a consistent advantage over males. There is an exception to this in the high density, 2 aliquot, 3 day timespan treatment; the fourth largest Prime male occurs with the fifth largest Prime female and the difference between them is only 1.28 mg. Both the Prime female mass and the Prime male mass at the high density resemble the pattern in the AxT interaction, but there is no correspondingly small difference between the Prime individuals in the AxT interaction. Nevertheless, there is no indication that competition between the Prime individuals is the cause of the DxAxT interaction. Competition should show up as a larger difference in size at an intermediate food level rather than a smaller one. This also suggests that interference competition is not involved; interference competition should result in a larger difference between the two masses as the sizes of the masses decrease.
13. The Prime female masses across the high density treatments in the DxAxT interaction resemble the Prime female masses in the AxT interaction. The Prime male masses across the high density treatments in the DxAxT interaction also resemble the Prime male masses in the AxT interaction. The AxT interaction does not involve density, so there is no effect of competition on the interaction. This suggests that there is no effect of competition between Prime males and Prime females at high density in the DxAxT interaction.
14. The Average male mass does not resemble the Average male mass in the AxT interaction at the low density in the DxAxT interaction; the Average male mass at the high density does resemble the Average male mass in the AxT interaction. The DxAxT interaction must be due to the Average male mass at the low density; this is not where competitive effects would show up (see Prime female above).
15. At low densities, the Average male mass is largest in the 4 aliquot, 6 day timespan treatment. The two 3 day timespan treatments are a little smaller and the 2 aliquot, 6 day timespan treatment is smallest.
16. This doesn’t correspond to the order of the Average female masses (not significant in this interaction), but the difference between the Average female mass and the Average male mass conforms to the size of the Average female. As the Average female decreases in size across the treatments in this interaction, so does the difference between the Average female and the Average male. There is a similar asymmetry in the high density, 2 aliquot, 3 day timespan treatment for the difference between the Average female and the Average male masses as described above for the Prime female and Prime male masses. This suggests that males and females are growing in response to different aspects of the food supply, since both Prime males and females and Average males and females show the same pattern: the fourth largest male occurs in the test tubes with the fifth or sixth largest female, resulting in the smallest difference between the Prime masses and also between the Average masses. (The smallest Prime and Average individuals of both sexes occur in the high density, 2 aliquot, 6 day timespan treatment, and the non-Prime males grow larger than the Prime males resulting in an even lower difference between the Average female mass and the Average male mass, but this appears to be different from the effect in the high density, 2 aliquot, 3 day timespan treatment.)
17. The two largest Average female masses (low density, 3 day timespan) are much larger than the respective Average males (1.83 mg and 1.90 mg larger, for 2 aliquots and 4 aliquots, respectively). The difference between the Average female and the Average male in the 4 aliquot, 6 day timespan (the largest Average male) is only 1.49 mg. The females in the low density, 3 day timespan test tubes appear to have a significant advantage over the males. These are the second and third largest Average males in the interaction, yet the Average female masses are more than two-thirds again as big (69% and 73% of the corresponding Average male masses). In the low density, 3 day timespan treatments food is abundant, and the females grow much larger than the males.
18. The 2 aliquot treatment is better for males at the 3 day timespan, but the 4 aliquot, 6 day timespan results in the largest Average males, probably due to the even inputs of food combined with the final input after the Prime males have pupated. The anomaly that causes the DxAxT interaction for the Average male mass appears to be the high value at low density, 4 aliquots and the 6 day timespan. This is an interaction of density with food distribution (AxT) that is independent of food level or competition (FxD).
19. The Prime males pupate on day 5 in this treatment combination, and most of the Prime females pupate on day 6, before the addition of the 4th aliquot of food. This allows the non-Prime males to grow almost as large as the Prime male (2.70 mg for the Prime male and 2.69 mg for the Average male in this treatment combination). With only 4 larvae in the low density test tubes, and with the Prime male and Prime female pupating before the addition of the food on day 6, there are only two larvae remaining to consume one-quarter of the total food.
20. The same situation should be true for the low density, 2 aliquot, 6 day timespan treatment combination, although more of the Prime females pupate after day 6. In this case, half the food is input on day 0 and half on day 6. Both males and females in this treatment are smaller than in the other low density treatments. The remaining larvae receive an input equal to the initial input, but this still does not allow them to grow as large as Average males in the other low density treatments. Later inputs of food do not make up for early food scarcity. The difference between the Average female mass and the Average male mass in this treatment is the smallest (1.31 mg) of the low density treatments. At low density, the 6 day timespan results in larger Average males relative to the size of the Average females. The non-Prime males appear to grow relatively larger on the final aliquot of food compared to the females. This suggests a biological difference in the males and females; perhaps the females are limited in their maximum size by a physical or physiological characteristic that is determined at the last molt and the males are able to respond to late additions of food by growing larger (but not as large as the females).
21. Unlike the Average males at low density, the males in both high density, 3 day timespan treatments are largest, followed by the males in the 4 aliquot, 6 day timespan; the males in the 2 aliquot, 6 day timespan are smallest. This resembles the asymmetry in the AxT interaction. At high densities, the Average male mass is largest in the 2 aliquot, 3 day timespan treatment; the 4 aliquot, 3 day timespan is a little smaller. The 2 aliquot, 6 day timespan is much smaller than the other three and the 4 aliquot, 6 day timespan treatment is intermediate, but closer to the 3 day timespan treatments than to the 2 aliquot, 6 day timespan.
22. Across the high density treatments, the difference between the Average female mass and the Average male mass in the 2 aliquot, 3 day timespan is smallest relative to the size of the Average male (1.20 mg, 52% of the Average male mass). This is not the smallest difference, but it corresponds to the smallest difference between the Prime females and the Prime males. (see above)
23. The Average female in the 4 aliquot, 3 day timespan is larger than that in the 2 aliquot, 3 day timespan, but the Average male is larger in the 2 aliquot, 3 day timespan. This asymmetry also corresponds to that of the Prime individuals.
24. Across the high density, for the 4 aliquot, 3 day timespan and both 6 day timespan treatments, the difference between the Average female mass and the Average male mass decreases as the Average female mass decreases, also indicating the absence of interference competition.
25. Another asymmetry is that the high density, 2 aliquot, 6 day timespan Average male is larger than the Prime male in this treatment. The Average female mass, the Average male mass, and the difference between the Average female and the Average male in this treatment are the smallest across the high density treatments, and across the interaction. This is the most food stressed treatment, resulting in the smallest males and females, but the relative advantage of the females over the males is the smallest because the non-Prime males manage to grow larger on the food input after the Prime males have pupated. The non-Prime females do not respond to the food input in the same way (see #423).
26. In the high density test tubes for the DxAxT interaction, the Prime males all pupate before day 6 while the Prime females in the 6 day timespan treatments pupate well after day 6. There are possibly seven larvae in each test tube when the final aliquot is added. The non-Prime males in the high density, 2 aliquot, 6 day timespan treatment combination grow larger than the Prime males on the additional food (the Average male mass is 0.05 mg larger than the Prime male mass). They do not grow as large as the Average males in the high density, 4 aliquot, 6 day treatment; later inputs of food do not make up for early food shortages.
27. Females may dominate the competition overall by changing their feeding behavior in response to food availability, but males appear to compete more intensely with each other than with females.
28. In the FxD interaction the competitive release on the non-Prime males accounted for a 0.01 mg difference in the size of the Average male relative to the Prime male. In the FxDxT interaction, the difference included both the competitive release and the final addition of food on day 6 and accounted for 0.14 mg. In the DxAxT interaction the corresponding treatment resulted in a difference of 0.05 mg. It is unclear whether this represents competitive release and the final addition of food or only the food. However, there is no comparable situation, where the non-Prime males grow larger than the Prime male, in the AxT interaction. There is a similar difference (0.04 mg in the low food, 6 day timespan treatment) in the FxT interaction, which should only describe growth (no competitive release).

S42 Table. Means (SE) for Prime female mass and Average male mass for the interaction DxAxT. Prime female mass MINUS Average female mass, Prime female mass MINUS Prime male mass, Prime male mass MINUS Average male mass, Average female mass MINUS Average male mass.

| Density x Aliquot | Timespan | Prime female mass at pupation (mg) | Average male mass at pupation (mg) | Prime female mass MINUS Average female mass (mg) | Prime female mass MINUS Prime male mass (mg) | Prime male mass MINUS Average male mass (mg) | Average female mass MINUS Average male mass (mg) |
| --- | --- | --- | --- | --- | --- | --- | --- |
| 4 larvae, 2 aliquots | 3 days | 4.67 (0.32) | 2.66 (0.06) | 0.18 (0.35) | 1.96 (0.23) | 0.05 (0.07) | 1.83 (0.27) |
|  | 6 days | 3.72 (0.63) | 2.19 (0.44) | 0.22 (0.69) | 1.45 (0.52) | 0.08 (0.42) | 1.31 (0.61) |
| 4 larvae, 4 aliquots | 3 days | 4.58 (0.25) | 2.60 (0.01) | 0.08 (0.28) | 1.89 (0.19) | 0.09 (0.08) | 1.90 (0.21) |
|  | 6 days | 4.37 (0.69) | 2.69 (0.26) | 0.19 (0.69) | 1.67 (0.52) | 0.01 (0.26) | 1.49 (0.52) |
| 8 larvae, 2 aliquots | 3 days | 3.72 (1.07) | 2.29 (0.43) | 0.23 (1.04) | 1.28 (0.84) | 0.15 (0.47) | 1.20 (0.78) |
|  | 6 days | 3.15 (0.56) | 1.78 (0.36) | 0.40 (0.47) | 1.42 (0.60) | -0.05 (0.51) | 0.97 (0.36) |
| 8 larvae, 4 aliquots | 3 days | 3.87 (1.17) | 2.28 (0.37) | 0.24 (1.15) | 1.46 (0.87) | 0.13 (0.37) | 1.35 (0.83) |
|  | 6 days | 3.54 (1.05) | 2.08 (0.43) | 0.19 (1.03) | 1.42 (0.84) | 0.04 (0.49) | 1.27 (0.77) |

**DxAxT summary**

This interaction affects the Prime female mass and the Average male mass. Because the Prime female mass is included in the Average female mass, the non-Prime females must be affected collectively in an equal and opposite manner than the Prime female. The net effect on the Average female mass is insignificant. The effect on the Average male mass without the Prime male mass indicates that only the non-Prime males are affected by this interaction. For both the Prime female mass and the Average male mass, the high density treatments resemble the results of the AxT interaction, so they are probably not the cause of the 3-way interaction. The Prime female mass and the Average male mass differ from the AxT interaction at the low density and they also differ from each other.

This interaction involves density and two attributes of the food supply describing the temporal availability of food. It is possible that this interaction describes another type of competition in addition to the food level x density competition described in the first set of interactions (FxDxT, FxDxAxT, FxDxA, FxD). Within each density food should be most abundant in the 4 aliquot, 3 day timespan and most restricted in the 2 aliquot, 6 day timespan. The largest Prime females and Average males should be in the low density, 4 aliquot, 3 day timespan treatment and the smallest should be in the high density, 2 aliquot, 6 day timespan treatment. The smallest Prime females and Average males are in the high density, 2 aliquot, 6 day timespan treatment, but neither the Prime female nor the Average male are largest in the low density, 4 aliquot, 3 day timespan treatment. The Prime female and the Average male deviate from the predicted optima in different ways. At the low density, the size of the Prime female follows the food/larva after day 4 and the total food after day 4 (they are in the same order). At the low density, the size of the Average male follows the estimated growth rate of the Prime male, an indicator of the competitive stress in the test tubes.

The Prime female is largest at the low density, 2 aliquot, 3 day timespan. This treatment also shows the fastest estimated growth rate, the earliest age at pupation, and the largest difference between the Prime female and the Prime male. The Prime female in the low density, 4 aliquot, 3 day timespan is second largest, has the second fastest estimated growth rate, the second earliest age at pupation and the second largest difference between the Prime female and the Prime male. The Prime female appears to benefit from the large input of food on day 3 compared to the smaller inputs on days 1-3. The relative abundance of food particles due to the day 3 input cause the females to actively filter; they grow larger and the size distribution also increases in size (the non-Prime females are reduced in size compared to the Prime female). This accounts for the different outcomes between the two highest food/larva levels for Prime females. The timing of the larger food input (2 aliquot) on day 3 favors the growth of the Prime female over the other larvae. This is similar to the effect of large food inputs on day 3 for Average females (FxDxAxT interaction) and Prime females (FxAxT).

The Average male is largest at the low density, 4 aliquot, 6 day timespan. This treatment is associated with the second largest Prime male mass, the highest estimated growth rate for the Prime male, the earliest age at pupation for the Prime male, the second smallest difference between the Prime male and Average male, the third largest difference between the Prime female and Prime male, and the third largest difference between the Average female and the Average male. None of the other parameters that describe the growth and potential competition of these larvae reach their highest value except for the Average male mass and the Prime male age at pupation and estimated growth rate. The non-Prime males benefit from the pupation of the Prime male and grow larger on the food added on day 6. They grow almost as large as the Prime male in this treatment. The low density, 4 aliquot, 6 day timespan treatment is associated with the fastest Prime male growth rate suggesting that the competitive environment for males in these test tubes is less stressful even though the total food and food/larva levels are not the highest after day 4. Males are competing differently than females for the same yeast food resource. The next two largest Average males are at the low density, 2 aliquot, 3 day timespan, and the low density, 4 aliquot, 3 day timespan. These correspond to the two largest Prime females and the two highest total food and food/larva levels. The Prime male estimated growth rates for these treatments are only a little smaller than for the low density, 4 aliquot, 6 day timespan. The non-Prime males benefit from the lower competitive stress in these test tubes, and the non-Prime males in the low density, 4 aliquot, 6 day timespan treatment also benefit from the added food on day 6. This is the only treatment in which the non-Prime males grow larger on the 6 day timespan than on the 3 day timespan (at the same density and aliquot).

The non-Prime males in the low density, 4 aliquot, 6 day timespan treatment do considerably better than the non-Prime males in the low density, 2 aliquot, 6 day timespan treatment because the lower food availability in the 2 aliquot test tubes causes the females to switch to retention and the males are reduced in size relative to the females (0.43 mg for the Prime males and 0.50 mg for the Average males, between the 4 aliquot and 2 aliquot treatments at low density and the 6 day timespan). The final aliquot of food is larger on day 6 for the 2 aliquot treatment, but the smaller larvae are unable to make up the difference on the late input of food. Late inputs are less valuable to the growth of the larvae than earlier inputs of equal size.

The two low density treatments with the 3 day timespan result in Average male masses that are almost as large as the Average male in the low density, 4 aliquot, 6 day timespan, but competition with females and competition with the Prime male affect the size of the non-Prime males and there is no addition of food after the Prime male pupates to boost the size of the non-Prime males. The non-Prime males in the high density, 2 aliquot, 6 day timespan treatment also grow larger on the final aliquot of food on day 6; they grow larger than the Prime male in that treatment. It is likely that the non-Prime males in the other 6 day treatments benefit from the final aliquot as well, but it isn’t obvious in the numerical relationships.

Females appear to grow in response to the food/larva and total food at low density, but the amount and timing of the food input on day 3 increases the size of the Prime female (and implicitly reduces the size of the non-Prime females) relative to other equal and similar food levels. The masses of the Prime females in this interaction can be explained by exponential growth processes without invoking competition.

Non-Prime males appear to grow in response to the competition with the Prime males, but benefit from the final input on day 6. At the lowest level of competition, the non-Prime males grow largest, but at the highest level of competition, the non-Prime males grow relatively larger; they are larger than the Prime males in the same test tubes.

**The DxA interaction (not significant in the MANOVA)**

S43 Table shows the means and standard errors for the Prime female mass and age at pupation and the Average female mass at pupation. Prime female age is the only variable significantly affected by this interaction in the ANOVA (r squared = 0.01); the two mass variables are included to be able to relate the age at pupation to the growth rates and the differences between Prime and Average female masses (both potential indicators of competition). This is the smallest of the 4 significant interaction contrasts for Prime female age. The main effects predict that low density is better (earlier) than high density and 4 aliquots are better than 2 aliquots. A heuristic 3D plot of the Prime female age at pupation for the DxA interaction is presented in S33 Fig.

1. Prime females pupated earliest at the low density with 4 aliquots of food (5.98 days), and latest at the high density with 2 aliquots (7.58 days). The effects of the two treatments appear to match the main effects.
2. The impact of aliquot on age at pupation is small at low density (0.12 days), and large at high density (1.0 days). The 2 aliquot treatment adds half the food on day 0 and the second half on day 3 or day 6. The 4 aliquot treatment adds a quarter of the food on day 0, a second quarter on day 1 or day 2, the third quarter on day 2 or day 4, and the final quarter on day 3 or day 6. Most of the Prime females in the low density treatment pupate before the final food aliquot on day 6 (although timespan is not part of this interaction). At the high density, half the Prime females in the 4 aliquot treatment delay pupation until after day 6, and all of the Prime females in the 2 aliquot treatment delay pupation until after day 6. Regardless of the food level (total food per test tube or food/larva), receiving half the food on day 0 and not receiving more food until day 3 or day 6 results in a delay in pupation for the Prime female that is much greater at high density.
3. The projected means (based on the main effects) range from 6.16 days to 6.96 days (a span of 0.80 days), while the actual mean ages at pupation range from 5.98 days to 7.58 days (a span of 1.60 days, twice that of the projection). The actual mean ages at pupation are earlier than the projected means except for the high density, 2 aliquot treatment, which is 0.62 days later. The low density, 2 aliquot treatment pupates 0.34 days earlier than projected, while two 4 aliquot treatments pupate only 0.10 days and 0.18 days earlier. The low density, 2 aliquot treatment pupates disproportionately early and the high density, 2 aliquot treatment pupates disproportionately late.
4. The Prime female mass at pupation is not affected by this interaction, and the Prime female masses are in the same order as the ages; the largest masses correspond to the earliest ages at pupation. The estimated growth rates do not offer insight into this interaction.
5. The asymmetry that causes this interaction for the Prime female age appears to be the large difference due to density in the 2 aliquot treatment compared to the much smaller effect in the 4 aliquot treatment. High density results in later pupation than low density; the ages at pupation are later at high density regardless of aliquot treatment. The addition of food in 2 aliquots results in delayed pupation compared to 4 aliquots at both densities, but the delay is much greater at the high density. However, the Prime females in the low density, 2 aliquot treatment pupate earlier than expected by a larger difference than either of the 4 aliquot treatments, and the Prime females in the high density, 2 aliquot treatment pupate later than expected.
6. The Prime females in the 4 aliquot treatments have the same total food in both densities after day 4 (12 mg, 16 mg, 24 mg or 32 mg depending on food level and timespan). The food/larva in the high density is half that of the low density. The Prime females in the 2 aliquot treatments have slightly less total food after day 4 (8 mg, 16 mg, 16 mg, or 32 mg again depending on food level and timespan). The food/larva in the high density is also half that of the low density. In the FxDxT interaction the Prime female age at pupation is influenced more by the total food than by the food/larva (after day 4). In the FxD interaction the Prime female age increases as competitive environment increases, but the age increases disproportionately when the food/larva is less that 4 mg. Neither total food after day 4, nor food/larva after day 4 explains the asymmetry in age at pupation that causes this interaction. Food/larva on day 0 (the initial input of food) appears to match the deviations from the projected values of Prime female age at pupation. The Prime females in the test tubes with low density and 2 aliquots have twice as much food on day 0 as the other treatments. This suggests that a large amount of food on day 0 may accelerate the growth of females while not affecting the mass at pupation. The Prime females in the test tubes with high density and 2 aliquots probably delay pupation because they have less food/larva after day 4 than the other 3 treatments, similarly to the FxD interaction.
7. In the FxD set of interactions, which describe competition among females, the difference between the Prime female mass and the Average females mass describes the size distribution among females and the relative advantage of the Prime female over the non-Prime females. Neither of these two mass variables is significantly affected by this interaction. If this interaction reflected competition as in the FxD set of interactions, the largest Prime female should be associated with the smallest difference between the Prime and Average female masses, the smallest Prime female should be associated with a similar small difference, and the high density, 4 aliquot treatment should be associated with the largest difference. This is not true. The largest difference between the Prime and Average females is associated with the smallest Prime female suggesting that this interaction is due to exponential growth processes rather than competition.

S43 Table. Means (SE) for Prime female mass and age at pupation and Average female mass at pupation for the interaction DxA. Estimated growth rates, differences between Prime and Average female masses, expected mean values for Prime female age, and food levels after day 4 and on day 0.

| Density x Aliquot | Prime female mass at pupation (mg) | Prime female age at pupation (days) | Average female mass at pupation (mg) | Estimated growth rate (mg/day) | Prime female mass MINUS Average female mass (mg) | Expected mean values for Prime female age (days) | Total food after day 4 (mg) | Food/larva after day 4 (mg) | Food/larva on day 0 (mg) |
| --- | --- | --- | --- | --- | --- | --- | --- | --- | --- |
| 4 larvae, 2 aliquots | 4.19 (0.68) | 6.10 (1.16) | 4.00 (0.75) | 0.69 (0.46) | 0.19 (0.51) | 6.44 (1.38) | 8, 16, 16, 32 | 2, 4, 4, 8 | 2, 4 |
| 4 larvae, 4 aliquots | 4.47 (0.44) | 5.98 (0.42) | 4.34 (0.47) | 0.75 (0.23) | 0.13 (0.32) | 6.16 (1.38) | 12, 24, 16, 32 | 3, 6, 4, 8 | 1, 2 |
| 8 larvae, 2 aliquots | 3.43 (0.77) | 7.58 (2.18) | 3.12 (0.75) | 0.45 (0.52) | 0.31 (0.54) | 6.96 (1.38) | 8, 16, 16, 32 | 1, 2, 2, 4 | 1, 2 |
| 8 larvae, 4 aliquots | 3.70 (0.92) | 6.58 (1.07) | 3.49 (0.88) | 0.56 (0.40) | 0.21 (0.64) | 6.68 (1.38) | 12, 24, 16, 32 | 1.5, 3, 2, 4 | 0.5, 1 |

**DxA summary**

This interaction is significant for only the ANOVA for Prime female age at pupation. This interaction doesn’t involve either the food level or timespan, so the difference across the interaction is the number of aliquots in the larval period. Density has a larger effect than aliquot and the ages at pupation are according to the expected main effects. The asymmetry that causes this interaction for the Prime female age appears to be the large difference due to density in the 2 aliquot treatment compared to the much smaller effect in the 4 aliquot treatment. Prime females pupate at almost the same age at low density regardless of aliquot treatment. Both low density treatments pupate earlier than projected, but the 2 aliquot treatment is much earlier compared to the main effects. At the high density, Prime females pupate much later in the 2 aliquot treatment than in the 4 aliquot treatment. The Prime female mass at pupation, the estimated growth rate and the food/larva after day 4 all show the same order as the Prime female age, but do not explain why the two low density treatments are so similar. In other significant interactions for the Prime female age at pupation, increased total food is related to earlier pupation. Total food after day 4 does not appear to be related to age at pupation in this interaction; the highest food/larva after day 4 (low density, 4 aliquots) pupates earliest followed by the next highest food/larva (low density, 2 aliquots). These two treatments are much closer to each other than expected based on the difference in the food/larva. The effect must be due to the addition of the aliquots. The test tubes with low density and 2 aliquots receive an average of 3 mg food/larva on day 0. They receive another 1.5 mg food/larva (average) on day 3 and another 1.5 mg on day 6. The test tubes with low density and 4 aliquots receive an average of 1.5 mg food/larva on day 0, 0.75 mg more on day 1, 1.5 mg more on day 2, 0.75 mg more on day 3, 0.75 mg more on day 4 and 0.75 mg more on day 6. The Prime females in both these treatments mostly pupate before the food addition on day 6. Prime females pupate relatively early in the test tubes with the most initial food (low density and 2 aliquots), although they don’t grow as large, as fast or pupate as early as those in the test tubes with more food during the larval period (low density and 4 aliquots). This suggests that the initial availability of food affects the trigger for pupation for females. In this interaction, the large initial input (low density, 2 aliquots) results in much earlier pupation than expected for the Prime female while not affecting Prime female mass or any other dependent variables. The longer delay in pupation for the Prime females in the high density, 2 aliquot test tubes is likely due to the low food/larva after day 4. This interaction appears to be due to exponential growth processes rather than competition.

**The DxT interaction (R squared = 0.84)**

The interaction between density and timespan shows the residual effect after the higher order interactions have been explained (FxDxT for all variables, FxDxAxT for Average female mass, DxAxT for Prime female mass and Average male mass). This interaction is significant for all 7 variables in the ANOVAS and the coefficients for this interaction in the MANOVA are all non-zero. This interaction has the second largest R squared value of any interaction in the MANOVA. For the four mass variables and the two age variables the main effects are: low density is better than high density and the 3 day timespan is better than the 6 day timespan. For Survival low density is better than high density, but the 6 day timespan is better than the 3 day timespan.

Because this interaction involves density and an attribute of the food supply, it is possible that competition is involved. Food level (total food) and density are independent factors in the experiment, and they jointly affect competition among the mosquito larvae, but growth and competition among mosquito larvae are also affected by food/larva, which is necessarily confounded with both food level and density. In the interaction between density and timespan, food level is either 16 mg or 32 mg, delivered over 3 days or 6 days at each density; the food level is not involved in the interaction. However, the food/larva is 4 mg/larva or 8 mg/larva at the low density and 2 mg/larva or 4 mg/larva at the high density. The joint effect of food and density (including the food/larva effect) is represented in the food x density interaction (FxD), and interacts with timespan in the FxDxT interaction, so the DxT interaction reflects the residual effects of density and timespan after the higher order interactions have been accounted for (FxDxT for all the variables, DxAxT for Prime female mass and Average male mass, FxDxAxT for Average female mass). The interpretation of the DxT interaction is that the number of larvae affects the growth of larvae at different timespan treatments. Timespan clearly affects the amount of food in the test tubes on a daily basis, so could be affecting the competition among larvae as well as growth of larvae.

Low density and the 3 day timespan treatment should provide the best growing conditions for the larvae; the food is delivered over 3 days, all the food is delivered before any larvae pupate, and low density means more food/larva and also less competition in the FxD set of interactions (above). The low density, 6 day timespan treatment provides less food during the early larval period and some of the food is delivered after the first larvae pupate; there should be more competition among the larvae because there is less food early in the larval period, but a release from competition due to the final aliquot of food on day 6. Increased competition shows up as a reduction in size of all larvae and as a reduction in the size distribution among females (as they switch from active filtering to retention at an earlier point in the larval lifespan). Increased competition reduces the size of males, and increases the size distribution among males, but the release from competition can reduce the size distribution. The high density, 3 day timespan treatment also provides good growing conditions, but the larger number of larvae decrease the available food and may increase the competition for food. Both effects of the larger number of larvae decrease the size of the larvae; competition should decrease the size distribution among females and increase the size distribution among males (the relative advantage of the Prime over the Average individual). There is no late release of competition from the final aliquot (delivered on day 3). The high density, 6 day timespan treatment provides less food early in the larval period and more larvae. The pupae should be smallest and the size distribution of the females should be smallest (due to competition and the change in feeding behavior to retention). The size distribution of males should increase due to competition, but may decrease due to the release from competition and the late food input. Competition should also reduce the size of the Prime male relative to that of the Prime female and reduce the size of the Average male relative to that of the Average female, but that may be obscured by the release of competition due to the day 6 input of food.

Because this interaction could indicate competition among the larvae, each dependent variable is examined for a resemblance to the set of FxD interactions. If variable doesn’t appear match the pattern of competition, it must be due to exponential growth processes in response to the availability of food.

S44 Table shows the means and standard errors for Survival. This interaction explains 2% of the variance in Survival across the experiment, but it is one of only three significant interactions for Survival.

1. The arcsine transformed percent survival is higher at both low density treatments than at either high density treatment, so density has a much greater effect on Survival than timespan.
2. Within each density, Survival is better at the 6 day timespan than the 3 day timespan.
3. Survival is best at low density and the 6 day timespan and worst at the high density and the 3 day timespan. The interaction appears to be due to the much better Survival at low density and the 6 day timespan than at any other treatment.
4. The range of the expected values (based on the main effects) is 1.15 to 1.28 (0.13, all units are for arcsine transformed percent survival). The actual range of means is 1.11 to 1.36 (0.25).
5. The low density, 6 day timespan does better than expected (0.08 better), but the other three treatments are all lower than projected (0.01 to 0.05 lower). The low density treatment is better for Survival, and the 6 day timespan is better than the 3 day timespan, but the low density treatment with the 6 day timespan is the only one where Survival is better than projected.
6. The interaction between density and timespan, an attribute of the food supply, may indicate competition among the larvae affecting survival. In this case, the low density, 6 day timespan appears to increase survival. This is not the treatment where competition would be lowest, so this may be related to the availability of food (see FxT above).

S44 Table. Means (SE) for arcsin transformed percent Survival for the interaction DxT. Expected values.

| Density x Timespan | Survival | Expected mean values of Survival |
| --- | --- | --- |
| 4 larvae, 3 days | 1.23 (0.13) | 1.24 (0.17) |
| 4 larvae, 6 days | 1.36 (0.11) | 1.28 (0.17) |
| 8 larvae, 3 days | 1.11 (0.17) | 1.15 (0.17) |
| 8 larvae, 6 days | 1.14 (0.18) | 1.19 (0.17) |

The S45 Table shows the means and standard errors for the Prime female mass and age at pupation and the Average female mass at pupation for the DxT interaction. This contrast accounts for a similar amount of variance for each of the three variables (r squared = 0.10, 0.11, 0.10, respectively). This is the second most important interaction for the two mass variables (by r squared value) and the most important for the Prime female age at pupation. Because this interaction involves density and an attribute of the food supply, it is possible that the interaction describes competition among females. A heuristic 3D plot of the Prime female mass and age at pupation is presented in S34 Fig.

1. The largest value of the Prime female mass is in the low density, 3 day timespan treatment (4.62 mg). The smallest value is in the high density, 6 day timespan treatment (3.34 mg). The other two treatments are intermediate, but both low density treatments are larger than either high density treatment. Density is more important than timespan for Prime female mass. The 3 day timespan treatments are larger than the 6 day timespan treatments within each density, but the difference between the timespan treatments is larger at the low density (0.58 mg) than at the high density (0.45 mg).
2. The column in S45 Table labeled “Expected mean values for Prime female mass at pupation” shows the projected mean values based on the main effects of density and timespan (see explanation for S27 Table). The range of the expected means is 3.63 mg to 4.27 mg, a span of 0.64 mg. The range of the actual means is 3.34 mg to 4.62 mg, a span of 1.28 mg, twice the span of the expected means.
3. The highest value of the Prime female mass is 0.35 mg larger than the projected value and the lowest is 0.29 mg lower than the projected value. The intermediate treatments are closer to the projected values. The asymmetry that causes this interaction for the Prime female mass appears to be the larger than expected mass at low density and 3 day timespan and the smaller than expected mass at the high density and 6 day timespan.
4. The Prime female age at pupation is best (earliest) at the low density, 3 day timespan (5.68 days) and latest at the high density, 6 day timespan (7.96 days). These are also the best and worst treatments for Prime female mass. Prime female age differs from the Prime female mass in that the two 3 day timespan treatments are both lower than the two 6 day timespan treatments; for Prime female age, timespan is more important than density. Within each timespan treatment, the low density pupates before the high density. There is a small difference between the density treatments at the 3 day timespan (0.51 days), but a much larger difference between the density treatments at the 6 day timespan (1.56 days, three times greater).
5. The column labelled “Expected mean values for Prime female age at pupation” shows the projected mean values based on the main effects of density and timespan. The range of the expected mean values is from 5.99 days to 7.13 days, a span of 1.14 days. The observe range is from 5.68 days to 7.96 days, a span of 2.28 days, twice as large.
6. The lowest age at pupation is 0.31 days lower than projected, but the latest age at pupation is 0.83 days later than projected. Both of the intermediate treatments are also lower than projected (0.21 days for low density, 6 day timespan and 0.32 days for high density, 3 day timespan). Either increasing the density from 4 to 8 larvae per test tube, or increasing the timespan from 3 days to 6 days increases the age at pupation, but increasing both the density and timespan increases the age at pupation disproportionately more. This appears to be the source of this interaction for the Prime female age at pupation.
7. In contrast to the clear dominance of the density treatment over the timespan treatment for the mass variable, the Prime female age at pupation is relatively more affected by the timespan treatment. The Prime females pupate earliest at the low density and 3 day timespan and latest at the high density and 6 day timespan, but the Prime females at the low density and 6 day timespan pupate later than those at the high density and 3 day timespan. For the Prime female age at pupation, the interaction results from the much later pupation in the high density, 6 day timespan test tubes.
8. The Prime females grow largest and pupate earliest in the test tubes with the low density and 3 day timespan. They pupate between the 5th and 6th day, but all of the food has been added by the 3rd day. The next largest females (low density, 6 day timespan) delay pupation by 0.72 days, but most of them pupate before the addition of the last aliquot of food on day 6. The third largest females (high density, 3 day timespan) pupate earlier than the second largest females, but don’t grow as large; density has a greater effect on the Prime female mass than timespan. The smallest Prime females (high density, 6 day timespan) pupate almost 2 days after the last aliquot of food is added, but they do not grow as large as the high density, 3 day timespan Prime females. The late addition of food does not make up for the same amount of food provided earlier in the larval period. The small size and much later pupation of the Prime females in the high density, 6 day timespan treatment appears to cause the significant interaction for these two dependent variables.
9. The estimated growth rate combines the mass and age at pupation into a biologically meaningful number. The values of Prime female mass and age vary indirectly in this interaction; the largest mass occurs with the earliest age and vice versa. The low density, 3 day timespan treatment has a growth rate of 0.81 mg/day, almost twice that of the high density, 6 day timespan (0.42 mg/day). The intermediate treatments are about halfway in between the two extremes (0.63 mg/day for the low density, 6 day timespan; 0.61 mg/day for the high density, 3 day timespan). The females in the low density, 6 day timespan treatment grow larger than the ones in the high density, 3 day timespan, but take longer. The growth rates of the two intermediate treatments match the Prime female mass rather than the age at pupation. The r squared values of the two variables are almost the same (0.10 and 0.11, respectively).
10. The Average female mass is similar to the Prime female mass. The largest value is in the low density, 3 day timespan treatment (4.49 mg) and the smallest value is in the high density, 6 day timespan (3.05 mg). The two low density treatments result in larger Average females than the two high density treatments. Density is more important than timespan for the Average female (as it was for the Prime female). Within each density the 6 day timespan is smaller than the 3 day timespan. The difference between the two timespan treatments at low density (0.65 mg) is larger than the comparable difference at high density (0.51 mg), also parallel to the Prime female mass.
11. The column “Expected mean values for Average female mass at pupation” shows the projected means based on the main effects, density and timespan. The values at low density are larger than projected and the values at high density are smaller than projected. The range of the projected values is 3.37 mg to 4.10 mg, a span of 0.73 mg. The range of actual values is 3.05 mg to 4.49 mg, a span of 1.44 mg, almost twice as large. The largest value of Average female mass is 0.39 mg larger than projected and the smallest value is 0.32 mg smaller than projected. The two intermediate values are closer to the projections. The interaction appears to be caused by the more extreme values at the high and low end of the range, corresponding to the low density, 3 day timespan and the high density, 6 day timespan, respectively. This is also similar to the Prime females.
12. The difference in size between the Prime female mass and the Average female mass indicates the shape of the size distribution of females in each of these treatments. The smallest difference between Prime and Average females is in the test tubes with the low density and 3 day timespan (0.13 mg). The greatest difference is in the test tubes with the high density and 6 day timespan (0.29 mg). The low density, 6 day timespan treatments and the high density, 3 day timespan treatments are intermediate (0.20 mg and 0.23 mg, respectively). The difference in size between the Prime female mass and the Average female mass increases as their mass at pupation decreases. Females do not appear to switch from active filtering to retention in response to this interaction. This is different from the size distributions in the FxD set of interactions that describe competition. This interaction describes exponential growth processes.
13. In the FxD set of interactions, the Prime female mass follows the total food per test tube when the food/larva is greater than 4 mg (after day 4). The low density, 3 day timespan treatment is the only one where all the test tubes experience 4 mg food/larva or more after day 4. Both the total food after day 4 and the food/larva after day 4 are the highest across this interaction. These test tubes produce the largest Prime and Average females and the Prime females pupate earliest.
14. The smallest Prime and Average females are in the high density, 6 day timespan treatment. These Prime females pupate latest. All the test tubes in this treatment experience food/larva less than 4 mg (after day 4). This should correspond to the most competition treatment in the FxD interaction, but the small sizes of the Prime and Average females with the larger difference between them suggest that this interaction describes the growth of females rather than competition among them.
15. In the two treatments with the Prime and Average females in between the largest and smallest ones, half of the test tubes have food/larva levels of 4 mg or greater and half have food/larva levels less than 4 mg (after day 4). These are the low density, 6 day timespan treatment and the high density, 3 day timespan treatment. The total food is lower for the low density, 6 day timespan (12 mg, 24 mg), but the food/larva is higher (3 mg, 6 mg). This results in larger Prime and Average females, but the Prime female takes longer to pupate. The total food is higher for the high density, 3 day timespan treatment (16mg, 32 mg), but the food/larva is lower (2 mg, 4 mg). This accounts for the smaller sizes of the Prime and Average females and the earlier pupation compared to the low density, 3 day timespan treatment (because the total food is higher).
16. The DxT interaction for the female variables appears to be explained by the amount of food in the test tubes after day 4, and to describe exponential growth processes. There does not appear to be any anomaly that reflects competition among the females.

S45 Table. Means (SE) for Prime female mass and age at pupation and Average female mass at pupation for the interaction DxT. Expected mean values, growth rates, differences between Prime and Average female masses, food levels.

| Density x Timespan | Prime female mass at pupation (mg) | Prime female age at pupation (days) | Average female mass at pupation (mg) | Estimated Prime female growth rate (mg/day) | Prime female mass MINUS Average female mass (mg) | Expected mean values for Prime female mass at pupation (mg) | Expected mean values for Prime female age at pupation (days) | Expected mean values for Average female mass at pupation (mg) | Total food after day 4 (mg) | Food/larva after day 4 (mg) |
| --- | --- | --- | --- | --- | --- | --- | --- | --- | --- | --- |
| 4 larvae, 3 days | 4.62 (0.24) | 5.68 (0.31) | 4.49 (0.28) | 0.81 (0.16) | 0.13 (0.18) | 4.27 (0.77) | 5.99 (1.38) | 4.10 (0.81) | 16, 32 | 4, 8 |
| 4 larvae, 6 days | 4.04 (0.66) | 6.40 (1.05) | 3.84 (0.70) | 0.63 (0.39) | 0.20 (0.48) | 4.01 (0.77) | 6.61 (1.38) | 3.81 (0.81) | 8, 12, 16, 24 | 2, 3, 4, 6 |
| 8 larvae, 3 days | 3.79 (0.92) | 6.19 (0.86) | 3.56 (0.88) | 0.61 (0.39) | 0.23 (0.64) | 3.89 (0.77) | 6.51 (1.38) | 3.66 (0.81) | 16, 32 | 2, 4 |
| 8 larvae, 6 days | 3.34 (0.72) | 7.96 (1.94) | 3.05 (0.71) | 0.42 (0.43) | 0.29 (0.51) | 3.63 (0.77) | 7.13 (1.38) | 3.37 (0.81) | 8, 12, 16, 24 | 1, 1.5, 2, 3 |

S46 Table shows the means and standard errors for the Prime male mass and age at pupation and the Average male mass at pupation for the DxT interaction. This interaction is several times more important for the Prime male age (r squared = 0.17) than for either of the mass variables (r squared = 0.03 for the Prime male mass and 0.06 for the Average male mass). It is the most important interaction (by r squared value) for the Prime male age, but ranks in the middle of the interactions for both mass variables. Density x timespan explains 3% of the variance in the ANOVA for the Prime male mass. Three other interactions explain more variance (FxDxT, FxT, and AxT). This interaction explains 6% of the variance in the ANOVA for Average male mass, twice the explained variance of the Prime male mass. Two interactions explain more of the variance for Average male mass (FxDxT and FxT). Because this interaction involves density and an attribute of the food supply, it is possible that competition is involved. A heuristic 3D plot of the Prime male mass and age at pupation is presented in S35 Fig.

1. The largest value of the Prime male mass is in the low density, 3 day timespan treatment (2.70 mg). The smallest value is in the high density, 6 day timespan (1.92 mg). The other two treatments are intermediate, but both low density treatments are larger than either high density treatment. Density is more important than timespan for the mass of the Prime male. The 3 day timespan treatments are larger than the 6 day timespan treatments within each density, but the difference between the timespan treatments is larger at the high density (0.50 mg) than at the low density (0.22 mg). The high density, 3 day timespan treatment is more like the two low density treatments than it is like the high density, 6 day timespan treatment. The three largest Prime males are grouped (a span of 0.28 mg) with a gap between the lowest in the group and the high density, 6 day timespan treatment (a gap of 0.50 mg). This is likely the source of the interaction for Prime male mass.
2. The column in S46 Table labelled “Expected mean values for Prime male mass at pupation” shows the projected mean values based on the main effects of density and timespan. The range of the expected means is 2.19 mg to 2.58 mg, a span of 0.39 mg. The range of the actual means is 1.92 mg to 2.70 mg, a span of 0.78 mg, twice the span of the expected means. The highest value of the Prime male mass is 0.12 mg larger than the projected value and the lowest is 0.27 mg lower than the projected value. The intermediate treatments are closer to the projected values, but both observed means are larger than the projected means. The asymmetry that causes this interaction for the Prime male mass appears to be the larger than expected masses in the group of large Prime males and the much smaller than expected mass at the high density and 6 day timespan.
3. All the Prime males pupate before the final aliquot is delivered in the 6 day timespan treatment. The Prime male age at pupation is earliest in the test tubes with low density and the 6 day timespan, and latest at the high density and 6 day timespan. The two 3 day timespan treatments are intermediate, but more like the earliest than the latest. The three earliest are grouped (a span of 0.10 days) and separated by a gap of 0.25 days from the latest. The asymmetry that causes the interaction is the disproportionately late pupation for the Prime male in the high density, 6 day timespan treatment. This treatment is also the one with the smallest mass for the Prime male.
4. The column in S46 Table labelled “Expected mean values of Prime male age at pupation” shows the projected mean values based on the main effects of density and timespan. The range of expected means is 5.06 days to 5.19 days, a span of 0.13 days. The range of the observed means is 5.00 days to 5.35 days, a span of 0.35 days, more than twice the span of the expected means.
5. The expected values of age at pupation correspond inversely with the Prime male masses at pupation, but the observed values of age at pupation do not. The low density, 3 day timespan treatment (largest Prime male) and the high density, 6 day timespan treatment (smallest Prime male) both pupate later than projected. The two intermediate-sized Prime males, the low density, 6 day timespan and the high density, 3 day timespan treatments, both pupate earlier than projected. The low density, 3 day timespan treatment (corresponding to the largest Prime male) has the most food, suggesting that these Prime males delay pupation to grow larger (see S45 Table for food levels by treatment). This is one of the asymmetries that causes this interaction.
6. There is also no gap between the expected values of the earlier three and the latest of the ages at pupation, so the expected value of age at pupation in the high density, 6 day timespan treatment suggests another asymmetry.
7. The estimated growth rate combines the Prime male mass and Prime male age at pupation in a single, biologically meaningful number. The fastest growth rate is an indication of the best growing conditions and possibly the least competition. The growth rates correspond to the Prime male mass, not the Prime male age. This interaction explains less of the variability in the Prime male mass than the Prime male age, but the numerical differences across the mass variable are much greater than those for the age variable, so the estimated growth rate is determined by the mass. There is a large gap between the estimated growth rates in the three treatments with the largest and earliest Prime males to pupate, and the high density, 6 day treatment that produces the smallest and latest Prime males. This gap also resembles the pattern of the mass variable rather than that of the age variable.
8. Prime males grow largest in the low density, 3 day timespan treatment, but take longer to pupate (5.10 days instead of 5.00 days). Despite delaying pupation (by 0.10 days) the estimated growth rate is highest in this treatment. This treatment has the most total food and the food/larva (after day 4). It seems probable that the Prime males delay pupation in order to grow larger compared to the other treatment combinations in this interaction.
9. The Prime males in the low density, 6 day timespan treatment are second largest, and grow almost as fast as the largest males, but pupate earliest (5.00 days).
10. The Prime males in the high density, 3 day timespan treatment grow almost as large and pupate slightly later than those in the low density, 6 day timespan treatment. The estimated growth rate in these test tubes is almost as high as in the previous two treatments. These Prime males have more total food than in the low density, 6 day timespan treatment, but lower food/larva (after day 4), so they pupate slightly smaller and slightly later.
11. The Prime males in the high density, 6 day timespan treatment are smallest, pupate latest, and have a slower estimated growth rate than the other three treatments. These males have less food than the high density, 3 day timespan Prime males and delay pupation significantly but do not grow as large. They have the least food across the interaction (both total food after day 4, and food/larva after day 4); they pupate later and do not grow as large, or as fast, as any of the other three treatments.
12. The Average male mass corresponds to the Prime male mass; the largest Average males are in the low density, 3 day timespan test tubes (2.63 mg) and the smallest ones are in the high density, 6 day timespan (1.93 mg). The low density Average males are larger than any high density Average males, so density is more important than timespan. Within each density, the Average males in the 3 day timespan treatment are larger than those in the 6 day timespan.
13. The Average male mass is not as tightly grouped as the Prime male mass; the three treatments with largest Average males span 0.35 mg and the difference between the smallest of those three and the smallest Average male mass in the high density, 6 day timespan is also 0.35 mg.
14. The column in S46 Table labelled “Expected mean values of Average male mass at pupation” shows the projected mean values based on the main effects of density and timespan; they correspond to the observed means in rank order. The projected means range from 2.14 mg to 2.49 mg, a span of 0.35 mg. The observed means range from 1.93 mg to 2.63 mg, a span of 0.70, twice the span of the projections. The largest observed value is 0.14 mg greater than the projection and the second largest is 0.08 mg larger. Both are at the low density. The third largest is equal to the projection, but the smallest observed value is 0.21 mg less than the projection. These are both high density treatments.
15. The largest Average male mass is larger than the projection and the smallest is smaller than the projection. This is likely one source of the asymmetry that causes this interaction.
16. Another asymmetry is that the Average male mass is larger than the Prime male mass in the high density, 6 day timespan (the smallest Prime and Average males).
17. The difference between Prime male mass and Average male mass indicates changes in the distribution of sizes across the treatments as well as the relative advantage of the Prime over the non-Prime males. In the treatment combination low density and 3 day timespan, where both Prime and Average males grow largest, and the Prime males have the fastest growth rate, the difference between the Prime and Average males is 0.07 mg. These test tubes provide the optimal growth conditions for the male larvae across this interaction, so this difference between Prime and Average male masses represents growth under the most benign conditions (most food, potentially the least competition).
18. At the high density and the 3 day timespan, both the mass variables are smaller than in the low density, 3 day timespan treatment, but the difference between the Prime and Average male masses is larger, 0.14 mg.
19. The differences between the Prime and Average male masses in the test tubes with the 6 day timespan are smaller than the differences at the 3 day timespan. In the test tubes with the 6 day timespan, the non-Prime males grow larger relative to the Prime males, probably on the additional food on day 6. The non-Prime males in the high density, 6 day timespan treatment grow larger than the Prime male (1.92 mg for the Prime male and 1.93 mg for the Average male mass). The effect is not as great at the low density (2.48 mg for the Prime male and 2.44 mg for the Average male).
20. Timespan has a different effect on the Prime and Average males. The Prime male experiences the 6 day timespan as lower total food because all the Prime males pupate before the addition of the day 6 aliquot of food. The non-Prime males (in Average male mass) experience the 6 day timespan as more total food because the day 6 aliquot is added after the Prime male pupates.
21. At low density Prime and Average males grow best and Prime males grow fastest. The food is delivered earlier in the larval period in the 3 day timespan, and there is more total food during the larval period of the Prime male, so all the males grow larger in this treatment. The difference between the Prime and Average males is smallest and this suggests that these test tubes represent optimal growth across this interaction.
22. At low density and the 6 day timespan, the food is delivered more slowly and there is less total food during the larval period of the Prime male. All of the males are smaller than in the previous treatment, and the Prime male grows more slowly despite pupating earlier. The Average male mass is relatively larger compared to the Prime male mass because the non-Prime males grow larger on the day 6 input of food after the Prime male pupates (mostly on day 5).
23. At the high density the males in the 3 day timespan treatment also grow larger than the males in the 6 day timespan treatment, but the relationship between the Prime male and the Average male is altered. At the high density, the Prime male grows larger relative to the Average male in the 3 day timespan treatment, but the Average male grows larger (relatively and absolutely) than the Prime male in the 6 day timespan.
24. Food level is not involved in this interaction, so timespan is the attribute of the food supply that could affect competition. The food is delivered over 3 days, or 6 days. In the test tubes with the 3 day timespan, all the food is delivered by day 3 and differences in feeding ability or competition among the males determine the size of the Prime male and Average male. The high density Prime and Average males should experience more competition than the low density ones; both the Prime male mass and the Average male mass are lower at the high density, but the difference between them is greater as well. In the test tubes with the 6 day timespan, the Prime males pupate before the final input on day 6, but some or all of the non-Prime males grow larger on the extra food. The Average males at the low density are only 0.04 mg smaller than the Prime males (compared to 0.07 mg for the Average males at the 3 day timespan). The Average males at the high density are 0.01 mg larger than the Prime males. This could reflect either a release from competition or an abundance of food, or both. The non-Prime males in both treatments with the 6 day timespan take advantage of the additional food delivered on the 6th day to grow larger. At least one of the non-Prime males in the high density, 6 day timespan treatment must grow larger than the Prime male. Nevertheless, the Average males do not grow as large as their counterparts in the 3 day timespan treatments; additional food later in the larval lifespan does not benefit the larvae as much as the equivalent food early in the larval period. Similarly, a release from competition late in the larval period does not benefit the non-Prime males as much as more food or lower density earlier.
25. There are two characteristics of the FxD set of interactions regarding competition among males. At the higher density of the two intermediate competition treatments the difference between the Prime and Average males is larger than in any other treatment, and in the treatment with the smallest Prime and Average males, the non-Prime males grow larger than the Prime male due to release from competition and the additional food on day 6 after the Prime male pupates. Both of these characteristics appear in this interaction suggesting that the males are competing for food availability based on the timespan rather than the food level. This is different from the females (above).

S46 Table. Means (SE) for Prime male mass and age at pupation and Average male mass at pupation for the interaction DxT. Expected mean values, growth rates, differences between Prime and Average male masses.

| Density x Timespan | Prime male mass at pupation (mg) | Prime male age at pupation (days) | Average male mass at pupation (mg) | Estimated Prime male growth rate (mg/day) | Prime male mass MINUS Average male mass (mg) | Expected mean values of Prime male mass at pupation (mg) | Expected mean values of Prime male age at pupation (days) | Expected mean values of Average male mass at pupation (mg) |
| --- | --- | --- | --- | --- | --- | --- | --- | --- |
| 4 larvae, 3 days | 2.70 (0.08) | 5.10 (0.06) | 2.63 (0.05) | 0.53 (0.01) | 0.07 (0.01) | 2.58 (0.44) | 5.06 (0.24) | 2.49 (0.39) |
| 4 larvae, 6 days | 2.48 (0.37) | 5.00 (0.00) | 2.44 (0.41) | 0.50 (0.14) | 0.04 (0.31) | 2.40 (0.44) | 5.11 (0.24) | 2.36 (0.39) |
| 8 larvae, 3 days | 2.42 (0.36) | 5.05 (0.10) | 2.28 (0.33) | 0.48 (0.14) | 0.14 (0.24) | 2.37 (0.44) | 5.14 (0.24) | 2.28 (0.39) |
| 8 larvae, 6 days | 1.92 (0.53) | 5.35 (0.43) | 1.93 (0.37) | 0.36 (0.47) | -0.01 (0.42) | 2.19 (0.44) | 5.19 (0.24) | 2.14 (0.39) |

**Males versus females**

S47 Table shows the Prime female mass, the differences between the Prime female mass and the Average female mass, between the Prime male mass and the Average male mass, between the Prime female mass and the Prime male mass, and between the Average female mass and the Average male mass. These differences between the mass variables indicate differences in the size distributions among the larvae and the relative advantages of females over males. A heuristic 3D plot of the masses of Prime and Average females, and Prime and Average males, against the average food/larva after day 4 is presented in S36 Fig.

1. Females outcompete males by their larger size (increased filtering ability) and by their presumed change in behavior to retain particles as the relative density of particles decreases. The males grow and compete in an environment controlled by the females. The difference between the Prime male mass and the Prime female mass indicates changes in the relative advantage of the Prime females over the Prime males. The largest Prime males occur in the same treatment as the largest Prime females (low density, 3 day timespan) and the smallest Prime males occur in the same treatment as the smallest Prime females (high density, 6 day timespan).
2. As the Prime female decreases in size across the 4 treatment combinations, the difference between the Prime female and the Prime male also decreases (1.92 mg, 1.56 mg, 1.37 mg), except in the worst treatment combination, high density and 6 day timespan (1.42 mg). Both the Prime female and the Prime male are disproportionately small relative to the other three treatments at the high density, 6 day timespan, yet the Prime males are at a greater disadvantage in this treatment with the least food and/or the most competition.
3. One explanation is that the Prime males pupate before the final aliquot of food on day 6; they pupate at 5.35 days. The females pupate after the final aliquot of food at 7.96 days; they have more food than the males. Despite the extra food, these Prime females do not grow as large as in the other treatments. Prime males may be subject to intense competition and reduced food levels, but Prime females are also adversely affected by these conditions.
4. The difference in age at pupation between the Prime male and the Prime female does not indicate relative advantage, but it does highlight the difference in strategy between Prime females and males in the different treatments. Prime females pupate after Prime males. In the three treatments associated with the largest Prime females and Prime males, the differences are 0.58 days, and 1.14 days, in the two 3 day timespan treatments, and 1.40 days, in the low density, 6 day timespan. The difference between the ages at pupation in the high density, 6 day timespan is 2.61 days; the Prime females pupate disproportionately later than the Prime males.
5. The difference between the Average female mass and the Average male mass also indicates changes in relative advantage of females over males. The Average male mass also decreases as the Average female mass decreases, and the difference between the Average female and male masses decreases as the Average female mass decreases.
6. The Prime female mass and Prime male mass are included in the Average female and male masses, so this comparison is not independent of the difference between the Prime female and male masses above. Both differences are largest in the low density, 3 day timespan treatment, where all four mass variables are largest. The differences in both comparisons decrease as the food/larva (after day 4) decreases. The difference between the two Average masses is always smaller than the difference between the two Prime masses. As the food/larva decreases across the 4 treatments, the relative advantage of females over males also decreases. This is in contrast to the size distributions with each sex.
7. Nevertheless, the Prime female has a greater advantage over the Prime male in the high density, 6 day timespan treatment, while the Average female has a smaller advantage over the Average male. Both the Prime female and the non-Prime males grow larger on the final aliquot after the Prime male pupates. The Average female probably also grows larger on the final aliquot on day 6, but does not benefit differentially as the non-Prime males do over the Prime male.
8. The relatively small difference between the Prime female mass and the Prime male mass in the high density, 3 day timespan treatment corresponds to the largest difference between the Prime male mass and the Average male mass. This suggests that Prime males do better in relation to the Prime females in this treatment as well as against the non-Prime males.

S47 Table. Means (SE) for Prime female mass at pupation for the interaction DxT. Differences between Prime and Average female masses, Prime and Average male masses, Prime female and Prime male masses, and Average female and male masses. Food/larva.

| Density x Timespan | Prime female mass at pupation (mg) | Prime female mass MINUS Average female mass (mg) | Prime male mass MINUS Average male mass (mg) | Prime female mass MINUS Prime male mass (mg) | Average female mass MINUS Average male mass (mg) | Food/larva after day 4 (mg) |
| --- | --- | --- | --- | --- | --- | --- |
| 4 larvae, 3 days | 4.62 (0.24) | 0.13 (0.18) | 0.07 (0.01) | 1.92 (0.13) | 1.86 (0.14) | 4, 8 |
| 4 larvae, 6 days | 4.04 (0.66) | 0.20 (0.48) | 0.04 (0.31) | 1.56 (0.38) | 1.40 (0.41) | 2, 3, 4, 6 |
| 8 larvae, 3 days | 3.79 (0.92) | 0.23 (0.64) | 0.14 (0.24) | 1.37 (0.49) | 1.28 (0.47) | 2, 4 |
| 8 larvae, 6 days | 3.34 (0.72) | 0.29 (0.51) | -0.01 (0.42) | 1.42 (0.45) | 1.12 (0.40) | 1, 1.5, 2, 3 |

**DxT summary**

Density and timespan jointly affect Survival and the growth of males and females. Survival is better at low density and best at low density and the 6 day timespan. There is only a small effect of timespan on Survival at the high density. This suggests that competition, which should be highest at high density and the 6 day timespan treatment, is not important in this interaction for Survival. It is more likely that the interaction is due to better Survival at low density and the longer timespan; other interactions suggest that larvae survive better when food additions are spread out across the larval period.

Referring to the discussion of FxD competition in the DxAxT interaction, the same relationships should apply to the DxT interaction.

If the DxT interaction is the result of competition for food, the females at the highest food/larva (4 mg food/larva or greater; see S45 Table for Total food after day 4 and Food/larva after day 4) should resemble the females in the least competition treatment in FxD, the females at the lowest food/larva should resemble the females in the most competition treatment, and the intermediate females should resemble the females in the intermediate competition treatments. The low density, 3 day timespan treatment has 4 mg or 8 mg food/larva by day 3, so it should resemble the least competition treatment. The high density, 6 day timespan treatment has much less food (1 mg, 1.5 mg, 2 mg or 3 mg food/larva by the end of day 4); this is the lowest food/larva across this interaction, so it should resemble the most competition treatment. However, the two intermediate treatments are not precisely similar to the intermediate competition treatments. The high density, 3 day timespan treatment receives 2 mg or 4 mg food/larva at the end of day 3, but the low density, 6 day timespan treatment receives 2 mg, 3 mg, 4 mg or 6 mg food/larva at the end of day 4. By analogy with the FxD interaction, the Prime female should be larger in the high density intermediate food level, but in the DxT interaction, the low density intermediate food level does have more food.

For females, the largest Prime female mass, Average female mass, estimated growth rate, and the earliest age at pupation all occur in the low density, 3 day timespan treatment. This treatment is also associated with the smallest difference in mass between the Prime and Average females, and the largest positive deviations from the expected values for the masses of the Prime and Average females. All these indicators line up with the least competition treatment in the FxD set of interactions. The smallest Prime female mass, Average female mass, estimated growth rate, and the latest age at pupation occur in the high density, 6 day timespan treatment. This treatment is also associated with the largest difference in mass between the Prime and Average females, and the largest negative deviations from the expected values for the masses of the Prime and Average females. This doesn’t correspond entirely with the outcomes from the most competition treatment (FxD set of interactions); specifically, at low food levels the Prime female should switch from actively filtering to retention and this should result in a smaller size distribution between the Prime and Average females. Despite the low food/larva level, the ongoing food inputs over the timespan appear to allow the females to actively filter particles rather than switching to retention.

The two intermediate treatments fall between the low density, 3 day timespan and the high density, 6 day timespan treatments for the masses of the Prime and Average females, the estimated growth rates, the ages at pupation, the differences in mass between the Prime and Average females, and the deviations from the expected values. Half of the test tubes with the high density, 3 day timespan treatment have more total food after day 4 (32 mg) than any of the test tubes with the low density, 6 day timespan treatment. However, half of the test tubes with the low density, 6 day timespan treatment have more food/larva after day 4 (2 mg, 3 mg, 4 mg, 6 mg versus 2 mg, 4 mg). If competition were the predominant factor influencing the growth of these females, the high density, 3 day timespan treatment should produce the larger, faster growing, earlier pupating females with a larger difference between the Prime and Average females. This is not true, except that the Prime females in the high density, 3 day timespan treatment pupate slightly earlier than those in the low density, 6 day timespan treatment. With the exception of the ages at pupation, the outcome of this interaction for females seems best explained by the food/larva rather than total food or competition. The DxT interaction appears to describe exponential growth processes for females.

For males, the largest Prime male mass, Average male mass, and estimated growth rate, all occur in the low density, 3 day timespan treatment. This treatment is also associated with the largest positive deviations from the expected values for the masses of the Prime and Average males, but not with the earliest age at pupation or the smallest difference in mass between the Prime and Average males*.*  All these indicators line up with the least competition treatment in the FxD set of interactions. The smallest Prime male mass, Average male mass, estimated growth rate, and the latest age at pupation occur in the high density, 6 day timespan treatment. This treatment is also associated with the largest negative deviations from the expected values for the masses of the Prime and Average males, but not with the largest difference in mass between the Prime and Average males. Non-Prime males grow larger after the pupation of the Prime males, and especially on the final input of food on day 6. Some non-Prime males in the high density, 6 day timespan grow larger than the Prime male in that treatment. For males, this interaction resembles the competition in the FxD set of interactions.

For males, the two intermediate treatments also fall between the low density, 3 day timespan and the high density, 6 day timespan treatments for the masses of the Prime and Average males, the estimated growth rates and the expected mean values of masses, but not for the ages at pupation, the differences between the Prime and Average male masses, and the expected mean values of ages. As explained for the female larvae in the DxT interaction above, the larvae in the low density, 6 day timespan treatment have more food/larva after day 4 than the high density, 6 day timespan treatment so they grow larger. In contrast to the females, the difference between the Prime and Average male masses is largest in the high density, 6 day timespan. This resembles the competitive interaction (FxD set of interactions) and differs from the outcome for the females.

For males, the congruence between the Prime male mass, the Average male mass, the estimated growth rate, the difference between the Prime male and the Average male and the deviations from the expected values of the masses are clear. Based on the outcomes for the mass of males, the DxT interaction looks like males are competing for food due to the timespan treatment.

This is not true of the ages at pupation for males or for females. The Prime female age at pupation in the FxDxT interaction follows the total food after day 4. The Prime female age at pupation in the FxD interaction is affected by both the food/larva and the total food. The Prime females in the two FxD intermediate competition treatments have the same food/larva after day 4; the Prime females with the higher total food (high food, high density) pupate earlier than those with the lower total food (low food, low density). The Prime females in the intermediate treatments for the DxT interaction pupate earlier in the test tubes with less food/larva after day 4, but the higher total food. Total food appears to be more important to the Prime female age at pupation that the food/larva. The Prime female mass and the Average female mass follow the food/larva rather than the total food.

The Prime males pupate earlier than projected in three of the four treatments in the DxT interaction. They pupate latest in the high density, 6 day timespan treatment which appears to correspond to the most competition treatment in the FxD set of interactions. The Prime males pupate later than the projected value in this treatment as well. Competition delays pupation of the Prime male, although all the Prime males pupate before the final input of food on day 6.

The Prime male ages at pupation in the other three treatments do not correspond to the food/larva or the total food after day 4. The treatment with the earliest pupation is the low density, 6 day timespan treatment, which has less food (both food/larva and total food) than the low density, 3 day timespan treatment. It appears that the Prime males in the low density, 3 day timespan treatment delay pupation due to high levels of food. It appears that high levels of total food cause Prime males to delay pupation perhaps to grow larger or to improve physiological status. The Prime males in the high density, 3 day timespan treatment also delay pupation relative to the low density, 6 day timespan treatment; the food/larva after day 4 is less than in the earliest pupating treatment, but the total food is higher. This treatment also appears associated with increased competition among males; both more abundant food and more competition increase the age at pupation for males in other interactions.

In other FxD competition interactions, the Prime males appear to delay pupation in some treatments when food is available and also when competition is intense. It is not clear what causes the Prime male to delay pupation when food is available; neither food/larva nor total food consistently predict this response. Prime males consistently delay pupation when competition is intense, but do not delay it long enough to benefit from the additional food on day 6 (in the day 6 timespan treatments).

Density and timespan jointly affect the mass and age variables for males and females, but they affect them differently for the two sexes. Females grow largest, fastest, the Prime females pupate earliest, and the relative advantage of the Prime female over the non-Prime females is smallest at the low density and 3 day timespan. Either the 6 day timespan or the high density reduce the size, the growth rate, and increase the age at pupation and the relative advantage of the Prime female over the non-Prime females. The test tubes with the high density and the 6 day timespan produce the smallest females, the slowest growth rate, the latest age at pupation, and the largest relative advantage of the Prime female over the non-Prime females. The females do disproportionately well in the low density, 3 day timespan and disproportionately poorly in the high density, 6 day timespan treatments. The interaction between density and an attribute of the food supply could be an indication of competition independent of the food level, but there is no evidence for it. As the food availability early in the larval period changes due to a longer timespan and higher density, the females all pupate at smaller sizes, grow more slowly, and the size distribution increases. The simple explanation is that these are the result of exponential growth processes rather than competition, because females should switch from active filtering to retention at low food levels (and they appear to do so in other interactions in this experiment), and that would cause the size distribution to compress rather than to increase as observed.

Males are also largest and grow fastest at the low density, 3 day timespan treatment, and smallest, grow slowest and pupate latest at the high density, 6 day timespan treatment. They differ from the females in that the earliest age at pupation is in the low density, 6 day timespan treatment and relative advantage of the Prime male over the non-Prime males is more affected by timespan than by density. The size distribution of the males increases with density in the 3 day timespan treatments, similar to that of the females. However, the size distribution of males decreases with increasing density in the 6 day timespan treatments, probably due to the added food after the Prime male pupates. In contrast with the females in this interaction, the Prime male mass and age, the Average male mass, and the difference between the Prime and Average male masses jointly resemble the pattern in the set of FxD interactions that describe competition (above). This suggests that males are competing for food in response to the changes in availability caused by the timespan treatment independently of the food level treatment.

The estimated growth rates for Prime males and females decrease from the best treatment (low density, 3 day timespan) to the worst (high density, 6 day timespan), but there are differences that reflect differences in both the masses and ages between the sexes. Prime females grow much faster in the best treatment and much slower in the worst, with the two middle treatments being close together and about halfway between the extremes. The Prime females in the best treatment are larger than expected and pupate earlier than expected; those in the worst treatment are smaller than expected and pupate later than expected. The two in the middle are similar to their expected values in size, but the larger of the two pupates later, so the estimated growth rates are closer together than their masses.

The three largest Prime males grow at about the same rate. The one with the largest mass pupates later than the other two, but there is less difference in size and age at pupation among the three largest than there is between them and the worst treatment (high density, 6 day timespan, the same as the worst treatment for the females).

Prime males and females delay pupation to increase size at low food availability (the worst treatment, high density, 6 day timespan), but males pupate on day 5 or day 6 while some females delay pupation well after day 6. Males also delay pupation when food is abundant (the best treatment, low density, 3 day timespan), although they still pupate on day 5 or day 6.

Females grow larger and faster than males in all treatments. The relative difference in size of the Prime female over the Prime male and that of the Average female over the Average male could also indicate competitive interactions. S47 Table shows two new columns: the differences between the Prime female masses and the Prime male masses, and the differences between the Average female masses and the Average male masses for the interaction DxT. These differences correspond to the relative advantage of the Prime female over the Prime male and of the non-Prime females over the non-Prime males. In both cases the advantage decreases as the mass of the males and females decreases, with one exception. The Prime male in the high density, 3 day timespan treatment is relatively larger than in the high density, 6 day timespan (so the difference between the Prime female and the Prime male is smaller). Prime males in this treatment are also relatively larger than the Average males. The high density with the 3 day timespan increases the relative advantage of the Prime male over the non-Prime males, but also as compared to the females. This interaction may not affect competition among females, but it appears to affect competition among males and possibly between males and females.

The differences between the Prime female and Prime male masses, and the Average female and Average male masses in the high density, 6 day timespan treatment indicate the differences between the non-Prime males and females. Non-Prime males grow larger after the Prime males pupate (mostly on day 5) either due to competitive release, the addition of food on day 6, or both. Non- Prime females probably also grow larger on the additional food on day 6, but this doesn’t change the size distribution among females as it does with males. The relative advantage of the Prime female over the Average female increases as the food level decreases.

**Main effects**

The main effects represent the residual variance that is explained by the individual factors after the interactions have been removed. The means and standard errors for the pairs of treatments for the 4 main effects are presented in S11 Table; the explained variance (r squared) is presented in S10 Table. For the 4 mass variables and 2 age variables, the explained variance due just to the main effects is around 50% (45% to 56%). This is primarily an indication that the levels of the factors affected the growth of the larvae as hoped for during the design of the experiment. Two aspects of the main effects indicate something novel about the biology of these larvae. First, the Survival of larvae is not affected by food, density or timespan except through interactions; there is no residual significance for any of these three factors on Survival. Survival is affected by the factor aliquot, and there are no significant interactions between aliquot and any other factor. Food, density and timespan jointly affect the Survival of larvae, but aliquot only affects Survival independently of the other factors. The three interactions with food, density and timespan account for 15% of the variance across the ANOVA, and the main effect of aliquot accounts for 12%. Survival is better on 2 aliquots than on 4 aliquots. Since there is no interaction between aliquot and the other factors, the biological reason for the difference is not obvious. The second novelty within the main effects is that both female mass variables have no residual effect of density; all of the effects of density on female mass are explained by the interactions between density and the 3 factors that describe the amount and timing of food inputs. This contrasts with the large residual effect of density on age at pupation for the Prime females (32%). It also contrasts with the significant main effect of density on male mass, although the explained variance is low (1%, and less than 1%, for Prime and Average male mass, respectively). The Prime male age at pupation also has a large residual effect of density (31%).
