# Supplementary material for: Competition and growth among Aedes aegypti larvae: Effects of distributing food inputs over time
Source: PLoS One. 2020 Oct 2;15(10):e0234676. doi: 10.1371/journal.pone.0234676 (PMC7531853; doi:10.1371/journal.pone.0234676)
Supplement: S4 Text — First experiment results summary by interaction. (DOCX) [file pone.0234676.s116.docx]

**S4 Text. First experiment results summary by interaction**

**Survival across the experiment**

The experimental conditions were selected so that Survival would be high. Survival is treated as a dependent variable to investigate whether it is affected by the independent factors, and specifically whether it is affected by competition (FxD interactions). The total r squared for Survival is 0.27 (27% of the variance is explained by the treatments), but only 5% of that variance is associated with one of the competition interactions (FxDxT). Survival is lower in the most competition treatments than in the other treatments. The interaction arises because the Survival is better with the 6 day timespan than with the 3 day timespan except at the lowest food level (most competition). There are two other interactions that describe the residual effects of these factors on Survival: FxT and DxT. In the FxT interaction, after removing the effect of competition, Survival is better at the high food level with the 6 day timespan than at the other three combinations of food and timespan. Survival appears to be improved by the late addition of food at high food levels and possibly compromised by too much food in the high food, 3 day timespan treatment. Similarly, in the DxT interaction, Survival is better at the low density with the 6 day timespan than at the other three combinations of density and timespan. The interaction between density and timespan, an attribute of the food supply, may indicate that competition among the larvae affects survival. In this case, the low density, 6 day timespan appears to increase survival. This is not the treatment where competition would be lowest, so this may be related to the availability of food (see FxT above). These two residual interactions account for 10% of the variance of Survival in this experiment. Finally, Survival is affected by the main effect of the aliquot treatment. Survival is higher in the 2 aliquot treatment compared to the 4 aliquot treatment. There are no interactions involving the aliquot treatment that are significant for Survival, so the effect of aliquot on Survival is independent of food, density and timespan. The r squared for the aliquot main effect is 0.12, so the aliquot treatment explains 12% of the variance in this experiment. The independent factors appear to affect Survival differently from the way they affect the mass and age variables (below). Most importantly, Survival is not strongly linked to competition (the FxD interaction and higher order interactions including both food and density). For this set of experimental conditions Survival and competition are largely independent.

**The FxDxT interaction (R squared = 0.87)**

The interaction between food and density is expected to produce 4 different competitive environments: least competition (high food, low density); most competition (low food, high density) and two intermediate levels of competition (high food, high density, and low food, low density). The least competition test tubes receive 32 mg of food or 8 mg food/larva. The most competition test tubes receive only 16 mg of food or 2 mg food/larva. The two intermediate competition treatments receive 32 mg (for 8 larvae) or 16 mg (for 4 larvae) of food resulting in 4 mg food/larva. Mosquito larvae should grow larger, faster, and pupate earlier in the least competition treatment, followed by the intermediate competition treatments. Pupae should be smallest, grow slowest and pupate latest in the most competition treatment. Within the intermediate competition treatments, the high food, high density treatment is expected to be better for mosquito larval growth than the low food, low density treatment because the total food is greater. These four treatments are crossed with the two timespan treatments (3 days or 6 days) for a total of 8 treatment combinations. The 3 day timespan is expected to be better than the 6 day timespan because more food is offered earlier in the larval period. The interaction between the FxD treatments and the timespan treatment reveals how timespan affects the competition for food among the larvae.

Prime females dominate the competition in the test tubes. Both mass and age at pupation respond to the factors: food, density and timespan. Food and density jointly set up four different competitive environments, and timespan modifies those environments by delaying the input of food. There are two aspects of the food supply that affect competition among females; the total food in the test tube, and the food/larva. Timespan changes both total food and food/larva with each incremental addition of food. The timespan treatment changes the amount of food in the test tubes as the different inputs are added; the last input across all the treatments is on day 6, but many of the Prime females pupate before that input, so the last effective input is on day 4. The Prime female mass at pupation follows the total food at high levels of food/larva (4 mg or greater, measured at the end of day 4). At levels less than 4 mg food/larva, the Prime female mass at pupation follows the food/larva, and the total food influences the mass when the food/larva is equal across treatments. Prime females increase in mass in response to the total food in the test tubes at levels of food/larva of 4 mg or greater on day 4, but in response to the food/larva at lower levels on day 4. Average females are larger at higher levels of food/larva with total food affecting mass when the food/larva is equal across treatments. That Prime females grow in response to total food at high levels of food/larva while Average females do not suggests that the non-Prime females are diminished in equal measure to the success of the Prime female (causing the Average to follow the food/larva despite the larger size of the Prime female at higher total food levels. Competition among females is explained by total food and by food/larva. [Note that total food equals density times food/larva, so density is implicit in the two measures.] Average females do not appear to benefit from the late addition of food on day 6 or any release from competition after the pupation of the Prime female.

In contrast to the Prime female mass, the Prime female age at pupation follows the total food per test tube with the food/larva influencing the age at pupation when the total food is equal across treatments. Prime females pupate earlier in response to higher levels of total food on day 4. The Prime female mass at pupation is affected by this interaction 3 times as much as the Prime female age at pupation, and this is reflected in the estimated growth rate for the Prime female. The estimated growth rate conforms to the Prime female mass rather than the Prime female age at pupation.

No evidence of interference competition is apparent.

Prime males grow and pupate as though they are affected by the total food and food/larva in the same way as the Prime females, but the Prime males pupate at much smaller sizes and earlier, and they are more similar in size across treatments except for the most competition, 6 day timespan treatment.

The Prime male age at pupation is similar across 7 of the 8 treatments; pupation is latest in the most competition, 6 day timespan treatment, although still before the last addition of food on day 6. This is a different pattern than for the Prime females and appears unrelated to competition or food except for the most competition, 6 day timespan treatment.

Competition with females may account for some of the compression in size, especially for the Prime males in the most competition, 6 day timespan treatment, but the early, largely simultaneous pupation on day 5 suggests that the males minimize the time to pupation rather than maximizing size at pupation. However, in two cases, Prime males with greater access to food delayed pupation slightly, perhaps to grow larger (or to improve their physiological status).

The Average male mass is affected by competition and by timespan. The two least competition treatments produce the largest Average male masses. In contrast to Prime and Average females, and Prime males, the Average males grow larger in the 6 day timespan treatment than in the 3 day timespan treatment. The Average male masses are also more similar across these two treatments than the corresponding Prime male masses. The least competition treatments provide the optimal growth environment for the Prime and Average females and the Prime males. The Average male mass is larger in the 6 day timespan than the 3 day timespan, suggesting that the non-Prime males take advantage of the large final food input on day 6 and the release from competition when the Prime male pupates. It is likely that the non-Prime males grow larger on the reduced competition and increased food after day 6 in all of the 6 day timespan treatments. In the most competition, 6 day timespan treatment, the test tubes with the most extreme competition across this interaction, the non-Prime males grow larger than the Prime males on the final food input.

Another difference from the Prime and Average females, and the Prime males, is the size order of the Average males in the two intermediate competition treatments with the 3 day timespan. For the females and Prime males, the masses in the high food, high density treatment, are larger than those in the low food, low density treatment. For the Average males, the order is reversed. In both treatments all the food is added by the end of day 3, so there is no late input of food; the difference between these treatments must be due to competition. The food/larva is the same in both treatments (4 mg food/larva), and the total food is greater in the high food, high density, 3 day timespan, where the Average male mass is lower. The increased density results in increased competition which benefits the Prime male over the non-Prime males; the Prime males grow faster and larger on the higher total food and the non-Prime males are smaller than the non-Prime males in the low food, low density, 3 day timespan treatment. This suggests that males compete differently or for a different aspect of the food supply than females. Average females appear to experience more competition in the high food, high density, 3 day timespan treatment than in the low food, low density, 3 day timespan treatment (greater difference between the Prime and Average female masses), but the Average female mass is greater in the first than the second treatment, as it is for Prime females and for Prime males.

Average male mass shows the effect of these factors on the non-Prime males. The Prime male dominates competition among males, and females dominate competition in general through their larger size and retention of particles, so the non-Prime males are most affected by competition and the food levels that increase competition. Prime males appear to minimize their age at pupation, reaching a minimum size determined by environmental conditions, and pupating. Non-prime males may adopt a different life history strategy. It is possible that after missing the early pupation target, these male larvae grow to a larger size on the increased food in order to benefit from being a larger adult rather than just being another small male.

**Males versus females**

Males are affected by the food, density, and timespan treatments, but also by the competition with females. Males should grow larger and pupate earlier when food is abundant and competition with females is least. The size of females is an indication of the outcome of competition, so larger sizes indicate less competition. Females grow largest in the test tubes with the least competition and those with intermediate competition and the 3 day timespan). Males also grow largest in these test tubes. Females are smaller in the test tubes with intermediate competition and the 6 day timespan, and smallest in the test tubes with the most competition. Males are also smaller in the test tubes with intermediate competition and the 6 day timespan, and smallest in the test tubes with the most competition. However, the FxDxT treatments affect male and female growth as well as competition among females, so the effect of competition among females on the competition among males is confounded.

In addition to the well-known differences between males and females in pupal size and timing of pupation, there are differences in the way the two sexes respond to variations in the abundance of food. For both sexes the 8 treatments fall into three groups: the four treatments with the most food and less competition, the two intermediate competition treatments with the 6 day timespan, and the two most competition treatments. There are distinct gaps in the masses at pupation between these groups.

Prime females and Prime males grow largest and pupate earliest in the first group. Mass at pupation follows the total food per test tube (after day 4), with the food/larva affecting the outcome when the total food is equal across treatments. The distribution of Prime male masses is compressed relative to Prime female masses, supporting the idea that they grow to a minimum size and pupate early rather than growing to the maximum size allowed by the food supply (as females appear to do). The Average females in these 4 treatments appear to grow in response to the food/larva level with the total food affecting the outcome when food/larva is equal across treatments. This has the effect of favoring the Average females in the least competition, 6 day timespan treatment over both intermediate competition 3 day timespan treatments. The Prime female benefits from the total food and the non-Prime females are correspondingly diminished (causing the Average female mass to track the food/larva rather than the total food). The effect may be due to lower density, higher food/larva, or the final food input on day 6. The Average male mass also deviates from the pattern followed by the Prime females and Prime males. The two least competition treatments result in the largest Average male masses, but the 6 day treatment is larger than the 3 day treatment, suggesting that release from competition with the Prime male and the extra food from the last food input contribute to the growth of the non-Prime males. The two intermediate competition treatments with the 3 day timespan also switch order compared to the Prime male masses; the Average males in the low food, low density, 3 day timespan treatment grow larger than those in the high food, high density, 3 day timespan. This suggests that density increases competition for the non-Prime males more than the higher total food offsets competition.

The masses of Prime females and Prime males follow the food/larva level (after day 4) in the intermediate competition treatments with the 6 day timespan and in the most competition treatments, with the total food affecting the outcome when the food/larva level is equal across treatments. Prime female masses are more dispersed across these four treatments than across the previous four. The two intermediate competition treatments with the 6 day timespan are separated from the two most competition treatments by a large gap in size, and there are also gaps between the two intermediate competition treatments and between the two most competition treatments. Prime male masses are also more dispersed across these four treatments than across the previous four. There are gaps between the two intermediate competition treatments with the 6 day timespan, between the intermediate competition treatments and the most competition treatments, and between the two most competition treatments, but the largest gap is between the most competition treatment with the 3 day timespan and the most competition treatment with the 6 day timespan. In contrast to the Prime female masses, where the two most competition treatments are relatively similar compared to the other 6 treatments, the Prime male mass in the most competition treatment with the 6 day timespan is 0.61 mg smaller than that of the most competition treatment with the 3 day timespan. The Prime male mass in the other 7 treatments span a range of 0.59 mg, so this level of food/larva (1 mg or 1.5 mg by day 4) is much harder on the Prime male than on the Prime female.

The Average female mass also follows the food/larva level (after day 4) in these four treatments, with the total food affecting the outcome when the food/larva is equal across treatments.

The Average male mass nominally follows the food/larva level (after day 4) in these treatments, with total food affecting the outcome when the food/larva is equal across treatments. The size of the Average male indicates the outcome for the non-Prime males. The non-Prime males grow larger after the Prime male pupates (release from competition), and the final increment of food on day 6 also allows them to grow larger. In the most competition, 6 day timespan treatment, the Average male grows larger than the Prime male. In the intermediate competition treatments with the 6 day timespan, the Average male grows almost as large as the Prime male, and the Average males in the intermediate competition treatments grow larger than the Prime males in the most competition treatments. Males pupate earlier and at a smaller size that females, but the relationship between timing, size and food level is flexible for non-Prime males, so that they grow larger than the Prime males on the day 6 food input.

Females are not affected by the pupation of the Prime male. Females benefit from additional food, but only in the intermediate competition treatment with the 6 day timespan is there an anomalous difference between the Prime and Average females. The Average female mass is about 0.20 mg less than the Prime female mass in the most competition treatments, where both Prime and Average female masses are small relative to the other treatments. The difference between the Prime and Average female masses is 0.25 mg for the low food, low density treatment with the 6 day timespan, but the difference is 0.38 mg for the high food, high density treatment with the 6 day timespan (intermediate competition, and 2 mg or 3 mg food/larva). The higher total food in the high density treatment benefits the Prime female over the non-Prime females in this treatment. This may also be the case between the Prime female and the Prime male, although the effect of competition for food and the propensity for males to reach a minimum size and pupate early is difficult to separate.

FxDxT summary. Timespan interacts with competition for all seven dependent variables. The timespan treatments alter the total food and food/larva within the competition treatments and both these factors have been shown to affect the outcome of competition for males and females. The results are consistent with the prior study on competition [1]. The primary effect of timespan on competition is through changing the total food and food/larva over time. Males and females respond differently to competitive stress; Prime males pupate consistently early and respond to competition by pupating at a lower mass. Prime females require more total food (16 mg in this experiment) in order to pupate at all, and extend their larval lifespan to grow larger in response to additional food late in the lifespan in those treatments where the food was insufficient to pupate. At the same time, Prime and Average females and non-Prime males do not grow as large on the equivalent food provided late in the lifespan compared to that provided earlier. There may be physical constraints (size of body parts at the molt from 3rd instar) that cause this observation in addition to possible physiological constraints. Prime males, Average males, Prime females and Average females all do relatively well at the lower levels of competition (when the food/larva is 4 mg/larva or greater), but there are large differences across the sexes and smaller differences within the sexes when competition is more intense (when the food/larva is less than 4 mg). The Prime male is most affected by competition (most competition, 6 day timespan) pupating at the smallest size and latest across the interaction. The Average male grows larger than the Prime male in this treatment due to the release of competition after the pupation of the Prime male, and also the effect of the additional food on day 6. The Prime and Average females are also smallest in this treatment combination, but they are more similar to the most competition, 3 day timespan treatment. They are less affected by timespan when competition is intense and more affected by timespan when competition is intermediate than the males. Females may change the way that they feed (filtering versus retention of particles) as the total food and/or food/larva changes and this may explain the difference between the 3 day timespan and 6 day timespan treatments at the intermediate levels of competition.

**The FxDxAxT interaction (not significant in the MANOVA)**

The 4-way interaction would indicate that both the number of aliquots of food (2 or 4) and the timespan of delivery of those aliquots (over 3 days or over 6 days) affect the competition among the larvae in the test tubes. Aliquot and timespan both affect the growth of the larvae and separately interact with competition, but they do not jointly interact with food and density in the MANOVA. The 4-way interaction is only significant for the Average mass of females in the ANOVA.

A large input of food on day 3 effectively eliminates competition among females for a period of time, allowing the non-Prime females to grow larger relative to the non-Prime females that received equivalent food earlier in the life cycle. A similar large input of food on day 6 does not have the same effect. For females, food early in the larval period is more beneficial than food later, and multiple inputs are better than single inputs, but a large input of food on day 3 benefits the non-Prime females by eliminating competition at a critical point in the larval growth.

Female larvae outcompete male larvae, but neither the Prime male mass nor the Average male mass is affected by this interaction and the elimination of competition due to the large food input on day 3. The lack of effect of this specific food input suggests that either the food level is already so high in this treatment that males do not respond to the additional food, or that the final mass of males is already determined by the food level before day 3 (at this high food level, some non-Prime males grow larger than the Prime males in treatments at low food levels).

**The FxDxA interaction (R squared = 0.22)**

Similar to the FxDxT interaction, the interaction between food and density is expected to produce 4 different competitive environments: least competition (high food, low density); most competition (low food, high density) and two intermediate levels of competition (high food, high density, and low food, low density). The least competition test tubes receive 32 mg of food or 8 mg food/larva. The most competition test tubes receive only 16 mg of food or 2 mg food/larva. The two intermediate competition treatments receive 32 mg (for 8 larvae) or 16 mg (for 4 larvae) of food resulting in 4 mg food/larva. Mosquito larvae should grow larger, faster, and pupate earlier in the least competition treatment, followed by the intermediate competition treatments. Pupae should be smallest, grow slowest and pupate latest in the most competition treatment. The high food, high density treatment is expected to be better for mosquito larval growth than the low food, low density treatment because the total food is greater. These four treatments are crossed with the two aliquot treatments (2 aliquots or 4 aliquots) for a total of 8 treatment combinations. The interaction between the food x density treatments and the aliquot treatment reveals how the aliquot treatment affects the competition for food among the larvae. However, this contrast was only significant for the Prime female mass and the Average female mass in the ANOVAs. Nevertheless, the interaction indicates that the aliquot treatment does have an effect on competition among female larvae (in addition to the FxDxAxT interaction described above).

Multiple food inputs (the 4 aliquot treatment) result in larger sizes at pupation for Prime and Average females. Multiple food inputs appear to reduce competition among females, but have no effect on competition among males. Prime females and Average females grow larger on multiple food inputs even when the food/larva is the same, and the food level (F) is lower in the 4 aliquot treatment than in the 2 aliquot treatment (comparing the Prime and Average females in the low food, low density, 4 aliquot treatment with those in the high food, high density, 2 aliquot treatment). This anomaly, along with the pupation of some of the Prime females before the addition of the final aliquot on day 6, suggests that the total food and the food/larva on day 4 is more indicative of the appropriate food supply for these larvae. Food/larva describes the size order of the 8 treatments for both the Prime and Average females, with the total food affecting the outcome when the food/larva is the same across treatments. In the comparison between the larger females in the low food, low density, 4 aliquot treatment relative to the high food, high density, 2 aliquot treatment above, the first treatment has a higher food/larva than the second (3 mg or 4 mg food/larva in the low food, low density, 4 aliquot treatment vs. 2 mg or 4 mg food/larva in the high food, high density, 2 aliquot treatment; the two numbers reflect the two different timespan treatments that are combined in this interaction). Food/larva on day 4 is more important to the mass at pupation of females in this interaction than total food on day 4. Early food inputs are more important than later ones for females (see the exception in FxDxAxT above).

Competition among females does not seem to be affected by the different initial inputs (day 0) or by the second input (on day 1 or day 2 depending on the timespan treatment), but rather by the third input (on day 2 or day 4 depending on the timespan treatment). The third aliquot of food changes the competitive environment among females, perhaps causing them to switch from retention to active filtering. This effect is more pronounced in the test tubes with the least competition and those with the higher food level, probably because the third aliquot is larger in these treatments. In contrast, the females in the most competition treatments are smaller and the Prime females grow slowest across the interaction. The Prime females in the most competition test tubes are more similar in size (across the aliquot treatment) than the Prime females in the test tubes with the least competition and the intermediate levels of competition. The Average females in these test tubes are also closer in size (across the aliquot treatment) than the Average females in the test tubes with the least competition and the intermediate levels of competition. This suggests that at low food/larva levels females retain particles and differences in outcome are caused by differential rates of absorption rather than differential filtering ability. Large females probably have faster rates of absorption, but the difference is not as great as size differences and growth rates due to filtration ability.

**FxDxA summary**

The Prime and Average female masses are affected by competition and the aliquot treatment. The masses of males, the ages of the Prime male and female at pupation, and survival, are not affected by this interaction. Competition among females for food is different from that among males.

The 4 aliquot treatment allows females to grow larger than the 2 aliquot treatment for each level of competition. The difference between the aliquot treatments is likely the third aliquot that increases the food level during the late larval growth period. The Prime female dominates competition among females. For each level of competition the Prime female is smaller and the difference between the Prime and Average female is greater in the test tubes with the 2 aliquot treatment compared to the 4 aliquot treatment. This is possibly explained by interference competition, but the largest difference is at the intermediate level of competition, so this is more likely explained by the Prime female switching to retention from active filtering and then switching back to active filtering on the subsequent inputs of yeast particles (the third and fourth aliquots). The Average females in the 2 aliquot treatments may experience a release from competition with the addition of the last aliquot on day 3 or day 6, but they do not grow as large as the Average females in the 4 aliquot treatments (for each level of competition). Late additions of food are not of equal value to the larvae as early food inputs. This may be due to constraints created during the molt to the fourth instar larva limiting either the growth in size directly or through the smaller sizes of feeding structures.

The opposite signs of the discriminant function coefficients for these two variables in this contrast in the MANOVA appears to be due to the relative sizes of the Prime female and the Average female. As the Prime female mass decreases across treatments, the difference between the Prime female and the Average female roughly increases.

Males are affected by competition with females in the interactions FxDxT (above) and FxD (below) as well as in other sources (see [1]). That they are not affected by competition with females in this interaction suggests that the third aliquot of food that is driving this effect among females is too late to affect the outcome of competition among males (see the FxDxAxT above). One possibility is that there is already surplus food at the higher food level for the males to grow and pupate, and that at lower food levels the females monopolize the smaller incremental food input so that males do not benefit. Another possibility is that males determine their final size before day 2 or day 4 and subsequent inputs of food don’t affect that target size. A third possibility is that the molt to the third or fourth instar limits the final size of the male pupa due to some physical constraint or some aspect of the feeding apparatus.

**The FxD interaction (R squared = 0.73)**

This interaction represents residual effects of competition on the seven dependent variables (the primary effects being in the higher level interactions, FxDxT, FxDxAxT and FxDxA, described previously). It is the third largest interaction in the MANOVA, but it is only significant for four of the dependent variables in the ANOVAs: Prime male age, Average male mass, Prime female age and Average female mass. For the mass and age at pupation variables, the main effects are: higher food level increases mass and decreases age, and higher density reduces mass and increases age. This contrast is much more important for the two age variables than for the two Average mass variables. Increased competition decreases the Average masses and also increases the Prime ages at pupation.  Because this interaction affects the Average masses and not the Prime masses, competition is likely affecting the mass of the non-Prime individuals. Competition (food x density) appears to have a residual effect on the Prime male and female ages at pupation and the Average male and female masses at pupation that is independent of the aliquot or timespan treatments.

The FxD interaction shows a residual effect on competition among female larvae after accounting for the higher order interactions (FxDxT, FxDxA, FxDxAxT). The most significant effect is the delay in the Prime female age at pupation in the test tubes with the most competition. The Prime female age followed the total food in the interaction FxDxT; the effect of that interaction has been removed and the residual effect of FxD on Prime female age remains. In this interaction both the food/larva after day 4 and the total food after day 4 influence the Prime female age at pupation, but the females in the test tubes with the most competition pupate disproportionately later than the other treatments.

The mass of the Prime female is affected by the main effects of food and density, and the previously described interactions, but not by the interaction between food and density. The age of the Prime female increases disproportionately with increasing competition, so the Prime female grows to a size dependent on the main effects of food and density, but takes longer to reach that size and pupate as competition increases. There is a similar effect on the mass at pupation for the non-Prime females in the most competition treatment. Since the mass of the Prime female is not affected by the interaction, and the mass of the Average female is affected, the mass of the non-Prime females across these treatment combinations must be the reason for the interaction on the mass of the Average female. The non-Prime females in the most competition treatment are disproportionately smaller than their counterparts in the other treatments. There is also an effect on the relative advantage of Prime females over the non-Prime females in the high food, high density, intermediate competition treatment. The size of the Prime female is reduced relative to the least competition treatment, but the Average females are even smaller, indicating that the increased density/competition primarily affects the non-Prime females. This anomaly is likely due to the different competitive mechanisms (active filtering vs retention) at different levels of total food per test tube.

The FxD interaction shows a residual effect on competition among male larvae after accounting for the higher order interaction (only FxDxT). The most significant effect is the delay in the Prime male age at pupation in the test tubes with the most competition. The mass of the Prime male is affected by the main effects of food and density, and the FxDxT interaction, but not by the interaction between food and density. The age of the Prime male increases disproportionately with increasing competition, so the Prime male grows to a size dependent on the main effects of food and density, but takes longer to reach that size and pupate as competition increases. There is a similar effect on the mass at pupation for the non-Prime males in the most competition treatment. Since the mass of the Prime male is not affected by the interaction, and the mass of the Average male is affected, the mass of the non-Prime males across these treatment combinations must be the reason for this interaction for the Average male mass. The non-Prime males in the most competition treatment are disproportionately smaller than their counterparts in the other treatments, but they are larger than the Prime male in the most competition treatment. This is likely due to the competitive release of non-Prime males after the Prime male pupates. There is also an effect on the relative advantage of Prime males over the non-Prime males in the high food, high density, intermediate competition treatment. The size of the Prime male is reduced relative to the least competition treatment, but the Average males are even smaller, indicating that the increased density/competition primarily affects the non-Prime males.

This is one of the most consequential interactions for Prime female age at pupation and Prime male age at pupation. Both Prime individuals grow to a size determined by the factors, food and density, and by other interactions, but the age at pupation is delayed by increasing competition. This effect of competition is independent of the two other factors, aliquot and timespan.

This interaction also describes the competition among the non-Prime males and females (see also the FxDxAxT interaction). Non-Prime males and non-Prime females are disproportionately affected by the increase in density and competition in the high food, high density treatment (relative to the least competition treatment). The Prime individuals have the largest advantage over the Average individuals in these test tubes. The non-Prime females are also disproportionately smaller in the most competition treatment, probably reflecting a switch in feeding behavior among the females from active filtering to retention. In contrast, the non-Prime males in both the low food treatments (low food, low density and most competition) grow larger than expected. This probably reflects a release from competition after the Prime male pupates, but may also reflect the compressed size distribution of males at pupation. There may be two alternative paths for males, either to grow as fast as possible to a target size and pupate as early as possible, or to grow to a larger size in response to late additions of food and pupate as a larger adult.

**FxD summary**

For the Prime female age at pupation and the Average female mass, the test tubes with the most competition produce the worst outcomes, disproportionately worse than the other three treatments, and also worse than projected based on the main effects. Greater competitive intensity looks like a reduced food level. The effect of the interaction on Prime female growth rate is consistent with the effect on age at pupation alone. The difference between the Prime and Average female masses suggests that competition in the high food, high density test tubes is qualitatively different from that in the other treatments. In the least competition test tubes, growth appears to be optimal for this interaction. In the two treatments at the lower food level, competition appears to result in smaller sizes and a similar (smaller) size distribution. In the high food, high density treatment, the Prime female grows larger, but the non-Prime females are smaller than in the low food, low density treatment (same food/larva, but less total food). This is suggestive of different competitive mechanisms among the females in the different treatments (actively filtering particles and passing them rapidly through the gut versus retaining the particles within the gut to extract more nutrients on each transit). The active filtering at the higher total food level results in larger Prime females and smaller non-Prime females compared to retention at the same food/larva but less total food.

For the Prime male age at pupation, there are multiple asymmetries that probably cause the interaction. Prime males pupate at a disproportionately late age in the test tubes with the most competition, and this is also later than expected based on the main effects. However, the Prime males in the high food, high density test tubes pupate earliest; the main effects projected the Prime males in the least competition test tubes to pupate earliest. The effect of the interaction on the Prime male estimated growth rate differs from that of the Prime female above. The least competition treatment supports the fastest growth rate and the largest Prime male mass at pupation, but not the earliest age at pupation. These Prime males delay their pupation, perhaps in response to abundant food, and grow larger than those in the high food, high density treatment (second fastest growth rate and earliest age at pupation). The Prime males in the low food, low density treatment also delay their pupation, but they are smaller than the high food, high density Prime males, and also have a slower growth rate. The Prime males in the most competition treatment are smallest, latest to pupate and have the slowest growth rate. The Prime males pupate in a small window of time, but delay pupation in response to abundant food, and to competition at lower food levels (retention by females). Prime males pupate earliest in those test tubes where the competitive mechanism among females is predominately active filtering. Prime males respond to food and density (and competition with females) differently than Prime females. The Prime female pupates earliest in the test tubes with the least competition; the Prime male delays pupation in response to abundant food in those test tubes. The Prime females’ estimated growth rates reflect their ages at pupation; the Prime males’ estimated growth rates reflect their masses rather than their ages at pupation. However, the Prime males pupate in a much tighter window of time than the Prime females, so there is more variability in the masses than in the ages at pupation. Prime males pupate earliest in the test tubes where the Prime females appear to dominate the non-Prime females to the greatest degree, the high food, high density treatment. The more intense competition among females in these test tubes compared to the least competition test tubes appears to cause the Prime male to pupate sooner and at a smaller size.

The Average male mass resembles the Average female mass; the test tubes with the most competition produce the worst outcomes, disproportionately worse than the other three treatments, and also worse than projected based on the main effects. Because the Prime male mass is not significant for this interaction, the affected individuals are the non-Prime males (paralleling the situation for the Prime and Average females above). There are two quantitative differences in the relationship between the Prime male mass and the Average male mass that are different from the females. First, the Average male mass is greater than the Prime male mass in the most competition treatment; at least one non-Prime male grows larger than the Prime male after the Prime male pupates. Second, the Prime male mass is larger in the high food, high density treatment than in the low food, low density treatment (same food/larva, but more total food), but the Average male mass is larger in the low food, low density treatment. In the high food, high density test tubes there is the same amount of food as in the least competition ones, but 4 more larvae. Competition among the larvae is more intense and this appears as a reduction in the amount of food. Because there is a high food level the nature of competition among the females is active filtering and rapid passing of the particles through the gut, the distribution of sizes is broad and the relative advantage of the Prime male over the non-Prime males is large. In the low food, low density test tubes, the total amount of food is half of that in the high food treatments, but the lower density means that the food/larva is the same across the two intermediate competition treatments (4 mg/larva). In this case, the lower number of particles causes the females to switch to retaining particles in their guts, further reducing the number of particles available for the males and compressing the size distribution. The Prime male is reduced in size and the Average male mass is similar to that in the high food, high density treatment, so the non-Prime males do better in the low food, low density treatment than in the high food, high density one (both relative to the Prime male and absolutely against the non-Prime males). The Average male mass is larger than expected at both low food treatments. The non-Prime males grow larger after the Prime male pupates in the treatments where the females are retaining food because of the low number of particles. The Prime male pupates, releasing food particles and reducing competition; this benefits the non-Prime males and allows them to grow larger. Timespan and aliquot are not involved in this interaction, so this effect (competitive release) is not affected by those factors.

The Prime males and females pupate in response to different cues in the test tubes. Prime females respond to food, density and competition (the interaction between food and density). Prime females grow best in the test tubes with the least competition and switch from active filtering to retention at the lower food level (two treatments). Prime males respond to the main factors and the interaction as well, but also to the nature of the competition among females. Prime males pupate earliest in the test tubes with the high food, high density treatment where the females are actively filtering, but extend their larval period in the test tubes where the females appear to have the most food. The males also extend the larval period in the test tubes at the two low food treatments where the females are retaining particles.

The Average male and female masses represent the effect of this interaction on the non-Prime males and females. The Prime females dominate the competition in these test tubes; they are larger (better at filtering) and switch from active filtering to retaining particles in their guts in response to lower food levels. In the test tubes with the least competition, both Prime and Average females grow largest. In the high food, high density test tubes, the difference between the Prime and Average females is greatest, indicating that the active filtering competitive mechanism creates a broad size distribution. The non-Prime females do relatively and absolutely less well compared to those in the least competition test tubes and compared to those in the low food, low density test tubes. The higher density compared to the least competition test tubes increases competition for particles. This reduces the size of the Prime female, but affects the non-Prime females to a larger degree. The different competitive mechanism at the lower food level (retention) also reduces the size of the Prime female, but does not reduce the size of the non-Prime females as much as in the high food, high density test tubes. There is a similar effect on the non-Prime males. Males compete more intensely with each other and are affected by the competition with females (but females are unaffected by competition with males [1]). In addition to the larger size distribution of males in the high food, high density test tubes, the non-Prime males in the two low food level treatments grow larger than expected. They experience a release from competition after the Prime male pupates. The difference between the Average female mass and the Average male mass is smallest in the most competition treatment where the non-Prime males grow larger than the Prime male. The difference is also small in the low food, low density treatment where the non-Prime males grow larger than the non-Prime males in the high food, high density treatment. At the high food levels, both the Average masses increase in size, but the Average female mass is increasingly larger than the Average male mass. The size advantage of larger females actively filtering and outcompeting smaller females also means that they are outcompeting the males.

**Interactions involving competition (FxD)**

Four interactions describe competition among mosquito larvae in this experiment: FxDxT; FxDxAxT; FxDxA; and FxD. FxDxT, FxDxA, and FxD are all significant in the MANOVA; the 4-way interaction, FxDxAxT, is not significant in the MANOVA. FxDxT is significant in the ANOVAs for each of the seven dependent variables. FxDxAxT is significant only for the ANOVA for Average female mass. FxDxA is significant for the Prime female mass and the Average female mass. FxD is significant for Prime male and female ages at pupation and for Average male and female masses. Together these interactions describe a complex relationship between the factors: food, density, aliquot and timespan; and the dependent variables that measure the growth and competition among male and female mosquito larvae. These interactions cross food (total food per test tube) with density (4 or 8 larvae per test tube) to compare the outcomes across different competitive environments.

The four mass variables decrease with increasing competition and the two age variables increase with increasing competition, so they are frequently negatively correlated in the MANOVA. The Prime female dominates competition in the test tubes and affects the size of the Average females and the size of the males as well as the age at pupation of the Prime male. The Prime female mass is significant in the FxDxT and FxDxA interactions (in the ANOVAs). The Prime female age is significant in the FxDxT and FxD interactions. The Average female mass is significant in all four interactions. When the Average female mass is significantly affected by a contrast, but the Prime female mass is not affected, the presumption is that the interaction reflects competition by the non-Prime females (FxDxAxT and FxD).

The Prime male dominates competition among the male larvae. The Prime male mass is significant only in the FxDxT interaction. The Prime male age is significant in the FxDxT and FxD interactions. The Average male mass is significant in the FxDxT and FxD interactions as well. When the Average male mass is significantly affected by a contrast, but the Prime male mass is not affected, the presumption is that the interaction reflects competition by the non-Prime males (FxD). This is also the case when the Average male mass is larger than the Prime male mass (the non-Prime males grow larger than the Prime male).

Prime females: The interaction FxDxT shows that the Prime female mass at pupation follows the total food level (on day 4 of the experiment) when the food/larva is 4 mg or greater, but follows the food/larva level at lower levels. This is probably related to the mechanism of feeding (active filtering versus retention) associated with the abundance or scarcity of food particles. Late additions of food do not have the same value as equal earlier inputs. The Prime female age at pupation follows the total food level across all treatments. In the interaction FxDxA, the Prime female mass follows the food/larva (on day 4 of the experiment) across all treatments (only the two least competition treatments have food/larva levels that are all greater than 4 mg, and the total food levels are in the same order as the food/larva for these two treatments). Late additions of food do not have the same value as equal earlier inputs in this interaction either. The Prime female age at pupation is not affected by the FxDxA interaction. In the FxDxA interaction, the third aliquot of food (only in the 4 aliquot treatment) appears to positively affect the mass of the Prime females. The Prime female mass is not affected by the FxD interaction, but the Prime female age is affected. Prime females pupate at masses determined by other factors, but competition (FxD) affects how long they take to pupate. For the Prime female, the timespan treatment reveals that competition changes in response to the highest levels of total food at high levels of food/larva, but that the timing of pupation is related to the total food. The FxDxA interaction reveals that multiple inputs of food are beneficial and that the third aliquot results in additional growth, probably by providing more food at a critical time in the larval lifespan. The FxD interaction shows that competition affects the timing of pupation while other factors (and other interactions) affect the mass at pupation. The mass at pupation of the Prime female is determined by one set of environmental parameters, and the age at pupation is determined by another set, which partially overlaps the first set. Mass and age at pupation are partially independent, but both respond to some of the same environmental cues (e.g. total food). When these cues change during the larval life, early changes have a greater effect than later changes. This could be because of biochemical and physiological characteristics of the larvae and/or physical characteristics such as the size of the head capsule or the feeding apparatus.

Average females: The interaction FxDxT shows that the Average female mass at pupation follows the food/larva level (on day 4) across all treatments. Average females do not appear to benefit from the late addition of food on day 6 or any release from competition after the pupation of the Prime female. The Prime female controls the food supply and benefits exclusively from the high total food, while the non-Prime females grow according to the food/larva level. The interaction FxDxAxT reveals that a large input of food on day 3 effectively eliminates competition among females for a period of time, allowing the non-Prime females to grow larger relative to non-Prime females that received equivalent food earlier in the life cycle. The specific timing of this food input makes food particles available when the Prime female would be switching from active filtering to retention, and allows the non-Prime females to grow larger (increasing the Average female mass). The FxDxA interaction reveals that multiple food inputs result in larger masses for Average females. Food/larva (on day 4) describes the size order of the mass at pupation of the Average females. Early food inputs are more important than later ones for the Average females (but see FxDxAxT above). The FxD interaction reveals that the mass of the non-Prime female is affected by competition after the higher order interactions are removed; the residual effect of food and density jointly on the mass of the non-Prime female is independent of the two other factors: aliquot and timespan.

The Prime female dominates competition in the test tube and solely benefits from the high total food level. The non-Prime females grow in response to the food/larva, although they can benefit from a large input of food (on day 3 in this experiment) that coincides with the switch from active filtering to retention by the Prime female as the relative abundance of particles decreases. Food is probably unlimited for 1^st^ instar larvae and similarly abundant for 2^nd^ instar larvae. As the larvae molt from the 2^nd^ instar to the 3^rd^ instar, their demand for particles increases and their ability to filter particles increases, reducing the availability of particles. During the 3^rd^ or 4^th^ instar, the demand for particles exceeds the availability and the females switch from actively filtering particles and passing them rapidly through their guts to retaining the particles to extract more nutrients. This is further exacerbated because the quality of the particles decreases over time. An input of food in the 3^rd^ instar would offset the relative decrease in particles and allow the females to continue to actively filter. Multiple such inputs would result in larger females by reducing competition as well as increasing food availability. The timing of a food addition in the third instar may offset multiple earlier inputs (of equal food) because the non-Prime females escape competition at the time when the Prime female would normally switch to retention, reducing the growth rate of all the females and reducing their eventual mass at pupation. Individual females grow as fast as possible in response to environmental conditions. As 1^st^ instars, food may be unlimited, but chance and the initial size of the larva (or egg size, or some other uncontrolled factor in the experiment) determine how fast the larva grows. At some point, this larva molts to the 2^nd^ instar. The larva may delay molting in order to grow larger because food is available, but there is also an advantage to molting in order to use the larger feeding apparatus of the 2^nd^ instar. This trade off between growing larger and molting should also be true for the 3^rd^ and 4^th^ instar molts. Other similar trade offs may be important for the timing of pupation.

In this experiment, the total food and food/larva in the test tubes change depending on the aliquot and timespan treatments. Both total food and food/larva affect competition among females; timespan is more important than aliquot, and their effects are independent (with the exception of non-Prime females in the FxDxAxT interaction). It appears that the environmental parameters on day 4 of the larval lifespan are the most important to determining the final size and age at pupation of females, although at lower total food levels females do not pupate until there is 16 mg total food in their test tubes (after the final food input for the 6 day timespan treatment).

Prime males: Males are affected by competition with females [1]. The Prime male benefits from reduced competition with females (at higher food levels, for instance) usually at the expense of non-Prime males. This suggests that males compete more intensely among themselves than with females. The Prime male mass at pupation is affected by total food and food/larva in the same way as the Prime females (on day 4 of the larval period), but Prime males are smaller and more similar in size to each other at pupation than are the Prime females. Competition with females may account for some of the compression of the size distribution, but the early, largely simultaneous pupation on day 5 suggests that Prime males minimize the time to pupation rather than maximizing size at pupation. Prime male age at pupation is affected by food and competition. In two treatments, Prime males pupate slightly after day 5 apparently because food is abundant (3 day timespan); both of these pupate at larger masses than their 6 day timespan peers. In the two most competition treatments, Prime males pupate slightly after day 5, with the 6 day timespan treatment pupating mostly on day 6, but still before the addition of food at the end of day 6. The mass at pupation of Prime males is determined by food, but can be modified by delaying pupation to take advantage of abundant food, or to continue growing because food is scarce. The aliquot treatment does not interact with competition for Prime males; there is no effect of dividing the total food into 2 or 4 aliquots. In the FxD interaction, the mass of the Prime male is determined by other factors, but the age at pupation is affected by the residual competition after the higher order interactions have been removed. Prime male age at pupation is earliest with the least competition treatment, a little later in the intermediate competition treatments and disproportionately delayed in the most competition treatment.

Average males: The Average male mass responds to food level, density, competition and timespan, but is not affected by the interactions between competition (FxD) and aliquot. The Average male mass deviates from the pattern shown by the Prime male and female mass variables and by the Average female mass. Non-Prime males experience a release from competition after the Prime male pupates, and in the 6 day timespan treatments, they receive additional food and appear to grow larger as a result. Prime males outcompete non-Prime males in the high food, high density, 3 day timespan resulting in larger Prime males and smaller non-Prime males compared to the equivalent food/larva treatment, low food, low density, 3 day timespan. Despite the same food/larva (4 mg food/larva) the Prime males are smaller and the Average male mass is larger at the low density than at the high density. There is also a residual effect of competition on the size of non-Prime males (in the FxD interaction). Non-Prime males decrease in size in the treatments with increased competition, but they are disproportionately small in the most competition treatment (FxD). Despite the small size of the non-Prime males, at least one of them grows larger than the Prime male so that the Average male mass is larger than the Prime male mass in this interaction. This is similar to the observations in the FxDxT interaction. There is no timespan treatment here, so the increase in size of the non-Prime male over the Prime males must be due to the release from competition rather than additional food. There is also evidence that competition among males is more intense in the high food, high density treatment than in the low food, low density treatment (with the same food/larva). The Prime male is larger, the Average male mass is smaller and the difference between them is larger in the high food, high density test tubes compared to the low food, low density test tubes.

In the FxDxT and FxD interactions, Prime and Average males are largest in the least competition treatments, and smallest in the most competition treatments. The high density intermediate competition treatments (4 mg food/larva) produce larger Prime males and smaller Average males than the low density intermediate competition treatments (also 4 mg food/larva). The largest difference between Prime and Average males is in the high density intermediate competition treatments. Average males grow larger than the Prime males in the most competition treatment; this represents the incremental difference in the size of the non-Prime males. In the FxD interaction, the Average male is 0.01 mg larger than the Prime male. In the FxDxT interaction, the Average male is 0.14 mg larger. The FxD increment represents only the release of competition after the Prime male pupates, while the FxDxT increment represents both the release of competition and the additional food at the end of day 6 in the 6 day timespan treatment. The effect of the release from competition with the Prime male is much smaller than the effect of the additional food after day 6.

In the FxDxT interaction, the total food after day 4 explains the size order of Prime males when the food/larva is 4 mg or above. The food/larva after day 4 explains the size order of Prime males below 4 mg food/larva. The outcome of competition for the Prime males is directly related to the availability of food after day 4. This is not true of the Average male mass. The outcome of competition among males in the 3 day timespan treatments of the FxDxT interaction resembles that of the FxD interaction. The outcomes for males in the 6 day timespan treatments are divergent. Prime males are smaller in the 6 day timespan for each treatment. The non-Prime males grow larger in the 6 day timespan treatment than in the 3 day timespan treatment in the least competition test tubes. The non-Prime males grow larger than the Prime males in the most competition test tubes. Both are due to the release from competition and the additional food after day 6. In the intermediate competition treatments (4 mg food/larva), the effect of the 6 day timespan is to reduce the size of both Prime and Average males, and to reduce the difference between them at the high density, but to increase that difference at the low density. This is different from the effect of the 6 day timespan on Prime and Average females in the intermediate competition treatments.

Competition among males is affected by females, but males appear to be competing more intensely among themselves than directly with females. Males grow to a size determined by various environmental factors and pupate earlier and at a smaller size than females. However, males that fail to pupate early may delay pupation and grow larger, suggesting that adult longevity and other benefits of larger size may be viable alternatives to early emergence.

**Interactions not involving FxD competition**

The previous four interactions describe competition (FxD) and the way that the factors timespan and aliquot affect competition (FxDxT, FxDxA, and FxDxAxT). Timespan and aliquot change the amount of food in the test tubes over time and this influences the nature and intensity of the competition among the larvae in those test tubes. The remaining 7 interactions describe the separate effects of food and density on timespan and aliquot (FxT, DxT, FxA, DxA), the interaction between timespan and aliquot (AxT) and the separate effects of food and density on that interaction (FxAxT, DxAxT).

**FxAxT, then FxA, FxT and AxT**

These four interactions only involve factors that affect the food supply. They describe the growth of larvae, not competition between larvae. The larvae are competing with each other in the test tubes, but without density as a factor in the interaction, the effect of competition is averaged out across the densities.

**The FxAxT interaction (not significant in the MANOVA)**

The interaction of food, aliquot and timespan is significant only for the Prime female mass and the Average female mass in the ANOVAs, and not significant at all in the MANOVA. However, the 2-way interactions between these three variables are significant in the MANOVA. It is necessary to account for the 3-way interaction before considering the 2-way ones. This interaction is between three characteristics of the food supply. No density is involved, so the interaction describes growth rather than competition. The behavior of the female larvae may still be affected, switching from active filtering to retaining particles, but the end points, mass and age at pupation indicate the nature of growth on different characteristics of the food, not different competitive environments.

**FxAxT summary**

This 3-way interaction describes the effect of three attributes of the food supply on the growth of females. The food level is the most important factor. Within each food level there is an interaction between aliquot and timespan; aliquot has little effect at the 3 day timespan, but a large effect at the 6 day timespan, and a larger effect at the high food level than at the low food level. The Prime female masses are arranged in 3 groups with large gaps between each group. The largest Prime females are in the high food treatment; the two largest receive 32 mg of food by day 3 and the third largest receives 24 mg of food by day 4. The next group receives 16 mg of food on day 0 or by day 3. The third group doesn’t receive 16 mg of food until day 6. Prime females require 16 mg of food to pupate, so the last group delays pupation compared to the first two groups. The total food is the most important factor, but the early delivery of food (3 day timespan and 4 aliquots, jointly) increases the size of the Prime females.

At the highest food level the Prime females in the 4 aliquot, 3 day timespan treatment grow largest, but those in the 2 aliquot, 3 day timespan treatment grow fastest and pupate earliest. The large input of food on day 3 appears to accelerate the growth of females. The timing of the input and/or the relative abundance of food particles affects the triggers for pupation in the Prime female at high food levels.

Within the middle group of Prime females, the ones that receive 16 mg of food on day 0 grow largest and fastest, followed by the ones in the 4 aliquot treatment (4 mg of food per day on day 0 - day 3). The Prime females in the 2 aliquot treatment (8 mg of food on day 0 and day 3) are smaller and grow more slowly than the other two. Early delivery of food (1 large input, then 4 aliquots, then 2 aliquots) increases the size of Prime females and their growth rates at moderate food levels.

The estimated growth rates of the Prime females in middle group correspond to their masses. There is no acceleration due to the large input on day 3 similar to that observed at the high food level.

At the low food level and the 6 day timespan, the Prime females do not have enough food to pupate until after the last aliquot on day 6. They grow larger and faster on the 4 aliquot treatment than the 2 aliquot treatment. Despite equal food and a longer larval period, they do not grow as large as the Prime females in the 3 day timespan treatments. Food early in the larval period is more important than later additions for both size and growth rate.

The Average female mass is similar to the Prime female mass, but food level is less important and the interaction between aliquot and timespan is more important. The Average female mass is largest in 3 of the 4 high food level treatments; the Average females in the high food, 2 aliquot, 6 day timespan treatment are smaller than the largest Average females in the low food treatments. Within each food level there is an interaction between aliquot and timespan; aliquot has little effect at the 3 day timespan, but a large effect at the 6 day timespan, and a larger effect at the high food level than at the low food level. The Average females in the 6 day timespan treatments are all smaller than those in the corresponding 3 day timespan treatments across the interaction. The Average females in the 6 day timespan treatments are also relatively smaller than the Prime females in these treatments (again compared to the corresponding 3 day timespan treatments). The additional food in the final aliquot on day 6 does not result in larger non-Prime females; the distribution of sizes among females is determined earlier in the larval growth period and doesn’t change despite the additional food on day 6.

The Average female masses are also arranged in 3 groups with large gaps between each group. The largest Average females are in the high food treatment; the two largest receive 32 mg of food by day 3 and the third largest receives 24 mg of food by day 4. The next group receives 16 mg of food on day 0 or by day 3. The third group doesn’t receive 16 mg of food until day 6. The total food is the most important factor, but the early delivery of food (3 day timespan and 4 aliquots) increases the size of the Average females as it does for the Prime females.

At the highest food level the Average female mass in the 4 aliquot, 3 day timespan is the largest and the closest to that of the Prime female mass. In this treatment with abundant food, the non-Prime females do better than in any other treatment. There is a bigger difference between the Average female mass in this treatment and the next largest Average female, than between the corresponding Prime females, so the non-Prime females do better on the 4 aliquot treatment than on the 2 aliquot treatment (at the high food level and 3 day timespan).

There is a larger gap in size between the 3 largest Average females and the middle group of Average females (compared to the same gap for the Prime females). This due to the smaller size of the Average females in the high food, 2 aliquot, 6 day timespan relative to the Prime female in that treatment and also relative to the group of largest Average females. The three treatments in this middle group all receive 16 mg of food either on day 0 or by day 3. The Prime females that receive 16 mg of food on day 0 grow larger than the other two, but the Average females in this treatment do not. The Average females in the 4 aliquot treatment do better than the other two, so again the non-Prime females benefit from the 4 aliquot treatment. There is also a smaller difference between the Prime female mass and the Average female mass in the 4 aliquot treatment, compared to the other two in this middle group. However, the difference between the largest and smallest of these three Average female masses in the middle group is smaller than the difference for the middle group of Prime females. This may indicate that the food supply attributes, aliquot and timespan, are less important to the non-Prime females than to the Prime females in this middle range of food.

The gap between the middle group of Average female masses and the two smallest Average female masses is about the same as that for the corresponding Prime females. The Average females in the 4 aliquot treatment do better than the ones in the 2 aliquot treatment (both in test tubes with low food, and 6 day timespan). The difference between the Prime and Average female masses also indicates that the non-Prime females grow relatively larger in the 4 aliquot treatment. The Prime and Average female masses in the low food, 2 aliquot, 6 day timespan treatment are the smallest across the interaction, and the difference between the Prime and Average female masses is the second largest, so the non-Prime females face the worst food environment in this treatment.

Large initial inputs of food allow the Prime female to grow faster and to dominate the food supply. Smaller, regular inputs (4 aliquot treatment) allow the non-Prime females to grow larger than equivalent amounts of food in fewer, larger inputs. The food level is the most important factor for the Prime females, followed by the interaction between aliquot and timespan. The interaction between aliquot and timespan is relatively more important for the non-Prime females. The final aliquot in the 6 day timespan appears essential to Prime females at the lowest food level, but does not appear to benefit the Prime females in the other treatments (because they pupate before the addition of the food), or the non-Prime females (perhaps because the size distribution is fixed by the molt into the fourth instar).

**The FxA interaction (R squared = 0.25)**

The FxA interaction indicates whether the apparent amount of food changes depending on the number of aliquots it is divided into. There is no density involved, so this examines the effect of food and aliquot on growth not competition. This interaction is significant for the Prime male mass and age at pupation and the Average male mass at pupation in the ANOVAs. The three variables: Prime male mass and age, and Average male mass, are not significant in the FxDxAxT, FxDxA, or FxAxT interactions described earlier. This is the highest order interaction between food and aliquot for males. Survival, and the female mass and age variables are not significant in the ANOVAs for this interaction.

FxA summary. This interaction shows that the growth of males is affected by the interaction of the amount of food and the number of aliquots it is divided into, separately from competition for food (food x density) and timespan. Both Prime and non-Prime males grow better on 4 aliquots than 2 aliquots. The Prime males pupate before the 6th day, but timespan is not a factor in this interaction; the difference between the 3 day timespan and the 6 day timespan is not important. It appears to be the food delivered in the day 2 to day 4 period (the third aliquot) that causes all the larvae to grow larger in the 4 aliquot treatment (the main effect of aliquot). This interaction between food and aliquot arises because males grow larger than expected and take longer in the low food, 4 aliquot treatment. Males are growing rapidly and filtering particles, so the added particles accelerate their growth, and this has a larger effect at low food levels than at high food levels. Prime males defer pupation slightly and extend their growth in response to the third aliquot of food and grow larger than Prime males at the same food level and the 2 aliquot treatment. Although the Prime and Average males are smaller in the low food treatment, the third aliquot has a larger impact on growth at the low food level compared to the high food level.

The low food treatment causes the females to switch from active filtering to retaining particles in their guts, reducing the available particles. This reduces the size and size distribution among the females, and also reduces the size and the size distribution among males. Males are smaller than projected at the low food level, especially in the 2 aliquot treatment, but the size distribution of the males is also compressed. This can be explained by exponential growth processes. At the low food level, Prime males have less of an advantage over the non-Prime males than at the high food level. Prime and Average male masses in the low food, 4 aliquot treatment grow larger than in the 2 aliquot treatment, and are almost as large as their projected values. This is due to the third aliquot of food that is delivered in the middle of the larval growth period (day 2 or day 4, depending on timespan treatment). Prime and non-Prime males grow larger but the relative advantage of the Prime female over the Prime male is smaller, so the Prime male benefits more from this third aliquot food addition than the non-Prime males. Males appear to actively filter at all food levels, unlike females, which appear to switch from active filtering to retaining particles in their guts. In this situation, the males respond sooner to the third aliquot and the Prime male grows larger relative to the Prime female. The Prime male also grows larger relative to the non-Prime males due to exponential growth processes and the initial size distribution before the third aliquot. The Prime males extend their larval period to grow larger on the extra food, and pupate latest in this treatment (across this interaction).

The non-Prime male masses are in the same order as the Prime male masses, so all males appear to benefit from the third aliquot, but the Average male mass in the low food, 2 aliquot treatment is the same as the Prime male mass. The non-Prime males in this treatment grow as large as the Prime males. They don’t grow as large as the non-Prime males in the low food, 4 aliquot treatment; the third aliquot delivers more food earlier in the larval lifespan. The non-Prime males in the low food, 2 aliquot treatment must take advantage of the additional food in the final aliquot (on day 3 or day 6) to grow as large as the Prime male. This suggests that there is a minimum mass at pupation for males that is determined by environmental conditions before day 3. Male larvae grow until they reach that minimum, or grow larger than the minimum, even delaying pupation, in response to greater availability of food.

The main effect of the 4 aliquot treatment increases the size of all larvae compared to the 2 aliquot treatment, but for males at the low food level, it provides a disproportionate benefit. There are two different anomalies: the Prime male mass in the low food, 4 aliquot treatment is disproportionately large relative to both the Average male and to the Prime female; and the Average male mass in the low food, 2 aliquot treatment is the same as the Prime male mass in that treatment. The Prime male grows larger on the third aliquot at the low food level in relation to the Prime female. The non-Prime males grow to the same size as the Prime males at the lowest food level, suggesting a minimum mass at pupation for males depending on the availability of food before day 3.

**The FxT interaction (R squared = 0.61)**

The interaction between food and timespan shows the residual effect after the higher order interactions have been explained (FxDxT for all variables, FxAxT for Prime and Average female mass, and FxDxAxT for Average female mass). The ANOVAs are significant for all the variables except for Prime female age. This interaction is about 3 times more important for males than for females. The main effects are: higher food is associated with better Survival, larger mass, and earlier pupation; 3 day timespan is associated with lower Survival, larger mass and earlier pupation. There is no density in this interaction, so the contrast describes the effect of food and timespan on the growth of larvae.

FxT summary. Males and females both grow according to the food level in the test tubes, but this food level is modified by the timespan treatment, and significantly affects the growth of both sexes. Females do not pupate until 16 mg have been added to the test tubes (at the end of the timespan for the low food level); males do pupate on less food. Females grow larger in stepwise increments as the amount of food increases; males also grow larger, but the increments are smaller and less regular. Both females and males are larger on the high food, 3 day timespan than projected by the main effects, and smaller than projected in the low food, 6 day timespan. The range of sizes of the Average females is slightly larger than that of the Prime females. The range of sizes of the Average males is compressed relative to that of the Prime males. Females may change their feeding behavior in response to the food inputs across the timespan, producing different size distributions. Most Prime females pupate on day 6; the Prime females in the low food, 6 day timespan pupate 2.28 days later. Most Prime males pupate on day 5; none appear to pupate late enough to take advantage of the day 6 food input. Non-Prime males do grow larger after the Prime males pupate; in some cases they grow larger than the Prime males. Females also grow larger on the late addition of food on day 6, and this may increase the size distribution suggesting that the females switch from retention to active filtering when the last aliquot of food is added.

In this interaction, females grow according to the total food in the test tubes; total food is affected by the food level treatment and the timespan treatment. The early instars experience unlimited food for the first few instars, but later instars are limited by food in some treatments. The total food after day 4 is the relevant measure of the amount of food during the late larval period. Prime and Average females are much larger than expected at the high food level and 3 day timespan treatment. There is about 0.20 mg difference in size between the Prime and Average females in each treatment. Prime and Average females are about 0.5 mg smaller at the high food and 6 day timespan treatment (half or three quarters of the total food, depending on aliquot treatment). They are about another 0.50 mg smaller at low food and the 3 day timespan treatment (half the total food of the high food treatment). They are another 0.5 mg smaller at the low food and 6 day timespan treatment. There is not enough food for the Prime female to pupate until they receive the full 16 mg total food on day 6, so they extend their larval life by two days. The residual effect of this interaction on females (after removing the effects of the FxDxT interaction, the FxAxT interaction, and the FxDxAxT interaction) is the direct, almost linear, relationship between the amount of food after day 4 and the mass at pupation.

This interaction affects male mass three to four times as much as female mass, and it also affects the Prime male age at pupation. The residual effect on males (after removing the effects of the FxDxT interaction) differs from that for the females. Male mass at pupation is also directly related to the total food after day 4, but the three treatments corresponding to the most food are closer in size for Prime males, and for Average males, while the treatment with the least total food is much smaller for both Prime and Average males. The three treatments with the most food are all larger than expected and the treatment with the least food is smaller than expected for both Prime males and Average males. Prime males pupate before the final input on day 6, but they delay pupation at the highest total food and at the two lower total food levels. They grow longer and larger when food is most available, and also grow longer when food is least available, but they still pupate on day 5 or day 6. Prime males decrease in size only about 0.20 mg with each decrease in total food (compared to 0.50 mg for Prime females). Prime males at the lowest total food are disproportionately small (0.50 mg smaller than the next largest), nevertheless, they are able to pupate before the final addition of food on day 6. This suggests that the available food for the males is much lower in this treatment, probably due to retention by females at low food levels.

The Average male mass at pupation is larger than the Prime male mass in the low food, 6 day timespan treatment, where both masses are lowest across the interaction. There is no competition in this interaction, so the difference represents the incremental effect of the food after day 6 on the growth of the non-Prime males at the lowest total food.

This interaction describes the growth of larvae rather than competition. Males actively filter particles throughout their larval period. Females in the low food levels switch to retention at some point as the number and quality of particles decreases relatively to demand. The Prime male grows at the expense of the non-Prime males and pupates in response to unknown triggers that may include size, physiological status, age, and environmental cues (availability of food particles).

The females and the non-Prime males experience a benefit when the final input of food is added on day 6 and grow larger. Some non-Prime males grow to be larger than the Prime male on the extra food. The larger females grow even larger on the extra food and perhaps switch from retention to actively filtering, but the incremental food is less valuable than the same amount of food earlier in the larval period (for instance, the 3 day timespan). None of the larvae in the low food, 6 day timespan treatment reach the size of corresponding larvae in the low food, 3 day timespan treatment despite the extra food and longer larval periods.

The estimated growth rates of the Prime females are greater than those for the Prime males in all treatments. For females, the estimated growth rates reflect similar Prime female sizes and divergent ages at pupation (affected by other interactions and main effects, but not by this interaction). For males, the estimated growth rates reflect similar ages at pupation, but divergent sizes. The estimated growth rates are in the same order as the total food for both males and females. The largest difference between the rates for females and those for males is at the highest total food. The differences decrease as the size of the pupae and the total food decrease, suggesting that this is due to exponential growth processes.

Males and females respond to food differently after the effects of competition and aliquot have been removed by the higher order interactions. Females grow linearly in response to the total food after day 4. They required 16 mg of food in order to pupate and delay pupation until after day 6 in the low food, 6 day timespan treatment. Males also grow larger in response to more total food, and they delay pupation both when food is available and when it is limiting, but pupate mostly on day 5. Males in the low food, 6 day timespan treatment are much smaller than the other males, but the non-Prime males grow larger than the Prime males on the final addition of food on day 6. This appears to be due to exponential growth processes in response to the total food, but modified by different pupation triggers between the two sexes.

**The AxT interaction (R squared = 0.51)**

This interaction between aliquot and timespan represents the residual effect of these factors after the higher level interactions have been resolved (FxAxT for Prime female mass and Average female mass; FxDxAxT for Average female mass; DxAxT for Average male mass and Prime female mass, below). This interaction is significant in the ANOVA for the four mass variables and the Prime male age at pupation. The main effects of aliquot and timespan are: for the mass variables, 4 aliquots are better than 2 aliquots and the 3 day timespan is better than the 6 day timespan; for the Prime male age at pupation, 2 aliquots are better (earlier) than 4 aliquots and the 3 day timespan is better than the 6 day timespan.

Prime and Average females grow largest on the 3 day timespan treatments with only a small difference between the 2 aliquot and 4 aliquot treatments. Prime and Average females in the 4 aliquot, 3 day timespan treatment are largest; those in the 2 aliquot, 3 day timespan treatment are a little smaller, but still larger than their expected values. Both Prime and Average females do much worse than expected at the 2 aliquot, 6 day timespan treatment. The obvious reason for this is that there is less food in these test tubes than in any of the others; the first aliquot of food is delivered on day 0 and no more food is delivered until after pupae are collected on day 6. The Prime females pupate almost 2 days after the final aliquot is delivered, but do not grow as large as any of the Prime females in other treatments. This is an effect of the distribution of food in time independent of the food level, density, or competition (food x density) factors.

The difference between the Prime female mass and the Average female mass indicates changes in size distribution of the females and the relative advantage that the Prime female has over the non-Prime females. The difference between the mass of the Prime female and that of the Average female is smallest in the test tubes where the females grow largest (4 aliquots, 3 day timespan) and greatest in the test tubes where the females are smallest (2 aliquots, 6 day timespan). This is the opposite of the effect due to exponential growth processes. The large size of the Prime female corresponds to the fastest growth rate and the most abundant food (more aliquots, delivered in 3 days). The small difference between the Prime and Average female mass (0.16 mg) indicates that all the females benefit from this food delivery scheme.

The temporal distribution of food affects the actual availability of food. Furthermore, the value of identical aliquots of food decreases as the timespan increases, so late additions do not increase the size of females as much as the same input delivered earlier in the larval lifespan.

Prime and Average males also grow largest on the 3 day timespan treatments with only a small difference between the 2 aliquot and 4 aliquot treatments. Prime and Average males in the 2 aliquot, 3 day timespan treatment are largest and much larger than their expected values; those in the 4 aliquot, 3 day timespan treatment are a little smaller, but still larger than their expected values. This is different from the Prime and Average females above. Both Prime and Average males do much worse at the 2 aliquot, 6 day timespan treatment than expected. This is similar to the Prime and Average females. Prime males pupate before the day 6 food input, so there is even less food for them than for the Prime and Average females.

In contrast to the Prime females, the Prime males pupate latest in the 4 aliquot, 6 day timespan treatment, but still before the final input of food on day 6. The Prime males in both 6 day timespan treatments delay their pupation to grow larger, but the ones with more food (3 aliquots instead of 1 aliquot) delay longer and grow larger than the ones in the 2 aliquot treatment. The estimated growth rates follow the Prime male mass; this interaction affects the mass 5 times more than the Prime male age.

The difference between the Prime male mass and the Average male mass indicates changes in the size distribution of males and the relative advantage of the Prime male over the non-Prime males; it is almost entirely affected by the timespan. The difference between the Prime and Average males is larger with the 3 day timespan than with the 6 day timespan. Both Prime and Average males are larger in the test tubes with the 3 day timespan. This is consistent with exponential growth processes, another contrast to the observation for females.

All the Prime males pupate before the addition of the final aliquot on the 6th day. In the 6 day timespan treatments some of the non-Prime males grow larger on the final aliquot and the mass of the Average males is more similar to that of the Prime males. This is likely due to increased food for the non-Prime males after the pupation of the Prime male and the final input on day 6. This is another difference between males and females.

The AxT interaction affects the growth of males and females differently. The size of females is affected by the number of aliquots and the timespan, and all females benefit from 4 aliquots delivered in 3 days over the other treatments. The value of identical aliquots decreases with later delivery (day 3 or 4 versus day 6). The size of males is also affected, but Prime males delay pupation slightly when food is more abundant and grow larger (on 3 aliquots rather than 1 aliquot). The non-Prime males grow larger on the final aliquot in the day 6 timespan treatment, changing the size distribution among males, another difference between males and females.

The difference between the Prime female and the Average female is greatest at 2 aliquots and the 6 day timespan where they are both smallest. This is where the Prime male and the Average male are also smallest, but the difference between them is least. The Prime female monopolizes the initial food input on day 0 and reduces the size of the non-Prime females and all the males, then the non-Prime males grow larger after the Prime male pupates, and on the day 6 food input.

The difference between the Prime and Average female masses is similar across the other three treatments, although it is slightly lower for the 4 aliquot, 3 day timespan treatment where both Prime and Average female masses are highest, indicating the best food supply for this interaction. The difference between the Prime and Average male masses is similar for the two 3 day timespan treatments and for the two 6 day timespan treatments. This difference between males and females also appears to be due to the non-Prime males growing larger on the day 6 food input.

The deviations from the expected values for all 4 mass variables are greatest and negative in the 2 aliquot, 6 day timespan treatment where all of the larvae are smallest. The second largest deviations from the expected values for all 4 mass variables are in the 2 aliquot, 3 day timespan treatments; these deviations are positive. All larvae grow better than expected with a large input of food on day 3. Males grow larger and faster in the 2 aliquot, 3 day timespan treatment than in the 4 aliquot, 3 day timespan, while females grow larger and faster in the 4 aliquot, 3 day timespan. There is the same amount of food/larva in each of these treatments; males respond more to the size of the input on day 3 and females grow better on more food earlier in the larval period (the 4 aliquot treatment). This explains the larger masses of males on the 2 aliquot, 3 day timespan and the larger masses of females on the 4 aliquot, 3 day timespan.

The larvae in the 4 aliquot, 6 day timespan treatment grow more slowly and attain a smaller final mass because the food supply is delivered over more time compared to the 3 day timespan treatments. The size distribution of females in the 4 aliquot, 6 day timespan treatment (the difference between the Prime and Average female masses) is similar to the size distributions of females in both the 3 day timespan treatments, although the Prime and Average female masses are smaller in the 4 aliquot, 6 day timespan treatment. The masses of the Prime and Average males are also closer to those of the males in the 3 day timespan treatments, but the size distribution of the males is affected by the growth of the non-Prime males on the day 6 food input, so it resembles the 2 aliquot, 6 day timespan.

The amount of food in the test tubes is altered by the aliquot and timespan treatments, and this affects the growth of both males and females. The 3 day timespan treatments have the most food by day 4; the 4 aliquot, 6 day timespan has three quarters as much food, and the 2 aliquot, 6 day timespan has half as much. At the higher food level (the 3 day timespan treatment) males and females grow differently on the two aliquot treatments. At the lower food levels (the 6 day timespan treatment) males and females are disproportionately smaller in the 2 aliquot treatment compared to the 4 aliquot treatment. Furthermore, the distribution of sizes of females is largest in this treatment (2 aliquot, 6 day timespan) while the distribution of sizes of males is smallest. The 6 day timespan treatment allows the non-Prime males to grow larger on the additional food in the final aliquot affecting the distribution of sizes among males, but not allowing the non-Prime males to catch up with non-Prime males in treatments with more food earlier in the larval period. The addition of a large amount of food on day 6 (the 2 aliquot treatment) allows females to switch from retention back to active filtering and this increases the size distribution among females, but also doesn’t allow the females to catch up to females in treatments with more food earlier in the larval period. The distribution of food (the aliquot and timespan treatments) affects the growth of males and females differently, and independently of the food level, density, and competition (food x density).

The difference between the Prime female and the Average female, between the Prime female and the Prime male, and between the Average female and the Average male, are all smallest in the 4 aliquot, 3 day timespan treatment. The difference between the Prime male and Average male is largest in this treatment. The difference between the Prime female and the Average female, between the Prime female and the Prime male, and between the Average female and the Average male, are all largest in the 2 aliquot, 6 day timespan treatment. The difference between the Prime male and Average male is smallest in this treatment. Food level is not part of this interaction, but two attributes of the food supply are, and these affect the availability of food in the test tubes. The treatment, 4 aliquots and the 3 day timespan, provides the most food, earliest in the larval period. The treatment, 2 aliquots and the 6 day timespan, provides the least food early in the larval period; the final aliquot is added after the Prime males pupate, and the Prime females delay pupation for more than a day after the next latest treatment. At the highest food availability (4 aliquots, 3 day timespan) the females grow largest and fastest, the non-Prime females grow almost as large as the Prime females, the Prime males grow large (second largest and second fastest growth rate) and there is a large difference between the Prime and non-Prime males, suggesting exponential growth due to active filtering for abundant particles, but also that males and females are feeding on different subsets of the yeast food supply. At the next highest food availability (2 aliquots, 3 day timespan) the females grow almost as large, almost as fast, the non-Prime females are a little smaller relative to the Prime females, the males grow largest, the Prime males grow fastest, and there is also a large difference between the Prime and non-Prime males. The males do better relative to the females because of the large input of food on day 3 (compared to the multiple inputs that favor female growth). At the third highest food availability (4 aliquots, 6 day timespan) the females are smaller, grow more slowly, the non-Prime females are the same relative to the Prime females as in the second highest, the males are smaller, the Prime males pupate latest, and there is a small difference between the Prime and Average males. The change in the size distribution of the males, along with the smaller size of the females suggests that the females switch to retention from active filtering and reduce the availability of food even further than the aliquot and timespan treatment. The Prime males delay pupation to grow larger (on the third aliquot of food on day 4), but still pupate before the final aliquot on day 6. At the lowest food availability (2 aliquots, 6 day timespan) the females are smallest, grow slowest, pupate much later, the difference between the Prime and Average females is larger, the males are smaller and the difference between the Prime and Average males is smallest. The change in the size distribution of the males, along with the smaller size of the females again suggests that the females switch to retention from active filtering and reduce the availability of food even further than the aliquot and timespan treatment. The increase in the size distribution of the females suggests that they switch back to active filtering after the final aliquot is added. The non-Prime males may also grow larger on this added food. None of the females or non-Prime males grow as large as their counterparts in other treatments. This describes the significant interaction of the two attributes of food availability affecting the mass and age at pupation of males and females independently of food level, density, or competition (food x density).

**Density, aliquot and timespan**

The remaining interactions cross density with aliquot and timespan (DxAxT, DxA, DxT). Aliquot and timespan are aspects of the food supply independent of each other and of the food level. In the previous interactions, both aliquot and timespan, and their interaction, AxT, appear to alter the availability of food for the larvae. The interaction of density (4 larvae or 8 larvae per test tube) and food level (16 mg food per test tube and 32 mg food per test tube) produces 4 different competitive environments (the FxD set of interactions, above). These three interactions between density, aliquot and timespan may also result in different competitive environments, or they may reveal additional information about growth. Competition among larvae alters the mass and age of the Prime individuals, the growth rate of the Prime individual, the Average mass, and the difference between the Prime and the Average mass. Furthermore, the specific differences are sex-dependent. For females, the 4 aliquot, 3 day timespan appears to provide the largest amount of food, earliest in the larval period (at both food levels) and represents the best growth conditions in the AxT interaction (above). Also for females, the 2 aliquot, 6 day timespan provides the least amount of food early in the larval period and represents the worst growth conditions across this interaction. The 2 aliquot, 3 day timespan and the 4 aliquot, 6 day timespan are intermediate, but the expectation is that the 3 day timespan treatment will provide more food earlier than the 6 day timespan. High density should increase competition for food and it should have a disproportionate effect at the lower food supply (both food levels, 32 mg/test tube and 16 mg/test tube, are present in each treatment combination, so this would be competition independent of the food level).

The 3-way interaction, DxAxT, has the smallest R squared value of any significant contrast in the MANOVA, but is the highest order interaction for these factors for the two dependent variables, Average male mass and Prime female mass. DxT is the second most significant interaction in the MANOVA (after FxDxT). It is significant for all 7 dependent variables in the ANOVAs (also like FxDxT). DxA is not significant in the MANOVA, but is significant for Prime female age at pupation in the ANOVA.

**The DxAxT interaction (R squared = 0.15)**

This interaction looks at the effect of density on the joint effect of aliquot and timespan; both aliquot and timespan are attributes of the distribution of food over time. The interaction of density, aliquot and timespan is significant only for Average male mass and Prime female mass in the ANOVAs, but it is significant (P<0.05) in the MANOVA (R squared = 0.15). The interaction explains only 1% of the variance for the Prime female mass and the same for the Average male mass, but the AxT and DxT interactions are among the most significant across the ANOVAs and the 3-way interactions have to be addressed before the 2-way interactions. (The AxT interaction was addressed before the DxAxT interaction because it does not involve density as a factor and therefore describes the growth of larvae regardless of competition among them.)

This interaction affects the Prime female mass and the Average male mass. Because the Prime female mass is included in the Average female mass, the non-Prime females must be affected collectively in an equal and opposite manner than the Prime female. The net effect on the Average female mass is not significant for the interaction. The effect of this interaction on the Average male mass but not on the Prime male mass indicates that only the non-Prime males are affected by the 3-way interaction.

This interaction involves density and two attributes of the food supply describing the temporal availability of food. It is possible that this interaction describes another type of competition in addition to the food level x density competition described in the first set of interactions (FxDxT, FxDxAxT, FxDxA, FxD). Within each density, food should be most abundant in the 4 aliquot, 3 day timespan and most restricted in the 2 aliquot, 6 day timespan. The largest Prime females and Average males should be in the low density, 4 aliquot, 3 day timespan treatment and the smallest should be in the high density, 2 aliquot, 6 day timespan treatment. The smallest Prime females and Average males are in the high density, 2 aliquot, 6 day timespan treatment, but neither the Prime female nor the Average male are largest in the low density, 4 aliquot, 3 day timespan treatment. The Prime female and the Average male deviate from the predicted optima in different ways. At the low density, the size of the Prime female follows the food/larva after day 4 and the total food after day 4 (they are in the same order). At the low density, the size of the Average male follows the estimated growth rate of the Prime male, an indicator of the competitive stress in the test tubes.

In the FxD set of interactions (above), competition among females results in large Prime and Average females in the test tubes with the least competition. These Prime females have the fastest estimated growth rate and a small difference between the Prime female mass and the Average female mass, indicating that all females have sufficient food to grow and pupate. In the test tubes with the most competition, the Prime and Average females are the smallest, the growth rate is the slowest, and there is also a small difference between the Prime female mass and the Average female mass. As the larvae grow, the females switch from actively filtering particles to retaining them in their guts; this compresses the size distribution of the females resulting in the small difference between the Prime and Average females. In the test tubes with intermediate levels of competition the Prime and Average females are intermediate in size, the growth rates are intermediate, but the difference between the Prime and Average females is largest at the high density. There are 8 larvae competing for 4 mg food/larva and the Prime female has an advantage over the smaller larvae. The higher total food per test tube allows the Prime female to grow almost as large as the Prime female in the least competition treatment, while the Average female is only a little larger than the Average female in the low density intermediate competition treatment.

If the DxAxT interaction is the result of competition for food, the females at the highest food/larva (4 mg food/larva or greater) should resemble the females in the least competition treatment, the females at the lowest food/larva should resemble the females in the most competition treatment, and the intermediate females should resemble the females in the intermediate competition treatments. Specifically, the intermediate treatments at high density should allow the Prime females to grow almost as large as those in the least competition treatment while the Average females grow only as large as the Average females in the low density intermediate competition treatments. This is clearly not true. The three largest Prime females occur at the low density; they have the three fastest estimated growth rates and they are associated with the three largest Average female masses. The next largest female does occur in the high density treatment, but the gap between the Prime and Average females is no larger than any of the other intermediate treatments. Finally, the smallest Prime and Average females occur in the high density, 2 aliquot, 6 day timespan treatment, which has the largest difference between the Prime and Average masses. Overall, the masses of the Prime female correspond to the food availability due to the DxAxT treatments, but the size distributions among the females associated with competition (in the FxD set of interactions) do not appear. For the Prime female, the DxAxT interaction describes the growth of females rather than competition among females. As the Prime female benefits from the aliquot and timespan treatments, the non-Prime females correspondingly decrease in size (collectively) so the Average female mass is not affected by this interaction. For the Prime female at the highest food/larva, receiving the large input of food on day 3 (low density, 2 aliquots, 3 day timespan) increases the mass, decreases the age at pupation and increases the estimated growth rate. At the intermediate food/larva (2 mg or 4 mg after day 4), the Prime female grows largest with 4 aliquots and the 3 day timespan (high density). There are two other treatments that have the same food/larva; these both achieve the same mass so receiving the food on day 0 and day 3 has the same effect as receiving it on day 0. At the intermediate food/larva 4 aliquots are better than 2 or 1. The lowest food/larva treatments grow to a smaller mass and grow more slowly according to the amount of food.

The Prime female is largest at the low density, 2 aliquot, 3 day timespan. This treatment also shows the fastest estimated growth rate, the earliest age at pupation, and the largest difference between the Prime female and the Prime male. The Prime female in the low density, 4 aliquot, 3 day timespan is second largest, has the second fastest estimated growth rate, the second earliest age at pupation and the second largest difference between the Prime female and the Prime male. The Prime female appears to benefit from the large input of food on day 3 compared to the smaller inputs on days 1-3. The relative abundance of food particles due to the day 3 input cause the females to actively filter; they grow larger and the size distribution also increases in size (the non-Prime females are reduced in size compared to the Prime female). This accounts for the different outcomes between the two highest food/larva levels for Prime females. The timing of the larger food input (2 aliquot) on day 3 favors the growth of the Prime female over the other larvae. This is similar to the effect of large food inputs on day 3 for Average females (FxDxAxT interaction) and Prime females (FxAxT).

Females appear to grow in response to the food/larva and total food at low density, but the amount and timing of the food input on day 3 increases the size of the Prime female (and implicitly reduces the size of the non-Prime females) relative to other equal and similar food levels. The mass of the Prime females can be explained by exponential growth processes without invoking competition.

This interaction arises because the Prime female grows larger in the low density, 2 aliquot, 3 day timespan treatment than in the low density, 4 aliquot, 3 day timespan treatment. The main effects predict that the 4 aliquot treatment should be larger. The large increment of food on day 3 benefits the Prime female at the expense of the Average female at this high food level. The size of the Prime female in the test tubes with lower food levels in this interaction follow the food/larva level after day 4. In the case where the food/larva is the same across cells, the total food affects the outcome, and for the two treatments where the food/larva and the total food are equal, the 4 aliquot treatment is larger than the 2 aliquot treatment.

Males compete with other males and with females for food; females have the advantage due to their larger size, but males appear to compete among themselves more intensely and for an aspect of the food resource that is different from the females. This interaction affects the growth of Prime females, and collectively diminishes the non-Prime females where it increases the size of the Prime females, but does not appear to affect competition among females. It is possible that this interaction affects the competition among the non-Prime males independently of the effect on Prime females.

In the FxDxT and FxD interactions, Prime and Average males are largest in the least competition treatments, and smallest in the most competition treatments. The high density intermediate competition treatments (4 mg food/larva) produce larger Prime males and smaller Average males than the low density intermediate competition treatments (also 4 mg food/larva). The largest difference between Prime and Average males is in the high density intermediate competition treatments. Non-Prime males grow larger than the Prime males in the most competition treatment. In the FxD interaction, this increment is 0.01 mg larger than the Prime males. In the FxDxT interaction, the increment is 0.14 mg larger. The FxD increment represents only the release of competition after the Prime male pupates, while the FxDxT increment represents both the release of competition and the additional food at the end of day 6 in the 6 day timespan treatment. The effect of the release from competition with the Prime male is much smaller than the effect of the additional food after day 6.

In the FxDxT interaction, the total food after day 4 explains the size order of Prime males when the food/larva is 4 mg or above. The food/larva after day 4 explains the size order of Prime males below 4 mg food/larva. The outcome of competition for the Prime males is directly related to the availability of food after day 4. This is not true of the Average male mass. Prime males are smaller in the 6 day timespan for each treatment. The non-Prime males grow larger in the 6 day timespan treatment than the 3 day timespan treatment in the least competition test tubes. The non-Prime males grow larger than the Prime males in the most competition test tubes. Both are due to the release from competition and the additional food after day 6. In the intermediate competition treatments (4 mg food/larva), the effect of the 6 day timespan is to reduce the size of both Prime and Average males, and to reduce the difference between the Prime and Average males across the timespan treatments at the high density, but to increase that difference at the low density. There appears to be a small benefit to the non-Prime males in the high density, 4 mg food/larva treatment with the 6 day timespan compared to the low density, 4 mg food/larva treatment with the 6 day timespan; this is probably due to the higher total food in those test tubes.

The Average male mass in the DxAxT interaction is similar to the Average male mass in the FxDxT interaction in several ways. First, the largest Average male occurs in the 6 day timespan treatment with the most food; these are the least competition treatment in FxDxT and the low density, 4 aliquot treatment in DxAxT. These are not the treatments with the most food after day 4 in either interaction. The Prime males in these treatments are not the largest in the respective interactions, but the differences between the Prime male mass and the Average male mass are the smallest (0.02 mg for FxDxT, 0.01 mg for DxAxT, but see below). Second, the next largest Average males occur in the 3 day timespan treatments with the most food/larva; these are also the treatments with the largest Prime males, but not necessarily in the same order as the Average males. There are 4 treatments in FxDxT where all test tubes have 4 mg food/larva or higher, and 3 treatments in DxAxT where all test tubes have 4 mg food/larva or higher. There is a gap between the 3 or 4 treatments above and the remainder in each interaction (0.36 mg for FxDxT and 0.31 mg for DxAxT). Below this gap the Average male mass and the Prime male mass are in the same order, roughly corresponding to the food/larva after day 4. In both interactions, the smallest Average males grow larger than the Prime males in their respective treatments; both treatments are 6 day timespans, so the non-Prime males grow larger on the release from competition and the additional food on day 6. In each interaction there is one treatment at an intermediate food level that results in a large Prime male mass, a smaller Average male mass, and a large difference between them. In the FxDxT interaction this treatment is the high food, high density, 3 day timespan (Prime male, 2.73 mg; Average male 2.57 mg; difference 0.16 mg). In the DxAxT interaction the treatment is the high density, 2 aliquot, 3 day timespan (Prime male, 2.44 mg; Average male, 2.29 mg; difference 0.15 mg). The relationships between Prime male mass, Average male mass, the difference between the two masses, and the food/larva after day 4 suggest that the non-Prime males are competing in the DxAxT interaction in the same way that they are competing in the FxDxT interaction; aliquot interacts with density and timespan for the non-Prime males similarly to the way that food level does in the FxDxT interaction.

In the FxDxT interaction for Average male mass, the 6 day timespan results in a smaller Average male than the 3 day timespan (with the exception of the largest Average male). Food and density are more important to the order (and to food/larva) than timespan. In the DxAxT interaction for Average males, the 6 day timespan results in a smaller Average male than the 3 day timespan (again with the exception of the largest Average male). The 4 treatments with the 3 day timespan follow the largest Average male (6 day timespan). The 3 smallest Average males are in test tubes with the 6 day timespan. Timespan is as important as density and both are more important that aliquot to the size order of the Average males. The 2 aliquot treatment produces larger Average males at both densities with the 3 day timespan, but 4 aliquots are better than 2 aliquots with the 6 day timespan. The simple explanation for the different effects of the 2 or 4 aliquot treatments at high and low food availabilities is that the 2 aliquot, 3 day timespan combination puts a large amount of food in the test tube on day 3 when the larvae are growing fastest and this increases the size of the male larvae. The 4 aliquot, 6 day combination provides more food, earlier than the 2 aliquot, 6 day combination. Both add more food on day 6, which benefits the non-Prime males, but the third input on day 4 has more benefit than a larger input on day 6.

The Average male is largest at the low density, 4 aliquot, 6 day timespan. This treatment is associated with the fastest Prime male growth rate suggesting that the competitive environment for males in these test tubes is less stressful even though the total food and food/larva levels are not the highest after day 4. Males are competing differently than females for the same yeast food resource. The next two largest Average males are at the low density, 2 aliquot, 3 day timespan, and the low density, 4 aliquot, 3 day timespan. These correspond to the two largest Prime females and the two highest total food and food/larva levels. The Prime male estimated growth rates for these treatments are only a little smaller than for the low density, 4 aliquot, 6 day timespan. The non-Prime males benefit from the lower competitive stress in these test tubes, and the non-Prime males in the low density, 4 aliquot, 6 day timespan treatment also benefit from the added food on day 6. This is the only treatment in which the non-Prime males grow larger on the 6 day timespan than on the 3 day timespan (at the same density and aliquot).

The non-Prime males in the low density, 4 aliquot, 6 day timespan treatment do considerably better than the non-Prime males in the low density, 2 aliquot, 6 day timespan treatment because the lower food availability in the 2 aliquot test tubes causes the females to switch to retention and the males are reduced in size relative to the females (0.43 mg for the Prime males and 0.50 mg for the Average males, between the 4 aliquot and 2 aliquot treatments at low density and the 6 day timespan). The final aliquot of food is larger on day 6 for the 2 aliquot treatment, but the smaller larvae are unable to make up the difference on the late input of food.

The two low density treatments with the 3 day timespan result in Average male masses that are almost as large as the Average male in the low density, 4 aliquot, 6 day timespan, but competition with females and competition with the Prime male affect the size of the non-Prime males and there is no addition of food after the Prime male pupates to boost the size of the non-Prime males. The non-Prime males in the high density, 2 aliquot, 6 day timespan treatment also grow larger on the final aliquot of food on day 6; they grow larger than the Prime male in that treatment. It is likely that the non-Prime males in the other 6 day treatments benefit from the final aliquot as well, but it isn’t obvious in the numerical relationships.

Non-Prime males appear to grow in response to the competition with the Prime males, but benefit from the final input on day 6. At the lowest level of competition with the Prime males (the fastest Prime male growth rate), the non-Prime males grow to their largest size. At the highest level of competition, the Prime and non-Prime males are smallest, but the non-Prime grow larger than the Prime males in the same test tubes due to the day 6 addition of food.

The order of the size of the Average male does not correspond to either the total food after day 4 or the food/larva after day 4. The largest Average males are in the test tubes where the Prime male has the highest estimated growth rate. In this interaction, the Prime male estimated growth rate predicts the Average male mass better than the total food or food/larva or any combination of the independent factors. The Prime male mass and age at pupation are not affected by this interaction, but the growth of the Prime male predicts the mass of the Average male. Growth rate is an indication of the competitive environment; high growth rates occur in the test tubes with the least competition (in the FxD interactions; estimated growth rates in the FxDxT interaction are in the same order as Prime males size and food level). This suggests that the Prime males are not affected by competition governed by aliquot and timespan, but that the non-Prime males are affected.

**The DxA interaction (not significant in the MANOVA)**

The Prime female age is the only variable significantly affected by this interaction in the ANOVA. This is the smallest of the 4 significant interaction contrasts for Prime female age. None of the higher order interactions between density and aliquot are significant for the Prime female age at pupation, so this represents the only interaction between these factors for Prime female age. The main effects predict that low density is better (earlier) than high density, and 4 aliquots are better than 2 aliquots.

The effect of density on the age at pupation implies possible competition for an attribute of the food supply. This interaction doesn’t involve either the food level or timespan, so the interaction reflects only the effect of the density and the number of aliquots on the age at pupation. Density has a larger effect than aliquot and the ages at pupation are according to the expected main effects. The asymmetry that causes this interaction for the Prime female age appears to be the small difference due to aliquot at low density compared to the much larger effect at high density. Prime females pupate at almost the same age at low density regardless of aliquot treatment. Both low density treatments pupate earlier than projected, the 4 aliquot treatment first followed closely by the 2 aliquot treatment. The source of the interaction appears to be that the 2 aliquot treatment is much earlier than projected by the main effects, compared to the 4 aliquot treatment. At the high density, Prime females pupate much later in the 2 aliquot treatment than in the 4 aliquot treatment, and much later than projected by the main effects. The Prime female mass at pupation, the estimated growth rate and the food/larva after day 4 all show the same order as the Prime female age, but do not explain why the two low density treatments are so similar. In the other significant interactions for the Prime female age at pupation, increased total food is related to earlier pupation. Total food does not appear to be related to age at pupation in this interaction. These two treatments are much closer to each other than expected based on the difference in the food/larva. The aliquot treatment affects the timing of food inputs and the density affects the food/larva. The test tubes with low density and 2 aliquots receive an average of 3 mg food/larva on day 0. They receive another 1.5 mg food/larva (average) on day 3 and another 1.5 mg on day 6. The test tubes with low density and 4 aliquots receive an average of 1.5 mg food/larva on day 0, 0.75 mg more on day 1, 1.5 mg more on day 2, 0.75 mg more on day 3, 0.75 mg more on day 4 and 0.75 mg more on day 6. The Prime females in both these treatments mostly pupate before the food addition on day 6. Prime females pupate relatively early in the test tubes with the most initial food (low density and 2 aliquots), although they don’t grow as large, as fast or pupate as early as those in the test tubes with more food during the larval period (low density and 4 aliquots). This suggests that the initial availability of food affects the trigger for pupation for females. In this interaction, the large initial input (low density, 2 aliquots) results in much earlier pupation than expected for the Prime female while not affecting Prime female mass or any other dependent variables. Based on the size distribution of the Prime and Average females, this appears to be due to exponential growth processes rather than competition. The mass at pupation of females is affected by large inputs of food on day 3 (FxDxAxT for Average females, FxDxA for all females, DxAxT for Prime females), but the age at pupation appears to be affected the amount of the initial input on day 0.

**The DxT interaction (R squared = 0.84)**

The interaction between density and timespan shows the residual effect after the higher order interactions have been explained (FxDxT for all variables, FxDxAxT for Average female mass, DxAxT for Prime female mass and Average male mass). For the four mass variables and the two age variables the main effects are: low density is better than high density and the 3 day timespan is better than the 6 day timespan.

Because this interaction involves density and timespan, an attribute of the food supply, it is possible that competition is involved. Food level (total food) and density are independent factors in the experiment, and they jointly affect competition among the mosquito larvae, but growth and competition among the larvae are also affected by food/larva, which is necessarily confounded with both food level and density. The DxT interaction reflects the residual effects of density and timespan on growth and competition. The interpretation of the DxT interaction is that the number of larvae affects the growth of larvae at different timespan treatments. Timespan clearly affects the amount of food in the test tubes on a daily basis, so could be affecting the competition among larvae as well as growth of larvae.

Referring to the discussion of FxD competition in the DxAxT interaction, the same relationships should apply to the DxT interaction.

If the DxT interaction is the result of competition for food, the females at the highest food/larva (4 mg food/larva or greater) should resemble the females in the least competition treatment, the females at the lowest food/larva should resemble the females in the most competition treatment, and the intermediate females should resemble the females in the intermediate competition treatments. The low density, 3 day timespan treatment has 4 mg or 8 mg food/larva by day 3, so it should resemble the least competition treatment. The high density, 6 day timespan treatment has much less food (1 mg, 1.5 mg, 2 mg or 3 mg food/larva by the end of day 4); this is the lowest food/larva across this interaction, so it should resemble the most competition treatment. However, the two intermediate treatments are not precisely similar to the intermediate competition treatments. The high density, 3 day timespan treatment receives 2 mg or 4 mg food/larva at the end of day 3, but the low density, 6 day timespan treatment receives 2 mg, 3 mg, 4 mg or 6 mg food/larva at the end of day 4. By analogy with the FxD interaction, the Prime female should be larger in the high density intermediate food level, but in the DxT interaction, the low density intermediate food level does have more food.

For females, the largest Prime female mass, Average female mass, estimated growth rate, and the earliest age at pupation all occur in the low density, 3 day timespan treatment. This treatment is also associated with the smallest difference in mass between the Prime and Average females, and the largest positive deviations from the expected values for the masses of the Prime and Average females. All these indicators line up with the least competition treatment in the FxD set of interactions. The smallest Prime female mass, Average female mass, estimated growth rate, and the latest age at pupation occur in the high density, 6 day timespan treatment. This treatment is also associated with the largest difference in mass between the Prime and Average females, and the largest negative deviations from the expected values for the masses of the Prime and Average females. This doesn’t correspond entirely with the outcomes from the most competition treatment (FxD set of interactions); specifically, at low food levels the Prime female should switch from actively filtering to retention and this should result in a smaller size distribution between the Prime and Average females. Despite the low food/larva level, the ongoing food inputs over the timespan appear to allow the females to actively filter particles rather than switching to retention.

The two intermediate treatments fall between the low density, 3 day timespan and the high density, 6 day timespan treatments for the masses of the Prime and Average females, the estimated growth rates, the ages at pupation, the differences in mass between the Prime and Average females, and the deviations from the expected values. Half of the test tubes with the high density, 3 day timespan treatment have more total food after day 4 (32 mg) than any of the test tubes with the low density, 6 day timespan treatment. However, half of the test tubes with the low density, 6 day timespan treatment have more food/larva after day 4 (2 mg, 3 mg, 4 mg, 6 mg versus 2 mg, 4 mg). If competition were the predominant factor influencing the growth of these females, the high density, 3 day timespan treatment should produce the larger, faster growing, earlier pupating females with a larger difference between the Prime and Average females. This is not true, except that the Prime females in the high density, 3 day timespan treatment pupate slightly earlier than those in the low density, 6 day timespan treatment. With the exception of the ages at pupation, the outcome of this interaction for females seems best explained by the food/larva rather than total food or competition. The DxT interaction appears to describe exponential growth processes for females. The residual effect of this interaction on both Prime female mass and Average female mass appears to be an almost linear increase as the food/larva increases.

For males, the largest Prime male mass, Average male mass, and estimated growth rate, all occur in the low density, 3 day timespan treatment. This treatment is also associated with the largest positive deviations from the expected values for the masses of the Prime and Average males, but not with the earliest age at pupation or the smallest difference in mass between the Prime and Average males*.*  All these indicators line up with the least competition treatment in the FxD set of interactions. The smallest Prime male mass, Average male mass, estimated growth rate, and the latest age at pupation occur in the high density, 6 day timespan treatment. This treatment is also associated with the largest negative deviations from the expected values for the masses of the Prime and Average males, but not with the largest difference in mass between the Prime and Average males. Non-Prime males grow larger after the pupation of the Prime males, and especially on the final input of food on day 6. Some non-Prime males in the high density, 6 day timespan grow larger than the Prime male in that treatment. For males, this interaction resembles the competition in the FxD set of interactions.

For males, the two intermediate treatments also fall between the low density, 3 day timespan and the high density, 6 day timespan treatments for the masses of the Prime and Average males, and the estimated growth rates, but not for the ages at pupation, the differences between the Prime and Average male masses, and the deviations from the expected values. Like the Prime male in the high food, high density treatment (FxD interaction), the Prime male in the high density, 6 day timespan treatment is larger than in the low density intermediate treatment, and the difference between the Prime and Average males is largest across the interaction. This resembles the competitive interaction (FxD set of interactions) and differs from the outcome for the females.

For males, the congruence between the Prime male mass, the Average male mass, the estimated growth rate, the difference between the Prime male and the Average male and the deviations from the expected values of the masses are clear. Based on the outcomes for the mass of males, the DxT interaction looks like males are competing for food due to the timespan treatment. Rather than the linear residual relationship between food/larva and mass at pupation observed for the females in this interaction, the males are disproportionately smaller in the high density, 6 day timespan treatment. The DxT interaction is analogous to the FxD interaction for males; competition appears to affect the outcome of this interaction despite the absence of food level as a factor. Furthermore, the non-Prime males experience a release from competition after the Prime male pupates, and they also grow larger on the input of food on day 6. This residual effect is on the order of the release from competition alone in FxD (0.01 mg incremental difference in size between the Average male mass and the Prime male mass).

The ages at pupation for males or for females do not follow the food/larva in this interaction. The Prime female age at pupation in the FxDxT interaction follows the total food after day 4. The Prime female age at pupation in the FxD interaction is affected by both the food/larva and the total food. The Prime females in the two FxD intermediate competition treatments have the same food/larva after day 4; the Prime females with the higher total food (high food, high density) pupate earlier than those with the lower total food (low food, low density). The Prime females in the intermediate treatments for the DxT interaction pupate earlier in the test tubes with less food/larva after day 4, but the higher total food. Total food appears to be more important to the Prime female age at pupation that the food/larva in this interaction as well as in the FxDxT interaction. The Prime female mass and the Average female mass follow the food/larva rather than the total food.

The Prime males pupate early in three of the four treatments in the DxT interaction. They pupate latest in the high density, 6 day timespan treatment which appears to correspond to the most competition treatment in the FxD set of interactions. The Prime males pupate later than the expected value in this treatment as well. Competition delays pupation of the Prime male in the DxT interaction as well as in the FxD set of interactions, although all the Prime males pupate before the final input of food on day 6.

The Prime male ages at pupation in the other three treatments do not correspond to the food/larva or the total food after day 4. The treatment with the earliest pupation is the low density, 6 day timespan treatment, which has less food (both food/larva and total food) than the low density, 3 day timespan treatment. It appears that the Prime males in the low density, 3 day timespan treatment delay pupation due to high levels of food (both food/larva and total food are highest in this treatment). The Prime males in the high density, 3 day timespan treatment also delay pupation relative to the low density, 6 day timespan treatment; the food/larva after day 4 is less than in the earliest pupating treatment, but the total food is higher. The Prime male in the FxDxT interaction grow according to the total food after day 4 in the test tubes with abundant food. High levels of total food may cause Prime males to delay pupation, perhaps to grow larger or to improve physiological status.

In other FxD competition interactions, the Prime males appear to delay pupation in some treatments when food is available and also when competition is intense. It is not clear what causes the Prime male to delay pupation when food is available; neither food/larva nor total food consistently predict this response. Prime males consistently delay pupation when competition is intense, but do not delay it long enough to benefit from the additional food on day 6 (in the day 6 timespan treatments).

**Males versus females**

Density and timespan jointly affect the mass and age variables for males and females, but they affect them differently for the two sexes. Females grow largest, fastest, the Prime females pupate earliest, and the relative advantage of the Prime female over the non-Prime females is smallest at the low density and 3 day timespan. Either the 6 day timespan or the high density reduce the size, the growth rate, and increase the age at pupation and the relative advantage of the Prime female over the non-Prime females. The test tubes with the high density and the 6 day timespan produce the smallest females, the slowest growth rate, the latest age at pupation, and the largest relative advantage of the Prime female over the non-Prime females. The females do disproportionately well in the low density, 3 day timespan and disproportionately poorly in the high density, 6 day timespan treatments. The interaction between density and an attribute of the food supply could be an indication of competition independent of the food level (FxD), but there is no evidence for it. As the food availability early in the larval period changes due to a longer timespan and higher density, the females all pupate at smaller sizes, grow more slowly, and the size distribution increases. The simple explanation is that these are the result of exponential growth processes rather than competition, because females should switch from active filtering to retention at low food levels (and they appear to do so in other interactions in this experiment), and that would cause the size distribution to compress rather than to increase as observed. This interaction includes only density and timespan; the food level and the aliquot treatments are not important, suggesting that changes in food level and aliquot are the triggers than cause females to switch from actively filtering to retention.

Males are also largest and grow fastest at the low density, 3 day timespan treatment, and smallest, grow slowest and pupate latest at the high density, 6 day timespan treatment. They differ from the females in that the earliest age at pupation is in the low density, 6 day timespan treatment and relative advantage of the Prime male over the non-Prime males is more affected by timespan than by density. The size distribution of the males increases with density in the 3 day timespan treatments, similar to that of the females. However, the size distribution of males decreases with increasing density in the 6 day timespan treatments, probably due to the added food after the Prime male pupates. In contrast with the females in this interaction, the Prime male mass and age, the Average male mass, and the difference between the Prime and Average male masses jointly resemble the pattern in the set of FxD interactions that describe competition (above). This suggests that males are competing for food in response to the changes in availability caused by the timespan treatment independently of the food level treatment.

The estimated growth rates for Prime males and females decrease from the best treatment (low density, 3 day timespan) to the worst (high density, 6 day timespan), but there are differences that reflect differences in both the masses and ages between the sexes. Prime females grow much faster in the best treatment and much slower in the worst, with the two middle treatments being close together and about halfway between the extremes. The Prime females in the best treatment are larger than expected and pupate earlier than expected; those in the worst treatment are smaller than expected and pupate later than expected. The two in the middle are similar to their expected values in size, but the larger of the two pupates later, so the estimated growth rates are closer together than their masses.

The three largest Prime males grow at about the same rate. The one with the largest mass pupates later than the other two, but there is less difference in size and age at pupation among the three largest than there is between them and the worst treatment (high density, 6 day timespan, the same as the worst treatment for the females).

Males and females delay pupation to increase size at low food availability (the worst treatment, high density, 6 day timespan), but males pupate on day 5 or day 6 while females delay pupation well after day 6. Males also delay pupation when food is abundant (the best treatment, low density, 3 day timespan), although they still pupate on day 5 or day 6.

Females grow larger and faster than males in all treatments. The relative difference in size of the Prime female over the Prime male and that of the Average female over the Average male could also indicate competitive interactions. These differences correspond to the relative advantage of the Prime female over the Prime male and of the non-Prime females over the non-Prime males. In both cases the advantage decreases as the mass of the males and females decreases, with one exception. The Prime male in the high density, 3 day timespan treatment is relatively larger than in the high density, 6 day timespan (so the difference between the Prime female and the Prime male is smaller). Prime males in this treatment are also relatively larger than the Average males. The high density with the 3 day timespan increases the relative advantage of the Prime male over the non-Prime males, but also as compared to the females. This interaction does not affect competition among females, but it appears to affect competition among males and possibly between males and females.

**Main effects**

The main effects represent the residual variance that is explained by the individual factors after the interactions have been removed. For the 4 mass variables and 2 age variables, the explained variance due just to the main effects is around 50% (45% to 56%). This is primarily an indication that the levels of the factors affected the growth of the larvae as hoped for during the design of the experiment. Two aspects of the main effects indicate something novel about the biology of these larvae. First, the Survival of larvae is not affected by food, density or timespan except through interactions; there is no residual significance for any of these three factors on Survival. Survival is affected by the factor aliquot, and there are no significant interactions between aliquot and any other factor. Food, density and timespan jointly affect the Survival of larvae, but aliquot only affects Survival independently of the other factors. The three interactions with food, density and timespan account for 15% of the variance across the ANOVA, and the main effect of aliquot accounts for 12%. Survival is better on 2 aliquots than on 4 aliquots. Since there is no interaction between aliquot and the other factors, the biological reason for the difference is not obvious.

The second novelty within the main effects is that both female mass variables have no residual effect of density; all of the effects of density on female mass are explained by the interactions between density and the 3 factors that describe the amount and timing of food inputs. This contrasts with the large residual effect of density on age at pupation for the Prime females (32%). It also contrasts with the significant main effect of density on male mass, although the explained variance is low (1%, and less than 1%, for Prime and Average male mass, respectively). The Prime male age at pupation also has a large residual effect of density (31%).
