# Supplementary material for: Competition and growth among Aedes aegypti larvae: Effects of distributing food inputs over time
Source: PLoS One. 2020 Oct 2;15(10):e0234676. doi: 10.1371/journal.pone.0234676 (PMC7531853; doi:10.1371/journal.pone.0234676)
Supplement: S5 Text — Third experiment analysis of individual ANOVA contrasts. (DOCX) [file pone.0234676.s117.docx]

**S5 Text. Third experiment analysis of individual ANOVA contrasts.**

**Overview**

The first experiment shows that the amount and timing of food inputs during the larval period affects competition and growth among mosquito larvae. Early food inputs are more beneficial to growth and reduce competition among larvae, but large inputs during the 3^rd^ and 4^th^ instars may also affect the outcome. The late food inputs (on day 6) had little effect on the outcomes except for the non-Prime males, which grow larger on the extra food after the Prime male has pupated.

The purpose of this experiment is to examine the effect of late additions of food on the growth of *A. aegypti* mosquito larvae. Individual larvae are fed a minimal amount of food initially, then more food is added after a delay of either 6 or 8 days. Pupal weight (mg), age at pupation (days) and sex are recorded upon pupation. The data are analyzed as a MANOVA with 3 factors: amount of incremental food (1 mg, 2 mg, or 3 mg); delay (6 or 8 days between the start of the experiment and the input of the incremental food); and sex (m or f). The two dependent variables are: mass at pupation (mg) and days to pupation after the second input of food. For the analysis, the age at pupation is calculated as: day of pupation MINUS the delay, 6 days or 8 days, depending on the treatment. The transformation of the age by subtracting the delay removes the numerical effect of the delay from the biological effect of the delay.

The mosquito larvae are not expected to be able to pupate on the initial amount of food (1 mg dry weight of yeast), but should be able to survive until the second food input. The amount of food at the second food input (1 mg, 2 mg or 3 mg, dry weight of yeast) should allow pupation, and the mass and age at pupation should improve with increasing amounts. The shorter delay should be better for both size and age than the longer delay. Better outcomes mean larger masses and earlier ages at pupation. Males and females may delay pupation in response to abundant food, so there are possible interactions between the mass and age variables, which should show up in the MANOVA. There is no competition in this experiment because larvae are alone in their test tubes, so sex is treated as a factor rather than as a covarying dependent variable.

**Results**

The MANOVA and the ANOVAs for mass and age at pupation are presented in the supporting file, S2 Text. The detailed analyses of the individual ANOVA contrasts for the two dependent variables, mass and age at pupation, are presented here.

**The food 2 x delay x sex interaction**

S56 Table shows the means and standard errors for mass for males and females by food input and delay treatments for the most significant interaction contrast, food 2 x delay x sex. It is the only 3-way interaction that is significant for both mass and age at pupation in the ANOVAs. The discriminant function coefficients for mass and age are both positive; the coefficient for mass is about twice as large as that for age (S54 Table). This interaction accounts for 11 % of the variance in mass across the experiment; the total explained variance for mass across the experiment is 83% (S55 Table). The masses of the female pupae are larger in all treatments than the masses of males in any treatment; sex is more important that the other factors for the mass at pupation. For males and females, mass at pupation is greater on 3 mg than on the average of 1 mg and 2 mg. Males grow larger on the day 8 delay at the lower food level, but females grow larger on the day 6 delay at the lower food level. Males grow to the same size on the higher food level regardless of the delay, but females grow larger on the day 6 delay. Females grow as expected based on the *a priori* predictions and the observed main effects. Males do not. There appear to be two different effects of the food 2 x delay x sex interaction on male mass: 1) at the low food level, the males grow larger in the test tubes with the longer delay; 2) at the higher food level, the males grow to be the same size regardless of the delay.

S56 Table. Means (SE) for mass (mg) for the interaction food 2 x delay x sex.

| Second food input (Food 2) | Delay (day 6 or day 8) | Mass (SE) of males (mg) | Mass (SE) of females (mg) |
| --- | --- | --- | --- |
| 1 mg + 2 mg | day 6 | 1.80 (0.21) | 2.56 (0.67) |
|  | day 8 | 1.98 (0.42) | 2.44 (0.49) |
| 3 mg | day 6 | 2.27 (0.27) | 3.74 (0.18) |
|  | day 8 | 2.26 (0.26) | 3.49 (0.28) |

S57 Table shows the means and standard errors for age for males and females by food input and delay treatments for the most significant interaction contrast, food 2 x delay x sex. This interaction accounts for 8% of the variance in the experiment; the total explained variance for age across the experiment is 64%. The age in the table is the time after the second food input; the numerical difference due to the delay has been removed so the time difference reflects the growth after the food input for both delays. Females grow for longer than males; sex again seems to be more important that the other two factors. The longer delay (day 8) results in longer time to pupation for both sexes. The second food input has the smallest overall effect on the ages at pupation, but affects males and females differently. Both males and females pupate earliest in the test tubes with the highest food level (3 mg) and the shorter delay (day 6). The other 3 treatments for females pupate at similar times (5.26 days to 5.50 days) with the next earliest being at the low food level and day 6 delay, and the latest being also at the low food level with the day 8 delay. The larger amount of food in the second input has a positive effect on the females (earlier pupation), but a much greater effect on the day 6 delayed females than on the day 8 delayed ones. Males respond to the food input and the delays differently. For males the time to pupation is similar for the 6 day delay at both food inputs, and for the 8 day delay at both food inputs, so the delay appears to be more important than the amount of food.

S57 Table. Means (SE) for age (days) for the interaction food 2 x delay x sex.

| Second food input (Food 2) | Delay (day 6 or day 8) | Age (SE) of males (days) | Age (SE) of females (days) |
| --- | --- | --- | --- |
| 1 mg + 2 mg | day 6 | 3.35 (0.21) | 5.26 (0.90) |
|  | day 8 | 4.03 (0.20) | 5.50 (0.24) |
| 3 mg | day 6 | 3.30 (0.82) | 4.40 (0.55) |
|  | day 8 | 4.20 (1.10) | 5.40 (0.89) |

S58 Table shows the estimated growth rates for males and females for this interaction (food 2 x delay x sex). These growth rates are calculated using only the period after the second food input, so they over estimate the actual growth rate, but they are comparable to one another despite the differences in the initial delay. These estimated growth rates are not comparable to those in the first experiment. This estimated growth rate combines the mass and age variables into a single biologically meaningful number. The estimated growth rates of males are higher at the shorter delay at both food inputs, but the highest rate at the low food input is the same as the lowest rate at the high food input. The estimated growth rates for the females are also higher at the shorter delay at both food inputs, but the rates at the high food input are much greater than at the low food input. For males the period of starvation is relatively more important than the amount of food, while for females the food appears to have a greater relative effect. For both males and females, the combination of the shorter delay with the large food input results in the best outcome (growth rate, mass and age at pupation). At the longer delay with the large food input, males grow to the same size as in the best outcome, but take longer (almost a day longer) and females grow larger than the females at the low food input, but also take almost a day longer. That the males at the high food input pupate at the same size suggests that there is an optimal largest size for males (that may also depend on the larval history, physiology and environment). That the females with less food pupate at the same time as the high input females with the long delay suggests that females have an optimal maximum duration of the larval period (that may depend on the same parameters as the males).

S58 Table. Means (SE) for estimated growth rates (mg/day) for the interaction food 2 x delay x sex.

| Second food input (Food 2) | Delay (day 6 or day 8) | Estimated growth rate (SE) of males (mg/day) | Estimated growth rate (SE) of females (mg/day) |
| --- | --- | --- | --- |
| 1 mg + 2 mg | day 6 | 0.54 (0.07) | 0.49 (0.15) |
|  | day 8 | 0.49 (0.11) | 0.44 (0.09) |
| 3 mg | day 6 | 0.69 (0.19) | 0.85 (0.11) |
|  | day 8 | 0.54 (0.15) | 0.65 (0.12) |

The delay until the second input of food amounts to a period of starvation for the larvae. The 1^st^ instar larvae probably have lots of food relative to their demand for it, as do the 2^nd^ instars, but the larvae are probably low on food in the 3^rd^ and 4^th^ instars, and few pupate on the initial 1 mg of food (only 10 of 150 larvae). These very low food pupae are all male and small (mean mass 1.06 mg, standard deviation 0.21); they pupate on day 7 or day 8 of the experiment (this would be day -1 or 0 once transformed by subtracting the delay). It is an indication of the difference in the biology of the male and female mosquitoes that males pupate at a small size after a period of starvation while females do not. Because the timing of the beginning of the starvation period is unknown, the total length of the starvation period is also unknown, but the day 8 delay treatment amounts to 2 more days of starvation compared to the day 6 delay treatment.

This experiment measures the response to a period of starvation followed by a second food input of various amounts. Males and females alter both the size at which they pupate and the amount of time that they continue to feed and grow. At the low food input, males grow larger after the longer delay, but take longer to do so. At the high food input they grow to the same size regardless of the delay, but take longer after the longer delay. For males, the longer delay results in a longer period of growth after the subsequent food input regardless of the size of the input. Females grow largest after the shorter delay combined with the high food input and take the least time to grow that large. At the low food input females also grow larger after the shorter delay and also take less time to pupate, but they do not grow as large or as fast as the females with the larger second food input.

Male larvae in previous experiments actively filter particles until they pupate; females switch from active filtering to retention at low food levels. This interaction suggests that males respond to starvation by increasing their larval period. They grow larger (or grow to a maximum size dependent on the factors mentioned previously), despite taking more time. This is different from the two reasons observed in prior experiments for males to delay pupation: abundant food resulting in larger size, and scarce food resulting in late pupation at a smaller size. In this experiment (as shown by this interaction), males respond to the same second food input (1 mg, 2 mg, or 3 mg dry weight of yeast) differently depending on the length of the period of starvation. The response to the abundance of food depends on the duration of the period of scarcity.

**The food 1 x delay x sex interaction**

S59 Table shows the means and standard errors for mass (mg) for males and females for the 3-way interaction food 1 x delay x sex. This interaction accounts for only 3% of the explained variance, but the residual 2-way interaction food 1 x delay explains another 7% of the variance. The 3-way interactions have to be understood before the residual 2-way interactions can be interpreted. Both males and females grow larger on the 2 mg food input than on the 1 mg food input. For this interaction, food input amount appears to be more important than delay for both males and females. There is not much effect of the delay at the 1 mg input for either males or females and a larger effect of delay at the 2 mg input for both males and females. Males grow larger on the 2 mg input with the day 8 delay, but females grow larger on the 2 mg input with the day 6 delay. The males in the 2 mg input, day 8 delay test tubes grow to the same size that the largest males in the previous interaction grow to, further suggesting that this is a maximum size for these conditions. Females grow larger on the 2 mg input than the 1 mg input in this interaction, but not as large as the 3 mg input in the previous interaction. The effect of the delay is large at the 2 mg food input and almost nothing at the 1 mg input; it is almost the same at the 2 mg food input as at the 3 mg food input in the previous interaction. For this interaction the size of the food input positively affects the growth of the larvae, but the 2 mg food input also increases the effect of the delay, and males grow larger on the day 8 delay while females grow larger on the day 6 delay. The interaction reflects the different joint effects of food input amount and delay on the two sexes.

S59 Table. Means (SE) for mass (mg) for the interaction food 1 x delay x sex.

| Second food input (Food 1) | Delay (day 6 or day 8) | Mass (SE) of males (mg) | Mass (SE) of females (mg) |
| --- | --- | --- | --- |
| 1 mg | day 6 | 1.65 (0.28) | 2.09 (0.35) |
|  | day 8 | 1.68 (0.27) | 2.10 (0.28) |
| 2 mg | day 6 | 1.95 (0.44) | 3.03 (0.23) |
|  | day 8 | 2.27 (0.12) | 2.79 (0.17) |

Since the largest males in S59 Table are the same size as the largest males in S56 Table across different second food input amounts, it appears that the optimal maximum size of males in this experiment is set before the second food input is added (earliest on day 6). Otherwise, the maximum size would reflect the different food inputs for males as it does for females. This is another difference between males and females.

S60 Table shows the means and standard errors for age at pupation (days) for males and females for the interaction food 1 x delay x sex. This interaction is not significant in the ANOVA, but the ages are significantly affected by the main factors and by 2-way interactions involving the factors. The ages are presented so that they can be combined with the mass values in S59 Table to estimate the growth rates of males and females (S61 Table).

S60 Table. Means (SE) for age (days) for the interaction food 1 x delay x sex.

| Second food input (Food 1) | Delay (day 6 or day 8) | Age (SE) of males (days) | Age (SE) of females (days) |
| --- | --- | --- | --- |
| 1 mg | day 6 | 3.50 (1.08) | 5.90 (1.37) |
|  | day 8 | 4.17 (0.41) | 5.67 (1.03) |
| 2 mg | day 6 | 3.20 (1.14) | 4.63 (0.92) |
|  | day 8 | 3.89 (0.78) | 5.33 (0.82) |

S61 Table shows the estimated growth rates for males and females for the interaction food 1 x delay x sex. These estimated growth rates are not comparable to those in the first experiment. The highest estimated growth rates are for the treatment with the 2 mg food input and the day 6 delay. These test tubes produce the largest mass and the earliest age at pupation for females across this contrast, so it appears to be the optimal treatment. However, the males in this treatment are only the second largest, the high estimated growth rate is due to the earliest age at pupation across the males. The largest males are in the 2 mg input and day 8 delay treatment. They grow for more than a half day longer and get 0.33 mg larger. The estimated growth rate for these test tubes is higher than the corresponding one for the females. The females in this treatment grow larger than the males (0.52 mg larger), but they take much longer (1.44 days longer), so the estimated growth rate is lower for the females than for the males. This relationship is also true for males and females at the 1 mg food input. Females grow larger than males (0.42 mg to 0.44 mg larger), but take much longer (1.50 days to 2.40 days longer) resulting in smaller estimated growth rates for the females than for the males. Within this interaction, the test tubes with the 2 mg food input and the shorter delay provide the optimal growing conditions for the larvae (growth rate, mass and age at pupation for females, growth rate and age at pupation for males). Males grow larger at both food levels with the day 8 delay, but take longer to do it. For both males and females there seems to be a target size relative to the food input that increases as the food input increases. It reaches a cap for males at about 2.27 mg. The size varies by food input, delay and sex and by the 3-way interaction between these factors. The time that the larva takes to reach that target size also varies by food input, delay and sex (but not by the 3-way interaction between them).

S61 Table. Means (SE) for estimated growth rates (mg/day) for the interaction food 1 x delay x sex.

| Second food input (Food 1) | Delay (day 6 or day 8) | Estimated growth rate (SE) of males (mg/day) | Estimated growth rate (SE) of females (mg/day) |
| --- | --- | --- | --- |
| 1 mg | day 6 | 0.47 (0.17) | 0.35 (0.10) |
|  | day 8 | 0.40 (0.08) | 0.37 (0.08) |
| 2 mg | day 6 | 0.61 (0.26) | 0.66 (0.14) |
|  | day 8 | 0.58 (0.12) | 0.52 (0.09) |

**Summary of the 3-way interactions together**

Combining the results for these two interactions shows differences between males and females as measured by the mass and age at pupation. Males increase in size in response to the second food input (1 mg input: 1.67 mg; 2 mg input: 2.10 mg; 3 mg input: 2.27 mg); they reach a maximum size of about 2.27 mg in this experiment and then pupate. Males take longer to pupate on the day 8 delay than the day 6 delay, but the latest ages at pupation are at the 1 mg and 3 mg food inputs. The ages at pupation for the day 6 delay treatments range from 3.20 days to 3.50 days (a range of 0.30 days). The ages at pupation for the day 8 delay treatments range from 3.89 days to 4.20 days (a range of 0.31 days). The difference between the male age at pupation for the day 6 and day 8 delay treatments across food inputs ranges from 0.67 days to 0.90 days, with the largest difference at the 3 mg food input. The additional two days of starvation causes males to delay pupation in order to grow larger, and possibly to improve their physiological state because the extra time does not show up in mass. A heuristic 3D graph of male masses and ages at pupation is in S37 Fig. A similar heuristic 3D graph of female masses and ages at pupation is presented alongside the graph for males.

Females increase in size in response to the second food input (1 mg input: 2.10 mg; 2 mg input: 2.91 mg; 3 mg input: 3.62 mg); each additional 1 mg increment of food results in larger masses at pupation. The mass of females receiving the 1 mg second food input is approximately 2 mg (2.10 mg wet weight). This corresponds to 2 mg dry weight of yeast (total food input including the initial 1 mg on day 0 and the second 1 mg on day 6 or day 8). The mass of females receiving the 2 mg second food input is approximately 3 mg (2.93 mg wet weight). The mass of females receiving the 3 mg second food input is less than 4 mg (3.62 mg wet weight). Each incremental 1 mg of yeast is less valuable than the previous one; as the amount of the second food input increases, the effect of the incremental food on the pupal mass is smaller. The effect of the delay also changes as the food input increases. At the 1 mg second food input, there is little effect of delay on the pupal mass of females. At the 2 mg and 3 mg food inputs, the day 8 delay treatment females are 0.24 mg to 0.25 mg smaller than the day 6 treatment females. There is also an effect of delay and food on age at pupation. The day 6 delay treatment females pupate earlier as the size of the second food input increases. The age at pupation for the 1 mg food input, day 6 delay females is 5.90 days. This is both the smallest mass at pupation and the latest age at pupation. The corresponding ages at pupation for 2 mg and 3 mg food inputs are 4.63 days and 4.40 days respectively. The incremental food reduces the age at pupation as it increases the mass at pupation, and the effect of the 1 mg of food is less with each additional increment. In contrast, the age at pupation for the day 8 delay treatment females doesn’t show a regular pattern in response to the incremental additions of food (5.67 days, 5.33 days, and 5.40 days for 1 mg, 2 mg, and 3 mg second food inputs, respectively). The additional two days of starvation change the way that females respond to additional food. They still grow larger on the extra food, but they don’t grow as large and they seem to grow for an amount of time that is not affected by the size of the food input. A heuristic graph of male and female masses at pupation is plotted against the total food input in each test tube (dry weight of yeast) in S38 Fig.

If we were to model the growth of males and females in response to these two independent factors, we would need different models for each sex, and the models would resemble computer programs with conditional logic, rather than mathematical formulas with smooth functions.

**The 2-way interactions**

The 2-way interactions describe the residual effects of the pairs of factors after the 3-way interactions have been addressed (S56 Table-S61 Table). The 2-way interaction with the largest R squared value (0.34) in the MANOVA is food 1 x delay. It is the only 2-way interaction that is significant for both mass and age at pupation in the ANOVAs. The discriminant function coefficients for mass and age are both positive; the coefficient for mass is about twice as large as that for age. The r squared values from the ANOVAs for mass and age are 0.07 and 0.06, accounting for 7% and 6% of the explained variance across the experiment, respectively. This interaction describes the effect of the factors, food 1 and delay, on the growth of mosquito larvae regardless of their sex. S62 Table shows the means and standard errors for the mass at pupation for the contrast food 1 x delay. The expected values for each treatment, based on the main effects, and the differences between the observed and expected values, are also shown. The expected values are projected based on the means of the main effects (see S53 Table) and presented in S62 Table- S69 Table. The standard errors are the standard errors for the expected values of mass and age across this experiment. The standard errors for the difference between observed and expected values are calculated as usual for the difference between two means.

**The food 1 x delay interaction**

S62 Table. Means (SE), expected values and differences for mass (mg) for the interaction food 1 x delay.

| Second food input | Delay | Mass (SE) (mg) | Expected value of mass (SE) (mg) | Difference between observed and expected values (SE) (mg) |
| --- | --- | --- | --- | --- |
| 1 mg | day 6 | 1.87 (0.31) | 2.15 (0.68) | -0.28 (0.37) |
|  | day 8 | 1.89 (0.29) | 2.14 (0.68) | -0.25 (0.37) |
| 2 mg | day 6 | 2.49 (0.77) | 2.48 (0.68) | 0.01 (0.51) |
|  | day 8 | 2.53 (0.36) | 2.47 (0.68) | 0.06 (0.39) |

The masses of the larvae (combined males and females) are lower at the 1 mg food input than at the 2 mg input. There is not a large effect of delay, but the masses of pupae from day 6 delay are consistently smaller than those from the day 8 delay. The observed values of the masses are lower than the expected values at the low food input and slightly larger at the higher input. The expected values for the day 6 delay are slightly larger than for the day 8 delay, in contrast to the observed values. Food input is more important than delay for the mass variables, but the delay has an effect opposite that expected. The smallest masses should be in the test tubes with low food input and day 8 delay, but they are in those with the low food input and day 6 delay. The largest masses should be in the test tubes with the higher food input and the day 6 delay, but they are in those with the high food input and the day 8 delay.

S63 Table shows the means and standard errors for the age at pupation for the food 1 x delay interaction. The expected values for each treatment, based on the main effects, and the differences between the observed and expected values, are also shown. There is no 3-way interaction for the age variable in the ANOVA for food 1 x delay x sex, so this is the highest order (and only) interaction for age, for the factors food 1 and delay. The larvae take longer to pupate at the 1 mg food input than at the 2 mg food input, and at the day 8 delay than at the day 6 delay. The 2 mg food input and day 6 delay results in the earliest pupation across the interaction. The expected values show the greater relative effect of delay on the age at pupation; the larvae should pupate earlier with the 1 mg food input and day 6 delay, than with the 2 mg input and the day 8 delay. This is not observed; the larval growth is more affected by the food input amount than by the delay. Three of the treatments result in observed values larger than the expected ones, but the best observed outcome is much earlier than the expected value (2 mg food input and day 6 delay). The asymmetry that causes this interaction is different from the source of the interaction for mass.

S63 Table. Means (SE), expected values and differences for age (days) for the interaction food 1 x delay.

| Second food input | Delay | Age (SE) (days) | Expected value of age (SE) (days) | Difference between observed and expected values (SE) (mg) |
| --- | --- | --- | --- | --- |
| 1 mg | day 6 | 4.70 (1.70) | 4.47 (0.93) | 0.23 (0.97) |
|  | day 8 | 4.92 (1.06) | 4.79 (0.93) | 0.13 (0.71) |
| 2 mg | day 6 | 3.91 (1.01) | 4.21 (0.93) | -0.30 (0.69) |
|  | day 8 | 4.61 (1.02) | 4.51 (0.93) | 0.10 (0.69) |

The optimal treatment for both mass and age at pupation across this interaction is the 2 mg food level and the day 6 delay; the mass is the second largest, but the age at pupation is much earlier. Both day 8 delay treatments grow slightly larger than their day 6 counterparts, but take longer to do so. The additional 2 days of starvation appears to change the parameters that determine when and at what size the larvae pupate.

**The food 2 x delay interaction**

S64 Table shows the means and standard errors for the mass at pupation for the contrast food 2 x delay. The expected values for each treatment, based on the main effects, and the differences between the observed and expected values, are also shown. The MANOVA R squared value for this contrast is the lowest across the experiment (0.08). The MANOVA coefficients for this interaction are large and positive for the mass variable and negative and near zero for the age variable. The ANOVA r squared value for the mass is 0.01; the ANOVA for the age is not significant. The masses are larger with the 3 mg food input than with the lower food input (the average of 1 mg and 2 mg). The effect of the delay on mass is smaller than that of the food input and differs depending on the food input amount. The mass at pupation is highest at the 3 mg food input and day 6 delay and lowest at the lower food input and day 6 delay. The expected values of mass are also more affected by the food input than by the delay, but the expected value of mass at the day 6 delay is slightly larger than that of the day 8 delay at both food inputs. The span of the expected values is 0.38 mg compared to the 0.82 mg span of the observed values. Both lower food inputs are lower than expected and both 3 mg food inputs are higher. The largest mass also has the greatest difference from the expected value and the smallest mass has the greatest negative difference, so the best and worst outcomes across this experiment are the source of the interaction.

S64 Table. Means (SE), expected values and differences for mass (mg) for the interaction food 2 x delay.

| Second food input | Delay | Mass (SE) (mg) | Expected value of mass (SE) (mg) | Difference between observed and expected values (SE) (mg) |
| --- | --- | --- | --- | --- |
| 1 mg + 2 mg | day 6 | 2.18 (0.60) | 2.32 (0.68) | -0.14 (0.45) |
|  | day 8 | 2.21 (0.46) | 2.31 (0.68) | -0.10 (0.41) |
| 3 mg | day 6 | 3.00 (1.05) | 2.69 (0.68) | 0.32 (0.62) |
|  | day 8 | 2.88 (0.87) | 2.67 (0.68) | 0.20 (0.55) |

S65 Table shows the means and standard errors for the age at pupation for the contrast food 2 x delay. This interaction is not significant in the ANOVA. The treatment associated with the largest mass (3 mg food input, day 6 delay) is also associated with the earliest age at pupation. The larvae in the test tubes with the day 8 delay take almost the same amount of time to pupate, but pupate at different sizes depending of the amount of food. The smallest pupae took more time to pupate than the largest (both with day 6 delay), but less time than either day 8 delay treatments.

S65 Table. Means (SE) for age (days) for the interaction food 2 x delay (not significant in the ANOVA).

| Second food input | Delay | Age (SE) (days) |
| --- | --- | --- |
| 1 mg + 2 mg | day 6 | 4.31 (1.23) |
|  | day 8 | 4.76 (0.87) |
| 3 mg | day 6 | 3.85 (0.78) |
|  | day 8 | 4.80 (0.85) |

**Summary of the food 1 x delay and food 2 x delay interactions**

These two interactions (S62 Table-S65 Table) describe the growth of larvae at 3 food input amounts and 2 different periods of starvation. Sex is not a factor in these interactions, so the relationship between the length of the period of starvation and the amount of food input at the end of that period is independent of sex. For both delay treatments, the second milligram of food results in a larger incremental increase in size than the third milligram. For the shorter delay, the incremental difference due to the third milligram of food input is 0.51 mg compared to that due to the second milligram, 0.62 mg. For the longer delay, an extra two days of starvation, the incremental difference due to the third milligram of food input is 0.35 mg compared to that due to the second milligram, 0.64 mg. The longer period of starvation reduces the value of the last milligram of food. The expected values of the masses are lower and the differences between the expected values (respective to the comparisons above) are also smaller. The masses of the pupae are much lower than the expected values at the 1 mg food input, close to the expected value at the 2 mg food input, but divergent and much higher than the expected values at the 3 mg food input.

Comparing the growth across the two delay treatments, the day 8 delay grows larger than the day 6 delay except at the highest (3 mg) food input. The expected values project that the day 8 delay treatment should result in smaller pupae than the day 6 delay treatment at all food inputs.

Age at pupation is significantly affected by the interaction of food input amount and delay at the lower input amounts (1 mg and 2 mg), but not at the highest input amount (3 mg). Comparing the incremental difference in age due to the second milligram of food, the larvae pupate earlier at both delays, but the effect is much greater (more than double) with the day 6 delay than the day 8 delay. The incremental difference due to the third milligram of food is smaller for the day 6 delay and the third milligram of food causes the larvae to grow for a longer time before pupation with the day 8 delay. Comparing the age at pupation across the two delay treatments, the day 8 delay takes longer than the day 6 delay, and the difference between the two treatments increases as the amount of the food input increases.

Because males and females are combined in this interaction and their growth rates are expected to be different, it doesn’t make sense to calculate estimated growth rates for these treatment combinations. The 1 mg food input results in pupae that weigh about 1.88 mg on 2 mg dry weight of yeast. This is less than the expected value based on the main effects. The pupae in the day 8 delay treatment are slightly larger than those in the day 6 delay treatment, in contrast to the expected values. The 2 mg food input results in pupae of about 2.51 mg on 3 mg dry weight of yeast. This is slightly more than the expected value. The day 8 delay treatment is slightly larger than the day 6 delay treatment, also in contrast to the expected values. The 3 mg food input results in pupae that weigh about 2.94 mg on 4 mg dry weight of yeast. This is higher than the expected value and the day 6 delay treatment is much larger than the day 8 delay treatment, also in contrast to the expected values, but in a different direction than the lower food inputs. Each increment of food results in larger pupae, but each additional increment increases the size of the pupae by a smaller amount. The age at pupation is only significant for the food 1 contrast, the two lower food inputs. The larvae that receive the 1 mg second food input pupate later than those that receive the 2 mg second food input. The larvae in the day 6 delay pupate earlier than those in the day 8 delay treatment in both food inputs. The age at pupation drops much more with the 2 mg second food input in the day 6 delay treatment, so the effect of the additional food on age at pupation changes depending on the period of starvation (delay treatment). The age at pupation decreases again in the 3 mg second food input with the day 6 delay treatment, but increases slightly in the 3 mg food input with the day 8 delay.

The optimal treatment across these interactions is the 3 mg food input with the day 6 delay. It is the most food and the shorter period of starvation. The next largest mass is in the treatment with the 3 mg food input and the day 8 delay, but it is associated with the second latest age at pupation. At the two lower food inputs (1 mg and 2 mg) the sizes are similar at each food level, but the day 8 pupae are slightly larger than the day 6 pupae. The ages at pupation at the 1 mg food input are later than either of the 2 mg food inputs, but the day 6 treatments pupate earlier than the day 8 treatments. For both mass and age the food input amount is more important than the delay, but at both food input amounts (1 mg and 2 mg) the mass is larger and the age at pupation is later for the day 8 delay. At the highest food input (3 mg) the masses are both larger than the lower food input treatments, and the day 6 delay pupates earlier than the lower food input treatments. However, the 3 mg food input, day 8 delay pupae are smaller than the day 6 pupae and pupate much later. The extra two days of starvation change the way the larvae use the additional food, affecting both the final mass and the timing of pupation. The 3 mg food input, day 8 delay larvae grow almost 1 mg larger than the 1 mg food input, day 8 delay treatment, and take almost as long. The 2 mg food input, day 8 treatment grows 0.62 mg larger than the 1 mg food input, day 8 treatment, in less time than either the 1 mg or 3 mg second food input treatments. This suggests that the larvae (both sexes) use the first milligram of food primarily for growth in mass, and the second milligram allows them to grow larger and faster, but the third milligram allows them to replenish the physiological resources that were depleted during the extra two days of starvation.

The pattern across these two interactions suggests underlying changes in the way that food is being used depending on the period of starvation. At the 1 mg food input, there is a small difference (0.02 mg) in the masses at pupation and a small difference (0.22 day) in the age at pupation. At the 2 mg food input, the larvae are larger than at the low food input and take less time to pupate. There is still a small difference (0.04 mg) in the masses at pupation, but a much larger difference (0.70 day) in the ages at the different delays. The incremental amount of food in the second treatment allows the day 6 delay larvae to grow both faster and larger than the low food treatments. The day 8 delay larvae take longer to pupate, but grow slightly larger than the day 6 ones. This suggests that the day 8 delay larvae are rebuilding nutritional reserves depleted during the extra two days of starvation they endured. This could also explain the 3 mg results. The day 6 larvae grow much larger and faster on the abundant food, and pupate at the largest mass and at the earliest age. The day 8 larvae grow larger than any of the lower food input treatments, but take longer than most of those treatments, probably to replace reserves depleted during the extra days of starvation. [Because females are much larger than males, this interaction may reflect the effect of the two factors on female masses primarily. Males at the 3 mg food input and at the 2 mg food input with the day 8 delay all reach the same size (2.27 mg) and pupate. The variance due to the difference between the sexes should have been removed mathematically by the 3-way interactions, but the biological differences between the sexes remain nonetheless.]

After removing the effects of the higher order interactions on growth of both males and females, there are residual interactions affecting all larvae due to the incremental food inputs and the period of starvation (the delay treatments). The first increment of 1 mg (second food input) appears to be required for pupation (at least in females). The second increment appears to contribute primarily to increasing mass, while the third increment appears to allow larvae to replenish physiological reserves, particularly after the longer period of starvation.

**The food 1 x sex and food 2 x sex interactions**

The final significant 2-way interactions are food 1 x sex and food 2 x sex. They both have MANOVA R squared values of 0.17. The MANOVA discriminant function coefficients are greater for age than for mass in both contrasts. The coefficients are both positive for food 1 x sex, but of opposite signs for food 2 x sex; this likely indicates a difference in the effects of the three food input amounts across the sexes. The r squared values for these interactions are both 0.07 for the age at pupation in the ANOVA. Neither interaction is significant for the mass at pupation in that ANOVA. There is no 3-way interaction in the ANOVA for the age variable for food 1 x delay x sex, so this is the highest order (and only) interaction for food 1 x sex for age at pupation. The food 2 x sex interaction represents the residual effect after the 3-way interaction, food 2 x delay x sex is removed.

**The food 1 x sex interaction**

S66 Table shows the means and standard errors for the age at pupation for the interaction food 1 x sex. The expected values for each treatment, based on the main effects, and the differences between the observed and expected values, are also shown. Female larvae take longer to pupate than male larvae; in both food input treatments males pupate earlier than females in either food treatment, so sex is more important for age at pupation than food input amount. Within each sex, the larvae at the 2 mg food input pupate earlier than those at the 1 mg food input. Males pupate earlier than projected based on the main effects and females pupate later than projected. The difference between the observed and expected values for males is slightly larger at the 2 mg food input, but for females the difference is much greater at the 1 mg food input. The source of this interaction is likely the asymmetry between the males and females at the two food inputs.

S66 Table. Means (SE), expected values and differences for age (days) for the interaction food 1 x sex.

| Second food input | Sex | Age (SE) (days) | Expected value of age (SE) (days) | Difference between observed and expected values (SE) (days) |
| --- | --- | --- | --- | --- |
| 1 mg | M | 3.83 (0.47) | 4.22 (0.93) | -0.39 (0.52) |
|  | F | 5.78 (0.16) | 5.01 (0.93) | 0.77 (0.47) |
| 2 mg | M | 3.54 (0.49) | 3.98 (0.93) | -0.44 (0.53) |
|  | F | 4.98 (0.50) | 4.72 (0.93) | 0.26 (0.53) |

S67 Table shows the means and standard errors for the mass at pupation for the interaction food 1 x sex. This interaction is not significant in the ANOVA, but the mass is affected by the main effects and the 3-way interaction. Females are larger than males at both food levels, but the females at the 1 mg food input are smaller than the males at the 2 mg food input. The difference between the size of the males and females is also greater at the high food level. The reason for presenting the mass data is to calculate the estimated growth rate in S68 Table.

S67 Table. Means (SE) for mass (mg) for the interaction food 1 x sex (not significant in the ANOVA).

| Second food input | Sex | Mass (SE) (mg) |
| --- | --- | --- |
| 1 mg | M | 1.67 (0.02) |
|  | F | 2.09 (0.01) |
| 2 mg | M | 2.11 (0.23) |
|  | F | 2.91(0.17) |

S68 Table shows the means and standard errors for the estimated growth rates for males and females in the interaction food 1 x sex. This estimate of growth rate uses only the time after the addition of the second food input, so it overestimates the growth rate. This technique enables a fair comparison across the two delay treatments (not a factor in this interaction), but these estimated growth rates are not comparable to those in the first experiment. Males grow faster than females on both food inputs, but do relatively better than females at the 1 mg food input. Females grow almost as fast as males on the 2 mg food input. Males and females grow larger and pupate earlier at the 2 mg food input compared to the 1 mg; the age at pupation is the significant variable.

S68 Table. Means (SE) for estimated growth rates (mg/day) for the interaction food 1 x sex.

| Second food input | Sex | Estimated growth rate (mg/day) |
| --- | --- | --- |
| 1 mg | M | 0.43 (0.05) |
|  | F | 0.36 (0.01) |
| 2 mg | M | 0.60 (0.10) |
|  | F | 0.58 (0.07) |

**The food 2 x sex interaction**

S69 Table shows the means and standard errors for the age at pupation for the residual interaction food 2 x sex. The expected values for each treatment, based on the main effects, and the differences between the observed and expected values, are also shown. Female larvae take longer to pupate than male larvae; in both food input treatments males pupate earlier than females in either food treatment, so sex is more important for age at pupation than food input amount. Males pupate earlier on the lower food input (1 mg + 2 mg) than the 3 mg food input, but females pupate earlier on the 3 mg food input than on the lower one. Males pupate earlier than projected based on the main effects and females pupate later than projected. The differences between the observed and expected values for both males and females are larger at the lower food input (1 mg + 2 mg) than the 3 mg food input; males pupate much earlier than projected at the lower food input and females pupate much later than projected. There is a residual effect of the size on the food input on the timing of pupation for both males and females after the 3-way interaction has been removed.

S69 Table. Means (SE), expected values and differences for age (days) for the interaction food 2 x sex.

| Second food input | Sex | Age (SE) (days) | Expected value of age (SE) (days) | Difference between observed and expected values (SE) (days) |
| --- | --- | --- | --- | --- |
| 1 mg + 2 mg | M | 3.69 (0.43) | 4.10 (0.93) | -0.41 (0.51) |
|  | F | 5.38 (0.56) | 4.87 (0.93) | 0.51 (0.54) |
| 3 mg | M | 3.75 (0.64) | 4.01 (0.93) | -0.26 (0.56) |
|  | F | 4.90 (0.71) | 4.75 (0.93) | 0.15 (0.59) |

S70 Table shows the means and standard errors for the mass at pupation for the interaction food 2 x sex. This interaction is not significant in the ANOVA, but the mass is affected by the main effects and 3-way interactions. Females are larger than males at both food levels, so sex is more important than food input for this interaction. The difference between the size of the males and females is also greater at the 3 mg food level. The size of the males in the 3 mg food input treatment appears to be almost uniform at 2.26 mg, as noted in the interaction food 2 x delay x sex. A maximum optimal size for males would account for the larger difference between the males and females at the 3 mg food input. The reason for presenting the mass data is to calculate the estimated growth rates in S60 Table.

S70 Table. Means (SE) for mass (mg) for the interaction food 2 x sex (not significant in the ANOVA).

| Second food input | Sex | Mass (SE) (mg) |
| --- | --- | --- |
| 1 mg + 2 mg | M | 1.89 (0.29) |
|  | F | 2.50 (0.48) |
| 3 mg | M | 2.26 (0.00) |
|  | F | 3.62 (0.18) |

S71 Table shows the means and standard errors for the estimated growth rates for males and females in the interaction food 2 x sex. This estimate of growth rate uses only the time after the addition of the second food input, so it overestimates the growth rate. This technique enables a fair comparison across the two delay treatments (not a factor in this interaction). These estimated growth rates are not comparable to those in the first experiment. Males grow faster than females on the lower food input, but females grow faster than males at the 3 mg food input. The lower food input reflects the interaction shown in S68 Table (food 1 x sex). The males in the 3 mg food input grow as fast as the males in the 2 mg food input; they grow larger and take longer to pupate than the males at the 2 mg food input, but the similar growth rates suggest they are not limited by food at the 2 mg input (or higher). Females grow much larger on the 3 mg input and pupate sooner than females on the 2 mg input resulting in a higher growth rate and suggesting that females are food limited at the 2 mg second food input, and possibly at the 3 mg second food input.

S71 Table. Means (SE) for estimated growth rates (mg/day) for the interaction food 2 x sex.

| Second food input | Sex | Estimated growth rate (mg/day) |
| --- | --- | --- |
| 1 mg + 2 mg | M | 0.51 (0.10) |
|  | F | 0.46 (0.10) |
| 3 mg | M | 0.60 (0.10) |
|  | F | 0.74 (0.10) |

**Summary of the food 1 x sex and food 2 x sex interactions**

These two interactions, food 1 x sex and food 2 x sex, describe the growth of larvae after a period of starvation in response to 3 different amounts of food. There are 3-way interactions between food 2, delay and sex for both mass and age, but the food 1, delay and sex interaction is only significant for mass. There are differences in the signs of the discriminant function coefficients between the food 1 x sex and food 2 x sex interactions, so the relationship between the input amount and sex changes between the two low levels of input (1 mg, 2 mg) and the highest level (3 mg). The 3-way interaction (food 2 x delay x sex, S56 Table-S58 Table) suggests that males pupate at an optimal maximum size at the highest food input, while females grow as large as the food input amount allows (and depending on the delay).

In the 6 tables above (S66 Table-S71 Table), the age, mass and growth rate of males and females are compared across the 3 food input amounts. Delay is not a factor in these tables. The mass is not significantly affected by either interaction, so the changes in mass across the food input amounts are caused by the main effects and the 3-way interaction. Both males and females increase in mass as the food input amount increases, but females are larger and increase more than males with each increment of food. For both males and females, there is a bigger difference between the 1 mg and 2 mg treatments than between the 2 mg and 3 mg treatments, so the second milligram of food is more valuable than the third milligram of food to the mass of the larvae. (see previous interactions)

Females pupate earlier at larger food input amounts. There is a bigger difference between the 1 mg and 2 mg treatments than between the 2 mg and 3 mg treatments, so the second milligram of food is more valuable than the third milligram of food to the age at pupation of females. Males pupate earlier on 2 mg than on 1 mg, but pupate later on 3 mg than on 2 mg. This is likely part of the difference between the food 1 and food 2 interactions with sex. Males pupate earlier than females at each food input amount, but the difference in age at pupation between males and females gets smaller as the food input amount increases.

The estimated growth rate of males increases from 1 mg to 2 mg, but is the same at 2 mg and 3 mg. This is also part of the difference between the food 1 and food 2 interactions with sex. The estimated growth rate of females increases from 1 mg to 2 mg and from 2 mg to 3 mg.

**Summary of the third experiment**

Mortality is higher for single larvae than for multiple larvae in test tubes (50 of 150 larvae died for a survival rate of 67%). There is no obvious pattern for deaths by time period of the experiment, across treatments, or by age at death. Dead larvae were not identified by sex, but the sex ratio of the surviving larvae is 56% male (or 60% male if the 10 larvae that pupated before the second input of food are included). Differential mortality does not seem to be an issue (see the second experiment).

**The 3-way interactions**

The two independent factors, food input amount and delay, and the sex of the larvae affect the mass and age at pupation. The MANOVA and the two ANOVAs are significant for the main effects and 6 of 7 interaction contrasts. The 3-way interactions between food, delay and sex are significant in the MANOVA and ANOVAs, although there are differences in the effects on mass and age.

The interaction, food 2 x delay x sex, is the most important interaction in the MANOVA and for both mass and age in the ANOVAs. Females pupate at larger sizes than males in each treatment combination and the mean mass of females in every treatment is larger than the mean mass of any male in any treatment across this interaction. Sex is more important than either food input or delay for the mass of pupae. The 3 mg food input results in larger females and larger males than the lower food input (the average of 1 mg and 2 mg food inputs). Food input is more important than delay for the mass of pupae. Females grow larger on the day 6 delay than on the day 8 delay at both food levels. Males grow larger on the day 8 delay at the lower food inputs, but grow to the same size at the 3 mg food input. This suggests that males reach a size determined by environmental conditions and pupate as soon as they reach that size, while females grow as large as possible.

The age at pupation in the analysis is the difference between the recorded age and the delay for that treatment; this removes the differences in age due to delay across treatments.  Females pupate later than males in each treatment combination and the mean age at pupation for females in every treatment is later than the mean age at pupation for males in any treatment across this interaction. Sex is more important than either food input or delay for the age at pupation. The day 6 delay treatment pupates earlier than the day 8 delay treatment for both males and females and at both the food inputs. Delay is more important to age at pupation than food input amount. Females pupate earliest in the test tubes with the 3 mg food input and the 6 day delay. The females in the other 3 treatment combinations pupate almost a day later. Males also pupate earliest in the test tubes with the 3 mg food input and the 6 day delay, but the males in the test tubes with the lower food input and the 6 day delay pupate shortly afterwards, followed by the lower food input and the 8 day delay, then the 3 mg food input and the 8 day delay. The similarity of the ages at pupation of the females in the treatments with lower food inputs with the longer delay, and in the 3 mg food input with the longer delay suggests that females may have an optimal maximum duration of the larval period. They grow to different sizes depending on the food input and the delay, but they take almost the same amount of time after the food input to do so. Males postpone pupation at the 3 mg food input and the day 8 delay to grow to the same size as the males in the 3 mg food input with the day 6 delay. This suggests that these males are replenishing physiological resources that were depleted during the extra two days of starvation.

The estimated growth rate combines the mass and age at pupation into a single biologically meaningful number that allows comparison of males and females across treatments. The estimated growth rates of females are higher than those of males in both 3 mg food input treatments, but lower than those of males in both lower food input treatments. Males and females grow more slowly on the day 8 delay than the day 6 delay at both food input amounts. The anomalies in the pupal mass of males in this interaction are offset by the later ages at pupation in the day 8 delay treatments. This suggests that both males and females are improving their physiological state at the same time as they are adding mass. The additional 2 days of starvation in the day 8 delay treatments results in the lower growth rates; males take longer to reach the same size (at 3 mg food input) while females grow for longer, but still pupate at a smaller mass than at the 6 day delay.

The other 3-way interaction, food 1 x delay x sex, is not as significant in the MANOVA (smaller R squared value), nor in the ANOVAs. It explains a small amount of variance for the mass at pupation and is not significant for the age at pupation, but there is a significant residual effect for food 1 x delay for both mass and age at pupation, and for food 1 x sex for age at pupation. Females pupate at larger masses than males within each treatment, but females in the 1 mg second food input treatment pupate at smaller masses than the males in the 2 mg second food input, day 8 delay treatment. Both males and females pupate at larger masses on the 2 mg food input. Food input amount (1 mg vs 2 mg) appears to be more important than sex, and also more important than the delay treatment; in the prior interaction, food input amount is less important than either sex or delay. Food is probably limiting the growth of larvae in the test tubes with 1 mg second food input, and may be limiting the growth of larvae in the test tubes with 2 mg second food input. The delay treatment has little effect on the mass of either males or females at the 1 mg food input. The delay treatment has a larger effect at the 2 mg food input, and the effect on males and females is opposite. Males pupate at the largest mass with the 2 mg food input and the day 8 delay, while females pupate at the largest mass with the 2 mg food input and the day 6 delay. Furthermore, the males in the 2 mg food input, day 8 delay test tubes pupate at the same size as the males in the 3 mg food input test tubes. They apparently reach the maximum size as determined by the environment. Since these males receive a different second food input than the 3 mg food input males, the maximum size of males must have been set before the second food input, so before day 6. Because the males in the 2 mg food input, day 8 delay treatment grow as large as males in both 3 mg food input treatments, males are probably not limited by food at the 2 mg food input. Females grow larger on the 3 mg second food input than on the 2 mg food input, so they probably are limited by food at the 2 mg food input.

The age at pupation is not significantly affected by this interaction, but it is affected by the main effects and the other interactions. Females pupate earliest in the 2 mg food input and day 6 delay treatment. This corresponds to the females with the largest masses. The females in the other treatment combinations pupate almost a day later. Males pupate earliest in the 2 mg food input and day 6 delay treatment as well, but these are not the largest males. The males in the day 8 delay treatments take longer to pupate than the males in the day 6 delay treatments, but grow larger. At the 1 mg food input, the males pupate at about the same size, but the day 8 delay males take 0.67 days longer. At the 2 mg food input, the 8 day delay males are larger (0.33 mg larger) but take 0.69 days longer.

The estimated growth rate shows that females grow more slowly than the corresponding males in all treatment combinations except the 2 mg food input with the 6 day delay. Females grow faster at the 2 mg food input than at the 1 mg food input, but they grow fastest at the 2 mg food input with the 6 day delay and slowest at the 1 mg food input with the 6 day delay. These don’t mirror the pupal mass directly, but are affected by the ages at pupation. Males grow faster at the 2 mg food input and at the day 6 delay. This does not reflect the pupal mass directly either. The estimated growth rate indicates that males and females respond differently to the effects of starvation (delay) and the amount of food in the second input. Females grow to almost the same size on the 1 mg second food input with both delay treatments. Males are smaller, but also similar in size to each other on the 1 mg food input with both delay treatments. The difference in growth rate across the two delay treatments for both sexes is due to the age at pupation (not significant in this interaction). At the 2 mg food input, males grow larger with the day 8 delay and take longer to pupate, but grow faster, pupate earlier and at a smaller size with the day 6 delay. Females grow largest and pupate earliest on the 2 mg food input with the day 6 delay, achieving the highest growth rate. Females on the 2 mg food input with the day 8 delay are smaller than the day 6 delay females and pupate later. There are distinct differences between males and females in this interaction. For both sexes, the growth rates support the idea that the larvae are rebuilding physiological reserves that do not show up as mass in the treatments with the 2 mg food input and the day 8 delay.

Combining the results for these two interactions shows differences between males and females as reflected in the mass and age at pupation. Males increase in size in response to the second food input; they reach a maximum size of about 2.27 mg in this experiment and then pupate. Males take longer to pupate on the day 8 delay than the day 6 delay, and the latest ages at pupation are at the 1 mg and 3 mg food inputs. Males in other experiments delay pupation at low food levels to grow larger, and at high food levels to take advantage of the abundant food. These results suggest that the 1 mg second food input at the day 8 delay is less than optimal (and the mass of the males also indicates this). The 3 mg second food input at the 8 day delay may result in an abundance of food so that males delay pupation to grow larger (and the mass of males also indicates this, but the males pupate at the same size as males in the 2 mg second food input with the 8 day delay and the 3 mg second food input with the 6 day delay). The additional two days of starvation causes males to defer pupation in order to grow larger, and possibly to improve their physiological state because the extra time spent feeding does not show up in mass.

Females increase in size in response to the second food input; each additional 1 mg increment of food results in larger masses at pupation. The mass of females receiving the 1 mg second food input is approximately 2 mg (2.10 mg wet weight). This corresponds to 2 mg dry weight of yeast (total food input including the initial 1 mg on day 0 and the second 1 mg on day 6 or day 8). The mass of females receiving the 2 mg second food input is approximately 3 mg (2.93 mg wet weight). The mass of females receiving the 3 mg second food input is less than 4 mg (3.62 mg wet weight). Each incremental 1 mg of yeast is less valuable than the previous one; as the amount of the second food input increases, the effect of the incremental food on the pupal mass is smaller. The effect of the delay also changes as the food input increases. At the 1 mg second food input, there is little effect of delay on the pupal mass of females. At the 2 mg and 3 mg food inputs, the day 8 delay treatment females are 0.24 mg to 0.25 mg smaller than the day 6 treatment females. There is also an effect of delay and food on age at pupation. The day 6 delay treatment females pupate earlier as the size of the second food input increases. The age at pupation for the 1 mg food input, day 6 delay females is 5.90 days. This is both the smallest mass at pupation and the latest age at pupation. The corresponding ages at pupation for 2 mg and 3 mg food inputs are 4.63 days and 4.40 days respectively. The incremental food reduces the age at pupation as it increases the mass at pupation, and the effect of the 1 mg of food on both mass and age of females is less with each additional increment. In contrast, the age at pupation for the day 8 delay treatment females doesn’t change much in response to the incremental additions of food (5.67 days, 5.33 days, and 5.40 days for 1 mg, 2 mg, and 3 mg second food inputs, respectively). The additional two days of starvation change the way that females respond to additional food. They still grow larger on the extra food, but they don’t grow as large and they seem to grow for an amount of time that is not affected by the size of the food input.

If we were to model the growth of males and females in response to these two independent factors, we would need different models for each sex, and the models would resemble computer programs with conditional logic, rather than mathematical formulas with smooth functions.

**The 2-way interactions**

The 2-way interactions reveal the residual effect of the pairs of factors on the mass and age at pupation after the 3-way interactions have been accounted for. The 2-way interaction with the largest R squared value (MANOVA) is food 1 x delay. This interaction shows the residual effect of the size of the second food input and the delay on the growth of larva regardless of sex. The second food input is more important than the delay in this interaction. There are two deviations from the expected values: the pupae are smaller than expected at the 1 mg food input and larger than expected at the 2 mg food input; and the pupae are larger on the day 8 delay than the day 6 delay, but the expected values are larger on the day 6 delay than the day 8 delay. The mass at the 1 mg food input is much smaller than expected while the mass at the 2 mg food input is only a little larger than expected, but the difference due to the delay is larger at the 2 mg food input.

The age at pupation is affected differently by these factors. The second food input is more important than the delay for age at pupation as well as mass at pupation; the larvae pupate earlier on the 2 mg food input than the 1 mg input, and earlier with the day 6 delay than the day 8 delay. The second food input increases the mass and decreases the age at pupation, but the delay treatment increases both the mass and the age. The 2 mg food input with the day 6 delay pupates earlier than the other three treatment combinations. The larvae in these test tubes pupate earlier than the expected value of age at pupation. The larvae in other three treatment combinations pupate later (0.70 days to 1.31 days later) and also later than their expected values.

The 2-way interaction between food 2 and delay compares the two lower second food inputs (1 mg and 2 mg) with the highest second food input (3 mg) crossed with the two delay treatments. This interaction has a small r squared value for mass and is not significant for age at pupation. The size of the second food input is also more important than the delay in this interaction and the lower food inputs are both smaller than their expected values while the 3 mg food input treatments are larger than their expected values. The lower food inputs grow larger on the day 8 delay (see above) but the 3 mg food inputs grow larger on the day 6 delay. The age at pupation is not significantly affected by this interaction; the larvae in the 3 mg food input and day 6 delay pupate much earlier than the other treatments, while those in the 3 mg food input and day 8 delay pupate latest.

These two 2-way interactions describe the residual interaction between the amount of the second food input and the period of starvation (delay treatment) after the effects of the 3-way interactions have been removed. Sex is not a factor so these interactions describe the growth of male and female larvae independently of their sex. For these interactions, food 1 x delay and food 2 x delay, the mass at pupation increases with each increment of food, but increases less with subsequent inputs. The masses at the 1 mg input are below the expected values and not much affected by the delay. The masses at the 2 mg input are close to the expected values and also not much affected by the delay. The masses at the 3 mg input are above the expected values and the day 6 delay treatment grows larger than the day 8 delay treatment. The ages at pupation decrease with increasing food in the day 6 delay treatment, but remain high in the day 8 delay treatment across all three second food inputs. The anomaly appears to be that the larvae in the 1 mg second food input with either delay take longer than expected to pupate despite growing to the smallest sizes across the two interactions. At the two lower food inputs the larvae pupate earlier with the 6 day delay (shorter period of starvation), and they take longer to pupate and grow slightly larger on the day 8 delay. At the 3 mg food input with the day 6 delay, the larvae grow largest and pupate earliest across the two interactions. The larvae in the 3 mg food input with the day 8 delay grow larger than any of the lower food treatments, but take as long as the lower food treatments to grow that large. The value of each increment of food to the mass of the pupa is less in the day 8 delay treatments and becomes relatively less with each added increment. The larvae appear to be using the food for something other than added mass in the day 8 delay treatments. It is possible that the early growth and molts of the larvae determine the largest size of the larvae, but that is similar for both the day 6 delay treatment and the day 8 delay treatment in this experiment. This suggests that the larvae are using some of the food and time to improve their physiological condition rather than building mass in the day 8 delay treatments. Perhaps the larvae (both sexes) use the first milligram of food primarily for growth in mass, and the second milligram allows them to grow larger and faster, but the third milligram allows them to replenish the physiological resources that were depleted during the extra two days of starvation.

The two interactions, food 1 x sex and food 2 x sex, describe the differential effects of the three second food inputs on the age at pupation of males and females. These interactions do not affect the mass at pupation, only the age at pupation. There is a significant 3-way interaction for food 2 x delay x sex for age at pupation, but not one for food 1 x delay x sex, so the food 2 x sex interaction represents the residual after the 3-way interaction is removed. The food 1 x sex interaction is the highest order interaction for these factors for age at pupation.

Males pupate earlier than females in each of the three food input treatments. Males at all three food inputs pupate earlier than any of the females, so sex is more important than food input. Males pupate earlier than their expected value while female pupate later. The difference between the age at pupation and the expected value for males at the three food inputs is similar (0.39 days, 0.44 days, and 0.26 days for 1 mg, 2 mg, and 3 mg second food inputs, respectively). The comparable differences for females decrease in amount (0.77 days, 0.26 days, and 0.15 days for 1 mg, 2 mg, and 3 mg second food inputs, respectively). Male and female ages at pupation deviate from the expected values in different ways.

The food 1 x sex interaction shows that males and females pupate earlier on the 2 mg food input compared to the 1 mg food input, but the difference between the male ages is less than half the difference between the female ages (0.29 days vs 0.80 days). Females benefit more from the 2 mg food input than males do. At the 3 mg food input, females pupate a little earlier than at the 2 mg food input, but males pupate slightly later (see the discussion of the 3-way interactions above). The extra increment of food has opposite effects on the ages at pupation for males and females.

The mass at pupation is not affected by this interaction, but combining the mass and age at pupation into an estimated growth rate for each treatment results in a single biologically meaningful number. These estimated growth rates are not directly comparable to those calculated for experiment 1; these estimated growth rates compare only the growth after the second food input. Males grow faster than females except at the 3 mg food input. The males in the 2 mg food input grow faster than the males at the 1 mg food input. The males in the 3 mg food input grow at the same rate as the males in the 2 mg food input. Males do not appear to be food limited in either the 2 mg or the 3 mg second food inputs. They grow for longer and reach a larger size at the 3 mg food input than at the 2 mg food input (but see the 3-way interactions above). In contrast, females grow faster, larger and pupate earlier with each increment of food. They grow more slowly than males at the 1 mg food input, at almost the same rate at the 2 mg food input, but faster than males at the 3 mg food input. This could be due to the apparent maximum size of males (2.27 mg in this experiment) or to exponential growth processes (the larger size of females) or both. Females appear to grow in response to the amount of food in the second food input; they may still be food limited at the 3 mg food input whereas the males appear to grow equally fast in both the 2 mg and the 3 mg food input, suggesting that even the 2 mg food input is more than sufficient food for them.

The interaction between delay and sex is not significant in the MANOVA or for either mass or age at pupation in the ANOVAs. This means that there is no residual effect of delay x sex on mass or age after the 3-way interactions are removed.
