# Supplementary material for: Competition and growth among Aedes aegypti larvae: Effects of distributing food inputs over time
Source: PLoS One. 2020 Oct 2;15(10):e0234676. doi: 10.1371/journal.pone.0234676 (PMC7531853; doi:10.1371/journal.pone.0234676)
Supplement: Attachment — Submitted filename: Response to reviewers.docx [file pone.0234676.s118.docx]

August 29, 2020

Dear Dr. Hwang, and reviewers,

Thank you for taking the time to read and comprehend my long manuscript. As requested in your letter of July 29, 2020, I have changed the title of the paper so that it matches in the manuscript and the submission forms. The laboratory procedures are submitted to protocols.io and so referenced in the manuscript. The datasets with the original data are included in the supporting information. I have rewritten the methods section in part, and the results section entirely, in order to streamline the text. The original methods and results sections have been moved to supporting information files and I have updated the list of supporting information files. I have reviewed the PLOS ONE style templates and my previous submission to PLOS ONE (11/15/2018), and I believe that the style requirements and file names conform to the guidelines.

I appreciate that there is a great deal of detailed description of results and comparisons among variables in the supporting information; this level of detail is necessary for anyone that would like to replicate the experiment or to challenge my conclusions (see the section on Statistical Reporting in the Submission Guidelines).

Before addressing the “Comments to the Author” specifically, I would like to reiterate that I know how long and tedious it is to work through this body of work and that I appreciate the feedback that I received. It took about 2 years to set up this experiment and more than 2 years to write up the results after the statistical analysis. I do see it as a manuscript about the biology of mosquitoes, not a statistical manuscript. The statistical detail is now primarily contained in the supporting information with the exception of a few references to the statistical tests and some specific comparisons. The focus of the manuscript itself is the biological implications of the results. I believe this improves the readability of the manuscript and I hope the reviewers agree with me.

*Reviewer’s comments:* (none)

*Reviewer’s Responses to Questions*

***Comments to the Author***

1. *Is the manuscript technically sound, and do the data support the conclusions?*

*The manuscript must describe a technically sound piece of scientific research with data that supports the conclusions. Experiments must have been conducted rigorously, with appropriate controls, replication, and sample sizes. The conclusions must be drawn appropriately based on the data presented.*

*Reviewer #1: Partly*

*Reviewer #2: Yes*

Reviewer #1 answered “Partly” to this question. I disagree. I believe that the experiments described in the manuscript were conducted rigorously, with appropriate controls, replication, and sample sizes. I had input from both mosquito researchers and statisticians to design the experiments and I was supervised during the running of the experiments by my post-doctoral advisor and observed by other colleagues. I assert that the high level of explained variance (greater than 80% for the mass variables and greater than 60% for the age variables in the first and third experiments) is an indication that the experiments themselves were well designed and well executed. I discuss the statistical results meticulously in the supporting information (now S3 Text for the first experiment, and S5 Text for the third experiment) and follow standard analytical techniques to determine the numerical explanation for the significance of the results. I then interpret the statistically significant results according to the biology of mosquitoes as understood by me and other researchers (please see my earlier paper for a full review of the results of other researchers). Finally, in S2 Text, I condense these results into the 16 novel findings that are listed in the abstract; these 16 novel findings now organize the results section in the main manuscript.

2. *Has the statistical analysis been performed appropriately and rigorously?*

*Reviewer #1: I Don’t Know*

*Reviewer #2: Yes*

Reviewer #1 answered “I don’t know” to this question. I appreciate the candor. The statistical treatments were determined before the experiments were conducted and are integral to the experimental designs. In the case of the second experiment, where the outcome deviated from the assumptions of normality, I did not perform the original statistical analysis, or any statistical analysis, but reported the deviation from normality as an observation. The failure of a large, replicated experiment can be instructive, particularly in designing other experiments to investigate the circumstances surrounding the failure. In this body of work, I used the failure of the second experiment to design the third experiment. In the first and third experiments, the outcomes were analyzed appropriately based on the experimental designs described in the methods section and reported in detail in the supporting information.

3. *Have the authors made all data underlying the findings in their manuscript fully available?*

*Reviewer #1: Yes*

*Reviewer #2: Yes*

The three Excel files of the raw data are included as supporting information.

4. *Is the manuscript presented in an intelligible fashion and written in standard English?*

*Reviewer #1: No*

*Reviewer #2: Yes*

Reviewer #1 answered “No” to this question. There were no specific issues listed, but there were comments to the author in section 5.

5. *Review Comments to the Author*

*Reviewer #1: There are too many raw data and too detail unnecessary duplicated information and the manuscript needs to have more concentration on the major objectives. The paper is too long with any unnecessary description. It is difficult for readers to catch the main ideas and what is the main findings after carefully reading for a couple of times. The paper needs to be rewritten and follow the PLoS one format.*

I take full responsibility for this criticism. All of my pre-submission reviewers were familiar with the experimental design and with mosquito biology, with two exceptions. The two pre-submission reviewers that did not have prior knowledge of the experiment caused me to add more detail about the mosquito larval life cycle and to explain the experimental analysis and statistical treatment more clearly. The comments by reviewer #1 showed me that I needed to explain my points better, and I rewrote the entire Results section to focus on the 16 major novel findings about Aedes aegypti larval biology. Although this reorganization of the content results in some further duplication because the statistical results support multiples of the 16 findings and have to be referenced under each of them, I think that the manuscript is much improved by the reorganization.

I am open to further modifications if there are outstanding issues that I haven’t addressed. I strongly believe that peer review improves the quality of manuscripts and of science in general.

*Reviewer #2: How authors determined the food competition among different life stages (1^st^-4^th^ instar) of Aedes population during experiment conditions. Have they synchronized the colony? Explain. What are behavioural changed were observed while shortage of food among both male and female population?*

These are great questions.

*How authors determined the food competition among different life stages (1^st^-4^th^ instar) of Aedes population during experiment conditions.*

Food competition among mosquito larvae has been the subject of many papers. I published an earlier paper in PLoS ONE (Steinwascher, 11 15 2018) that describes food competition in great detail. In the current manuscript, I used the results of the prior paper to set up a 2x2 grid that resulted in four competitive environments (food x density) and crossed this with 2 other factors, aliquot and timespan, that affected the availability of food on a daily basis. It isn’t possible to measure the trajectory of growth of the larvae without handling them, and it isn’t possible to sex the larvae visually until the very end of the 4^th^ instar or pupation. I used pupation to measure the outcome of growth of the larvae and calculated 7 dependent variables for each replicate (survival, pupal masses and ages of males and females). The 7 dependent variables, plus some post analytic comparisons (growth rate, size distributions) allowed me to infer the effects of the independent factors on the food competition and growth processes that occurred in the microcosms. I could not measure the growth of the early instars and the timing of the molts from 1^st^ to 2^nd^, etc. However, growth is generally accepted to resemble a sigmoid curve when mass is plotted against time and this model fits the data that I observed. The mechanics of filter feeding have been studied by Karen Porter among others, in the 1970s, and although I did not measure the filtering ability directly, the results of their experiments fit the data that I observed.

*Have they synchronized the colony? Explain.*

I fed the colony on a mouse or chick approximately once a week over several months. There was always an oviposition site available and the filter papers from the oviposition jars were changed once or twice a week. These filter papers with eggs attached were stored in an insectary until it was time to regenerate the colony or to conduct an experiment. They were then hatched and used for an experiment; the extra larvae were raised in batch trays and released into a new colony cage. This procedure was to make sure that I had enough eggs to hatch for each experiment, but it effectively resulted in synchronizing the colony. I have added a description of this to the expanded Methods section in the supporting information (S1 Text).

*What are behavioural changed were observed while shortage of food among both male and female population?*

I couldn’t tell a male larva from a female larva in the microcosms. I also couldn’t tell individual larvae apart except for their size during the 4^th^ instar. The behavior of the larvae in the microcosms was not obviously different across treatments. There was usually an amount of yeast on the bottom of the microcosm and larvae would spend time there, presumably feeding. Other larvae would float in the water column or at the surface. I didn’t see anything that looked like physical interaction, but the larvae wriggle and may contact one another in so doing. I had no way to measure these interactions. Other people have categorized the larval activity and done time/motion studies on them. I reviewed some of this work in my prior paper.

I thank you for the opportunity to resubmit the paper and I look forward to further comments if you have any.

Sincerely;

Kurt Steinwascher
