# Supplementary material for: Competition and growth among Aedes aegypti larvae: Effects of distributing food inputs over time
Source: PLoS One. 2020 Oct 2;15(10):e0234676. doi: 10.1371/journal.pone.0234676 (PMC7531853; doi:10.1371/journal.pone.0234676)
Supplement: Attachment — Submitted filename: Response to reviewers.docx [file pone.0234676.s119.docx]

September 14, 2020

Re: PONE-D-20-16489R1. Competition and growth among *Aedes aegypti* larvae: effects of distributing food inputs over time

Dear Dr. Hwang and reviewers,

Thank you for the opportunity to further improve my submission to PLoS ONE.

I have addressed the single comment by reviewer 1 in section *6. Review Comments to the Author* below.

I have also reviewed the manuscript itself to improve grammar and clarity further.

Please let me know if there are any further concerns. I look forward to hearing from you.

Sincerely;

Kurt Steinwascher

*Reviewers' comments:

Reviewer's Responses to Questions*

***Comments to the Author*** *1. If the authors have adequately addressed your comments raised in a previous round of review and you feel that this manuscript is now acceptable for publication, you may indicate that here to bypass the “Comments to the Author” section, enter your conflict of interest statement in the “Confidential to Editor” section, and submit your "Accept" recommendation.*

*Reviewer #1: All comments have been addressed*

*2. Is the manuscript technically sound, and do the data support the conclusions?

The manuscript must describe a technically sound piece of scientific research with data that supports the conclusions. Experiments must have been conducted rigorously, with appropriate controls, replication, and sample sizes. The conclusions must be drawn appropriately based on the data presented.*

*Reviewer #1: Yes*

*3. Has the statistical analysis been performed appropriately and rigorously?*

*Reviewer #1: Yes*

*4. Have the authors made all data underlying the findings in their manuscript fully available?

The* [*PLOS Data policy*](http://www.plosone.org/static/policies.action#sharing) *requires authors to make all data underlying the findings described in their manuscript fully available without restriction, with rare exception (please refer to the Data Availability Statement in the manuscript PDF file). The data should be provided as part of the manuscript or its supporting information, or deposited to a public repository. For example, in addition to summary statistics, the data points behind means, medians and variance measures should be available. If there are restrictions on publicly sharing data—e.g. participant privacy or use of data from a third party—those must be specified.*

*Reviewer #1: Yes*

*5. Is the manuscript presented in an intelligible fashion and written in standard English?

PLOS ONE does not copyedit accepted manuscripts, so the language in submitted articles must be clear, correct, and unambiguous. Any typographical or grammatical errors should be corrected at revision, so please note any specific errors here.*

*Reviewer #1: Yes*

*6. Review Comments to the Author

Please use the space provided to explain your answers to the questions above. You may also include additional comments for the author, including concerns about dual publication, research ethics, or publication ethics. (Please upload your review as an attachment if it exceeds 20,000 characters)*

*Reviewer #1: The author did well for the revision. However, there are still too many references for this kind of study report.*

Reviewer #1 says that there are too many references. I disagree with this statement.

This is a research article rather than a review of the field, so I understand the concern that reviewer #1 is expressing. The articles cited represent studies that I found pertinent to my own results whether they supported or disagreed with my results and conclusions. I cite them because the authors deserve credit for their research and findings and their own opinions, whether they agree with my conclusions or not. I did not dissect the experimental designs, the analyses, or the logic behind the conclusions in these articles, which is what would be appropriate for a review of the field.

There are 109 citations in the article. I examined each one and they fall into largely discreet categories:

1. Prior work that leads to the current study—references 1-33 (total 33). The prior work and the supporting articles about the methods (below) are important and necessary to the foundation of the research question and the experimental design. Some of these studies go back to the 1960s and are not available electronically, but they still represent important work in the field and the authors deserve credit for identifying the questions.
2. Supporting articles about the methods—references 34-38 (total 5). (see above)
3. Studies about inter specific competition among mosquito larvae—references 40-62 (total 23). The experiments themselves are about intra specific competition (discussed in [1]), but also relate to inter specific competition. I think it is noteworthy that none of the articles on inter specific competition among *Aedes* mosquito larvae measure the evolutionary and ecological winners of this competition, but rather measure the average size and age at pupation, which represents the losers of the competition. This is the second largest group of articles after the prior work.
4. Studies on oviposition preference among adult females—reference 63-72 (total 10). The results of the three experiments suggest the relationship between the larval environment and the oviposition preferences among adult females.
5. Studies on different food sources and their effect on mosquito larval growth—references 73-88 (total 16). The studies on food sources and growth contribute to the understanding of the results of the three experiments by providing an expanded context of food sources, specifically the implication that bacteria in the gut of the larvae may influence nutrition.
6. Studies on aspects of the life history strategy of adults and larvae—references 39, 89-103 (total 16). The studies on life history strategies contribute to the understanding of the results of the three experiments in the context of the environmental, ecological and evolutionary biology of the mosquitoes.
7. Studies on physiological triggers of pupation in mosquito larvae—references 104-109 (total 6). The physiological triggers of pupation were not originally something that I thought was important, but the results indicate that it is necessary to take these into consideration. There appears to be no other explanation for differences in the size of pupae after different periods of starvation late in the larval period.

In addition, there is a large body of work on intra specific competition among mosquito larvae that I do not cite in this paper because I cited it and commented on it in a prior paper [1].

In reviewing each of the 109 references I do not find any that should not reasonably be included as references in this paper. I am willing to reconsider this if reviewer #1 has specific issues with one or more of the references.
